# Supplementary material for: Sulfenamide-enabled ortho thiolation of aryl iodides via palladium/norbornene cooperative catalysis
Source: Nat Commun. 2019 Aug 7;10:3555. doi: 10.1038/s41467-019-11398-0 (PMC6685968; doi:10.1038/s41467-019-11398-0)
Supplement: Supplementary file 1 — Supplementary Information [file 41467_2019_11398_MOESM1_ESM.pdf]

Supplementary Information

Sulfenamide-Enabled *Ortho* Thiolation of Aryl Iodides via Palladium/Norbornene Cooperative Catalysis

Li, R. et al

Supplementary Tables

Supplementary Table 1. Control experiments

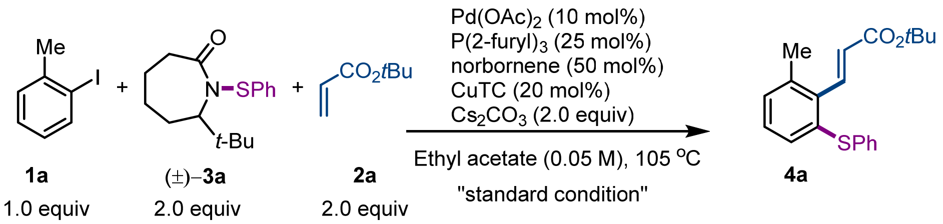

Entry	Change from the "standard condition"	Yield [%][a]
1	none	74
2	no $Pd(OAc)_2$	0
3	no norbornene	0
4	no Cs_2CO_3	6
5	no CuTC	54
6	5 mol% $Pd(OAc)_2$	15
7	$P(tBu)_3$• HBF_4 instead of P(2-furyl)$_3$	trace
8	PCy_3 instead of P(2-furyl)$_3$	0
9	XPhos instead of P(2-furyl)$_3$	7
10	PPh_3 instead of P(2-furyl)$_3$	3
11	P(4-OMeC_6H_4)$_3$ instead of P(2-furyl)$_3$	2
12	dioxane instead of EtOAc	66
13	toluene instead of EtOAc	32
14	85 °C	23

[a]Unless otherwise noted, the reaction was run with **1** (0.15 mmol), **2** (0.30 mmol), sulfur electrophile (0.30 mmol), $Pd(OAc)_2$ (0.015 mmol), P(2-furyl)$_3$ (0.0375 mmol), NBE (0.075 mmol), Cs_2CO_3 (0.30 mmol) and CuTC (0.03 mmol) in ethyl acetate (3.0 mL) at 105 °C for 12 h. The yield was determined by ^{1}H-NMR using 1,3,5-trimethoxylbenzene as the internal standard.

Supplementary Methods

General Information

Unless noted otherwise, all solvents were dried by filtration through a Pure-Solv MD-5 Solvent Purification System (Innovative Technology). Ethyl acetate was distilled freshly over calcium hydride and carefully freeze-pump-thawed. Reaction temperatures were reported as the temperatures of the bather surrounding the flasks or vials. Sensitive reagents and solvents were transferred under nitrogen into a nitrogen-filled glovebox with standard techniques. Cesium carbonate was purchased from STREM, stored and used directly in the glovebox. Analytical thin-layer chromatography (TLC) was carried out using 0.2 mm commercial silica gel plates (silica gel 60, F254, EMD chemical). Vials (15 x 45 mm 1 dram (4 mL) with PTFE lined cap attached) were purchased from Qorpak and flame-dried and cooled in a desiccator prior to usage. High resolution mass spectra (HR-MS) were recorded on an Agilent 6530 LC Q-TOF mass spectrometer using electrospray ionization with fragmentation voltage set at 115 V and processed with an Agilent MassHunter Operating System. Infrared spectra were recorded on a Nicolet 380 FTIR using neat thin film technique. Nuclear magnetic resonance spectra (^{1}H NMR and ^{13}C NMR) were recorded with a Bruker DMX 400 (400 MHz, ^{1}H at 400 MHz, ^{13}C at 101 MHz) or Bruker Model DMX 500 (500 MHz, ^{1}H at 500 MHz, ^{13}C at 126 MHz). Chemical shifts were reported in parts per million (ppm, δ), downfield from tetramethylsilane (TMS, δ=0.00ppm) and were referenced to residual solvent ($CDCl_3$, δ=7.26 ppm (^{1}H) and 77.00 ppm (^{13}C)). All the ^{19}F chemical shifts were not referenced. Coupling constants were reported in Hertz (Hz). Data for ^{1}H NMR spectra were reported as follows: chemical shift (ppm, referenced to protium, s = singlet, d = doublet, t = triplet, q = quartet, quin = quintet, dd = doublet of doublets, td = triplet of doublets, ddd = doublet of doublet of doublets, m = multiplet, coupling constant (Hz), and integration). All other materials were obtained from Sigma-Aldrich Corporation or Combi-Blocks Inc and were used as received.

Supplementary Figures

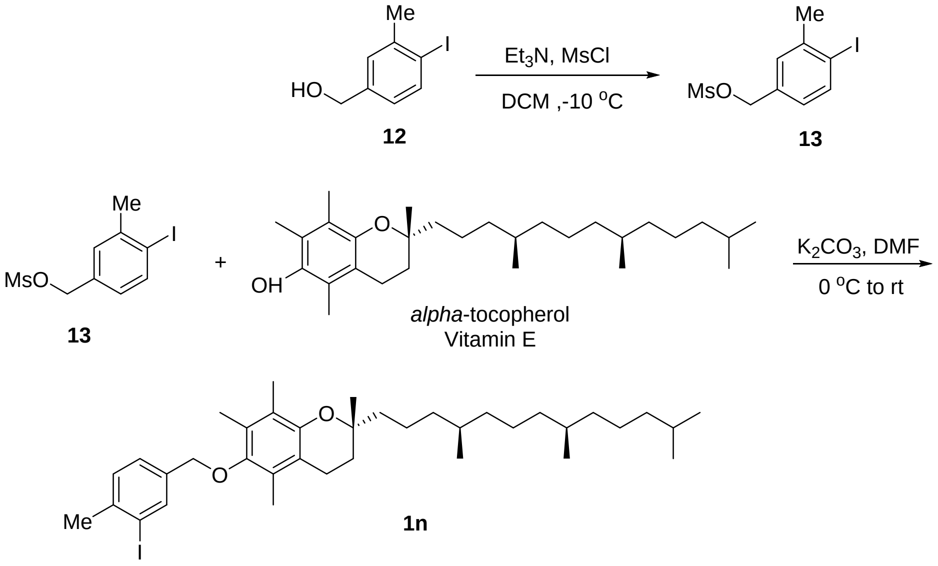

Supplementary Figure 1. Preparation of aryl iodides **1n**

A solution of **12** (2.13 g, 8.6 mmol, 1.0 equiv) and Et_3N (1.8 mL, 12.9 mmol, 1.5 equiv) in DCM (100 mL) was cooled to -10 °C using NaCl/ice cooled water bath. MsCl(1.18 g, 10.3 mmol, 1.2 equiv) was added dropwise over 5 min. The reaction mixture was stirred for 25min, maintaining a temperature between 0 and -10 °C, before pouring it into ice water (100 mL). The layers were separated and the organic layer was washed subsequently with additional ice water (100 mL), NH_4Cl solution (sat., 2x100 mL), $NaHCO_3$ solution (sat., 2x100 mL) and brine (100 mL). The resulting solution was then dried over $MgSO_4$, filtrated and concentrated under reduced pressure to give crude **13** (2.7 g, 96%).[1]

To a 40 mL vial charged with a stirred bar was added **13** (1.17 g, 3.6 mmol, 1.2 equiv) and vitamin E (1.29 g, 3.0 mmol, 1.0 equiv). 16 mL anhydrous DMF was added to the vial and the reaction was cooled at 0 ^{o}C followed by adding K_2CO_3 (829 mg, 6.0 mmol, 2.0 equiv). The reaction was then warmed to room temperature and stirred overnight. Upon completion, as judged by TLC analysis, the mixture was filtered through Celite and poured into water. The aqueous phase was extracted with Et_2O for three times and then washed with water, brine and dried over Na_2SO_4. The solvent was removed under reduced pressure and the residue was purified by silica gel chromatography to afford compound **1n** (1.3 g, 66 %) as a yellow oil.

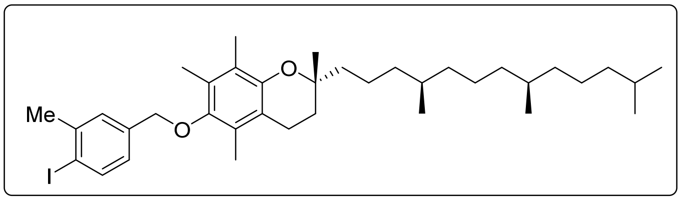

1n: Yellow oil (66%). **^{1}H NMR** (400 MHz, Chloroform-*d*) δ 7.83 (d, *J* = 8.1 Hz, 1H), 7.39 (d, *J* = 2.0 Hz, 1H), 7.02 (dd, *J* = 8.1, 2.1 Hz, 1H), 4.62 (s, 2H), 2.62 (s, 2H), 2.48 (s, 3H), 2.21 (s, 3H), 2.16 (s, 3H), 2.12 (s, 3H), 1.88 – 1.72 (m, 2H), 1.63 – 1.49 (m, 3H), 1.49 – 1.36 (m, 4H), 1.33 – 1.22 (m, 10H), 1.21 – 1.03 (m, 7H), 0.93 – 0.82 (m, 12H). **^{13}C NMR** (101 MHz, $CDCl_3$) δ 148.1, 148.1, 141.6, 139.1, 138.5, 129.0, 128.0, 126.7, 126.0, 123.1, 117.8, 100.1, 75.0, 74.0, 40.2, 39.5, 37.5, 32.9, 32.8, 31.4, 28.3, 28.1, 25.0, 25.0, 24.6, 24.0, 22.9, 22.8, 21.2, 20.8, 19.9, 19.8, 19.8, 13.0, 12.2, 12.0. **IR** (KBr): υ 2925, 2866, 1725, 1512, 1460, 1415, 1377, 1257, 1166, 1088 cm^{-1}. **HRMS** (ESI): Calculated for $C_{37}H_{58}IO_2$ $(M+H^+)$:661.3476, found:661.3475.

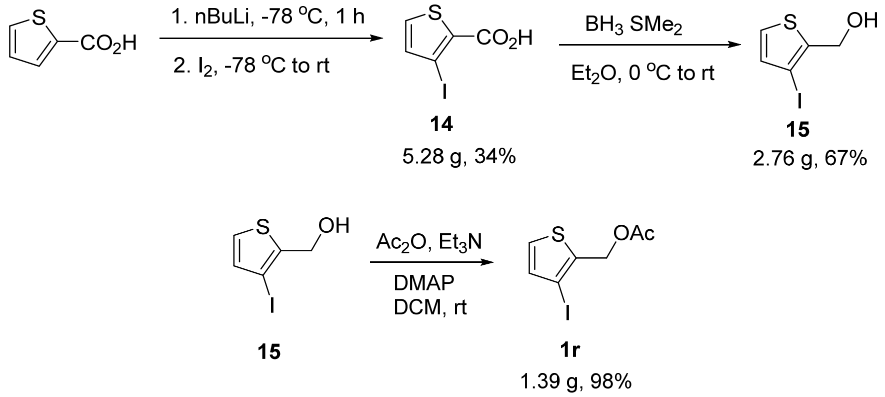

Supplementary Figure 2. Preparation of aryl iodides **1r**

14 and **15** were prepared according to the literature reported procedure.[2] To a solution of Ac_2O (766 mg, 7.5 mmol, 1.5 equiv)), Et_3N (759 mg, 7.5 mmol, 1.5 equiv) and DMAP (48.9 mg, 0.4 mmol, 0.08 equiv) in DCM (10 mL) was added **15** (1.2 g, 5 mmol, 1.0 equiv). The reaction mixture was then stirred at room temperature for 19 h. Upon completion, HCl (2M, 60 mL) was added into the reaction flask. The mixture was extracted with Et_2O and organic layers were washed with sat. $NaHCO_3$, brine and dried over $MgSO_4$. The solvent was removed under reduced pressure and the residue was purified by silica gel chromatography to afford compound **1r** (1.39 g, 98 %) as a colorless oil.

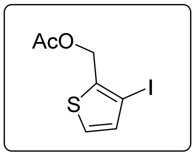

1r: Colorless oil (98%). **^{1}H NMR** (400 MHz, Chloroform-*d*) δ 7.31 (d, *J* = 5.3 Hz, 1H), 7.05 (d, *J* = 5.2 Hz, 1H), 5.22 (s, 2H), 2.11 (s, 3H). **^{13}C NMR** (101 MHz, $CDCl_3$) δ 170.7, 136.7, 135.2, 128.2, 82.5, 62.3, 21.0. **IR** (KBr): υ 3105, 2950, 1743, 1438, 1375, 1223, 1023, 857, 776, 710 cm^{-1}.

Preparation of thiolation reagent

S3-S18 were prepared according to literature reported procedure[3-8].

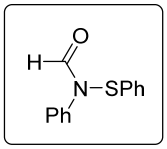

S4: Yellow oil (60%). **^{1}H NMR** (400 MHz, $CDCl_3$) δ = 8.75 (d, *J*=33.0, 1H), 7.47 – 7.25 (m, 10H). **^{13}C NMR** (101 MHz, $CDCl_3$) δ = 167.4, 163.9, 129.5, 129.1, 128.3, 127.4, 126.6, 125.6, 125.0. **IR** (KBr): υ 3060, 1696, 1593, 1489, 1440, 1253, 1126, 1024, 739, 689 cm^{-1}. **HRMS** (ESI): Calculated for $C_{13}H_{12}NOS$ ($M+H^+$): 230.0634, found: 230.0632.

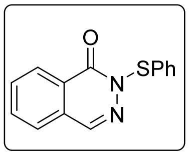

S6: White solid (52%). **^{1}H NMR** (400 MHz, $CDCl_3$) δ = 8.40 – 8.32 (m, 1H), 8.12 (s, 1H), 7.73 (dtd, *J*=16.6, 7.4, 1.4, 2H), 7.64 – 7.58 (m, 1H), 7.58 – 7.48 (m, 2H), 7.29 – 7.19 (m, 3H). **^{13}C NMR** (101 MHz, $CDCl_3$) δ = 161.1, 141.0, 135.9, 134.1, 132.3, 130.0, 129.6, 129.4, 129.1, 128.0, 127.9, 126.4. **IR** (KBr): υ 3058, 1674, 1594, 1475, 1440, 1321, 1287, 1232, 1136, 1051 cm^{-1}. **HRMS** (ESI): Calculated for $C_{14}H_{10}N_2OS$ (M^+): 254.0508, found: 254.0517.

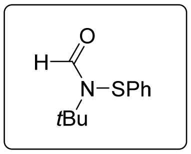

S5: Colorless oil (56%). **^{1}H NMR** (400 MHz, $CDCl_3$) δ = 8.58 (s, 1H), 7.33 – 7.09 (m, 5H), 1.40 (s, 9H). **^{13}C NMR** (101 MHz, $CDCl_3$) δ = 170.0, 164.0, 129.2, 127.0, 124.5, 61.5, 28.8. **IR** (KBr): υ 2976, 1689, 1582, 1478, 1440, 1366, 1258, 1207, 1146, 740 cm^{-1}. **HRMS** (ESI): Calculated for $C_{11}H_{15}NOSNa$ ($M+Na^+$): 232.0764, found: 232.0774.

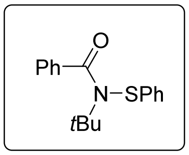

S7: Colorless oil (50%). **^{1}H NMR** (400 MHz, $CDCl_3$) δ = 7.43 – 7.37 (m, 2H), 7.26 – 7.17 (m, 5H), 7.11 – 7.04 (m, 3H), 1.51 (s, 9H). **^{13}C NMR** (101 MHz, $CDCl_3$) δ = 178.0, 141.0, 138.6, 129.8, 129.0, 127.8, 127.0, 126.3, 123.9, 63.4, 29.3. **IR** (KBr): υ 3059, 2975, 1663, 1581, 1478, 1393, 1363, 1287, 1187, 1117 cm^{-1}. **HRMS** (ESI): Calculated for $C_{17}H_{19}NOSNa$ ($M+Na^+$): 308.1080, found: 308.1090.

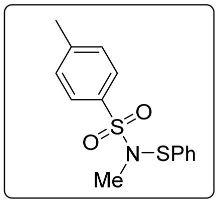

S10: White solid (43%). **^{1}H NMR** (400 MHz, $CDCl_3$) δ = 7.85 – 7.80 (m, 2H), 7.46 – 7.41 (m, 2H), 7.39 – 7.33 (m, 4H), 7.30 – 7.24 (m, 1H), 3.29 (s, 3H), 2.46 (s, 3H). **^{13}C NMR** (101 MHz, $CDCl_3$) δ = 144.2, 136.8, 135.3, 129.8, 129.2, 127.8, 127.7, 126.4, 42.5, 21.7. **IR** (KBr): υ 1580, 1437, 1350, 1302, 1164, 1088, 849, 819, 739, 707, 678 cm^{-1}. **HRMS** (ESI): Calculated for $C_{14}H_{15}NO_2S_2$ (M^+): 293.0539, found: 293.0544.

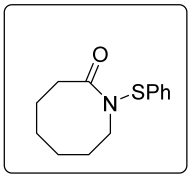

S14: Yellow solid (36%). **^{1}H NMR** (400 MHz, $CDCl_3$) δ = 7.37 – 7.29 (m, 4H), 7.26 – 7.20 (m, 1H), 3.91 – 3.74 (m, 2H), 2.79 – 2.67 (m, 2H), 1.87 (m, *J* =10.0, 8.8, 6.1, 2H), 1.75 (dt, *J* =11.8, 6.0, 2H), 1.58 (dt, *J* =12.1, 6.0, 2H), 1.49 (dt, *J* =10.2, 6.1, 2H). **^{13}C NMR** (101 MHz, $CDCl_3$) δ = 177.5, 137.4, 129.1, 127.2, 126.8, 53.5, 34.5, 29.6, 29.0, 26.1, 24.2. **IR** (KBr): υ 2926, 1664, 1477, 1439, 1374, 1245, 1120, 1084, 738, 690 cm^{-1}. **HRMS** (ESI): Calculated for $C_{13}H_{17}NOS$ (M^+): 235.1025, found: 235.1017.

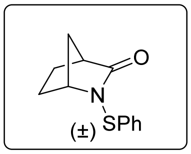

S15: Yellow solid (83%). **^{1}H NMR** (400 MHz, $CDCl_3$) δ = 7.36 – 7.27 (m, 4H), 7.26 – 7.19 (m, 1H), 3.99 (s, 1H), 2.96 (dd, *J*=2.7, 1.2, 1H), 2.13 – 2.01 (m, 1H), 1.98 – 1.88 (m, 1H), 1.81 (ddd, *J*=9.9, 5.8, 1.4, 1H), 1.76 – 1.62 (m, 2H), 1.49 (d, *J*=9.7, 1H). **^{13}C NMR** (101 MHz, $CDCl_3$) δ = 180.3, 137.9, 129.2, 127.3, 126.4, 66.4, 45.2, 40.1, 28.5, 24.3. **IR** (KBr): υ 2951, 2875, 1722, 1581, 1476, 1439, 1331, 1209, 1141, 1101 cm^{-1}. **HRMS** (ESI): Calculated for $C_{12}H_{13}NOS$ (M^+): 219.0712, found: 219.0720.

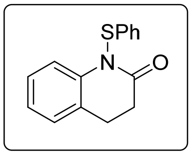

S17: Yellow solid (21%). **^{1}H NMR** (400 MHz, $CDCl_3$) δ = 7.57 (dd, *J*=8.2, 0.7, 1H), 7.24 – 7.09 (m, 7H), 6.97 (td, *J*=7.4, 1.0, 1H), 2.92 (dd, *J*=8.6, 5.6, 2H), 2.85 – 2.76 (m, 2H). **^{13}C NMR** (101 MHz, $CDCl_3$) δ = 172.0, 142.1, 137.1, 129.3, 127.8, 127.8, 127.4, 127.0, 124.9, 124.1, 118.5, 33.2, 25.6. **IR** (KBr): υ 2759, 1701, 1603, 1582, 1485, 1457, 1438, 1347, 1292, 1251 cm^{-1}. **HRMS** (ESI): Calculated for $C_{15}H_{13}NOSNa$ ($M+Na^+$): 278.0610, found: 278.0618.

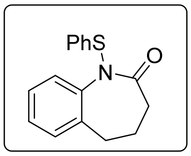

S18: Yellow solid (80%). **^{1}H NMR** (400 MHz, $CDCl_3$) δ = 7.47 (dd, *J*=8.0, 0.9, 1H), 7.44 – 7.38 (m, 2H), 7.36 – 7.24 (m, 4H), 7.15 (dtd, *J*=9.0, 7.5, 1.4, 2H), 2.60 (t, *J*=6.9, 2H), 2.49 (t, *J*=6.9, 2H), 2.18 (s, 2H). **^{13}C NMR** (101 MHz, $CDCl_3$) δ = 174.9, 145.2, 137.5, 134.6, 129.2, 128.9, 128.1, 128.0, 127.8, 127.0, 124.3, 33.50, 29.90, 28.4. **IR** (KBr): υ 2946, 1694, 1580, 1484, 1455, 1338, 1304, 1265, 1222, 1140 cm^{-1}. **HRMS** (ESI): Calculated for $C_{16}H_{15}NOS$ (M^+): 269.0874, found: 269.0872.

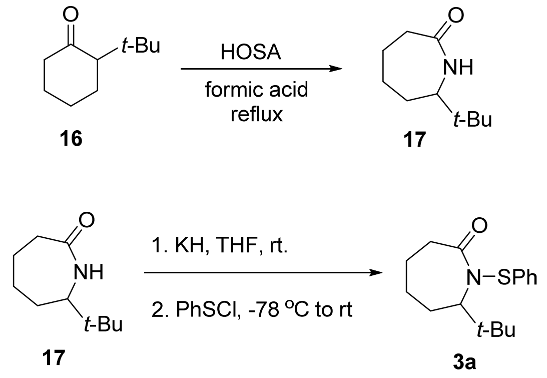

Supplementary Figure 3. Preparation of thiolation reagent **3a**

The seven-membered lactam was prepared according to the literature reported procedure.[9] In a 100 ml Schlenk flask, dry potassium hydride solid (0.48 g, 12 mmol, 1.2 equiv) was suspended in 15 ml dry THF solution. A 10 ml THF solution of the corresponding amide (10 mmol) was added dropwise at room temperature. The resulting solution was stirred at room temperature for two hours until no more hydrogen gas was released. The solution was cooled to -78℃ before a 10 ml THF solution of the corresponding sulfenyl chloride (freshly distilled) was added dropwise.[10] The resulting solution was allowed to warm up to room temperature slowly and stirred overnight. The reaction was quenched by 10% citric acid (50 ml), and the aqueous layer was extracted three times with ethyl acetate (75 ml X 3). The combined organic phases were washed with sodium bicarbonate solution (100 ml), water (100 ml) and brine. The mixture was dried over $MgSO_4$ and the solvent was removed under reduced pressure. The residue was purified by silica gel chromatography to afford the corresponding sulfenamide compound.

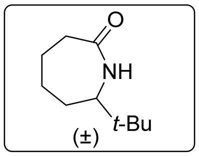

S17: White solid (59%). **^{1}H NMR** (400 MHz, Chloroform-*d*) δ 5.57 (s, 1H), 3.00 (ddd, *J* = 9.8, 5.9, 1.0 Hz, 1H), 2.55 – 2.45 (m, 1H), 2.44 – 2.36 (m, 1H), 1.99 (dddq, *J* = 10.2, 4.3, 2.9, 1.5 Hz, 1H), 1.91 (ddd, *J* = 13.7, 4.5, 3.0 Hz, 1H), 1.87 – 1.80 (m, 1H), 1.59 – 1.40 (m, 2H), 1.19 (dddd, *J* = 13.8, 12.5, 9.6, 3.1 Hz, 1H), 0.93 (s, 9H). **^{13}C NMR** (101 MHz, $CDCl_3$) δ 177.5, 63.0, 36.4, 33.6, 30.4, 29.9, 26.4, 23.4. **IR** (KBr): υ 3216, 3067, 2941, 2863, 1652, 1443, 1415, 1372, 1344, 1190 cm^{-1}. HRMS (ESI): Calculated for $C_{10}H_{19}NONa$(M+Na$^+$):192.1359, found:192.1367. Both ^{1}H NMR and ^{13}C NMR match the literature reported data.[11]

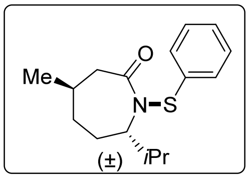

S19: White solid (74%). Mp = 96.1 – 96.6 °C. **^{1}H NMR** (400 MHz, Chloroform-*d*) δ 7.47 (d, *J* = 7.3 Hz, 2H), 7.29 (td, *J* = 7.3, 1.1 Hz, 2H), 7.25 – 7.19 (m, 1H), 3.57 (ddd, *J* = 10.3, 8.1, 2.7 Hz, 1H), 2.73 (dd, *J* = 13.6, 3.7 Hz, 1H), 2.63 (dd, *J* = 13.7, 6.8 Hz, 1H), 2.31 (dp, *J* = 9.2, 6.6 Hz, 1H), 2.06 – 1.93 (m, 1H), 1.91 – 1.73 (m, 2H), 1.54 (dt, *J* = 15.9, 8.5 Hz, 1H), 1.35 – 1.21 (m, 1H), 0.95 (dd, *J* = 12.4, 6.8 Hz, 9H). **^{13}C NMR** (101 MHz, $CDCl_3$) δ 176.5, 129.2, 128.9, 127.9, 72.8, 43.2, 31.5, 30.2, 28.1, 26.7, 20.8, 19.7. **IR** (KBr): υ 3751, 3650, 2961, 2870, 1793, 1701, 1654, 1533, 1457, 1388 cm^{-1}. **HRMS** (ESI): Calculated for $C_{16}H_{23}NOSNa$(M+Na$^+$): 300.1393, found: 300.1387.

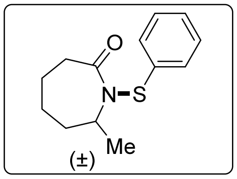

S21: Orange solid (76%). Mp = 96.1 – 96.6 °C. **^{1}H NMR** (400 MHz, Chloroform-*d*) δ 7.27 (d, *J* = 1.4 Hz, 4H), 7.17 (ddt, *J* = 7.1, 5.6, 2.2 Hz, 1H), 4.24 – 4.11 (m, 1H), 2.88 (ddd, *J* = 14.5, 8.9, 3.2 Hz, 1H), 2.75 (ddd, *J* = 14.1, 8.4, 3.0 Hz, 1H), 1.86 – 1.52 (m, 6H), 1.37 (d, *J* = 6.9 Hz, 3H). **^{13}C NMR** (101 MHz, $CDCl_3$) δ 178.4, 139.5, 129.1, 126.7, 125.8, 60.2, 37.3, 35.8, 26.5, 23.1, 20.7. **IR** (KBr): υ 2973, 2931, 2858, 1673, 1477, 1439, 1293, 1185, 739, 690 cm^{-1}. **HRMS** (ESI): Calculated for $C_{13}H_{17}NOSNa$(M+Na$^+$): 258.0923, found: 258.0930.

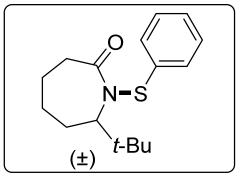

3a: Yellow oil (82%). **^{1}H NMR** (400 MHz, Chloroform-*d*) δ 7.51 – 7.45 (m, 2H), 7.33 – 7.23 (m, 3H), 3.73 (dd, *J* = 9.8, 6.8 Hz, 1H), 2.84 – 2.70 (m, 1H), 2.63 (d, *J* = 13.7 Hz, 1H), 1.82 – 1.69 (m, 2H), 1.68 – 1.56 (m, 2H), 1.50 (dt, *J* = 23.5, 8.8 Hz, 1H), 1.27 – 1.12 (m, 1H), 1.06 (s, 9H). **^{13}C NMR** (101 MHz, $CDCl_3$) δ 177.8, 138.0, 130.3, 128.9, 128.3, 74.8, 37.4, 34.6, 28.0, 26.4, 22.6. **IR** (KBr): υ 2950, 2868, 1670, 1478, 1439, 1401, 1275, 1164, 1080, 1024 cm^{-1}. **HRMS** (ESI): Calculated for $C_{16}H_{23}NOS(M^+)$: 300.1495, found: 300.1487.

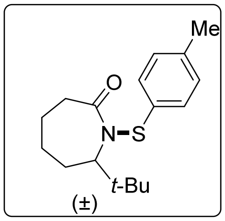

3b: Orange oil (82%). **^{1}H NMR** (400 MHz, $CDCl_3$) δ 7.42 (d, *J* = 8.1 Hz, 2H), 7.08 (d, *J* = 7.9 Hz, 2H), 3.80 – 3.64 (m, 1H), 2.74 – 2.48 (m, 2H), 2.29 (s, 3H), 1.59 (m, 5H), 1.03 (s, 10H). **^{13}C NMR** (101 MHz, $CDCl_3$) δ 177.5, 138.9, 134.2, 131.6, 129.6, 74.7, 37.3, 34.3, 27.8, 26.1, 22.5, 21.3. **IR** (KBr): υ 2950, 2868, 1669, 1491, 1479, 1401, 1366, 1275, 1164, 1141 cm^{-1}. **HRMS** (ESI): Calculated for $C_{17}H_{25}NOS$ (M^+):291.1651, found: 291.1659.

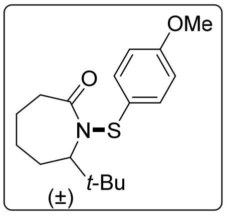

3c: Yellow solid (67%). **^{1}H NMR** (400 MHz, $CDCl_3$) δ 7.66 – 7.58 (m, 2H), 6.88 – 6.78 (m, 2H), 3.84 – 3.78 (m, 3H), 3.75 (dd, *J* = 12.6, 6.3 Hz, 1H), 2.66 (ddd, *J* = 10.3, 9.6, 4.5 Hz, 1H), 2.52 (s, 1H), 1.84 – 1.31 (m, 6H), 1.07 (s, 9H). **^{13}C NMR** (101 MHz, $CDCl_3$) δ 177.4, 160.8, 135.5, 128.5, 114.4, 114.4, 74.2, 55.3, 37.2, 34.2, 27.7, 25.8, 22.5. **IR** (KBr): υ 2950, 2868, 1664, 1590, 1493, 1428, 1172, 1140, 1028, 831 cm^{-1}. **HRMS** (ESI): Calculated for $C_{17}H_{25}NO_2S$ (M^+): 307.1601, found: 307.1603.

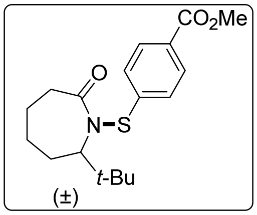

3d: Orange oil (30%). **^{1}H NMR** (400 MHz, $CDCl_3$) δ 8.00 – 7.88 (m, 2H), 7.31 (d, *J* = 8.4 Hz, 2H), 3.88 (s, 3H), 3.71 (dd, *J* = 9.6, 5.9 Hz, 1H), 2.78 (dt, *J* = 12.4, 7.4 Hz, 2H), 1.90 – 1.68 (m, 4H), 1.55 (dd, *J* = 22.4, 10.6 Hz, 1H), 1.42 (m, 1H), 1.04 (s, 9H). **^{13}C NMR** (101 MHz, $CDCl_3$) δ 178.1, 166.7, 144.7, 130.0, 128.4, 126.3, 74.7, 52.2, 37.1, 34.8, 28.1, 27.2, 23.4, 22.7. **IR** (KBr): υ 2951, 2869, 1720, 1674, 1593, 1479, 1436, 1399, 1276, 1177 cm^{-1}. **HRMS** (ESI): Calculated for $C_{18}H_{25}NO_3SNa(M+Na^+)$: 358.1447, found: 358.1449.

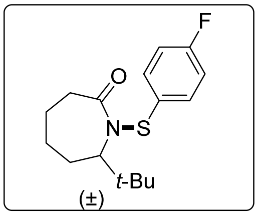

3e: Orange oil (85%). **^{1}H NMR** (400 MHz, $CDCl_3$) δ 8.28 – 8.06 (m, 2H), 7.37 – 7.23 (m, 2H), 3.83 – 3.67 (m, 1H), 2.85 (s, 2H), 1.75 (m, 6H), 1.08 (s, 9H). **^{13}C NMR** (101 MHz, Chloroform-*d*) δ 177.43, 163.12 (d, *J* = 249.5 Hz), 134.22 (d, *J* = 6.5 Hz), 132.97 (d, *J* = 2.8 Hz), 74.89, 37.25, 34.22, 27.81, 26.07, 22.43. **IR** (KBr): υ 2951, 2869, 1668, 1587, 1489, 1394, 1275, 1223, 1164, 1141 cm^{-1}. **HRMS** (ESI): Calculated for $C_{16}H_{22}FNOSNa$ $(M+Na^+)$: 318.1298, found: 318.1295.

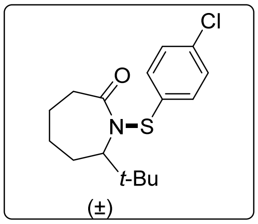

3f: Orange oil (74%). **^{1}H NMR** (400 MHz, $CDCl_3$) δ 7.44 (d, *J* = 8.5 Hz, 2H), 7.31 – 7.25 (m, 2H), 3.72 (dd, *J* = 9.8, 6.8 Hz, 1H), 2.80 – 2.55 (m, 2H), 1.85 – 1.42 (m, 5H), 1.20 (m, 1H), 1.05 (s, 9H). **^{13}C NMR** (101 MHz, $CDCl_3$) δ 177.8, 136.4, 134.4, 131.7, 129.1, 74.8, 37.2, 34.4, 27.9, 26.4, 22.5. **IR** (KBr): υ 2950, 2868, 1670, 1570, 1474, 1401, 1275, 1260, 1163, 1114 cm^{-1}. **HRMS** (ESI): Calculated for $C_{16}H_{22}ClNOS(M^+)$: 311.1105, found: 311.1104.

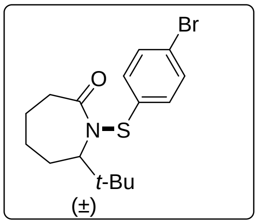

3g: Orange oil (70%). **^{1}H NMR** (400 MHz, $CDCl_3$) δ 7.43 – 7.38 (m, 2H), 7.33 (d, *J* = 8.5 Hz, 2H), 3.69 (dd, *J* = 9.8, 6.7 Hz, 1H), 2.77 – 2.5zxcVds5 (m, 2H), 1.83 – 1.40 (m, 5H), 1.22 (m, 1H), 1.03 (s, 9H). **^{13}C NMR** (101 MHz, $CDCl_3$) δ 177.9, 137.2, 132.1, 131.8, 122.5, 75.0, 37.3, 34.6, 28.1, 26.6, 22.7. **IR** (KBr): υ 2950, 2868, 1670, 1472, 1401, 1275, 1260, 1230, 1163, 1008 cm^{-1}. **HRMS** (ESI): Calculated for $C_{16}H_{22}BrNOSNa(M+Na^+)$: 378.0498, found: 378.0506.

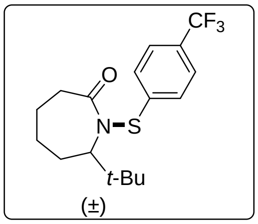

3h: Orange oil (87%). **^{1}H NMR** (400 MHz, 126.9, 125.7 (q, *J* = 3.8 Hz), δ 124.1 (q, *J* = 272.0 Hz). 74.7, 37.1, 34.8, 28.0, 27.2, 23.6, 22.6. **IR** (KBr): υ $CDCl_3$) δ 7.52 (d, *J* = 8.4 Hz, 2H), 7.37 (d, *J* = 8.2 Hz, 2H), 3.71 (dd, *J* = 9.6, 6.0 Hz, 1H), 2.87 – 2.66 (m, 2H), 1.90 – 1.62 (m, 4H), 1.55 gi(dd, *J* = 22.9, 10.9 Hz, 1H), 1.45 – 1.32 (m, 1H), 1.04 (s, 9H). **^{13}C NMR** (101 MHz, Chloroform-*d*) δ 178.0, 143.3, 128.9 (q, *J* = 32.7 Hz), 2953, 2870, 1675, 1605, 1479, 1400, 1326, 1164, 1123, 1088 cm^{-1}. **HRMS** (ESI): Calculated for $C_{17}H_{22}F_3NOS(M^+)$: 345.1369, found: 345.1361.

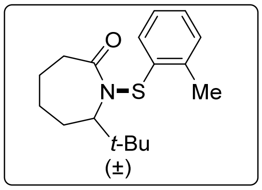

3i: Yellow solid (87%). Mp = 95.5 – 96.3 °C. **^{1}H NMR** (400 MHz, $CDCl_3$) δ 7.28 – 7.03 (m, 4H), 3.72 (dd, *J* = 9.6, 6.7 Hz, 1H), 2.82 – 2.62 (m, 2H), 2.36 (s, 3H), 1.88 – 1.56 (m, 5H), 1.46 – 1.31 (m, 1H), 1.06 (s, 9H). **^{13}C NMR** (101 MHz, $CDCl_3$) δ 177.9, 136.9, 135.1, 130.2, 128.2, 126.8, 126.4, 74.8, 37.4, 34.7, 28.2, 26.9, 19.8. **IR** (KBr): υ

2951, 2868, 1670, 1589, 1466, 1401, 1275, 1230, 1164, 1141 cm^{-1}. **HRMS** (ESI): Calculated for $C_{17}H_{25}NOS(M^+)$:291.1651, found:291.1645.

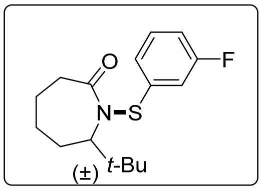

3j: Orange oil (81%). **^{1}H NMR** (400 MHz, $CDCl_3$) δ 7.34 – 7.08 (m, 3H), 6.92 (tdd, *J* = 8.4, 2.5, 1.0 Hz, 1H), 3.72 (dd, *J* = 9.7, 6.4 Hz, 1H), 2.87 – 2.60 (m, 2H), 1.88 – 1.62 (m, 4H), 1.55 (d, *J* = 10.8 Hz, 1H), 1.37 – 1.19 (m, 1H), 1.05 (s, 9H). **^{13}C NMR** (101 MHz, Chloroform-*d*) δ 177.9, 162.6 (d, *J* = 249.0 Hz), 140.4 (d, *J* = 7.2 Hz), 124.2, 115.7 (d, *J* = 23.4 Hz), 114.7 (d, *J* = 21.3 Hz), 74.8, 37.1, 34.6, 28.0, 26.7, 23.0, 22.6. **IR** (KBr): υ 2952, 2869, 1671, 1598, 1579, 1472, 1402, 1367, 1262, 1215 cm^{-1}. **HRMS** (ESI): Calculated for $C_{16}H_{22}FNOSNa(M+Na^+)$: 318.1298, found: 318.1303.

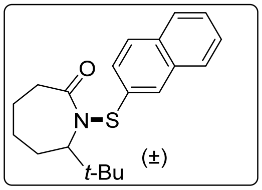

3k: Orange oil (81%). **^{1}H NMR** (400 MHz, $CDCl_3$) δ 7.93 (s, 1H), 7.79 (ddd, *J* = 8.8, 5.7, 3.3 Hz, 3H), 7.59 (dd, *J* = 8.6, 1.8 Hz, 1H), 7.52 – 7.43 (m, 2H), 3.77 (dd, *J* = 9.6, 7.0 Hz, 1H), 2.91 – 2.59 (m, 2H), 1.81 – 1.45 (m, 5H), 1.22 – 1.02 (m, 10H). **^{13}C NMR** (101 MHz, $CDCl_3$) δ 178.0, 135.4, 133.4, 133.0, 129.1, 128.7, 128.0, 127.8, 126.7, 126.6, 74.7, 37.4, 34.6, 28.0, 26.5, 22.7. **IR** (KBr): υ 2950, 2868, 1667, 1624, 1500, 1478, 1402, 1366, 1275, 1164 cm^{-1}. **HRMS** (ESI): Calculated for $C_{20}H_{25}NOS(M^+)$: 327.1651, found: 327.1647.

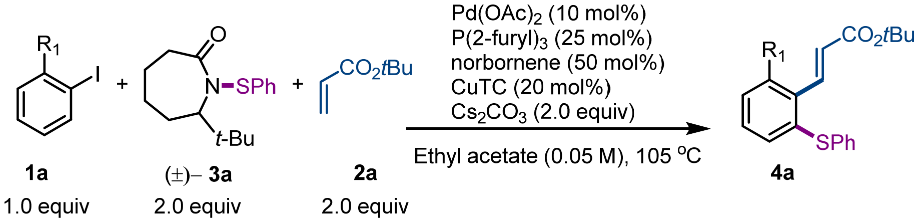

Supplementary Figure 4. Palladium/norbornene-catalyzed *ortho* thiolation reaction

A flame-dried 7.0 mL vial A was charged with $Pd(OAc)_2$ (4.6 mg, 0.02 mmol, 10 mol%), CuTC (7.6 mg, 0.04 mmol, 20 mol%), TFP (11.6 mg, 0.05 mmol, 25 mol%) and ArI (0.2 mmol, 1.0 equiv). To another 4.0 mL vial B was weighed the thiolation reagent (0.6 mmol). The two vials were directly transferred into a nitrogen-filled glovebox without caps. Then, Cs_2CO_3 (130.4 mg, 0.4 mmol, 2.0 equiv) was added to vial A. In the third empty 4.0 mL vial C, NBE (18.8 mg, 0.2 mmol) was dissolved in 1.0 mL dry ethyl acetate. Half of this NBE solution (0.5 mL, 0.1 mmol, 50 mol%) was transferred into vial A. To the 4.0 mL vial B containing thiolation reagent was added 0.75 mL dry ethyl acetate. Two thirds of this thiolation reagent solution (0.5 mL, 0.4 mmol, 2.0 equiv) was transferred into vial A, before another 3.0 mL dry ethyl acetate was added. After acrylate **2** (0.4 mmol, 2.0 equiv) was added, vial A was tightly sealed, transferred out of glovebox and stirred on a pie-block preheated to 105 ^{o}C for 12 hours. After completion of the reaction, the mixture was filtered through a thin pad of silica gel. The filter cake was washed with ethyl acetate and the combined filtrate was concentrated. The residue was loaded to a small amount of silica gel and subjected to flash column chromatography to give the desired *ortho* thiolation product.

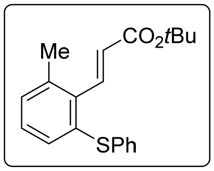

4a: Colorless oil (75%). R_f = 0.3 (hexane/ethyl acetate = 20:1). **^{1}H NMR** (400 MHz, $CDCl_3$) δ 7.83 (d, *J* = 16.3 Hz, 1H), 7.31 – 7.22 (m, 5H), 7.11 (d, *J* = 1.6 Hz, 3H), 6.01 (d, *J* = 16.4 Hz, 1H), 2.38 (s, 3H), 1.52 (s, 9H). **^{13}C NMR** (101 MHz, $CDCl_3$) δ 165.9, 141.0, 137.6, 136.2, 135.9, 135.6, 131.7, 129.7, 129.3, 128.6, 127.3, 126.8, 80.6, 28.3, 21.5. **IR** (KBr): υ 3057, 2977, 2930, 1711, 1639, 1583, 1478, 1367, 1314, 1152 cm^{-1}. **HRMS** (ESI): Calculated for $C_{20}H_{23}O_2S$ ($M+H^+$): 327.1413, found: 327.1403.

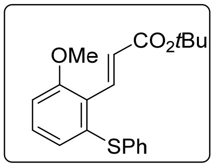

4b: Colorless oil (71%). R_f = 0.2 (hexane/ethyl acetate = 20:1). **^{1}H NMR** (400 MHz, $CDCl_3$) δ 8.08 (d, *J* = 16.1 Hz, 1H), 7.32 – 7.21 (m, 5H), 7.17 (t, *J* = 8.1 Hz, 1H), 6.89 (dd, *J* = 7.9, 1.1 Hz, 1H), 6.83 (d, *J* = 8.3 Hz, 1H), 6.67 (d, *J* = 16.1 Hz, 1H), 3.89 (s, 3H), 1.51 (s, 9H). **^{13}C NMR** (101 MHz, $CDCl_3$) δ 167.2, 159.5, 139.0, 137.3, 135.5, 131.6,

130.0, 129.3, 127.4, 125.6, 124.8, 124.3, 110.0, 80.2, 55.8, 28.4. **IR** (KBr): υ 2976, 2935, 1704, 1624, 1462, 1433, 1312, 1266, 1150, 1041 cm^{-1}. **HRMS** (ESI): Calculated for $C_{20}H_{22}O_3S$ Na(M+Na$^+$): 365.1182, found: 365.1185.

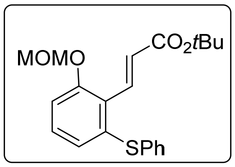

4c: White solid (52%). R$_f$ = 0.2 (hexane/ethyl acetate = 20:1). Mp = 96.4 – 97.2 °C. **^{1}H NMR** (400 MHz, Chloroform-*d*) δ 8.04 (d, *J* = 16.1 Hz, 1H), 7.34 – 7.27 (m, 4H), 7.26 – 7.22 (m, 1H), 7.14 (dd, *J* = 8.3, 7.7 Hz, 1H), 7.07 (ddd, *J* = 8.3, 1.3, 0.5 Hz, 1H), 6.92 (dd, *J* = 7.7, 1.3 Hz, 1H), 6.62 (d, *J* = 16.1 Hz, 1H), 5.25 (s, 2H), 3.49 (s, 3H), 1.51 (s, 9H). **^{13}C NMR** (101 MHz, $CDCl_3$) δ 167.0, 157.0, 138.8, 137.3, 135.3, 131.7, 130.0, 129.4, 127.4, 125.9, 125.8, 125.0, 113.6, 94.7, 80.4, 28.3. **IR** (KBr): υ 2977, 2932, 1705, 1626, 1565, 1455, 1367, 1312, 1254, 1150 cm^{-1}. **HRMS** (ESI): Calculated for $C_{21}H_{24}O_4S$ Na(M+Na$^+$): 395.1288, found: 395.1294.

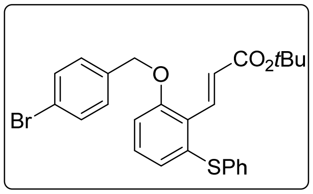

4d: White solid (57%). R$_f$ = 0.2 (hexane/ethyl acetate = 20:1). Mp = 129.5 – 130.1 °C. **^{1}H NMR** (400 MHz, Chloroform-*d*) δ 8.06 (d, *J* = 16.1 Hz, 1H), 7.54 – 7.47 (m, 2H), 7.35 – 7.26 (m, 7H), 7.12 (t, *J* = 8.1 Hz, 1H), 6.87 (dd, *J* = 7.9, 1.0 Hz, 1H), 6.80 (dt, *J* = 8.3, 0.8 Hz, 1H), 6.67 (d, *J* = 16.2 Hz, 1H), 5.12 (s, 2H), 1.50 (s, 9H). **^{13}C NMR** (101 MHz, $CDCl_3$) δ 166.8, 157.9, 139.2, 136.8, 135.4, 135.0, 131.8, 131.8, 129.8, 129.3, 128.8, 127.4, 126.0, 124.9, 124.5, 122.0, 111.3, 80.2, 70.0, 28.2. **IR** (KBr): υ 2976, 1704, 1627, 1581, 1449, 1367, 1312, 1267, 1150, 1071 cm^{-1}. **HRMS** (ESI): Calculated for $C_{26}H_{25}BrO_3S$ Na(M+Na$^+$):519.0600, found:519.0595.

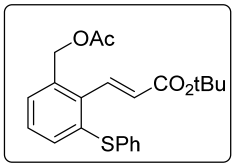

4e: White solid (74%). R$_f$ = 0.2 (hexane/ethyl acetate = 10:1). Mp = 89.1 – 90.3 °C. **^{1}H NMR** (400 MHz, Chloroform-*d*) δ 7.81 (d, *J* = 16.2 Hz, 1H), 7.36 – 7.26 (m, 6H), 7.25 – 7.18 (m, 2H), 5.99 (d, *J* = 16.2 Hz, 1H), 5.12

(s, 2H), 2.11 (s, 3H), 1.52 (s, 9H). **^{13}C NMR** (101 MHz, $CDCl_3$) δ 170.73, 165.27, 139.49, 137.15, 136.37, 134.85, 134.74, 132.18, 131.38, 129.46, 128.88, 128.59, 127.82, 127.74, 80.91, 64.39, 28.29, 21.13. **IR** (KBr): υ 3059, 2978, 1743, 1710, 1640, 1367, 1316, 1233, 1151, 1025 cm^{-1}. **HRMS** (ESI): Calculated for $C_{22}H_{24}O_4S$ (M^+): 384.1390, found: 384.1390.

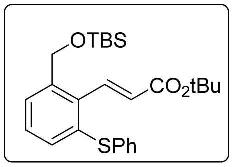

4f: Colorless oil (56%). R_f = 0.2 (hexane/ethyl acetate = 10:1). **^{1}H NMR** (500 MHz, Chloroform-*d*) δ 7.81 (d, *J* = 16.2 Hz, 1H), 7.42 (dd, *J* = 7.3, 1.5 Hz, 1H), 7.28 (dd, *J* = 4.0, 0.9 Hz, 4H), 7.24 – 7.17 (m, 3H), 6.10 (d, *J* = 16.2 Hz, 1H), 4.67 (s, 2H), 1.51 (s, 9H), 0.94 (s, 9H), 0.11 (s, 6H). **^{13}C NMR** (126 MHz, $CDCl_3$) δ 165.7, 140.4, 139.8, 136.1, 135.6, 135.4, 131.6, 131.0, 129.3, 127.4, 127.3, 127.3, 80.6, 63.5, 28.3, 26.1, 18.5, -5.1. **IR** (KBr): υ 3059, 2955, 2929, 2884, 2857, 1712, 1639, 1583, 1473, 1440, 1151 cm^{-1}. **HRMS** (ESI): Calculated for $C_{26}H_{36}O_3SSiNa$ (M+Na^+): 479.2047, found: 479.2044.

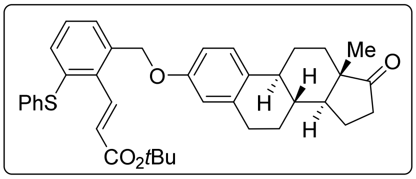

4g: Colorless oil (47%). R_f = 0.2 (hexane/ethyl acetate = 20:1). **^{1}H NMR** (400 MHz, Chloroform-*d*) δ 7.85 (d, *J* = 16.2 Hz, 1H), 7.49 – 7.44 (m, 1H), 7.34 – 7.29 (m, 4H), 7.29 – 7.26 (m, 1H), 7.25 – 7.22 (m, 2H), 7.22 – 7.18 (m, 1H), 6.75 (dd, *J* = 8.6, 2.8 Hz, 1H), 6.69 (d, *J* = 2.8 Hz, 1H), 6.02 (d, *J* = 16.3 Hz, 1H), 5.02 (s, 2H), 2.95 – 2.86 (m, 2H), 2.51 (dd, *J* = 18.7, 8.6 Hz, 1H), 2.43 – 2.36 (m, 1H), 2.27 (d, *J* = 10.0 Hz, 1H), 2.20 – 1.92 (m, 4H), 1.68 – 1.49 (m, 6H), 1.47 (s, 9H), 0.91 (s, 3H). **^{13}C NMR** (126 MHz, $CDCl_3$) δ 165.4, 156.6, 139.6, 138.0, 136.7, 136.4, 135.9, 135.1, 132.8, 131.9, 131.4, 129.4, 129.0, 128.1, 127.7, 127.6, 126.5, 115.3, 112.8, 80.8, 68.3, 50.6, 48.2, 44.2, 38.5, 36.0, 31.7, 28.3, 26.7, 26.1, 21.7, 14.0. **IR** (KBr): υ 2929, 1738, 1709, 1608, 1498, 1477, 1440, 1368, 1315, 1152 cm^{-1}. **HRMS** (ESI): Calculated for $C_{38}H_{42}O_4S$ Na(M+Na^+):617.2696, found: 617.2705.

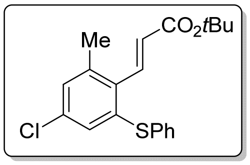

4h: Colorless oil (59%). R_f = 0.3 (hexane/ethyl acetate = 20:1). **^{1}H NMR** (400 MHz, Chloroform-*d*) δ 7.74 (d, *J* = 16.3 Hz, 1H), 7.37 – 7.29 (m, 5H), 7.07 (dt, *J* = 1.3, 0.6 Hz, 1H), 6.95 (d, *J* = 2.1 Hz, 1H), 6.03 (d, *J* = 16.3 Hz, 1H), 2.37 – 2.32 (m, 3H), 1.52 (s, 9H). **^{13}C NMR** (101 MHz, $CDCl_3$) δ 165.5, 139.9, 139.6, 139.0, 134.0, 133.7, 133.3, 132.7, 129.5, 128.9, 128.1, 127.7, 127.2, 80.8, 28.2, 21.2. **IR** (KBr): υ 2977, 1711, 1639, 1572, 1549, 1478, 1392, 1367, 1314, 1150 cm^{-1}. **HRMS** (ESI): Calculated for $C_{20}H_{20}ClOS$ Na[(M+Na^+)+(-H_2O)]:365.0737, found: 365.0734.

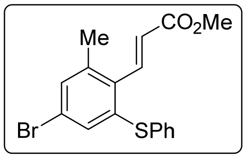

4i: Colorless oil (55%). R_f = 0.3 (hexane/ethyl acetate = 20:1). **^{1}H NMR** (400 MHz, Chloroform-*d*) δ 7.82 (d, *J* = 16.3 Hz, 1H), 7.39 – 7.29 (m, 5H), 7.25 – 7.22 (m, 1H), 7.10 (d, *J* = 2.0 Hz, 1H), 6.13 (d, *J* = 16.4 Hz, 1H), 3.80 (s, 3H), 2.34 (d, *J* = 0.6 Hz, 3H). **^{13}C NMR** (101 MHz, $CDCl_3$) δ 166.8, 141.2, 139.3, 139.3, 133.8, 133.7, 132.7, 132.0, 130.7, 129.7, 128.3, 125.3, 122.7, 52.0, 21.1. **IR** (KBr): υ 3059, 2949, 2925, 2360, 1722, 1639, 1565, 1438, 1306, 1272, 1195, 1172, 748, 690 cm^{-1}. **HRMS** (ESI): Calculated for $C_{17}H_{16}BrO_2S$ (M+H^+): 363.0049, found: 363.0058.

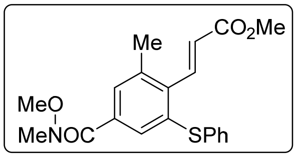

4j: Colorless oil (57%). R_f = 0.2 (hexane/ethyl acetate = 2:1). **^{1}H NMR** (400 MHz, Chloroform-*d*) δ 7.91 (d, *J* = 16.4 Hz, 1H), 7.41 (s, 1H), 7.37 – 7.26 (m, 6H), 6.15 (d, *J* = 16.3 Hz, 1H), 3.81 (s, 3H), 3.44 (s, 3H), 3.27 (s, 3H), 2.40 (s, 3H). **^{13}C NMR** (101 MHz, $CDCl_3$) δ 168.9, 166.7, 141.6, 137.5, 137.4, 136.7, 134.7, 134.4, 132.2, 129.5, 129.1, 128.5, 127.9, 125.5, 61.2, 52.0, 33.7, 21.3. **IR** (KBr): υ 2950, 1722, 1644, 1439, 1309, 1274, 1197, 1172, 748, 692 cm^{-1}. **HRMS** (ESI): Calculated for $C_{20}H_{21}NO_4SNa$ (M+Na^+): 394.1083, found: 394.1087.

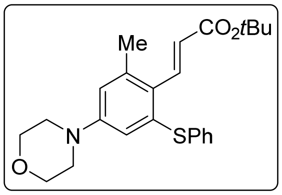

4k: White solid (64%). R$_f$ = 0.2 (hexane/ethyl acetate = 10:1). Mp = 93.4 – 94.3 °C. **^{1}H NMR** (400 MHz, Chloroform-*d*) δ 7.81 (d, *J* = 16.3 Hz, 1H), 7.19 (dd, *J* = 4.1, 0.8 Hz, 4H), 7.16 – 7.10 (m, 1H), 6.64 (d, *J* = 2.6 Hz, 1H), 6.59 (d, *J* = 2.6 Hz, 1H), 5.93 (d, *J* = 16.3 Hz, 1H), 3.75 – 3.67 (m, 4H), 3.06 – 2.97 (m, 4H), 2.32 (s, 3H), 1.42 (s, 9H). **^{13}C NMR** (101 MHz, $CDCl_3$) δ 166.5, 150.8, 140.8, 139.3, 137.5, 136.0, 131.0, 129.2, 127.0, 124.3, 117.0, 116.9, 80.3, 66.7, 48.3, 28.3, 22.5. **IR** (KBr): υ 2974, 2855, 1704, 1627, 1588, 1538, 1478, 1449, 1367, 1310 cm^{-1}. **HRMS** (ESI): Calculated for $C_{23}H_{29}NO_3S$ Na(M+Na$^+$): 434.1760, found: 434.1760.

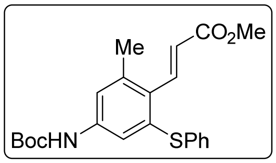

4l: Colorless oil (55%). R$_f$ = 0.3 (hexane/ethyl acetate = 5:1). **^{1}H NMR** (500 MHz, Chloroform-*d*) δ 7.93 (d, *J* = 16.3 Hz, 1H), 7.46 (s, 1H), 7.29 (d, *J* = 3.9 Hz, 4H), 7.24 (td, *J* = 3.3, 2.1 Hz, 1H), 6.84 (d, *J* = 2.3 Hz, 1H), 6.37 (s, 1H), 6.11 (d, *J* = 16.3 Hz, 1H), 3.78 (s, 3H), 2.38 (s, 3H), 1.47 (s, 9H). **^{13}C NMR** (101 MHz, $CDCl_3$) δ 167.3, 152.4, 141.9, 139.2, 138.9, 137.5, 135.2, 131.8, 129.4, 127.5, 123.6, 119.6, 119.2, 81.1, 51.8, 28.4, 21.9. **IR** (KBr): υ 3332, 2978, 1703, 1577, 1516, 1272, 1228, 1158, 1069, 739, 690 cm^{-1}. **HRMS** (ESI): Calculated for $C_{22}H_{25}NO_4S$ Na(M+Na$^+$): 422.1397, found: 422.1400.

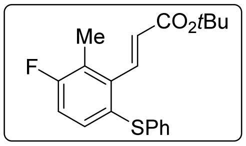

4m: Colorless oil (71%). R$_f$ = 0.3 (hexane/ethyl acetate = 20:1). **^{1}H NMR** (400 MHz, Chloroform-*d*) δ 7.75 (d, *J* = 16.4 Hz, 1H), 7.28 – 7.15 (m, 7H), 6.95 (t, *J* = 8.9 Hz, 1H), 5.91 (d, *J* = 16.3 Hz, 1H), 2.28 (d, *J* = 2.6 Hz, 3H), 1.50 (s, 9H). **^{13}C NMR** (101 MHz, Chloroform-*d*) δ 165.5, 161.2 (d, *J* = 245.9 Hz), 140.2 (d, *J* = 2.7 Hz), 139.3 (d, *J* = 4.8 Hz), 136.3, 132.3 (d, *J* = 8.8 Hz), 130.4, 130.1 (d, *J* = 3.5 Hz), 129.2, 127.7, 126.9, 124.8 (d, *J* = 17.4 Hz), 115.7

(d, J = 23.9 Hz), 80.8, 28.3, 12.8 (d, J = 5.4 Hz). **IR** (KBr): υ 2978, 2932, 1712, 1641, 1582, 1479, 1456, 1392, 1367, 1152 cm^{-1}. **HRMS** (ESI): Calculated for $C_{21}H_{22}FO_2S$ ($M+H^+$): 345.1319, found: 345.1325.

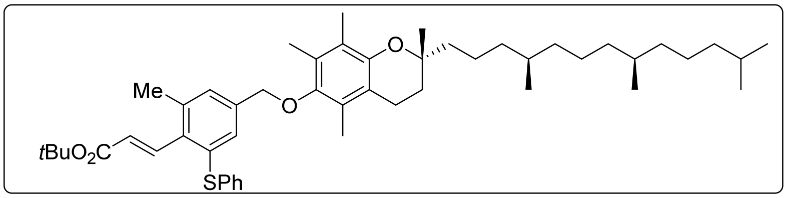

4n: Colorless oil (72%). R_f = 0.2 (hexane/ethyl acetate = 20:1). **^{1}H NMR** (400 MHz, Chloroform-d) δ 7.84 (d, J = 16.3 Hz, 1H), 7.34 – 7.29 (m, 3H), 7.28 – 7.19 (m, 4H), 6.03 (d, J = 16.3 Hz, 1H), 4.55 (s, 2H), 2.57 (t, J = 6.9 Hz, 2H), 2.43 (s, 3H), 2.15 – 2.05 (m, 9H), 1.87 – 1.72 (m, 2H), 1.52 (s, 10H), 1.46 – 1.33 (m, 5H), 1.33 – 1.23 (m, 11H), 1.17 – 1.03 (m, 7H), 0.86 (dd, J = 9.3, 6.5 Hz, 12H). **^{13}C NMR** (101 MHz, $CDCl_3$) δ 165.9, 148.1, 148.0, 140.8, 138.5, 137.9, 136.6, 135.4, 135.2, 131.7, 129.3, 129.0, 128.8, 127.9, 127.4, 126.8, 126.0, 123.1, 117.7, 80.7, 75.0, 74.0, 40.2, 39.5, 37.5, 32.9, 32.8, 31.4, 28.3, 28.1, 25.0, 25.0, 24.6, 24.0, 22.9, 22.8, 21.6, 21.2, 20.8, 19.9, 19.84, 19.78, 13.0, 12.1, 12.0. **IR** (KBr): υ 2927, 2867, 1712, 1638, 1553, 1460, 1366, 1313, 1256, 1150 cm^{-1}. **HRMS** (ESI): Calculated for $C_{50}H_{72}O_4SNa$ ($M+Na^+$):769.5224, found:769.52.

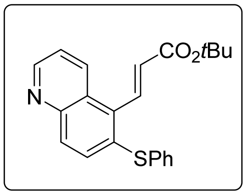

4o: Pale yellow oil (90%). R_f = 0.1 (hexane/ethyl acetate = 5:1). **^{1}H NMR** (400 MHz, $CDCl_3$) δ 8.80 (dd, J = 4.1, 1.5 Hz, 1H), 8.39 (ddd, J = 8.6, 1.5, 0.8 Hz, 1H), 8.04 (d, J = 16.2 Hz, 1H), 7.85 (d, J = 9.0 Hz, 1H), 7.41 – 7.21 (m, 7H), 6.11 (d, J = 16.2 Hz, 1H), 1.49 (s, 9H). **^{13}C NMR** (126 MHz, $CDCl_3$) δ 165.4, 150.3, 147.5, 138.7, 135.5, 134.3, 133.3, 132.8, 132.6, 131.4, 130.5, 129.6, 129.0, 128.1, 126.8, 122.0, 81.2, 28.4. **IR** (KBr): υ 2977, 1710, 1633, 1579, 1488, 1367, 1312, 1286, 1151, 1024 cm^{-1}. **HRMS** (ESI): Calculated for $C_{22}H_{21}NO_2SNa$ ($M+Na^+$): 386.1185, found: 386.1192.

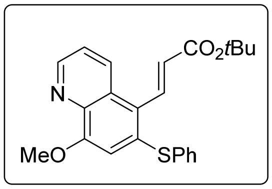

4p: Yellow solid (59%). R_f = 0.1 (hexane/ethyl acetate = 5:1). Mp = 139.7 – 140.2 °C. **^{1}H NMR** (400 MHz, $CDCl_3$) δ 8.91 (dd, J = 4.2, 1.6 Hz, 1H), 8.49 (dd, J = 8.7, 1.7 Hz, 1H), 8.10 (d, J = 16.2 Hz, 1H), 7.48 (dd, J = 8.6, 4.2 Hz, 1H), 7.43 – 7.38 (m, 2H), 7.37 – 7.28 (m, 3H), 6.84 (s, 1H), 6.15 (d, J = 16.2 Hz, 1H), 3.86 (s, 3H), 1.56 (s, 9H). **^{13}C NMR** (101 MHz, $CDCl_3$) δ 165.6, 155.3, 149.0, 138.5, 136.1, 134.4, 133.5, 132.3, 129.4, 128.0, 127.7, 127.5, 125.0, 122.5, 110.0, 80.9, 56.0, 28.2. **IR** (KBr): υ 2976, 1707, 1572, 1497, 1456, 1366, 1290, 1246, 1148, 1124 cm^{-1}. **HRMS** (ESI): Calculated for $C_{23}H_{23}NO_3SNa$ ($M+Na^+$): 416.1291, found: 416.1292.

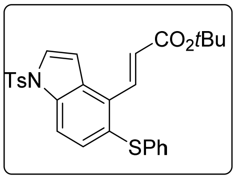

4q: Colorless oil (40%). R_f = 0.2 (hexane/ethyl acetate = 10:1). **^{1}H NMR** (400 MHz, $CDCl_3$) δ 8.13 (d, J = 16.2 Hz, 1H), 7.91 (dd, J = 8.7, 0.8 Hz, 1H), 7.77 (d, J = 8.4 Hz, 2H), 7.65 (d, J = 3.7 Hz, 1H), 7.38 (d, J = 8.7 Hz, 1H), 7.27 (d, J = 0.9 Hz, 1H), 7.25 (dd, J = 1.7, 0.9 Hz, 1H), 7.22 (dd, J = 2.3, 1.2 Hz, 1H), 7.21 (d, J = 0.9 Hz, 1H), 7.20 – 7.14 (m, 3H), 6.91 (dd, J = 3.7, 0.8 Hz, 1H), 6.27 (d, J = 16.2 Hz, 1H), 2.37 (s, 3H), 1.50 (s, 9H). **^{13}C NMR** (101 MHz, $CDCl_3$) δ 165.9, 145.5, 140.2, 136.9, 135.1, 134.8, 131.0, 130.5, 130.2, 130.1, 130.0, 129.9, 129.2, 127.8, 127.0, 126.7, 125.7, 114.8, 108.2, 80.8, 28.3, 21.8. **IR** (KBr): υ 2977, 2359, 2341, 1706, 1633, 1596, 1582, 1478, 1375, 1170 cm^{-1}. **HRMS** (ESI): Calculated for $C_{28}H_{27}NO_4S_2$ (M^+):505.1376, found:505.1381.

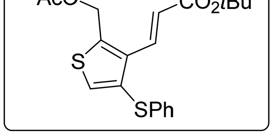

4r: Pale yellow solid (36%). R_f = 0.2 (hexane/ethyl acetate = 10:1). Mp = 84.5 – 85.2 °C. **^{1}H NMR** (400 MHz, $CDCl_3$) δ 7.60 (d, J = 16.2 Hz, 1H), 7.37 (s, 1H), 7.25 (m, 2H), 7.22 – 7.15 (m, 3H), 6.22 (d, J = 16.2 Hz, 1H), 5.29 (s, 2H), 2.13 (s, 3H), 1.48 (s, 9H). **^{13}C NMR** (101 MHz, $CDCl_3$) δ 170.6, 165.9, 138.7, 136.3, 136.0, 134.8, 130.8,

129.7, 129.3, 129.1, 126.8, 124.6, 80.8, 59.5, 28.3, 21.0. **IR** (KBr): υ 2977, 1745, 1707, 1632, 1582, 1478, 1367, 1312, 1284, 1150 cm^{-1}. **HRMS** (ESI): Calculated for $C_{20}H_{22}O_4S_2$ Na(M+Na$^+$): 413.0852, found: 413.0861.

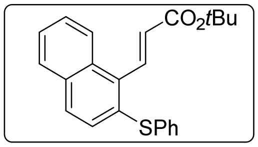

4s: Colorless oil (93%). R_f = 0.3 (hexane/ethyl acetate = 20:1). **^{1}H NMR** (400 MHz, $CDCl_3$) δ 8.13 – 8.02 (m, 2H), 7.79 – 7.66 (m, 1H), 7.60 (d, *J* = 8.7 Hz, 1H), 7.43 (dddd, *J* = 17.5, 8.1, 6.9, 1.4 Hz, 2H), 7.27 – 7.15 (m, 6H), 6.14 (d, *J* = 16.3 Hz, 1H), 1.49 (s, 9H). **^{13}C NMR** (101 MHz, $CDCl_3$) δ 165.8, 140.0, 135.6, 133.8, 133.7, 132.6, 131.8, 131.7, 129.4, 129.2, 128.7, 128.5, 128.4, 127.5, 127.3, 126.3, 125.2, 80.9, 28.4. **IR** (KBr): υ 3056, 2977, 2930, 1709, 1635, 1582, 1477, 1367, 1312, 1284 cm^{-1}. **HRMS** (ESI): Calculated for $C_{23}H_{22}O_2SNa$(M+Na$^+$): 385.1233, found: 385.1236.

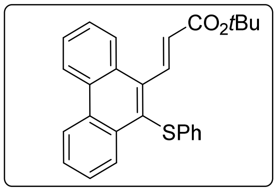

4t: White solid (45%). R_f = 0.4 (hexane/ethyl acetate = 20:1). Mp = 174.8 – 175.4 °C. **^{1}H NMR** (500 MHz, $CDCl_3$) δ 8.77 (d, *J* = 8.3 Hz, 1H), 8.73 (d, *J* = 8.2 Hz, 1H), 8.67 (dd, *J* = 8.3, 0.9 Hz, 1H), 8.29 – 8.20 (m, 2H), 7.74 (ddd, *J* = 8.3, 7.0, 1.2 Hz, 1H), 7.69 (ddd, *J* = 8.3, 7.0, 1.2 Hz, 1H), 7.64 (ddd, *J* = 8.2, 7.1, 1.1 Hz, 1H), 7.60 (ddd, *J* = 8.1, 7.0, 1.1 Hz, 1H), 7.12 (t, *J* = 7.5 Hz, 2H), 7.09 – 6.99 (m, 3H), 6.02 (d, *J* = 16.4 Hz, 1H), 1.55 (s, 9H). **^{13}C NMR** (101 MHz, $CDCl_3$) δ 165.5, 141.7, 140.0, 137.7, 132.0, 131.3, 130.8, 130.1, 128.9, 127.9, 127.9, 127.8, 127.6, 127.6, 127.5, 127.2, 127.1, 127.1, 125.3, 123.0, 122.8, 80.7, 28.2. **IR** (KBr): υ 3854, 3712, 3629, 2360, 2343, 1735, 1712, 1654, 1560, 1154 cm^{-1}. **HRMS** (ESI): Calculated for $C_{27}H_{25}O_2S$ (M+H$^+$): 413.1570, found: 413.1578.

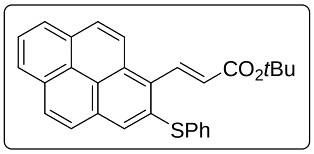

4u: Yellow solid (91%). R_f = 0.4 (hexane/ethyl acetate = 10:1). Mp = 116.4 – 117.9 °C. **^{1}H NMR** (400 MHz, Chloroform-*d*) δ 8.43 – 8.33 (m, 2H), 8.18 (t, *J* = 7.3 Hz, 2H), 8.14 – 7.98 (m, 4H), 7.87 (dd, *J* = 8.9, 1.1 Hz, 1H), 7.39 – 7.26 (m, 5H), 6.28 (d, *J* = 16.2 Hz, 1H), 1.60 (s, 9H). **^{13}C NMR** (101 MHz, $CDCl_3$) δ 165.7, 140.7, 135.9, 133.5, 131.8, 131.7, 131.5, 131.3, 130.8, 129.8, 129.5, 128.8, 128.7, 128.7, 128.2, 127.4, 126.9, 126.5, 125.9, 125.7, 124.6, 124.6, 124.2, 80.9, 28.4. **IR** (KBr): υ 3048, 2977, 2930, 1708, 1635, 1581, 1530, 1478, 1367, 1150 cm^{-1}. . **HRMS** (ESI): Calculated for $C_{29}H_{24}O_2SNa$ (M+Na$^+$): 459.1389, found: 459.1387.

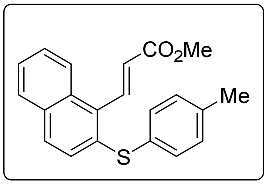

5a: Colorless oil (86%). R_f = 0.3 (hexane/ethyl acetate = 20:1). **^{1}H NMR** (400 MHz, Chloroform-*d*) δ 8.30 (d, *J* = 16.2 Hz, 1H), 8.15 – 8.09 (m, 1H), 7.81 – 7.76 (m, 1H), 7.69 – 7.62 (m, 1H), 7.55 – 7.43 (m, 2H), 7.30 (d, *J* = 8.2 Hz, 2H), 7.22 (d, *J* = 8.7 Hz, 1H), 7.18 – 7.13 (m, 2H), 6.36 (d, *J* = 16.3 Hz, 1H), 3.88 (s, 3H), 2.36 (s, 3H). **^{13}C NMR** (101 MHz, $CDCl_3$) δ 166.9, 141.3, 138.1, 135.2, 132.8, 132.3, 132.2, 131.6, 131.1, 129.4, 128.6, 127.6, 127.4, 126.1, 126.1, 124.9, 52.0, 21.3. **IR** (KBr): υ 2948, 1721, 1636, 1584, 1492, 1434, 1308, 1280, 1171, 1127 cm^{-1}. **HRMS** (ESI): Calculated for $C_{21}H_{19}O_2S$ (M+H$^+$): 335.1100, found: 335.1096.

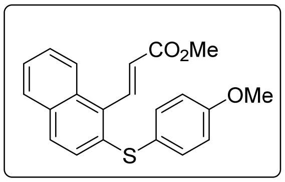

5b: Yellow oil (71%). R_f = 0.2 (hexane/ethyl acetate = 20:1). **^{1}H NMR** (400 MHz, Chloroform-*d*) δ 8.29 (d, *J* = 16.3 Hz, 1H), 8.10 (dd, *J* = 8.6, 1.2 Hz, 1H), 7.77 (dd, *J* = 8.0, 1.5 Hz, 1H), 7.63 (d, *J* = 8.8 Hz, 1H), 7.55 – 7.37 (m, 4H), 7.11 (d, *J* = 8.8 Hz, 1H), 6.90 (d, *J* = 8.8 Hz, 2H), 6.38 (d, *J* = 16.3 Hz, 1H), 3.88 (s, 3H), 3.83 (s, 3H). **^{13}C NMR** (101 MHz, $CDCl_3$) δ 167.0, 160.1, 141.2, 136.5, 135.7, 132.0, 131.5, 130.8, 129.3, 128.6, 127.4, 126.5, 126.1, 125.9, 124.7, 124.4, 115.2, 55.5, 52.0. **IR** (KBr): υ 2948, 1721, 1636, 1584, 1492, 1434, 1308, 1280, 1171, 1127 cm^{-1}. **HRMS** (ESI): Calculated for $C_{21}H_{19}O_3S$ (M+H$^+$): 351.1049, found: 351.1039.

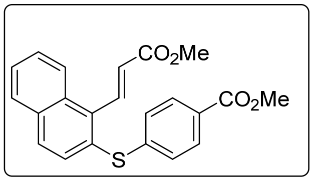

5c: Yellow solid (82%). R_f = 0.3 (hexane/ethyl acetate = 5:1). Mp = 94.5 – 95.1 °C. **^{1}H NMR** (400 MHz, $CDCl_3$) δ 8.20 – 8.02 (m, 2H), 7.87 – 7.67 (m, 4H), 7.49 (ddd, *J* = 5.6, 4.2, 2.1 Hz, 2H), 7.38 (d, *J* = 8.6 Hz, 1H), 7.17 – 7.07 (m, 2H), 6.20 (d, *J* = 16.3 Hz, 1H), 3.81 (s, 3H), 3.76 (s, 3H). **^{13}C NMR** (101 MHz, $CDCl_3$) δ 166.7, 166.7, 143.3, 141.3, 136.3, 133.4, 131.8, 130.5, 130.4, 130.3, 129.9, 128.7, 128.5, 128.0, 127.6, 127.1, 126.5, 125.5, 52.3, 52.0. **IR** (KBr): υ 2950, 2360, 1720, 1636, 1594, 1559, 1506, 1435, 1399, 1308 cm^{-1}. **HRMS** (ESI): Calculated for $C_{22}H_{19}O_4S$ ($M+H^+$): 379.0999, found: 379.1002.

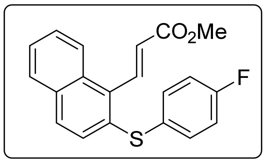

5d: Colorless oil (82%). R_f = 0.3 (hexane/ethyl acetate = 20:1). **^{1}H NMR** (400 MHz, Chloroform-*d*) δ 8.28 (d, *J* = 16.3 Hz, 1H), 8.15 – 8.09 (m, 1H), 7.80 (dd, *J* = 8.2, 1.5 Hz, 1H), 7.68 (d, *J* = 8.7 Hz, 1H), 7.57 – 7.45 (m, 2H), 7.36 (dd, *J* = 8.8, 5.2 Hz, 2H), 7.21 (d, *J* = 8.7 Hz, 1H), 7.03 (t, *J* = 8.7 Hz, 2H), 6.34 (d, *J* = 16.3 Hz, 1H), 3.87 (s, 3H). **^{13}C NMR** (101 MHz, Chloroform-*d*) δ 166.83, 162.63 (d, *J* = 248.5 Hz), 141.18, 134.52 (d, *J* = 8.2 Hz), 134.37, 132.70, 132.43, 131.56, 130.09 (d, *J* = 3.4 Hz), 129.54, 128.59, 127.72, 127.48, 126.35, 126.32, 124.94, 116.67 (d, *J* = 22.0 Hz), 52.01. **^{19}F NMR** (470 MHz, $CDCl_3$) δ -113.3. **IR** (KBr): υ 2949, 1719, 1636, 1588, 1505, 1489, 1435, 1280, 1172, 1127 cm^{-1}. **HRMS** (ESI): Calculated for $C_{20}H_{16}FO_2S$ ($M+H^+$): 339.0850, found: 339.0856.

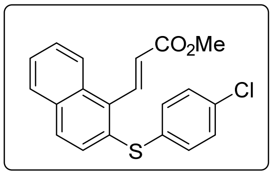

5e: Colorless oil (82%). R_f = 0.3 (hexane/ethyl acetate = 20:1). **^{1}H NMR** (400 MHz, $CDCl_3$) δ 8.18 (d, *J* = 16.3 Hz, 1H), 8.08 – 8.02 (m, 1H), 7.74 (dd, *J* = 7.1, 2.3 Hz, 1H), 7.64 (d, *J* = 8.7 Hz, 1H), 7.45 (dqd, *J* = 8.4, 6.9, 1.6 Hz, 2H), 7.25 – 7.12 (m, 5H), 6.23 (d, *J* = 16.3 Hz, 1H), 3.78 (s, 3H). **^{13}C NMR** (101 MHz, $CDCl_3$) δ 166.8, 141.2,

134.2, 134.0, 133.6, 133.0, 132.8, 132.6, 131.6, 129.7, 129.6, 128.7, 128.6, 127.5, 126.6, 126.4, 125.1, 52.0. **IR** (KBr): υ 3055, 2948, 1721, 1637, 1475, 1434, 1309, 1280, 1193, 1172 cm^{-1}. **HRMS** (ESI): Calculated for $C_{20}H_{16}ClO_2S$ ($M+H^+$): 335.0554, found: 335.0552.

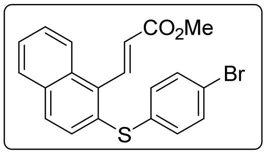

5f: Colorless oil (86%). R_f = 0.3 (hexane/ethyl acetate = 20:1). **^{1}H NMR** (400 MHz, $CDCl_3$) δ 8.19 (d, *J* = 16.3 Hz, 1H), 8.06 (dd, *J* = 8.5, 1.0 Hz, 1H), 7.76 (dd, *J* = 7.0, 2.4 Hz, 1H), 7.66 (d, *J* = 8.7 Hz, 1H), 7.51 – 7.41 (m, 2H), 7.38 – 7.31 (m, 2H), 7.25 (d, *J* = 8.7 Hz, 1H), 7.16 – 7.05 (m, 2H), 6.25 (d, *J* = 16.3 Hz, 1H), 3.80 (s, 3H). **^{13}C NMR** (101 MHz, $CDCl_3$) δ 166.8, 141.2, 135.0, 134.2, 132.8, 132.7, 132.7, 132.5, 131.6, 129.7, 128.8, 128.6, 127.5, 126.7, 126.4, 125.2, 121.4, 52.0. **IR** (KBr): υ 3853, 3745, 3649, 3055, 2948, 2360, 1719, 1636, 1471, 1172 cm^{-1}. **HRMS** (ESI): Calculated for $C_{25}H_{16}BrO_2S$ ($M+H^+$): 399.0049, found: 399.0044.

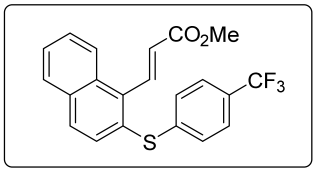

5g: White solid oil (85%). R_f = 0.2 (hexane/ethyl acetate = 20:1). Mp = 76.0 – 77.0 °C. **^{1}H NMR** (400 MHz, Chloroform-*d*) δ 8.24 (d, *J* = 16.3 Hz, 1H), 8.18 – 8.11 (m, 1H), 7.90 – 7.83 (m, 1H), 7.81 – 7.77 (m, 1H), 7.60 – 7.54 (m, 2H), 7.53 – 7.42 (m, 3H), 7.28 (d, *J* = 0.8 Hz, 2H), 6.28 (d, *J* = 16.3 Hz, 1H), 3.84 (s, 3H). **^{13}C NMR** (101 MHz, Chloroform-*d*) δ 166.7, 141.9, 141.3, 136.2, 133.4, 131.8, 130.4, 130.3, 130.0, 129.2, 128.7, 127.6, 127.5 – 119.4 (m), 127.1, 126.6, 126.0 (q, *J* = 3.8 Hz), 125.5, 52.0. **^{19}F NMR** (470 MHz, $CDCl_3$) δ -62.5. **IR** (KBr): υ 3853, 3057, 2951, 1723, 1639, 1605, 1436, 1327, 1280, 1170 cm^{-1}. **HRMS** (ESI): Calculated for $C_{21}H_{16}F_3O_2S$ ($M+H^+$): 389.0818, found: 389.0818.

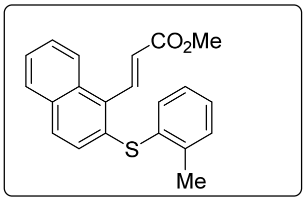

5h: Colorless oil (91%). R_f = 0.2 (hexane/ethyl acetate = 20:1). **^{1}H NMR** (400 MHz, $CDCl_3$) δ 8.20 (d, *J* = 16.3 Hz, 1H), 8.09 – 7.99 (m, 1H), 7.77 – 7.64 (m, 1H), 7.55 (d, *J* = 8.8 Hz, 1H), 7.40 (dddd, *J* = 20.5, 8.0, 6.9, 1.3 Hz, 2H), 7.23 (d, *J* = 7.5 Hz, 1H), 7.20 – 7.12 (m, 2H), 7.06 (ddd, *J* = 8.4, 5.5, 2.4 Hz, 1H), 6.98 (d, *J* = 8.7 Hz, 1H), 6.29 (d, *J* = 16.3 Hz, 1H), 3.78 (s, 3H), 2.26 (s, 3H). **^{13}C NMR** (101 MHz, $CDCl_3$) δ 167.0, 141.2, 140.6, 134.6, 134.0, 133.5, 132.3, 132.1, 131.6, 130.9, 129.4, 128.6, 128.5, 127.4, 127.0, 127.0, 126.1, 126.1, 124.8, 52.0, 20.9. **IR** (KBr): υ 3058, 2948, 1721, 1637, 1584, 1434, 1280, 1172, 1059, 1036 cm^{-1}. **HRMS** (ESI): Calculated for $C_{21}H_{19}O_2S$ ($M+H^+$): 335.1100, found: 335.1098.

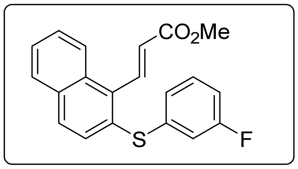

5i: White solid (92%). R_f = 0.2 (hexane/ethyl acetate = 20:1). Mp = 80.4 – 81.2 °C. **^{1}H NMR** (400 MHz, $CDCl_3$) δ 8.18 (d, *J* = 16.3 Hz, 1H), 8.11 – 8.00 (m, 1H), 7.82 – 7.73 (m, 1H), 7.68 (d, *J* = 8.7 Hz, 1H), 7.55 – 7.41 (m, 2H), 7.32 (d, *J* = 8.7 Hz, 1H), 7.19 – 7.13 (m, 1H), 6.97 (ddd, *J* = 7.8, 1.6, 1.0 Hz, 1H), 6.91 – 6.81 (m, 2H), 6.22 (d, *J* = 16.3 Hz, 1H), 3.78 (s, 3H). **^{13}C NMR** (101 MHz, Chloroform-*d*) δ 166.75, 163.10 (d, *J* = 248.9 Hz), 141.32, 138.49 (d, *J* = 7.8 Hz), 135.10, 133.09, 131.74 (d, *J* = 9.5 Hz), 130.56 (d, *J* = 8.5 Hz), 129.71 (d, *J* = 14.5 Hz), 128.64, 127.52, 126.85, 126.42, 126.03 (d, *J* = 3.0 Hz), 125.32, 117.23 (d, *J* = 23.2 Hz), 114.10 (d, *J* = 21.2 Hz), 52.03. **^{19}F NMR** (470 MHz, Chloroform-*d*) δ -111.75 (q, *J* = 8.7 Hz). **IR** (KBr): υ 3060, 2949, 1721, 1639, 1597, 1580, 1473, 1433, 1309, 1281 cm^{-1}. **HRMS** (ESI): Calculated for $C_{20}H_{16}FO_2S$ ($M+H^+$): 339.0850, found: 339.0847.

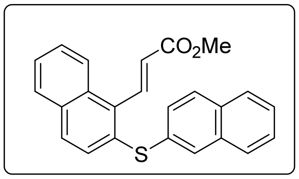

5j: White solid (86%). R_f = 0.2 (hexane/ethyl acetate = 20:1). Mp = 122.0 – 123.0 °C. **^{1}H NMR** (400 MHz, $CDCl_3$) δ 8.27 (d, *J* = 16.3 Hz, 1H), 8.09 (d, *J* = 8.3 Hz, 1H), 7.82 (s, 1H), 7.77 – 7.66 (m, 4H), 7.61 (d, *J* = 8.7 Hz, 1H), 7.51 – 7.38 (m, 4H), 7.31 – 7.19 (m, 2H), 6.30 (d, *J* = 16.3 Hz, 1H), 3.78 (s, 3H). **^{13}C NMR** (101 MHz, $CDCl_3$) δ 166.9, 141.3, 134.0, 133.9, 133.4, 132.6, 132.60, 132.5, 131.6, 130.9, 129.5, 129.2, 129.2, 128.6, 128.6, 127.9, 127.6, 127.4, 126.9, 126.6, 126.4, 126.3, 125.1, 52.0. **IR** (KBr): υ 3053, 2947, 1720, 1636, 1584, 1557, 1500, 1434, 1309, 1281 cm^{-1}. **HRMS** (ESI): Calculated for $C_{24}H_{19}O_2S$ (M+H$^+$): 371.1100, found: 371.1096.

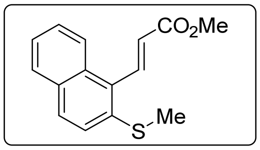

5k: Colorless oil (40%). R_f = 0.2 (hexane/ethyl acetate = 20:1). **^{1}H NMR** (400 MHz, $CDCl_3$) δ 8.21 (d, *J* = 16.3 Hz, 1H), 8.07 (dd, *J* = 8.6, 1.1 Hz, 1H), 7.90 (dd, *J* = 10.6, 8.0 Hz, 1H), 7.85 – 7.77 (m, 2H), 7.54 – 7.41 (m, 3H), 6.36 (d, *J* = 16.3 Hz, 1H), 5.97 (d, *J* = 16.3 Hz, 1H), 3.87 (s, 3H), 2.56 (s, 3H). **^{13}C NMR** (101 MHz, $CDCl_3$) δ 167.0, 141.2, 136.1, 131.6, 131.4, 130.8, 129.4, 128.6, 127.4, 126.0, 125.7, 124.5, 124.2, 52.0, 16.9. **IR** (KBr): υ 3685, 2947, 1720, 1633, 1583, 1504, 1434, 1281, 1191, 1172 cm^{-1}. **HRMS** (ESI): Calculated for $C_{15}H_{15}O_2S$ (M+H$^+$):259.0787, found:259.0793.

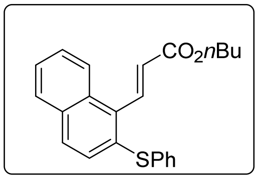

5l: Colorless oil (84%). R_f = 0.3 (hexane/ethyl acetate = 20:1). **^{1}H NMR** (400 MHz, Chloroform-*d*) δ 8.28 (d, *J* = 16.2 Hz, 1H), 8.14 (d, *J* = 8.2 Hz, 1H), 7.81 (dd, *J* = 8.1, 1.4 Hz, 1H), 7.69 (d, *J* = 9.1 Hz, 1H), 7.57 – 7.46 (m, 2H), 7.37 – 7.24 (m, 6H), 6.32 (d, *J* = 16.3 Hz, 1H), 4.27 (t, *J* = 6.7 Hz, 2H), 1.73 (ddt, *J* = 8.8, 7.9, 6.6 Hz, 2H), 1.53 – 1.39 (m, 2H), 0.99 (t, *J* = 7.4 Hz, 3H). **^{13}C NMR** (101 MHz, $CDCl_3$) δ 166.5, 141.0, 135.4, 133.8, 133.6, 132.6, 131.7, 131.6, 129.4, 128.6, 128.6, 127.5, 127.4, 126.7, 126.4, 125.1, 64.8, 30.9, 19.4, 13.9. **IR** (KBr): υ 2958, 2872, 1714, 1639, 1582, 1477, 1306, 1280, 1257, 1174 cm^{-1}. **HRMS** (ESI): Calculated for $C_{23}H_{23}O_2S$ (M+H$^+$): 363.1413, found: 363.1418.

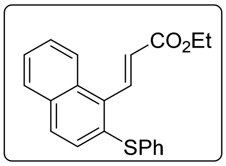

5m: Colorless oil (87%). R_f = 0.3 (hexane/ethyl acetate = 20:1). **^{1}H NMR** (400 MHz, Chloroform-*d*) δ 8.28 (d, *J* = 16.3 Hz, 1H), 8.17 – 8.11 (m, 1H), 7.81 (dd, *J* = 8.1, 1.4 Hz, 1H), 7.69 (d, *J* = 8.9 Hz, 1H), 7.57 – 7.47 (m, 2H), 7.40 – 7.26 (m, 6H), 6.32 (d, *J* = 16.2 Hz, 1H), 4.33 (q, *J* = 7.1 Hz, 2H), 1.38 (t, *J* = 7.1 Hz, 3H). **^{13}C NMR** (101 MHz, $CDCl_3$) δ 166.5, 141.1, 135.4, 133.9, 133.5, 132.6, 131.8, 131.6, 129.4, 128.6, 128.6, 127.5, 127.4, 126.6, 126.4, 125.1, 60.8, 14.5. **IR** (KBr): υ 3056, 2980, 1715, 1638, 1582, 1477, 1440, 1368, 1305, 1281 cm^{-1}. **HRMS** (ESI): Calculated for $C_{21}H_{19}O_2S$ ($M+H^+$): 335.1100, found: 335.1107.

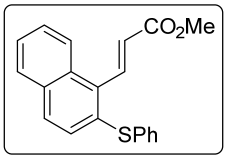

5n: Pale yellow oil (88%). R_f = 0.2 (hexane/ethyl acetate = 20:1). **^{1}H NMR** (400 MHz, Chloroform-*d*) δ 8.30 (d, *J* = 16.3 Hz, 1H), 8.17 – 8.10 (m, 1H), 7.84 – 7.78 (m, 1H), 7.70 (d, *J* = 8.9 Hz, 1H), 7.58 – 7.46 (m, 2H), 7.37 – 7.25 (m, 6H), 6.34 (d, *J* = 16.3 Hz, 1H), 3.87 (s, 3H). **^{13}C NMR** (101 MHz, $CDCl_3$) δ 166.9, 141.4, 135.4, 133.9, 133.4, 132.6, 131.7, 131.6, 129.5, 129.4, 128.6, 128.6, 127.6, 127.4, 126.4, 126.2, 125.1, 52.0. **IR** (KBr): υ 3056, 2948, 2360, 2342, 1721, 1638, 1582, 1434, 1280, 1172 cm^{-1}. **HRMS** (ESI): Calculated for $C_{20}H_{17}O_2S$ ($M+H^+$): 321.0944, found: 321.0950.

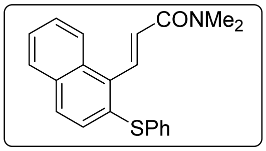

5o: White solid (93%). R_f = 0.2 (hexane/ethyl acetate = 5:1). Mp = 120.7 – 121.4 ^oC. **^{1}H NMR** (400 MHz, Chloroform-*d*) δ 8.19 – 8.11 (m, 2H), 7.80 (dd, *J* = 7.7, 1.8 Hz, 1H), 7.68 (d, *J* = 8.7 Hz, 1H), 7.54 – 7.45 (m, 2H), 7.35 – 7.21 (m, 6H), 6.73 (d, *J* = 15.8 Hz, 1H), 3.07 (d, *J* = 13.5 Hz, 6H). **^{13}C NMR** (101 MHz, $CDCl_3$) δ 166.3, 138.2, 136.0, 135.1, 132.7, 132.6, 132.0, 131.4, 129.3, 129.0, 128.9, 128.4, 127.3, 127.1, 126.6, 126.3, 125.4, 37.5,

36.0. **IR** (KBr): υ 3054, 2928, 1653, 1617, 1582, 1477, 1395, 1140, 1056, 1023 cm^{-1}. **HRMS** (ESI): Calculated for $C_{21}H_{20}NOS$ (M+H$^+$): 334.1260, found: 334.1269.

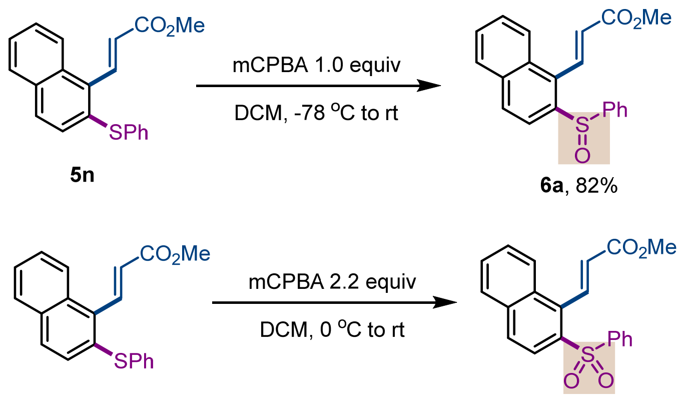

Supplementary Figure 5. Selective oxidation of the *ortho* thiolation product

A Schlenk tube was charged with a solution of **5n** (96.1 mg, 0.3 mmol) in DCM (5 mL). A solution of mCPBA (67.2 mg, 77%, 0.3 mmol, 1.0 equiv) in DCM (5 mL) was then added dropwise at -78 °C. The resulting mixture was allowed to warm to room temperature overnight. Subsequently, the reaction mixture was washed by saturated aq. Na_2CO_3 (10 mL) solution three time. The organic layers were washed with water and brine before they were dried over $MgSO_4$ and concentrated. The residual was then purified by silica gel chromatography (acetone/hexane = 1/5) to afford **6a** (82.3 mg, 82%) as a white solid.

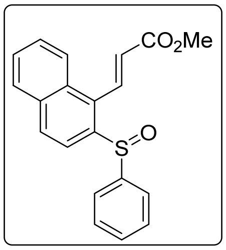

6a: White solid (82%). R_f = 0.2 (hexane/acetone = 5:1). Mp = 151.7 – 152.1 °C. **^{1}H NMR** (400 MHz, Chloroform-*d*) δ 8.26 (d, *J* = 16.3 Hz, 1H), 8.11 – 8.06 (m, 1H), 8.05 – 7.96 (m, 2H), 7.91 – 7.84 (m, 1H), 7.64 – 7.53 (m, 4H), 7.45 – 7.36 (m, 3H), 6.32 (d, *J* = 16.3 Hz, 1H), 3.90 (s, 3H). **^{13}C NMR** (101 MHz, $CDCl_3$) δ 166.0, 144.9, 141.7, 139.1, 134.6, 132.5, 131.2, 130.8, 130.7, 129.4, 128.9, 128.2, 128.2, 127.9, 125.5, 125.3, 120.1, 52.3. **IR** (KBr): υ 3745, 3057, 2950, 1844, 1718, 1675, 1670, 1570, 1419, 1280 cm^{-1}. **HRMS** (ESI): Calculated for $C_{20}H_{16}O_3SNa$(M+Na$^+$): 359.0712, found: 359.0703.

A Schlenk tube was charged with a solution of **5n** (96.1 mg, 0.3 mmol) in DCM (5 mL). A solution of mCPBA (147.9 mg, 77%, 0.66 mmol, 2.2 equiv) in DCM (5 mL) was then added dropwise at 0 °C. The resulting mixture was allowed to warm to room temperature overnight. Subsequently, the reaction mixture was washed by saturated aq. Na_2CO_3 (10 mL) solution three time. The organic layers were washed with water and brine before they were dried over $MgSO_4$ and concentrated. The residual was then purified by silica gel chromatography (acetone/hexane = 1/5) to afford **6b** (85.5 mg, 80%) as a white solid.

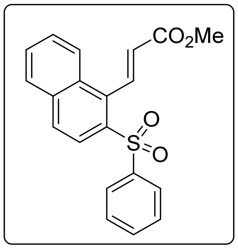

6b: White solid (80%). R_f = 0.25 (hexane/acetone = 5:1). Mp = 130.7 – 131.3 °C. **^{1}H NMR** (400 MHz, $CDCl_3$) δ 8.29 (dd, *J* = 23.9, 12.6 Hz, 2H), 8.04 (dd, *J* = 28.6, 8.7 Hz, 2H), 7.95 – 7.82 (m, 3H), 7.71 – 7.48 (m, 3H), 7.44 (dd, *J* = 10.5, 4.8 Hz, 2H), 5.81 (d, *J* = 16.4 Hz, 1H), 3.88 (s, 3H). **^{13}C NMR** (101 MHz, $CDCl_3$) δ 165.9, 141.5, 140.0, 136.4, 135.6, 135.2, 133.4, 131.1, 129.4, 129.1, 128.9, 128.6, 128.2, 128.0, 127.7, 126.7, 123.8, 52.2. **IR** (KBr): υ 2950, 2360, 2341, 1722, 1582, 1446, 1309, 1280, 1170, 1151 cm^{-1}. **HRMS** (ESI): Calculated for $C_{20}H_{17}O_4S(M+H^+)$: 353.0842, found: 353.0853.

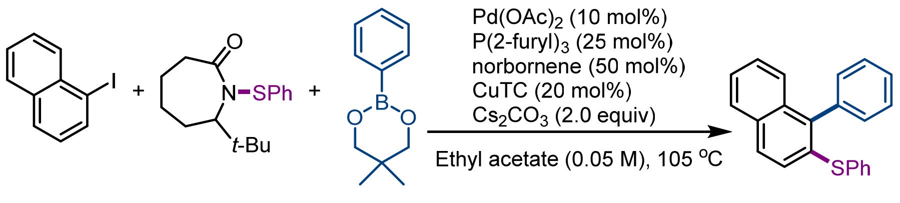

Supplementary Figure 6. *Ipso* functionalization with arylboronates

A flame-dried 7.0 mL vial A was charged with $Pd(OAc)_2$ (4.6 mg, 0.02 mmol, 10 mol%), CuTC (7.6 mg, 0.04 mmol, 20 mol%), TFP (11.6 mg, 0.05 mmol, 25 mol%), phenylboronate **8** (76 mg, 0.4 mmol, 2.0 equiv) and ArI (0.2 mmol, 1.0 equiv). To another 4.0 mL vial B was weighed **3a** (0.6 mmol). Two vials were directly transferred into a

nitrogen-filled glovebox without caps. Then, Cs_2CO_3 (130.4 mg, 0.4 mmol, 2.0 equiv) was added to the vial A. In the third empty 4.0 mL vial C, NBE (18.8 mg, 0.2 mmol) was dissolved in 1.0 mL dry ethyl acetate. Half of this NBE solution (0.5 mL, 0.1 mmol, 50 mol%) was transferred into the vial A. To the 4.0 mL vial B containing **3a** was added 0.75 mL dry ethyl acetate. Two thirds of this **3a** solution (0.5 ml, 0.4 mmol, 2.0 equiv) was transferred into the vial A, before another 3.0 mL dry ethyl acetate was added. Vial A was tightly sealed, transferred out of glovebox and stirred on a pie-block preheated to 105 °C for 12 hours. After completion of the reaction, the mixture was filtered through a thin pad of silica gel. The filter cake was washed with ethyl acetate and the combined filtrate was concentrated. The residue was loaded to a small amount of silica gel and then purified by flash column chromatography to give the desired *ortho* thiolation product.

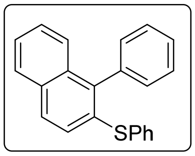

9: Colorless oil (58%). R_f = 0.4 (hexane/ethyl acetate = 20:1). **^{1}H NMR** (400 MHz, $CDCl_3$) δ 7.78 – 7.72 (m, 1H), 7.66 (d, *J* = 8.7 Hz, 1H), 7.44 – 7.35 (m, 4H), 7.31 – 7.14 (m, 10H). **^{13}C NMR** (101 MHz, $CDCl_3$) δ 140.3, 138.8, 136.2, 133.3, 133.0, 132.4, 131.8, 130.4, 129.2, 128.4, 128.4, 128.2, 128.0, 127.8, 127.2, 126.7, 126.5, 125.9. **IR** (KBr): υ 3054, 2953, 1581, 1560, 1505, 1491, 1476, 1439, 1379, 1070 cm^{-1}. **HRMS** (ESI): Calculated for $C_{22}H_{17}S$ ($M+H^+$): 313.1045, found: 313.1055.

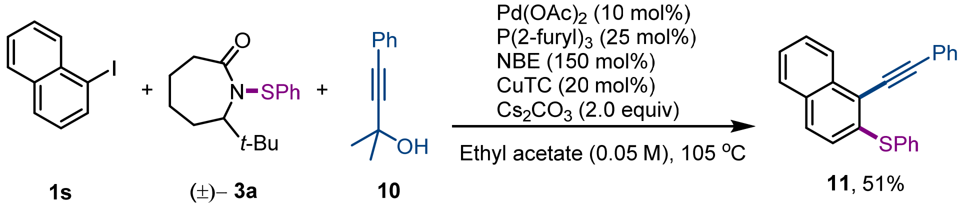

Supplementary Figure 7. *Ipso* Sonogashira quench

A flame-dried 7.0 mL vial A was charged with $Pd(OAc)_2$ (4.6 mg, 0.02 mmol, 10 mol%), CuTC (7.6 mg, 0.04 mmol, 20 mol%), TFP (11.6 mg, 0.05 mmol, 25 mol%), **10** (64.1 mg, 0.4 mmol, 2.0 equiv) and ArI (0.2 mmol, 1.0 equiv). To another 4.0 mL vial B was weighed thiolation reagent (0.6 mmol). Two vials were directly transferred into a nitrogen-filled glovebox without caps. Then, NBE (28.2 mg, 0.3 mmol, 150 mol%) and Cs_2CO_3 (130.4 mg, 0.4

mmol, 2.0 equiv) was added to the vial A. To the 4.0 mL vial B containing thiolation reagent was added 0.75 mL dry ethyl acetate. 0.5 mL of this thiolation reagent solution (0.4 mmol, 2.0 equiv) was transferred into the vial A, before another 3.5 mL dry ethyl acetate was added. Vial A was tightly sealed, transferred out of glovebox and stirred on a pie-block preheated to 105 °C for 12 hours. After completion of the reaction, the mixture was filtered through a thin pad of silica gel. The filter cake was washed with ethyl acetate and the combined filtrate was concentrated. The residue was loaded to a small amount of silica gel and then purified by flash column chromatography on silica gel to give the desired *ortho* thiolation product.

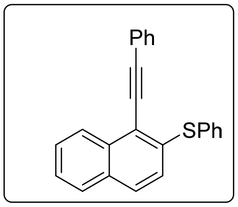

11: Yellow oil (51%). R_f = 0.4 (hexane/ethyl acetate = 20:1). **^{1}H NMR** (400 MHz, Chloroform-*d*) δ 8.42 (dd, *J* = 8.4, 1.1 Hz, 1H), 7.80 – 7.75 (m, 1H), 7.69 – 7.63 (m, 3H), 7.60 (ddd, *J* = 8.3, 6.9, 1.3 Hz, 1H), 7.55 – 7.51 (m, 2H), 7.48 (ddd, *J* = 8.1, 6.9, 1.3 Hz, 1H), 7.43 – 7.33 (m, 6H), 7.13 (d, *J* = 8.7 Hz, 1H). **^{13}C NMR** (101 MHz, $CDCl_3$) δ 139.1, 133.7, 133.3, 131.7, 131.4, 129.5, 128.7, 128.6, 128.4, 128.2, 128.1, 127.5, 126.1, 126.1, 125.9, 123.3, 119.2, 101.2, 85.5. **IR** (KBr): υ 3055, 2921, 1616, 1581, 1555, 1489, 1129, 1085, 1068, 1024 cm^{-1}. **HRMS** (ESI): Calculated for $C_{24}H_{17}S$ $(M+H^+)$: 337.1045, found: 337.1055.

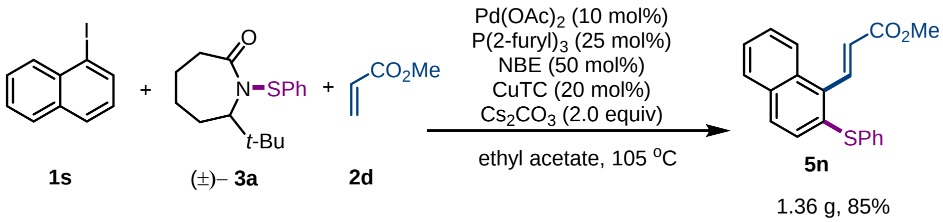

Supplementary Figure 8. Gram-scale reaction

A flame-dried 100 mL vessel was charged with $Pd(OAc)_2$ (115 mg, 0.5 mmol, 10 mol%), CuTC (190 mg, 1.0 mmol, 20 mol%), TFP (290 mg, 1.25 mmol, 25 mol%), sulfenamide **3a** (2.77 g, 10.0 mmol, 2.0 equiv) and ArI **1s** (1.27 g, 5.0 mmol, 1.0 equiv). The vessel was directly transferred into a nitrogen-filled glovebox without caps. Then, NBE (235 mg, 2.5 mmol, 50 mol%) and Cs_2CO_3 (3.26 g, 10 mmol, 2.0 equiv) was added. 100 mL dry ethyl acetate was added before acrylate **2d** (860 mg, 10 mmol, 2.0 equiv) was added. Then the vessel was tightly sealed, transferred

out of glovebox and stirred in an oil bath preheated to 105 °C for 12 hours. After completion of the reaction, the mixture was filtered through a thin pad of silica gel. The filter cake was washed with ethyl acetate and the combined filtrate was concentrated. The residue was loaded to a small amount of silica gel and subjected to flash column chromatography to give the desired *ortho* thiolation product **5n** (1.36 g, 85%).

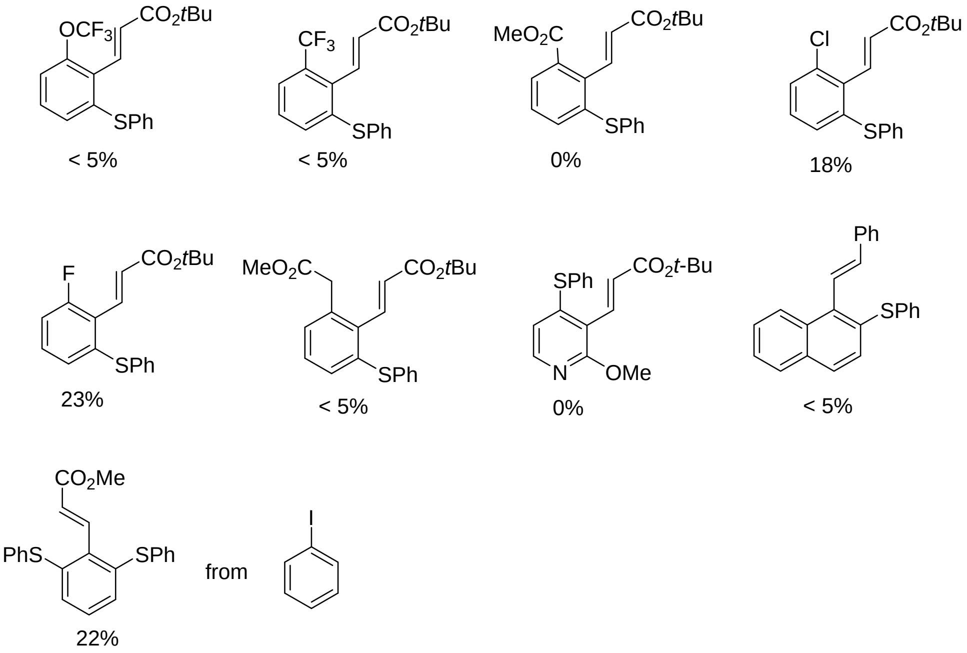

Supplementary Figure 9. Less successful or unsuccessful substrates

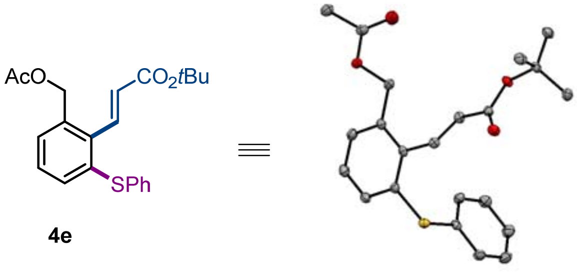

CCDC: 1906772

Identification code	RHL-key
Empirical formula	$C_{22}H_{24}O_4S$
Formula weight	384.47
Temperature/K	100(2)
Crystal system	triclinic
Space group	P-1
a/Å	5.5071(5)
b/Å	11.5833(10)
c/Å	15.6570(14)
α/°	97.152(2)
β/°	93.856(2)
γ/°	102.375(2)
Volume/$Å^3$	963.41(15)
Z	2
$\rho_{calc}g/cm^3$	1.325
μ/mm^{-1}	0.193
F(000)	408.0
Crystal size/mm^3	0.1 × 0.1 × 0.03
Radiation	MoKα (λ = 0.71073)
2Θ range for data collection/°	4.786 to 55.092
Index ranges	$-7 \le h \le 7$, $-15 \le k \le 15$, $-20 \le l \le 20$
Reflections collected	27080
Independent reflections	4421 [R_{int} = 0.0392, R_{sigma} = 0.0311]
Data/restraints/parameters	4421/0/248
Goodness-of-fit on F^2	1.031
Final R indexes [I>=2σ (I)]	R_1 = 0.0377, wR_2 = 0.0811
Final R indexes [all data]	R_1 = 0.0538, wR_2 = 0.0877
Largest diff. peak/hole / e $Å^{-3}$	0.38/-0.24

Supplementary Figure 10. X-ray structure and crystallographic data of **4e**

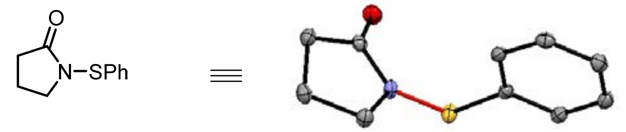

S11

CCDC: 1906766

Identification code	RHL-5ring
Empirical formula	$C_{10}H_{11}NOS$
Formula weight	193.26
Temperature/K	100(2)
Crystal system	orthorhombic
Space group	$P2_12_12_1$
a/Å	9.3377(19)
b/Å	9.727(2)
c/Å	9.976(2)
α/°	90
β/°	90
γ/°	90
Volume/Å^3	906.1(3)
Z	4
ρ_{calc}g/cm^3	1.417
μ/mm^{-1}	0.312
F(000)	408.0
Crystal size/mm^3	0.07 × 0.05 × 0.03
Radiation	MoKα (λ = 0.71073)
2Θ range for data collection/°	5.85 to 60.79
Index ranges	-13 ≤ h ≤ 12, -13 ≤ k ≤ 8, -12 ≤ l ≤ 14
Reflections collected	7220
Independent reflections	2454 [R_{int} = 0.0249, R_{sigma} = 0.0318]
Data/restraints/parameters	2454/0/118
Goodness-of-fit on F^2	1.100
Final R indexes [I>=2σ (I)]	R_1 = 0.0318, wR_2 = 0.0775
Final R indexes [all data]	R_1 = 0.0386, wR_2 = 0.0831
Largest diff. peak/hole / e Å^{-3}	0.41/-0.31
Flack parameter	0.01(3)

Supplementary Figure 11. X-ray structure and crystallographic data of **S11**

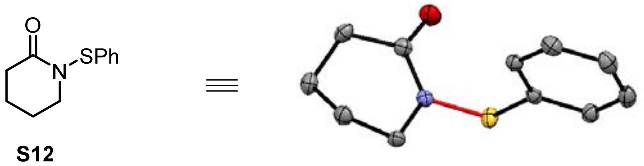

S12

CCDC: 1906767

Identification code	RHL-6ring
Empirical formula	$C_{11}H_{13}NOS$
Formula weight	207.28
Temperature/K	100(2)
Crystal system	tetragonal
Space group	$P4_2/n$
a/Å	16.5787(10)
b/Å	16.5787(10)
c/Å	7.4975(5)
α/°	90
β/°	90
γ/°	90
Volume/Å^3	2060.7(3)
Z	8
ρ_{calc}g/cm^3	1.336
μ/mm^{-1}	0.279
F(000)	880.0
Crystal size/mm^3	0.12 × 0.1 × 0.08
Radiation	MoKα (λ = 0.71073)
2Θ range for data collection/°	4.914 to 61.264
Index ranges	$-21 \leq h \leq 20$, $-22 \leq k \leq 19$, $-10 \leq l \leq 10$
Reflections collected	16147
Independent reflections	2895 [R_{int} = 0.0293, R_{sigma} = 0.0291]
Data/restraints/parameters	2895/0/127
Goodness-of-fit on F^2	1.038
Final R indexes [I>=2σ (I)]	R_1 = 0.0374, wR_2 = 0.0798
Final R indexes [all data]	R_1 = 0.0557, wR_2 = 0.0862
Largest diff. peak/hole / e Å^{-3}	0.31/-0.17

Supplementary Figure 12. X-ray structure and crystallographic data of **S12**

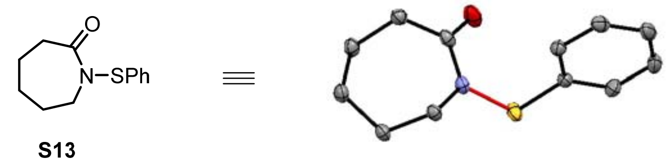

S13

CCDC: 1906768

Identification code	RHL-7membered
Empirical formula	$C_{12}H_{15}NOS$
Formula weight	221.31
Temperature/K	100(2)
Crystal system	monoclinic
Space group	$P2_1/c$
a/Å	9.1705(6)
b/Å	8.8692(6)
c/Å	14.0231(9)
α/°	90
β/°	92.720(2)
γ/°	90
Volume/Å^3	1139.28(13)
Z	4
ρ_{calc}g/cm^3	1.290
μ/mm^{-1}	0.257
F(000)	472.0
Crystal size/mm^3	0.2 × 0.15 × 0.1
Radiation	MoKα (λ = 0.71073)
2Θ range for data collection/°	5.436 to 60.54
Index ranges	$-12 \leq h \leq 12$, $-12 \leq k \leq 11$, $-19 \leq l \leq 19$
Reflections collected	16162
Independent reflections	3136 [R_{int} = 0.0263, R_{sigma} = 0.0208]
Data/restraints/parameters	3136/27/136
Goodness-of-fit on F^2	1.049
Final R indexes [I>=2σ (I)]	R_1 = 0.0331, wR_2 = 0.0780
Final R indexes [all data]	R_1 = 0.0418, wR_2 = 0.0823
Largest diff. peak/hole / e Å^{-3}	0.35/-0.20

Supplementary Figure 13. X-ray structure and crystallographic data of **S13**

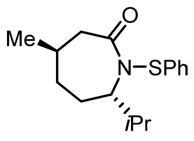

(±)- **S19**

≡

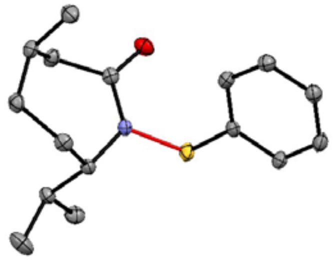

CCDC: 1906771

Identification code	zhe1
Empirical formula	$C_{16}H_{23}NOS$
Formula weight	277.41
Temperature/K	293(2)
Crystal system	orthorhombic
Space group	$P2_12_12_1$
a/Å	9.4262(12)
b/Å	11.2142(14)
c/Å	14.1135(17)
α/°	90
β/°	90
γ/°	90
Volume/$Å^3$	1491.9(3)
Z	4
$\rho_{calc}g/cm^3$	1.235
μ/mm^{-1}	0.210
F(000)	600.0
Crystal size/mm^3	? × ? × ?
Radiation	MoKα (λ = 0.71075)
2Θ range for data collection/°	6.342 to 61.016
Index ranges	-13 ≤ h ≤ 13, -16 ≤ k ≤ 15, -19 ≤ l ≤ 19
Reflections collected	23427
Independent reflections	4390 [R_{int} = 0.0213, R_{sigma} = 0.0156]
Data/restraints/parameters	4390/0/176
Goodness-of-fit on F^2	1.056
Final R indexes [I>=2σ (I)]	R_1 = 0.0257, wR_2 = 0.0671
Final R indexes [all data]	R_1 = 0.0269, wR_2 = 0.0678
Largest diff. peak/hole / e $Å^{-3}$	0.30/-0.20
Flack parameter	0.01(6)

Supplementary Figure 14. X-ray structure and crystallographic data of **S19**

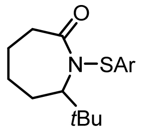

Ar = 4-OMeC_6H_4

3c

≡

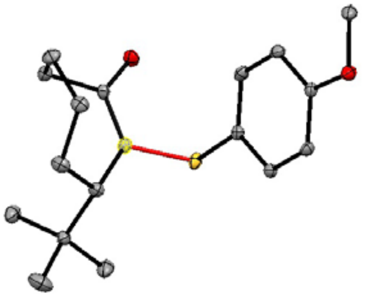

CCDC: 1906769

Identification code	RHL-7ring
Empirical formula	$C_{17}H_{25}NO_2S$
Formula weight	307.44
Temperature/K	100(2)
Crystal system	orthorhombic
Space group	Pbca
a/Å	10.0223(5)
b/Å	16.1566(8)
c/Å	20.2915(10)
α/°	90
β/°	90
γ/°	90
Volume/$Å^3$	3285.7(3)
Z	8
ρ_{calc}g/cm^3	1.243
μ/mm^{-1}	0.202
F(000)	1328.0
Crystal size/mm^3	0.1 × 0.1 × 0.1
Radiation	MoKα (λ = 0.71073)
2Θ range for data collection/°	5.042 to 61.126
Index ranges	$-11 \leq h \leq 14$, $-22 \leq k \leq 22$, $-24 \leq l \leq 28$
Reflections collected	28148
Independent reflections	4691 [R_{int} = 0.0299, R_{sigma} = 0.0274]
Data/restraints/parameters	4691/0/194
Goodness-of-fit on F^2	1.032
Final R indexes [I>=2σ (I)]	R_1 = 0.0405, wR_2 = 0.0968
Final R indexes [all data]	R_1 = 0.0591, wR_2 = 0.1059
Largest diff. peak/hole / e $Å^{-3}$	0.44/-0.22

Supplementary Figure 15. X-ray structure and crystallographic data of **3c**

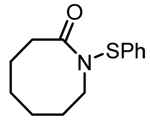

S14

≡

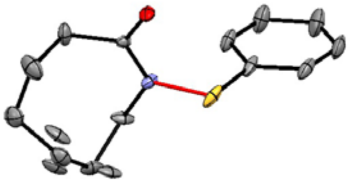

CCDC: 1906770

Identification code	RHL-8membered
Empirical formula	$C_{13}H_{17}NOS$
Formula weight	235.33
Temperature/K	100(2)
Crystal system	monoclinic
Space group	$P2_1/n$
a/Å	11.001(2)
b/Å	5.9033(12)
c/Å	18.619(4)
α/°	90
β/°	90.427(7)
γ/°	90
Volume/Å^3	1209.2(4)
Z	4
ρ_{calc}g/cm^3	1.293
μ/mm^{-1}	0.246
F(000)	504.0
Crystal size/mm^3	0.1 × 0.1 × 0.1
Radiation	MoKα (λ = 0.71073)
2Θ range for data collection/°	4.314 to 49.032
Index ranges	$-12 \leq h \leq 12, -6 \leq k \leq 6, -21 \leq l \leq 21$
Reflections collected	9404
Independent reflections	1999 [R_{int} = 0.0658, R_{sigma} = 0.0577]
Data/restraints/parameters	1999/93/172
Goodness-of-fit on F^2	1.052
Final R indexes [I>=2σ (I)]	R_1 = 0.0797, wR_2 = 0.2105
Final R indexes [all data]	R_1 = 0.1016, wR_2 = 0.2246
Largest diff. peak/hole / e Å^{-3}	0.45/-0.43

Supplementary Figure 16. X-ray structure and crystallographic data of **S14**

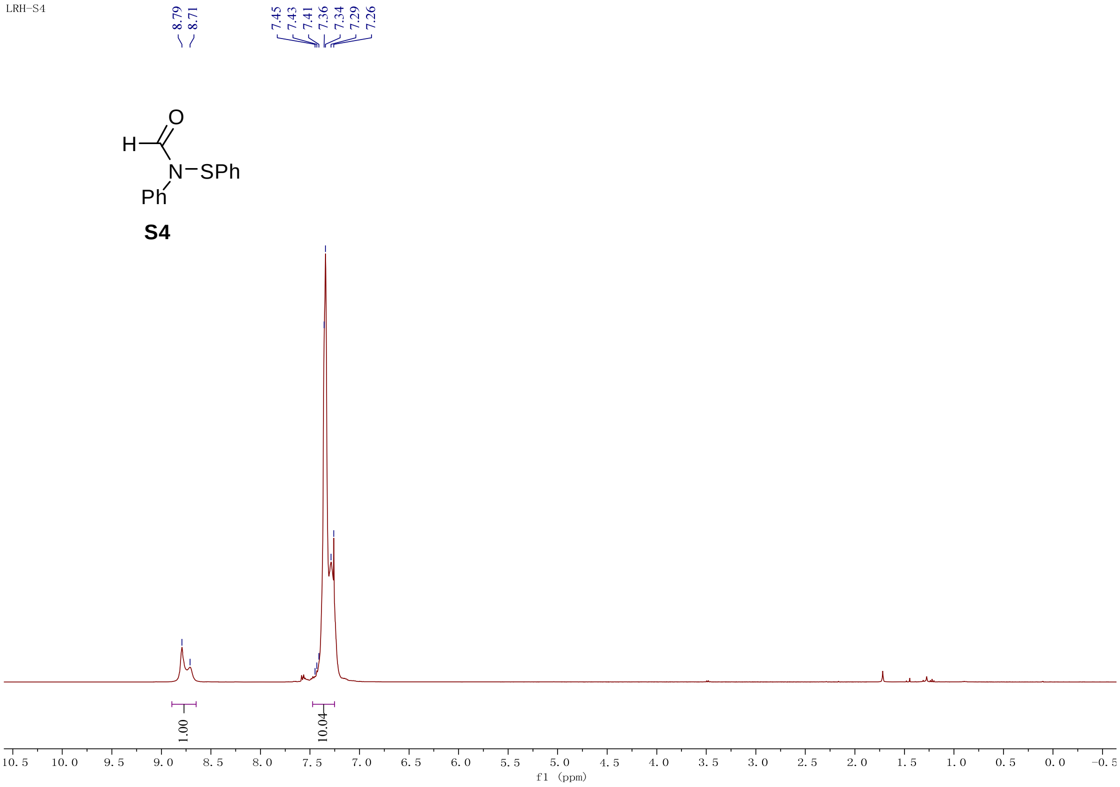

Supplementary Figure 17. ^{1}H NMR Spectrum of **S4**

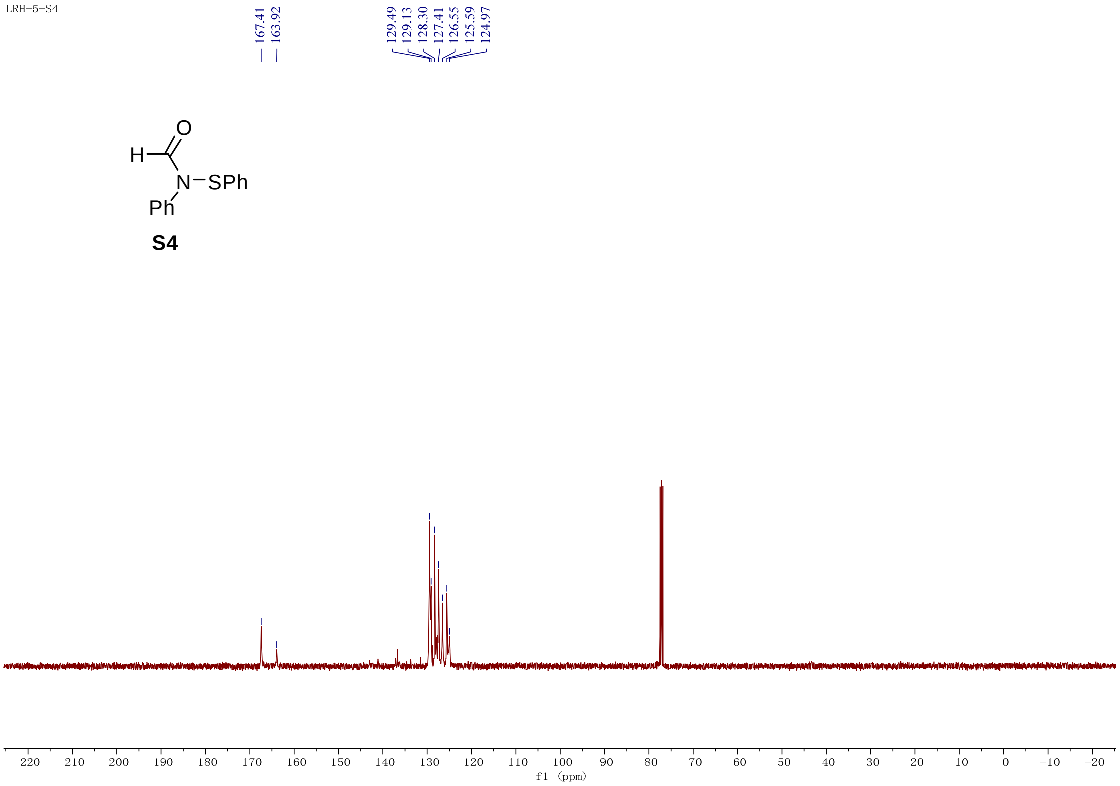

Supplementary Figure 18. ^{13}C NMR Spectrum of **S4**

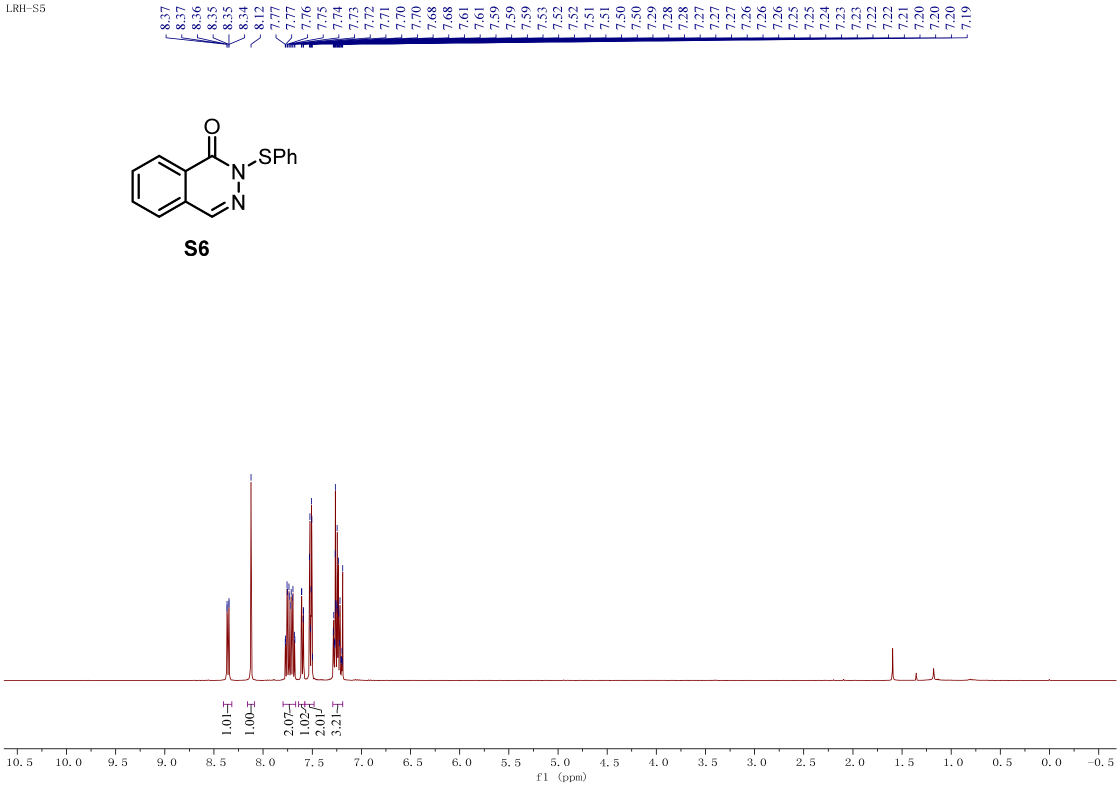

Supplementary Figure 19. ^{1}H NMR Spectrum of **S6**

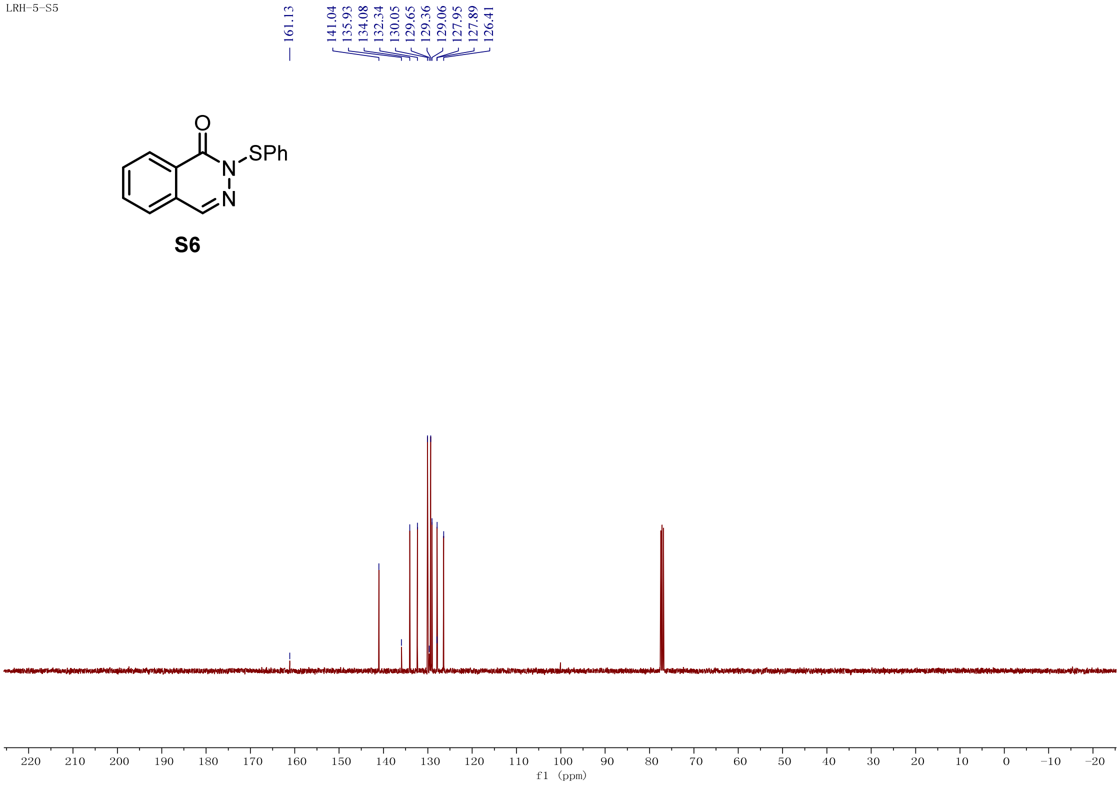

Supplementary Figure 20. ^{13}C NMR Spectrum of **S6**

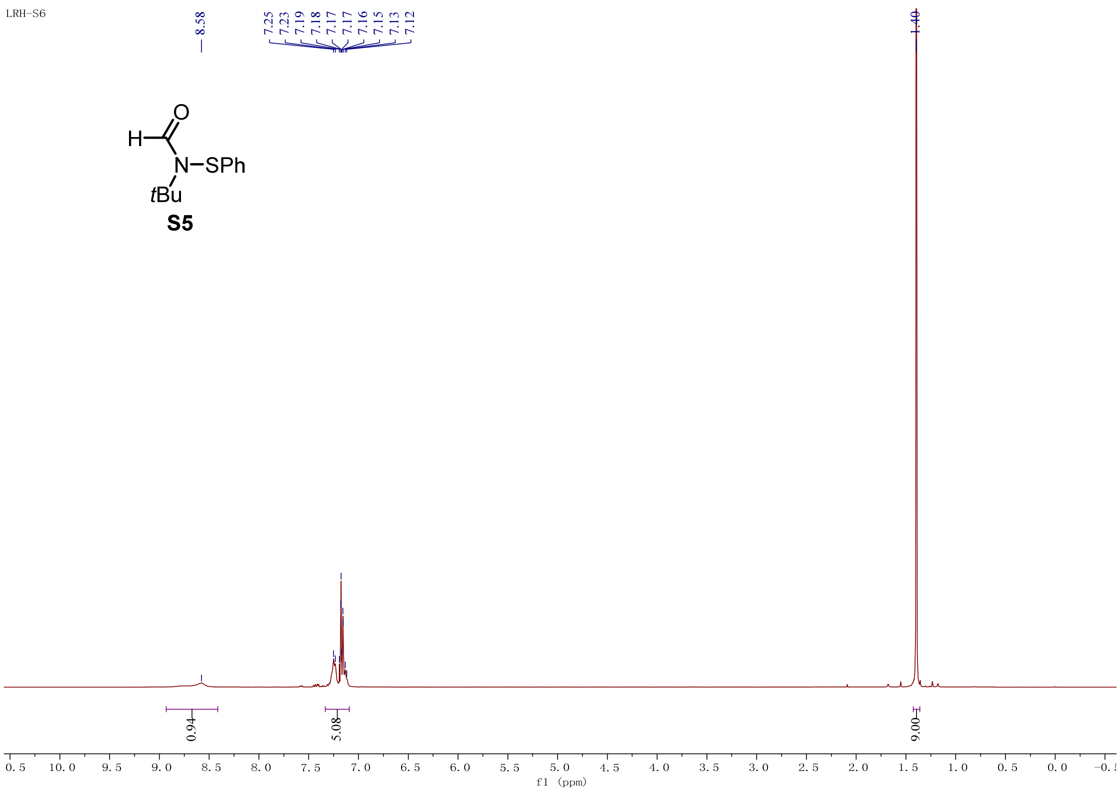

Supplementary Figure 21. ^{1}H NMR Spectrum of **S5**

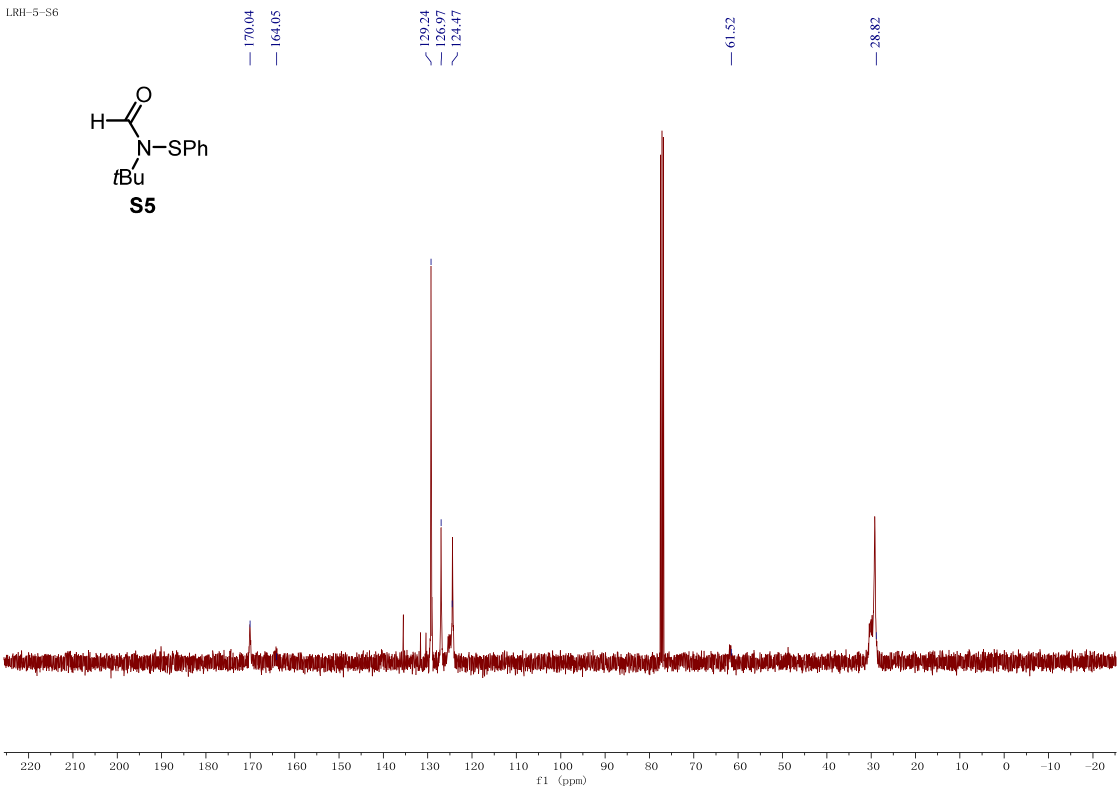

Supplementary Figure 22. ^{13}C NMR Spectrum of **S5**

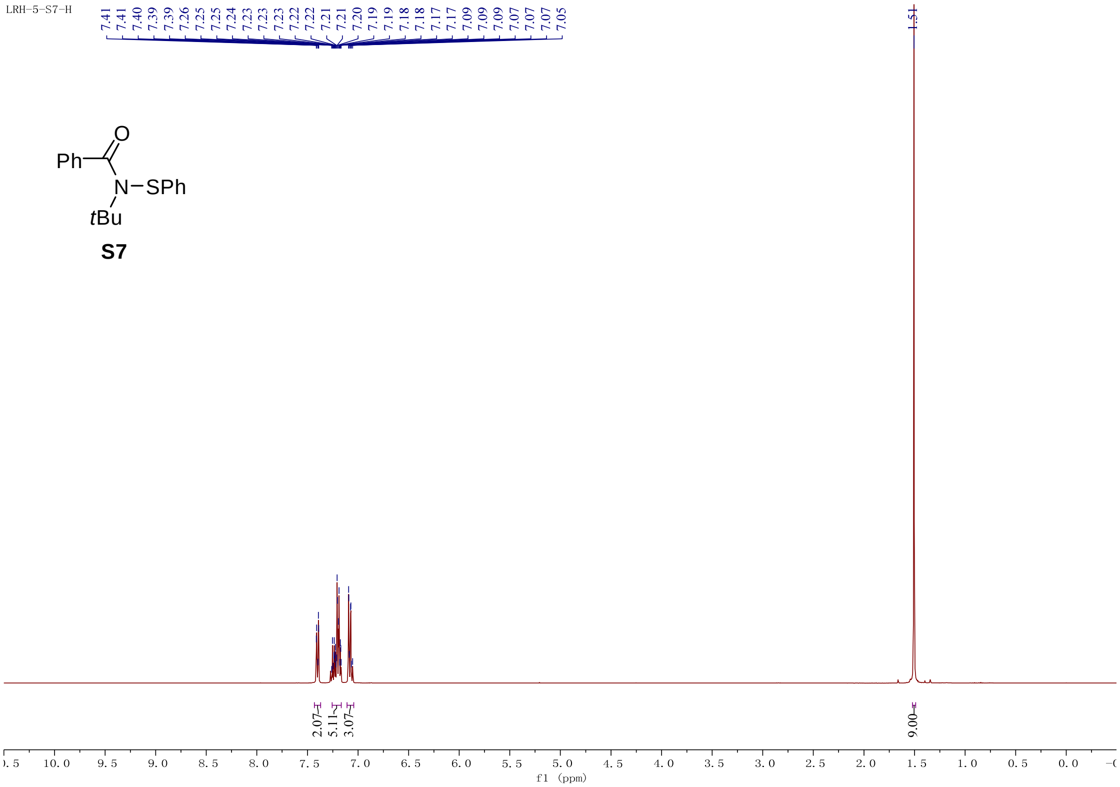

Supplementary Figure 23. ^{1}H NMR Spectrum of **S7**

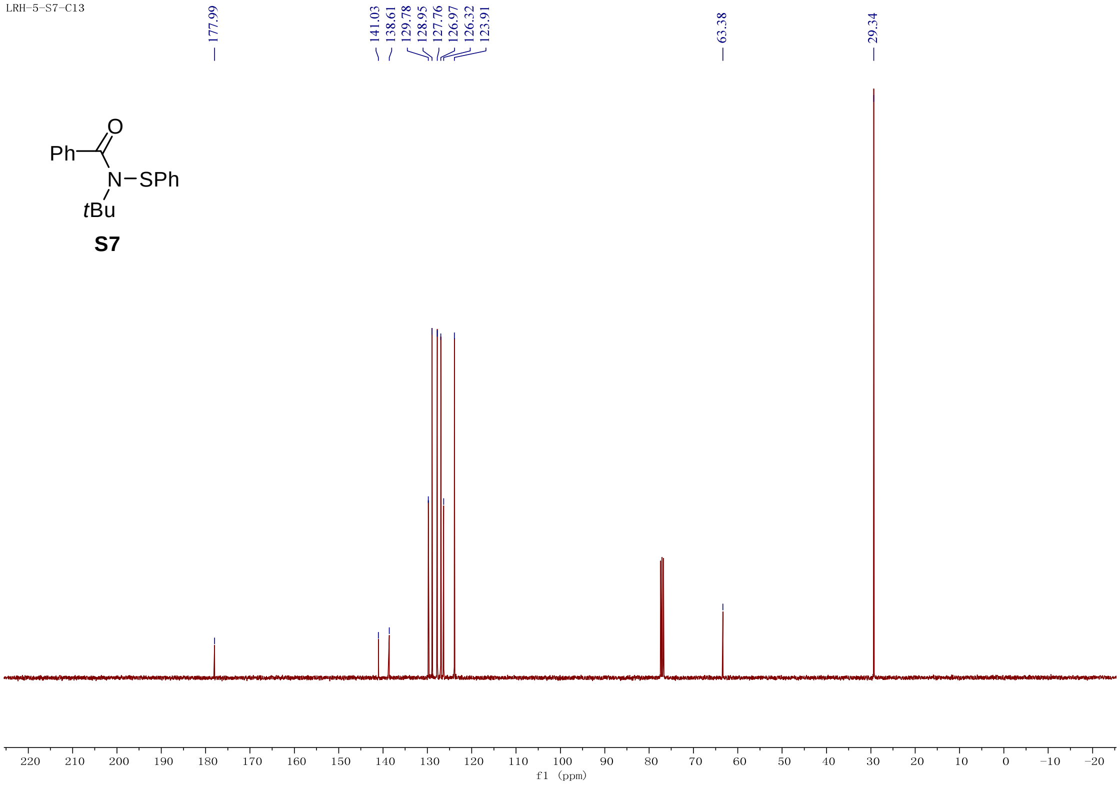

Supplementary Figure 24. ^{13}C NMR Spectrum of **S7**

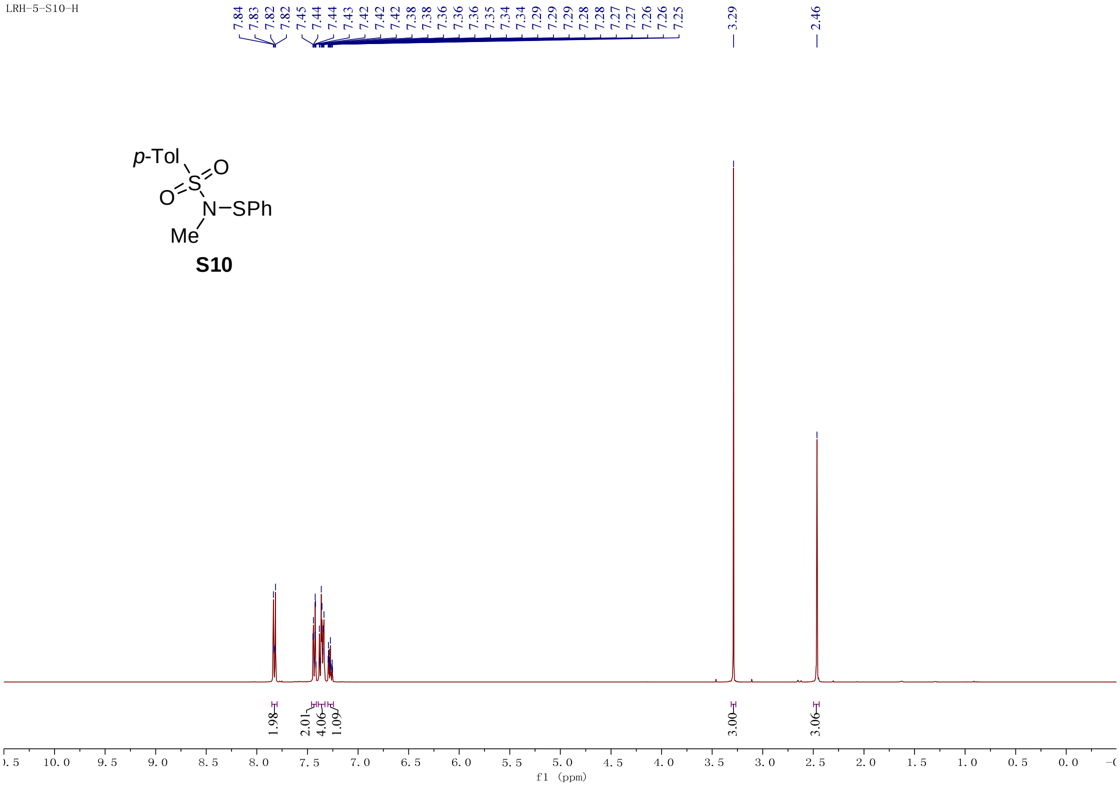

Supplementary Figure 25. ^{1}H NMR Spectrum of **S10**

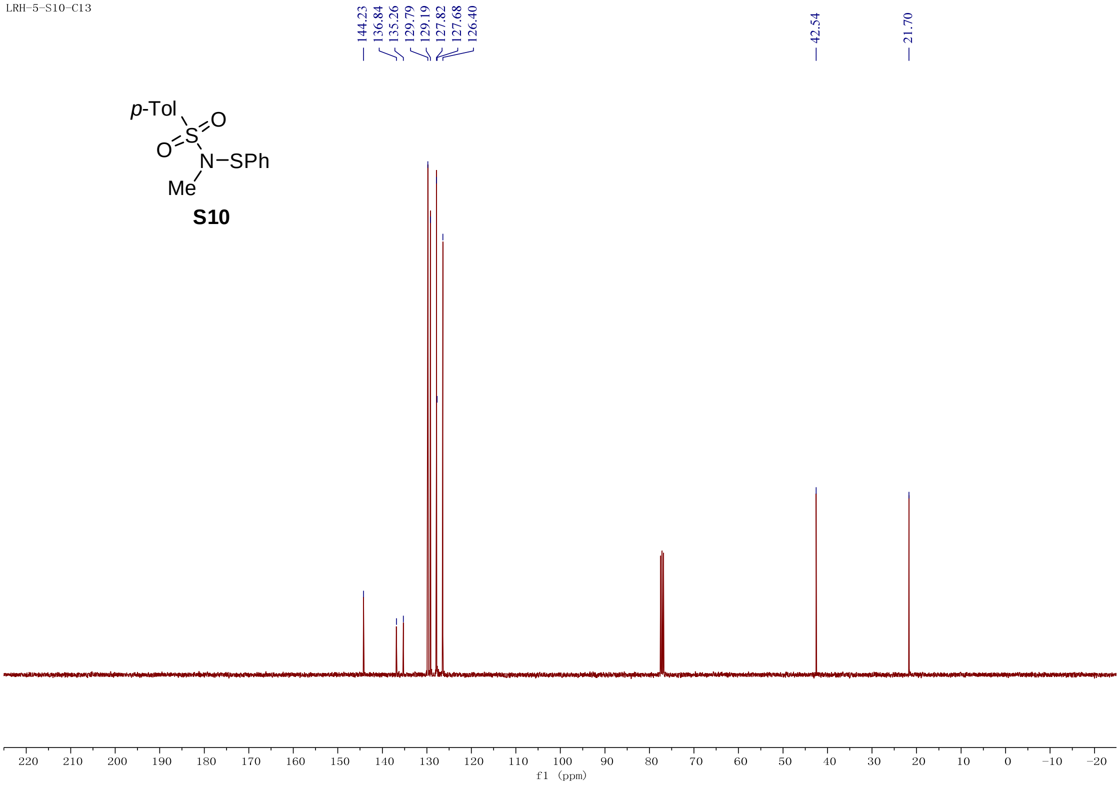

Supplementary Figure 26. ^{13}C NMR Spectrum of **S10**

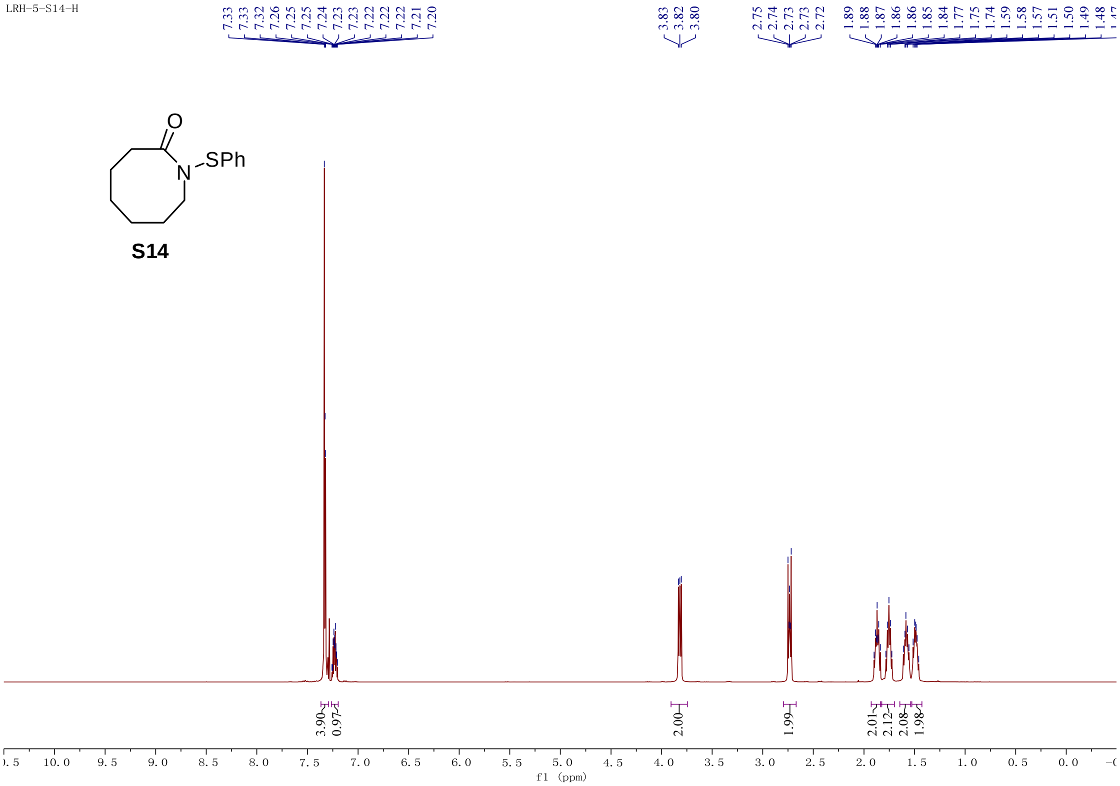

Supplementary Figure 27. ^{1}H NMR Spectrum of **S14**

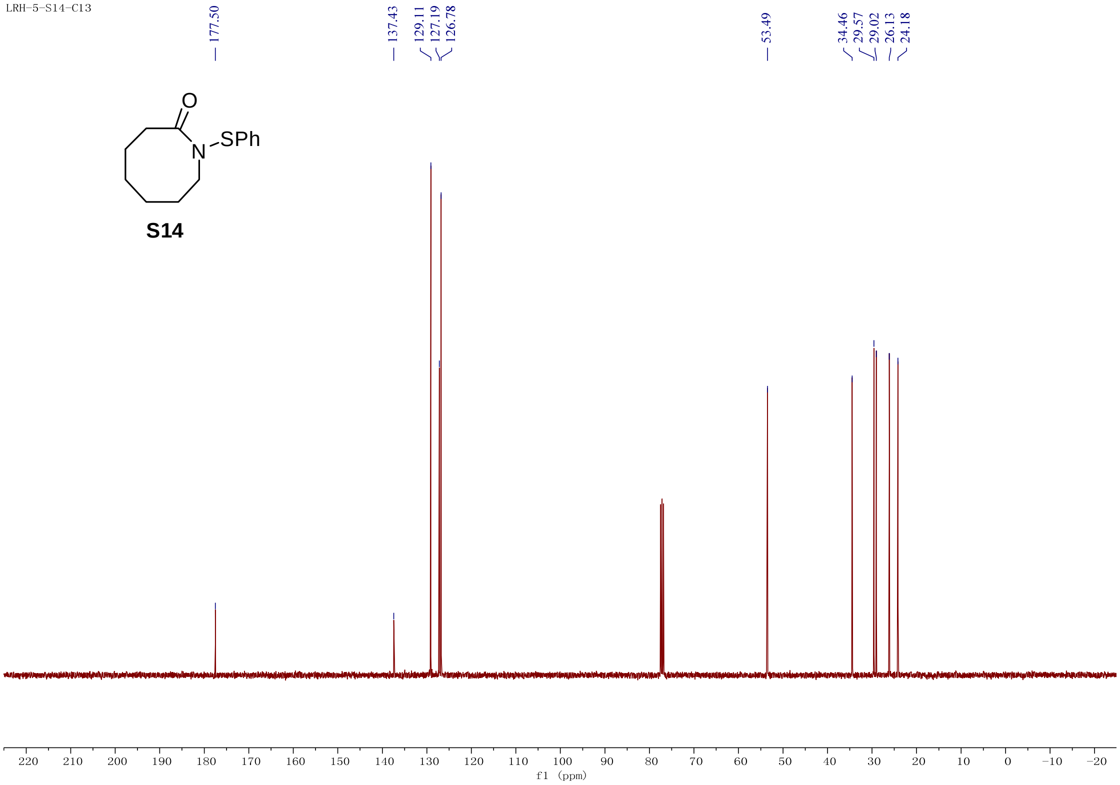

Supplementary Figure 28. ^{13}C NMR Spectrum of **S14**

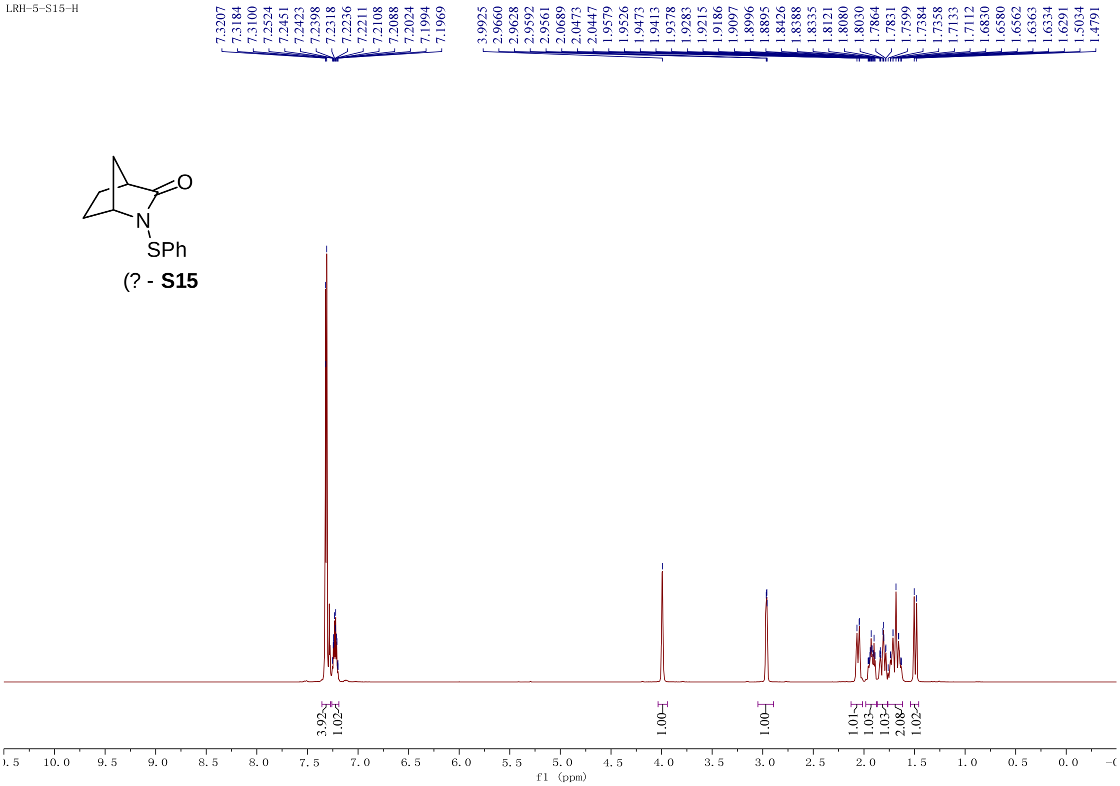

Supplementary Figure 29. ^{1}H NMR Spectrum of **S15**

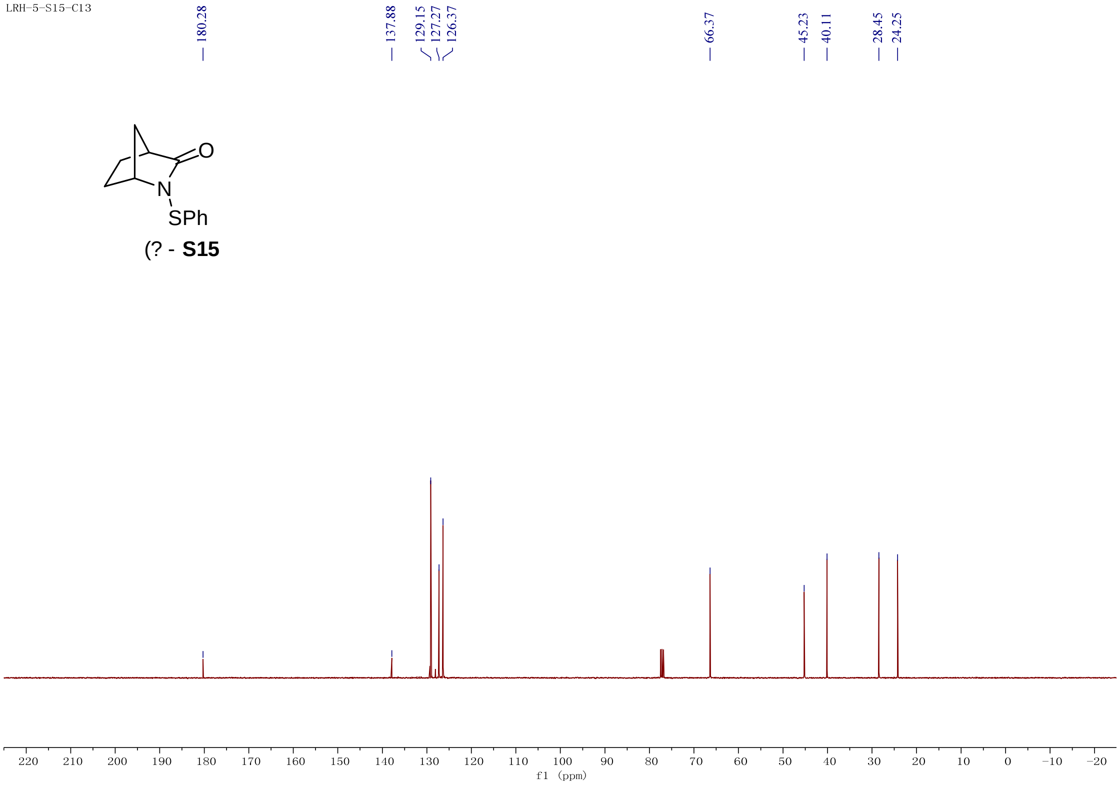

Supplementary Figure 30. ^{13}C NMR Spectrum of **S15**

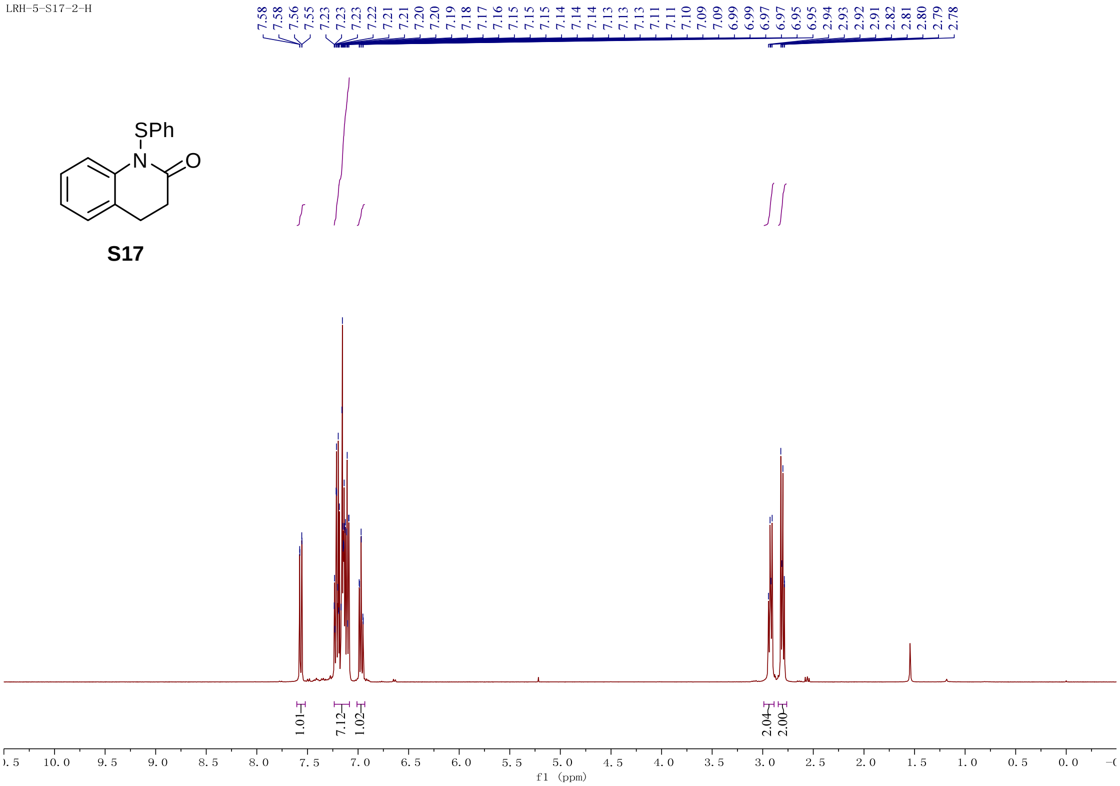

Supplementary Figure 31. ^{1}H NMR Spectrum of **S17**

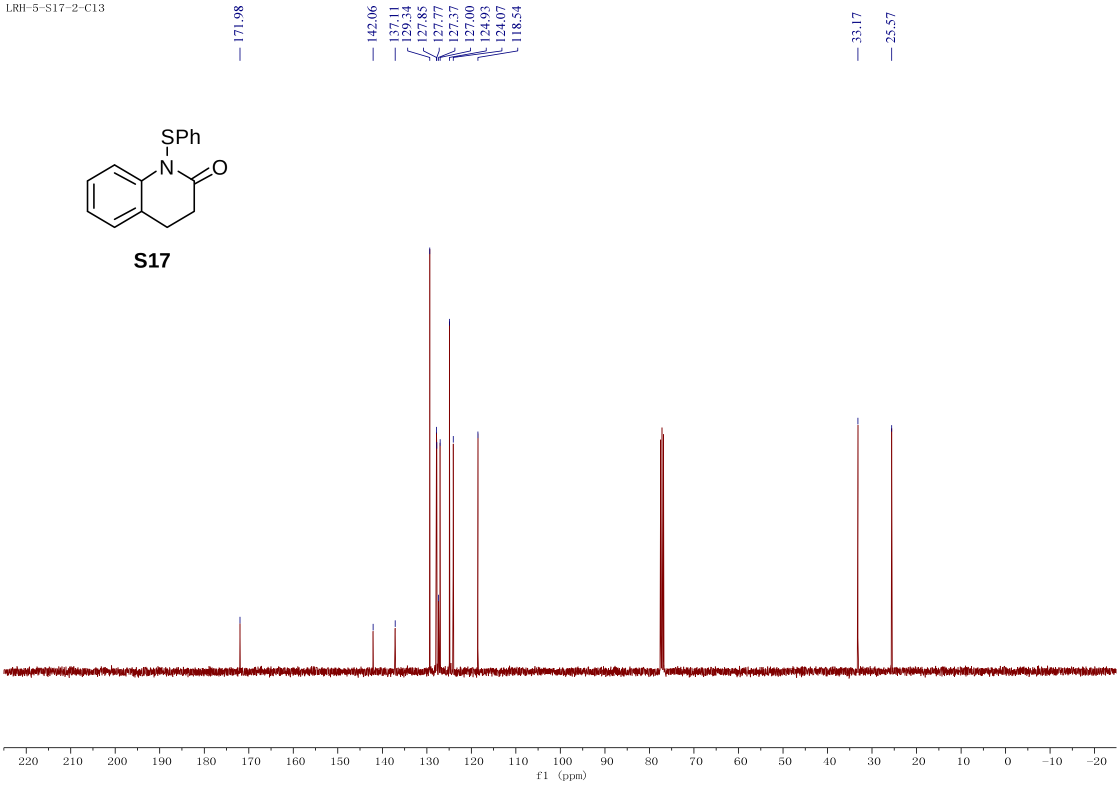

Supplementary Figure 32. ^{13}C NMR Spectrum of **S17**

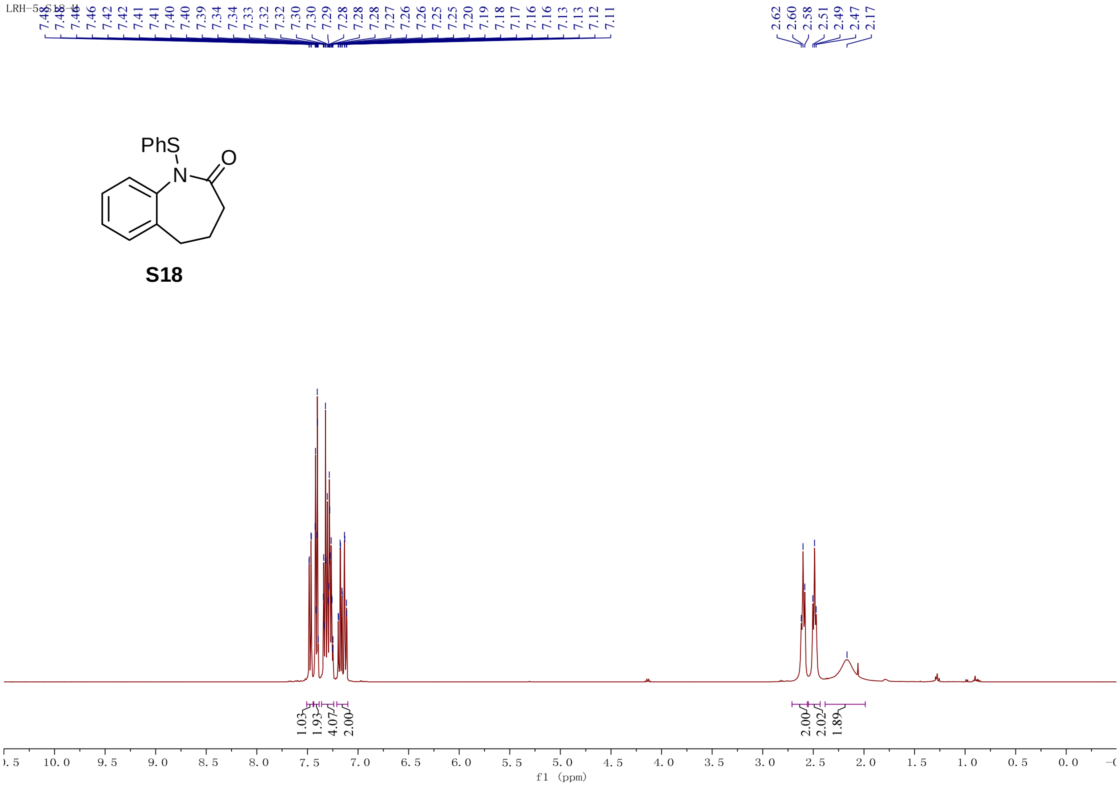

Supplementary Figure 33. ^{1}H NMR Spectrum of **S18**

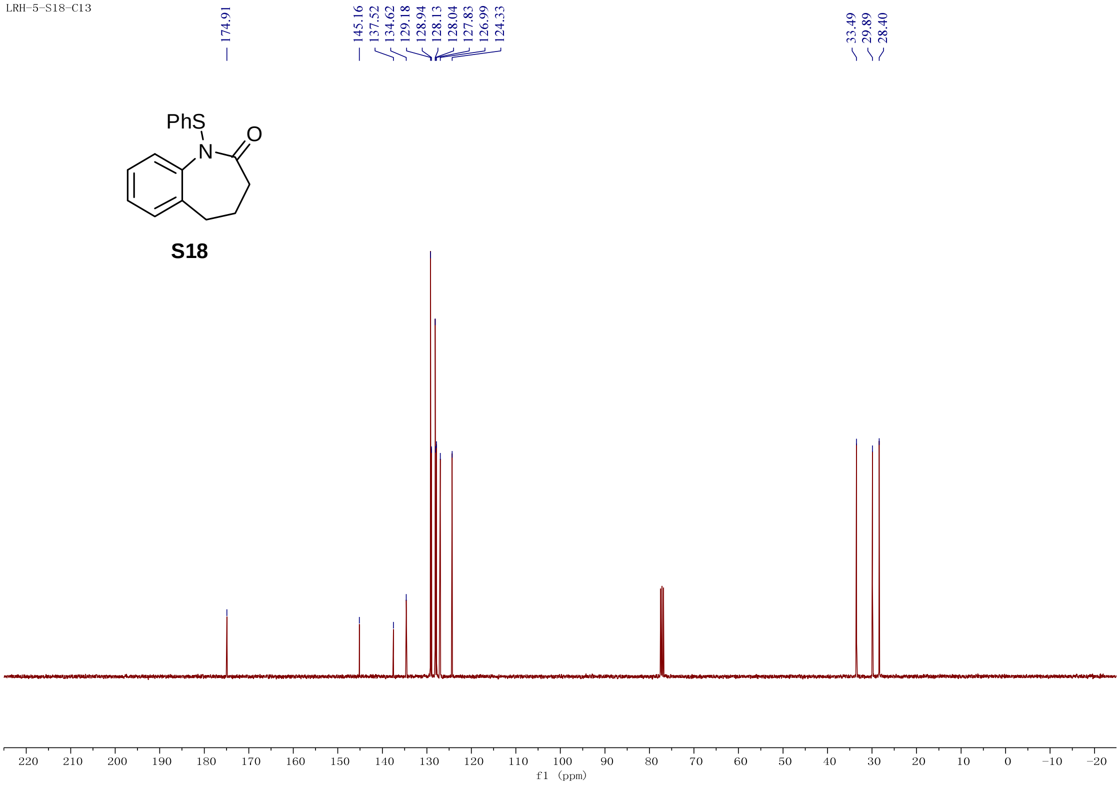

Supplementary Figure 34. ^{13}C NMR Spectrum of **S18**

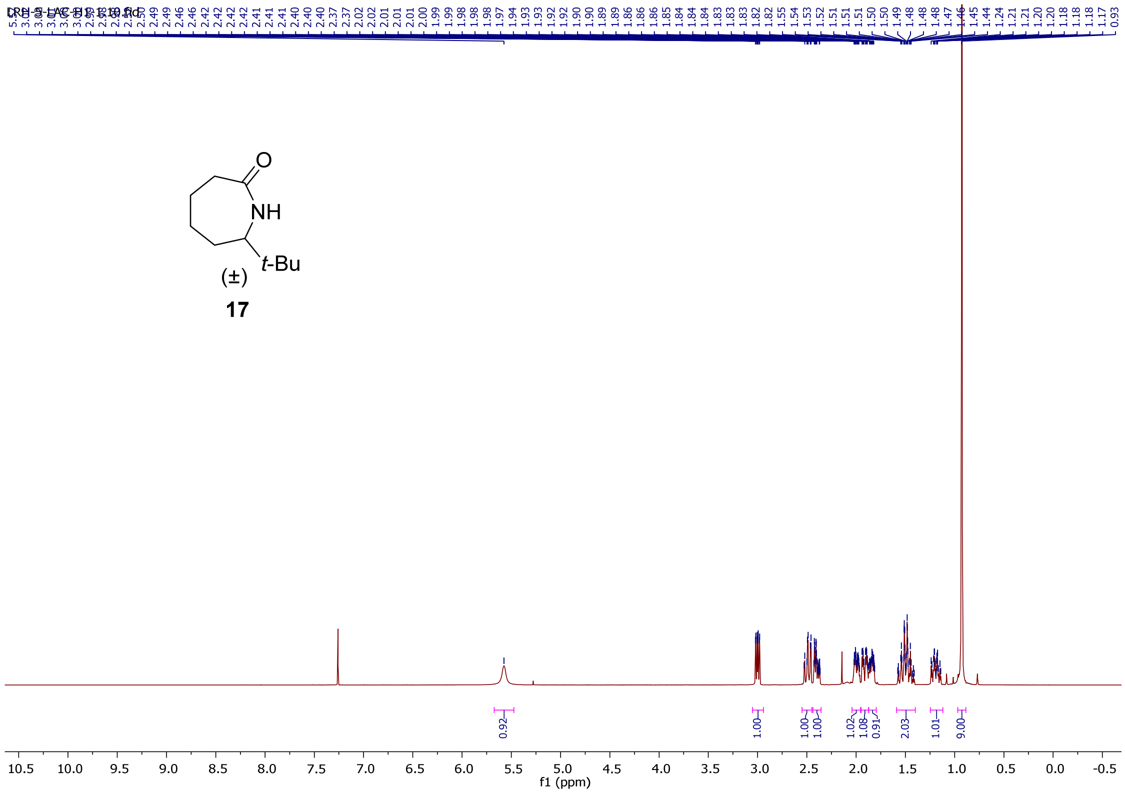

Supplementary Figure 35. ^{1}H NMR Spectrum of **17**

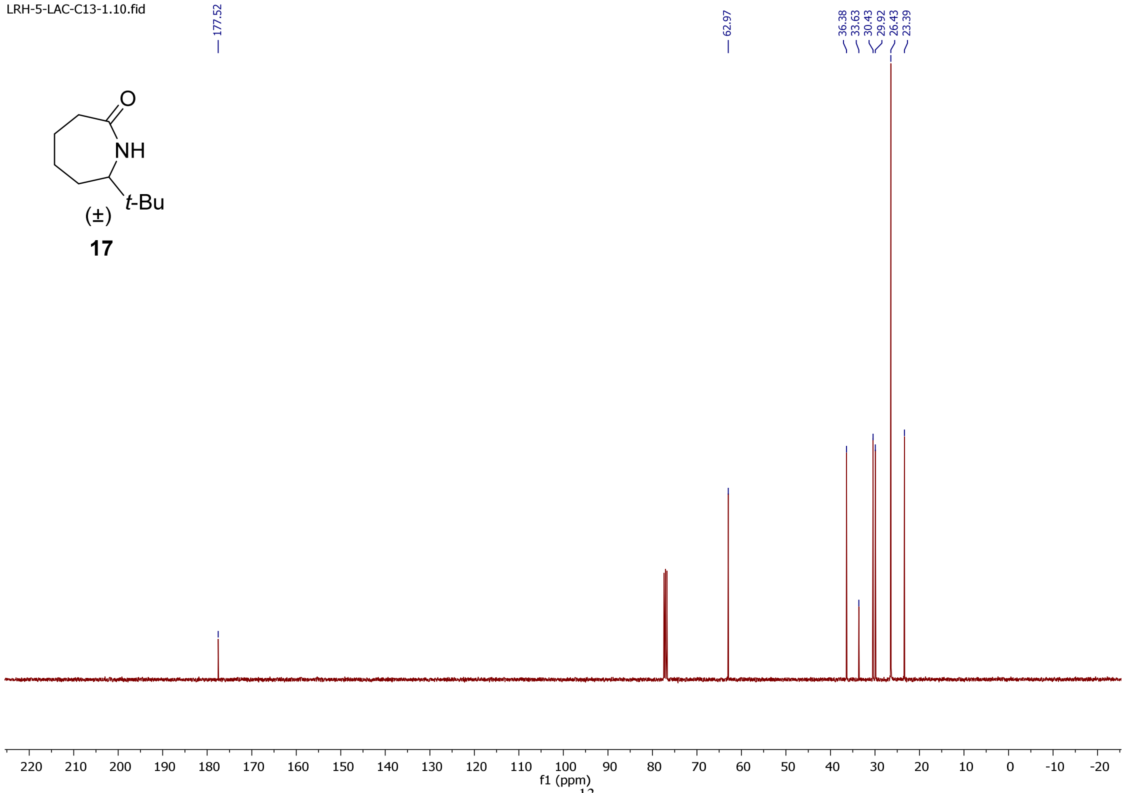

Supplementary Figure 36. ^{13}C NMR Spectrum of **17**

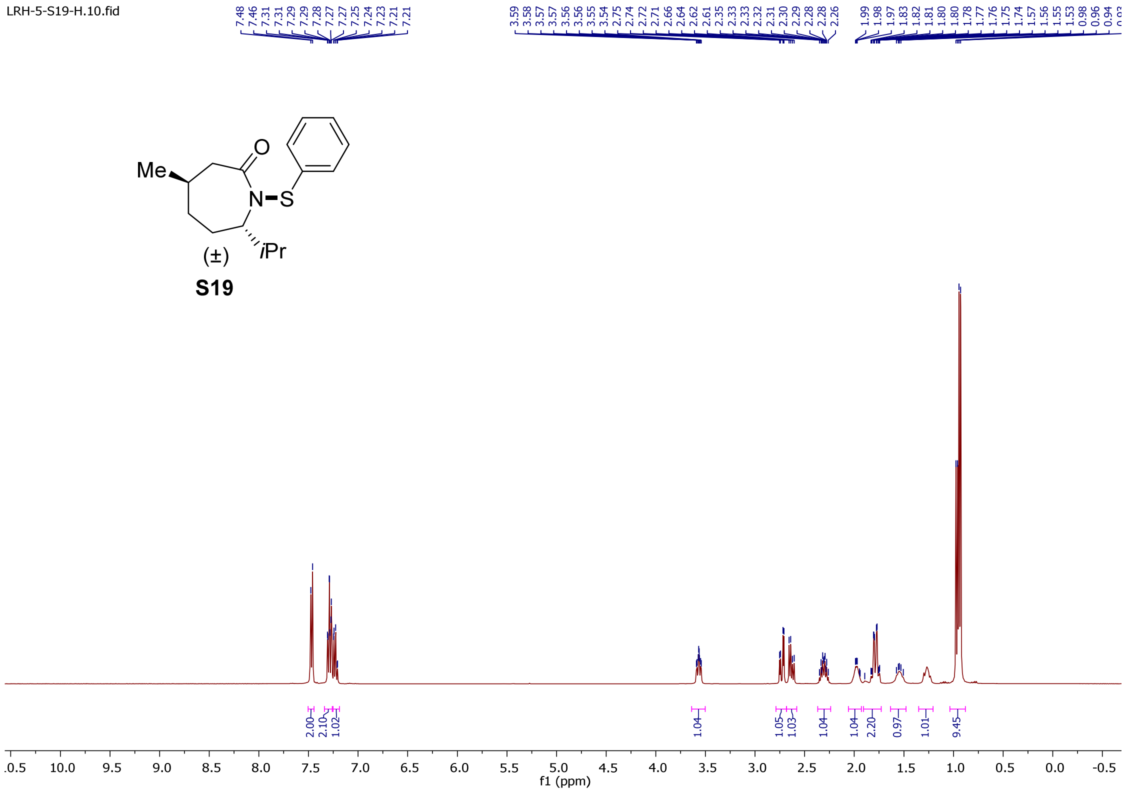

Supplementary Figure 37. ^{1}H NMR Spectrum of **S19**

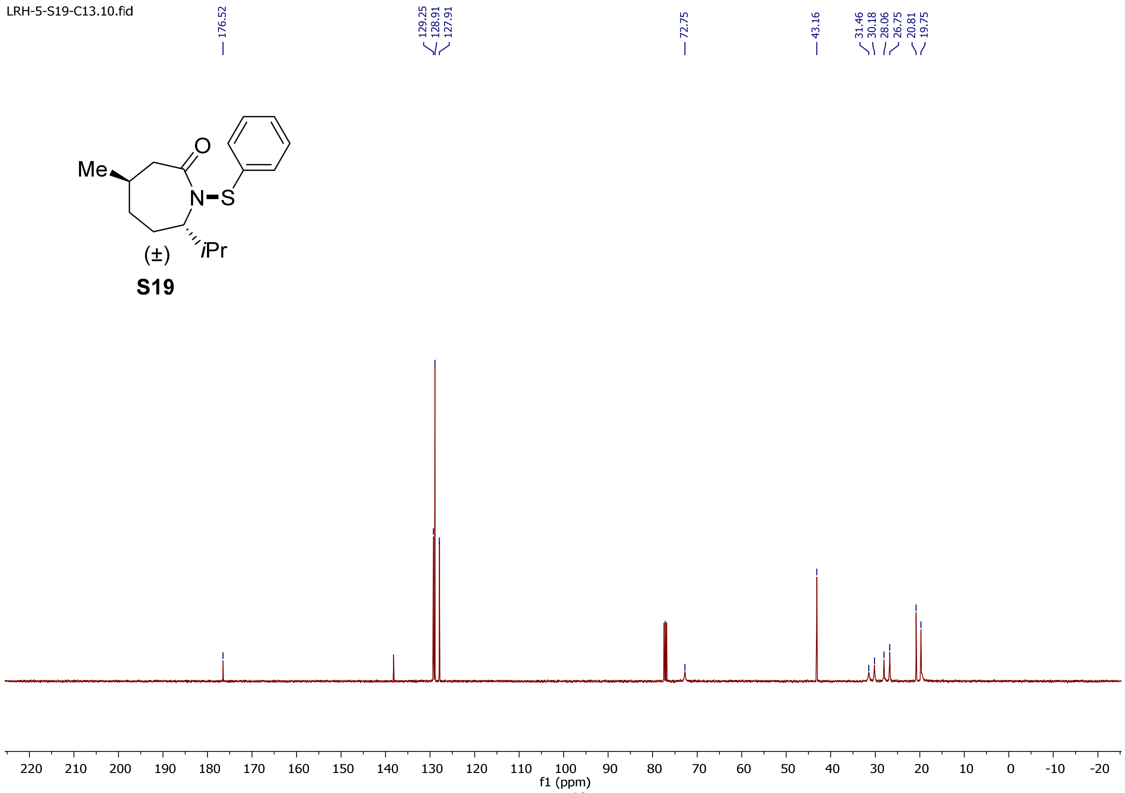

Supplementary Figure 38. ^{13}C NMR Spectrum of **S19**

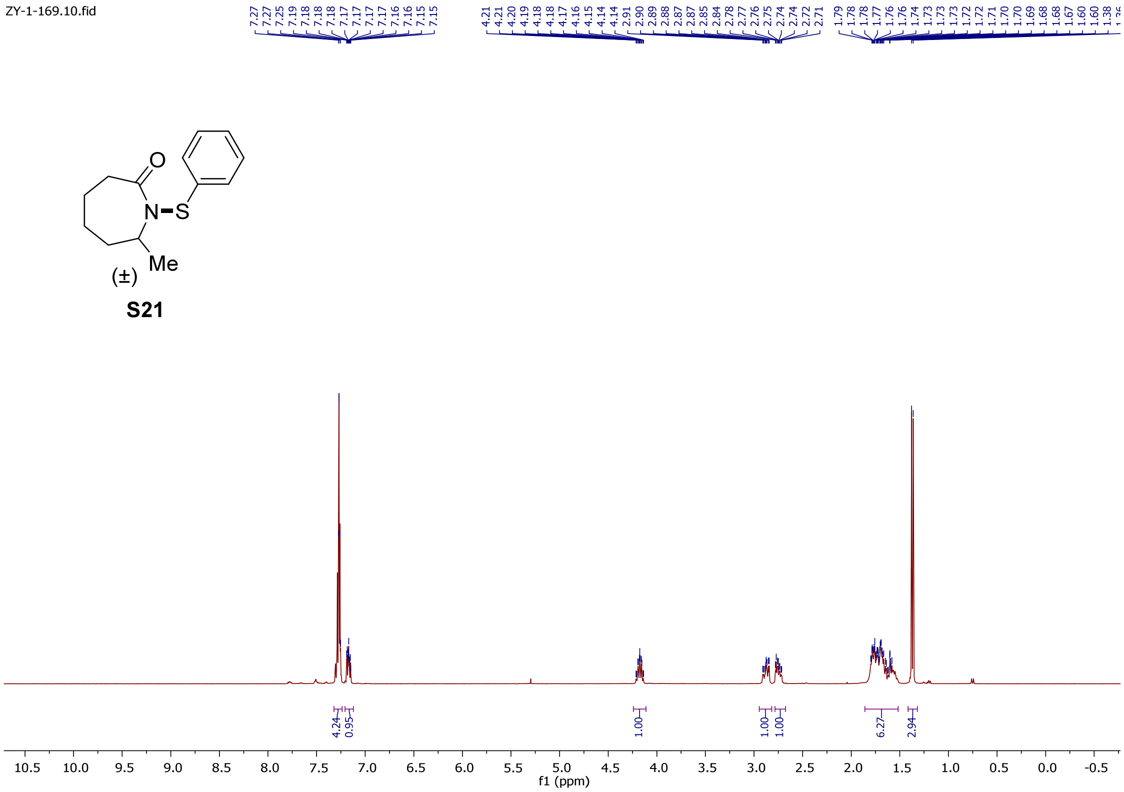

Supplementary Figure 39. ^{1}H NMR Spectrum of **S21**

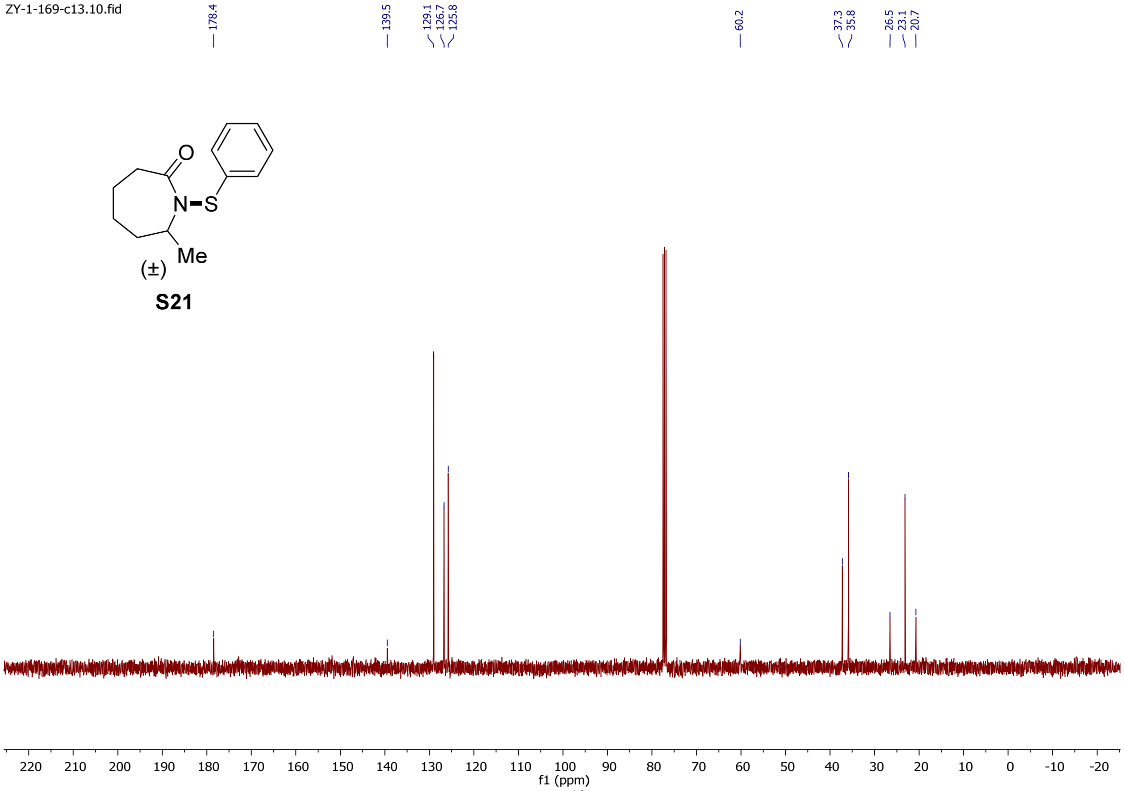

Supplementary Figure 40. ^{13}C NMR Spectrum of **S21**

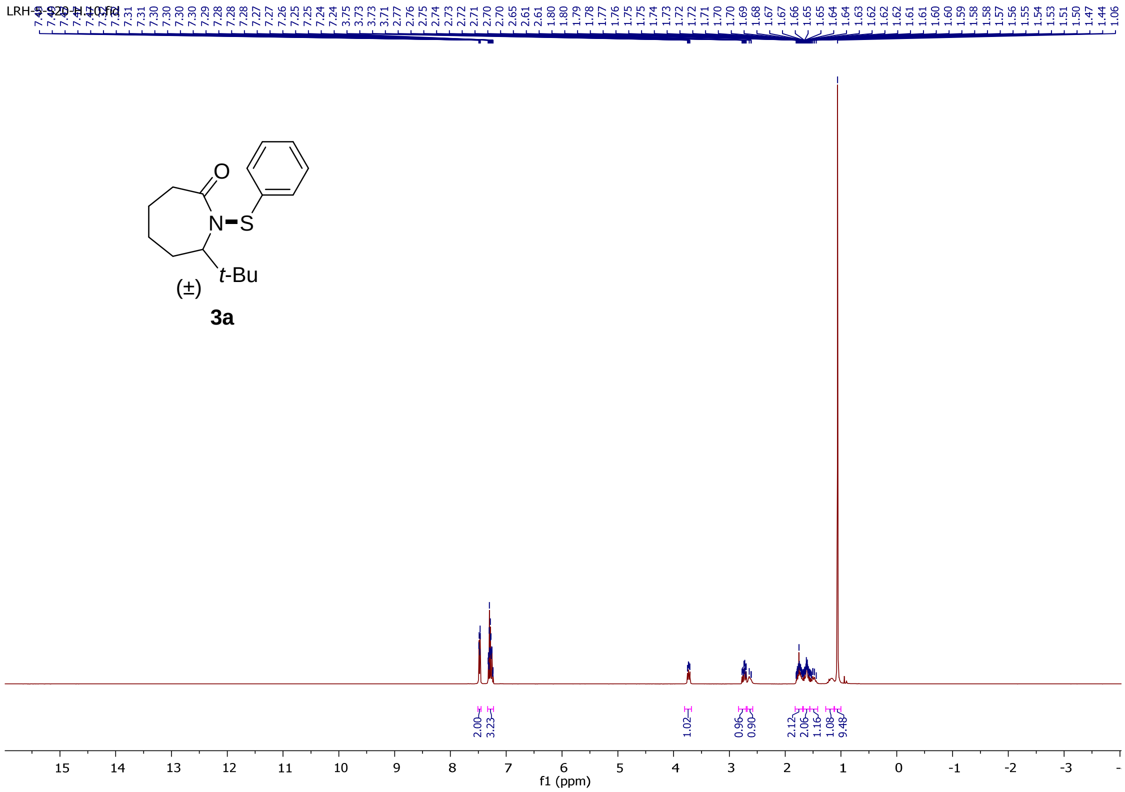

Supplementary Figure 41. ^{1}H NMR Spectrum of **3a**

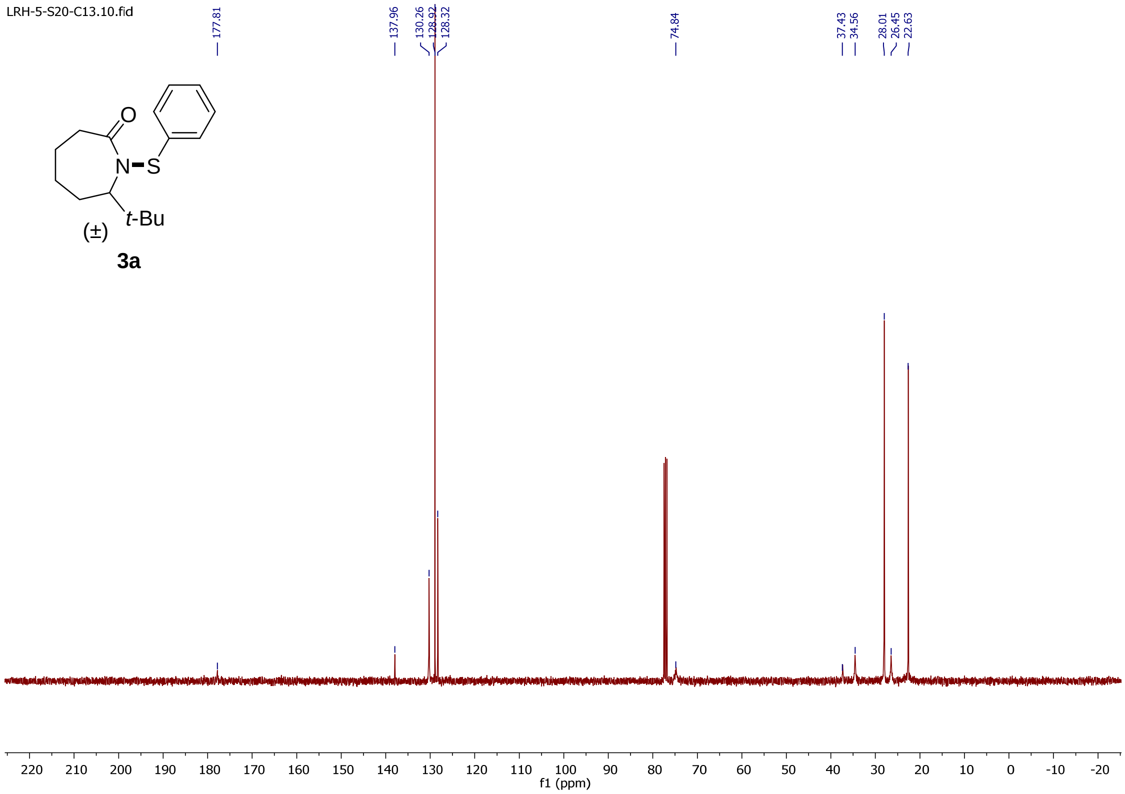

Supplementary Figure 42. ^{13}C NMR Spectrum of **3a**

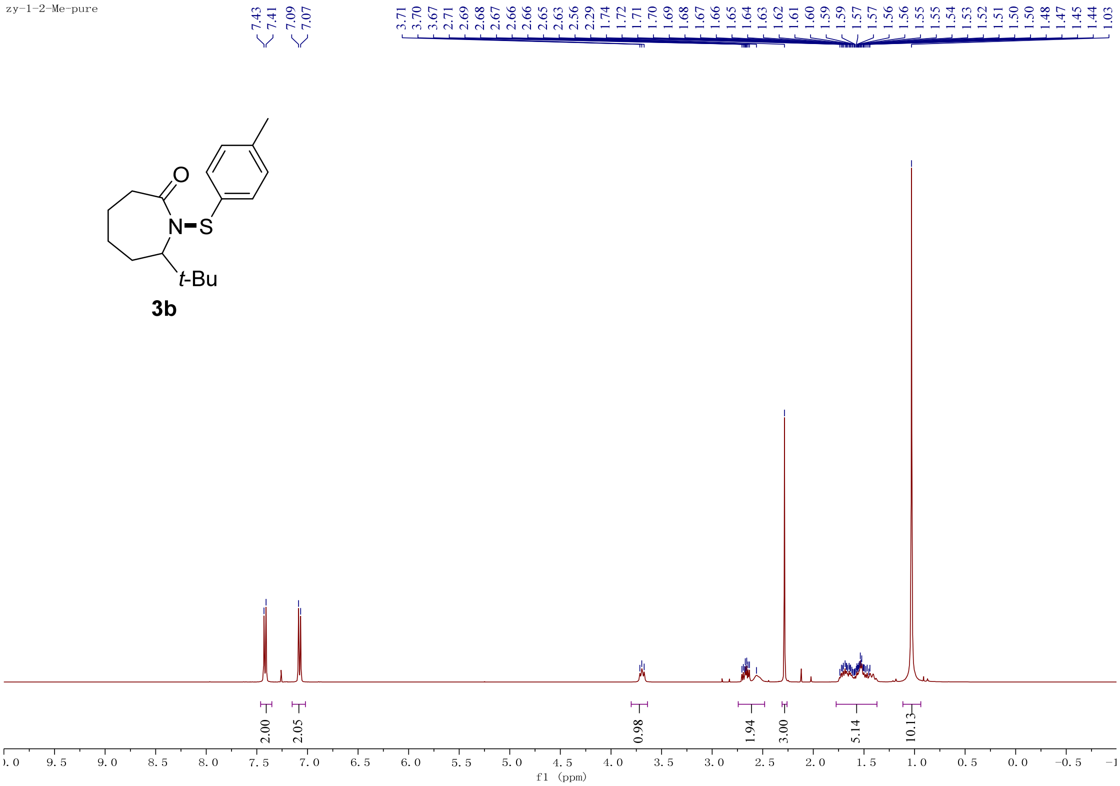

Supplementary Figure 43. ^{1}H NMR Spectrum of **3b**

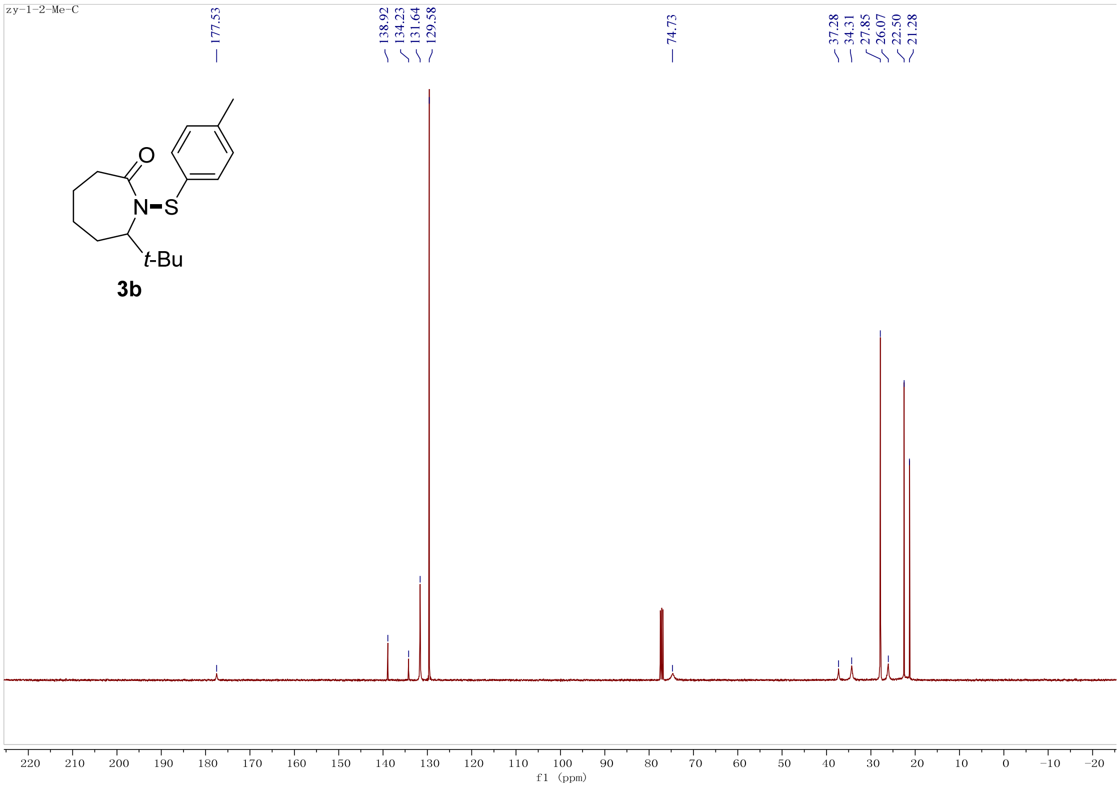

Supplementary Figure 44. ^{13}C NMR Spectrum of **3b**

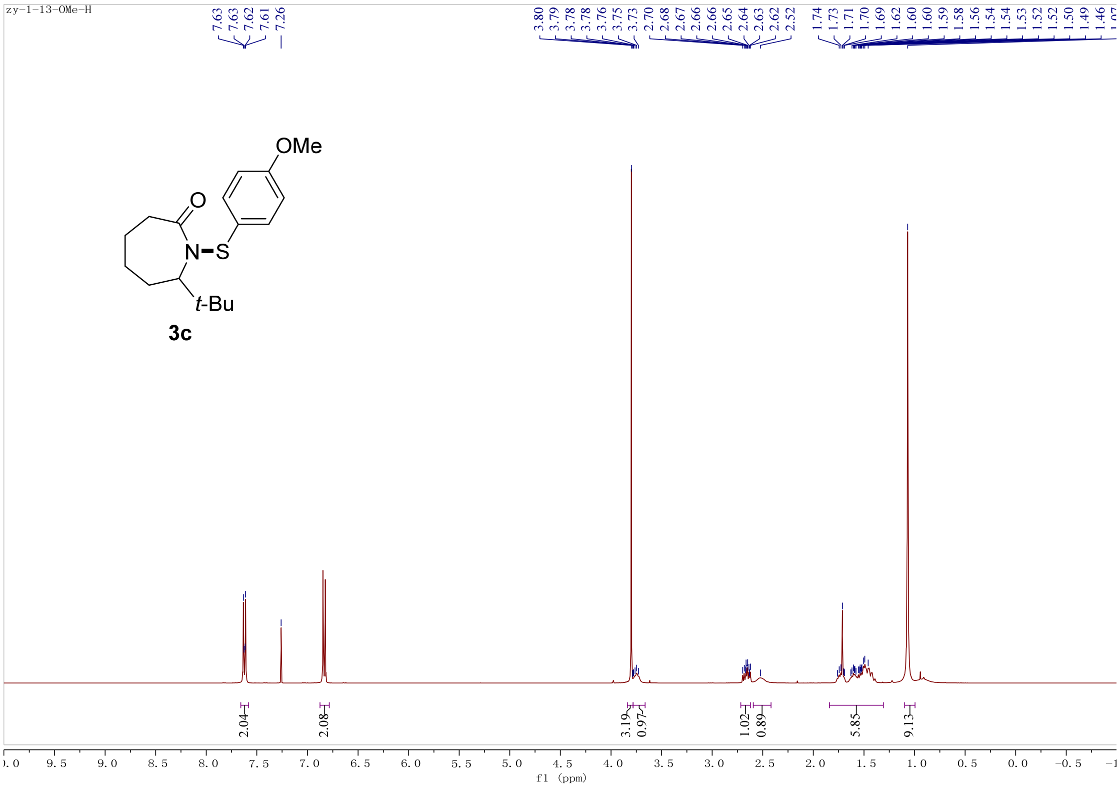

Supplementary Figure 45. ^{1}H NMR Spectrum of **3c**

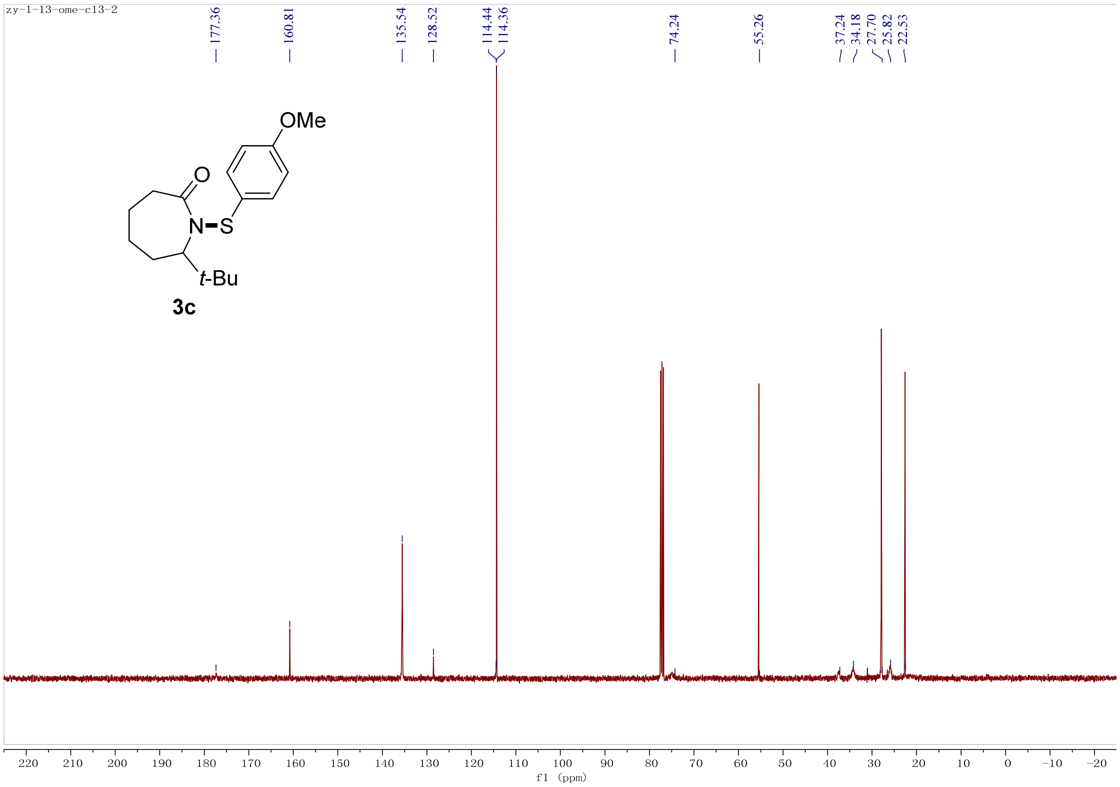

Supplementary Figure 46. ^{13}C NMR Spectrum of **3c**

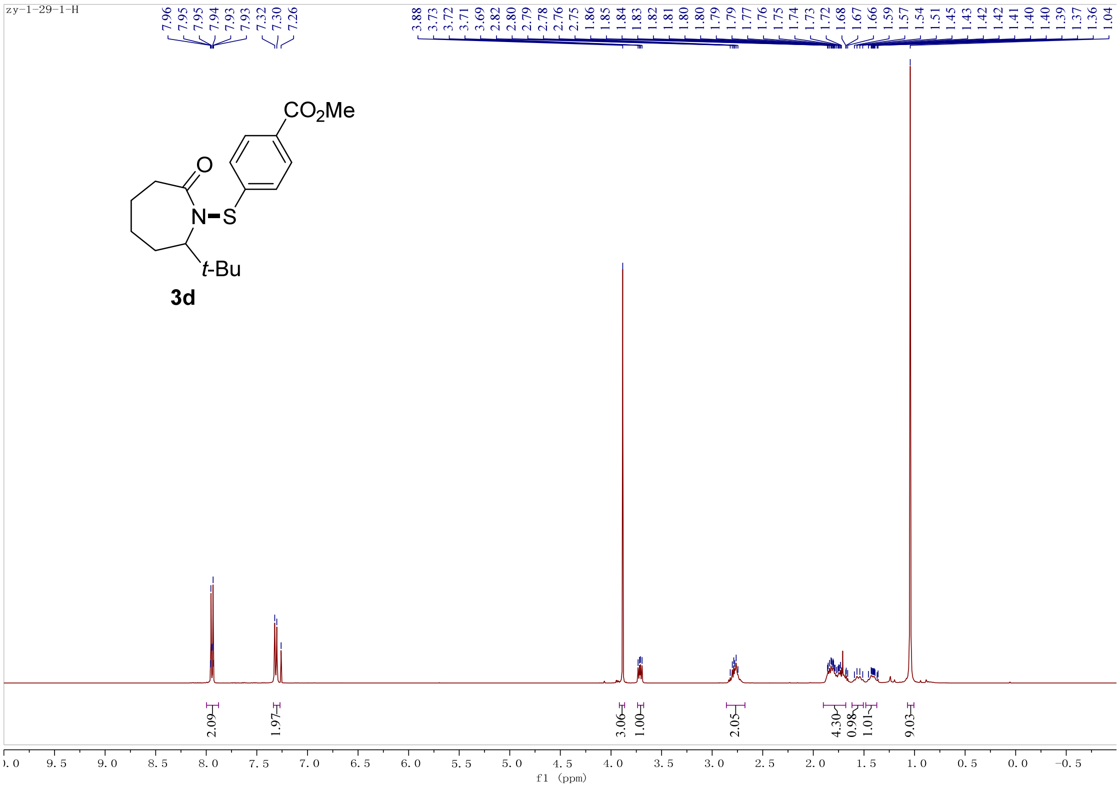

Supplementary Figure 47. ^{1}H NMR Spectrum of **3d**

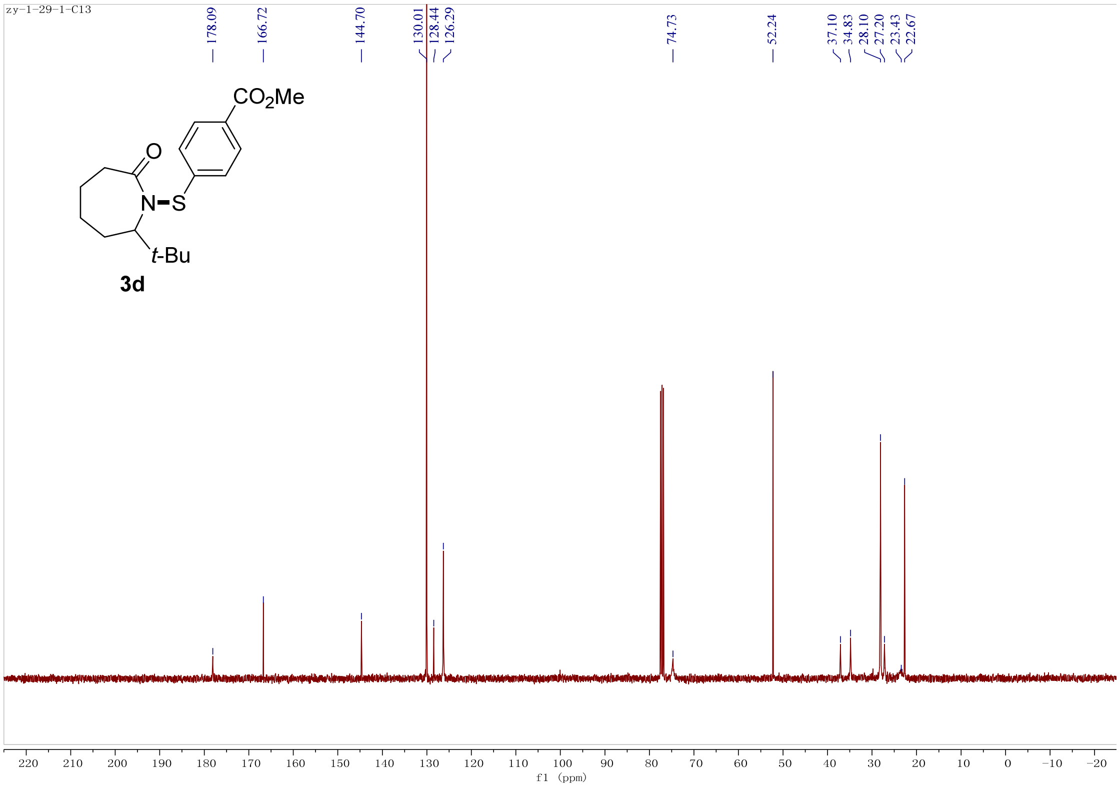

Supplementary Figure 48. ^{13}C NMR Spectrum of **3d**

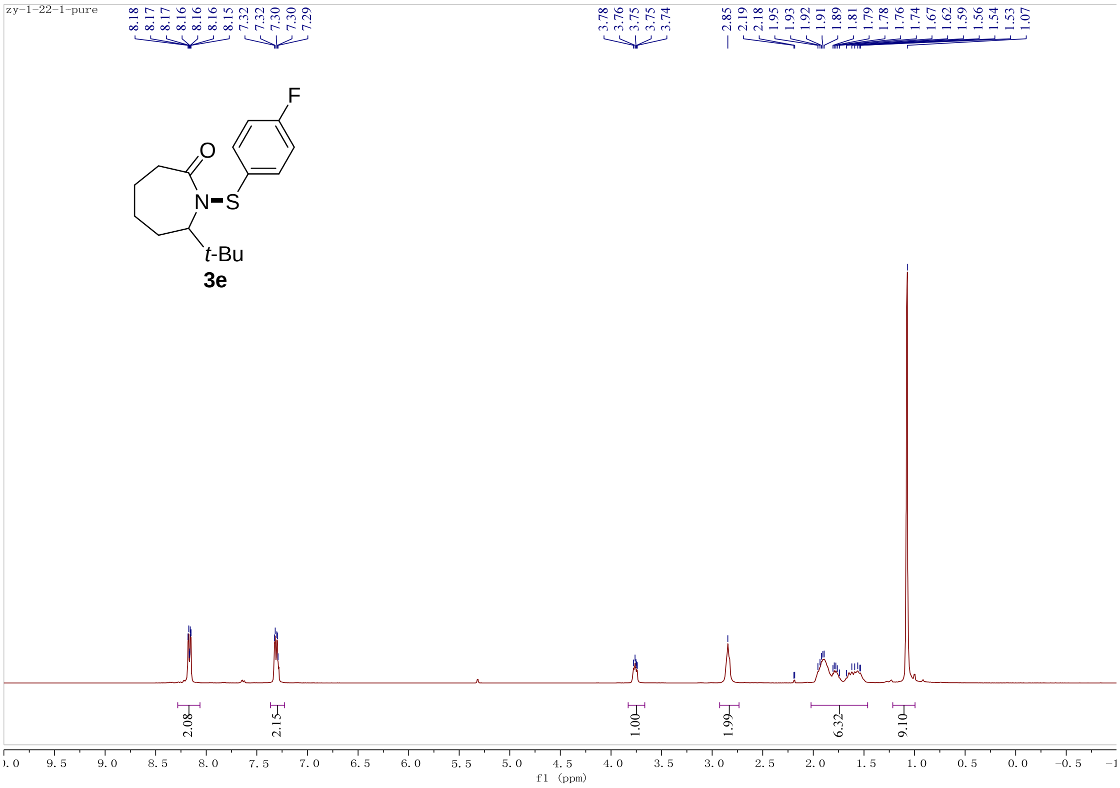

Supplementary Figure 49. ^{1}H NMR Spectrum of **3e**

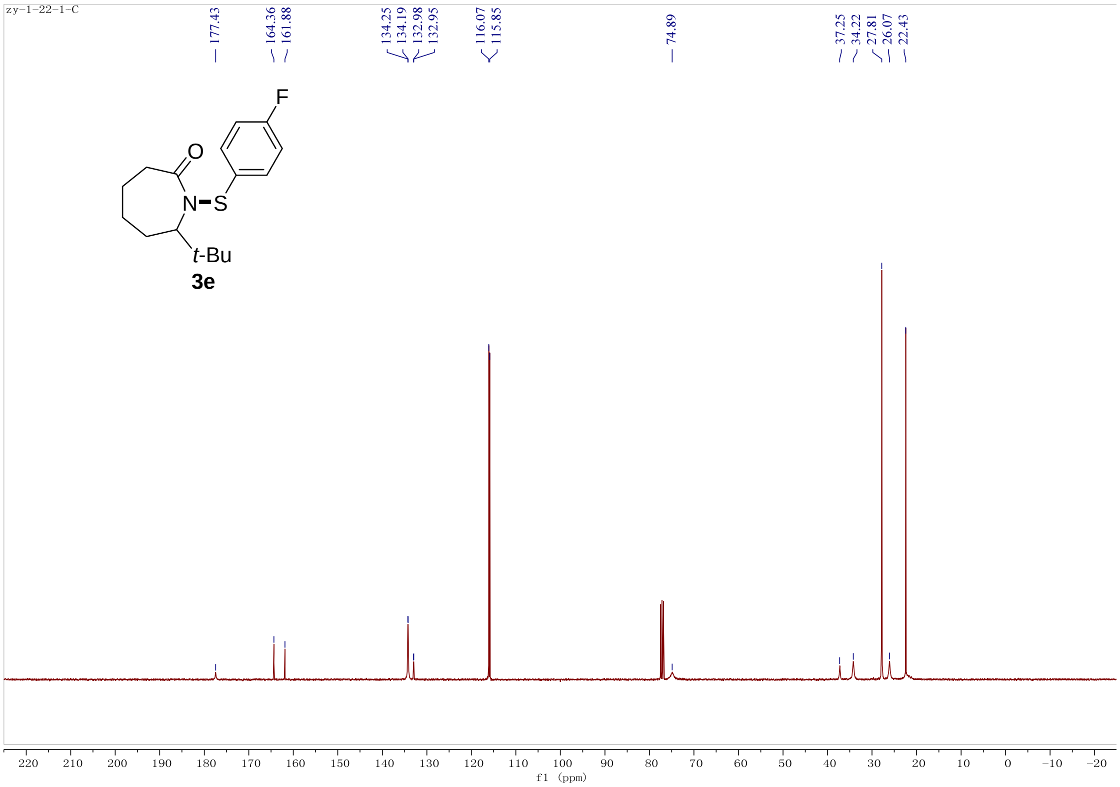

Supplementary Figure 50. ^{13}C NMR Spectrum of **3e**

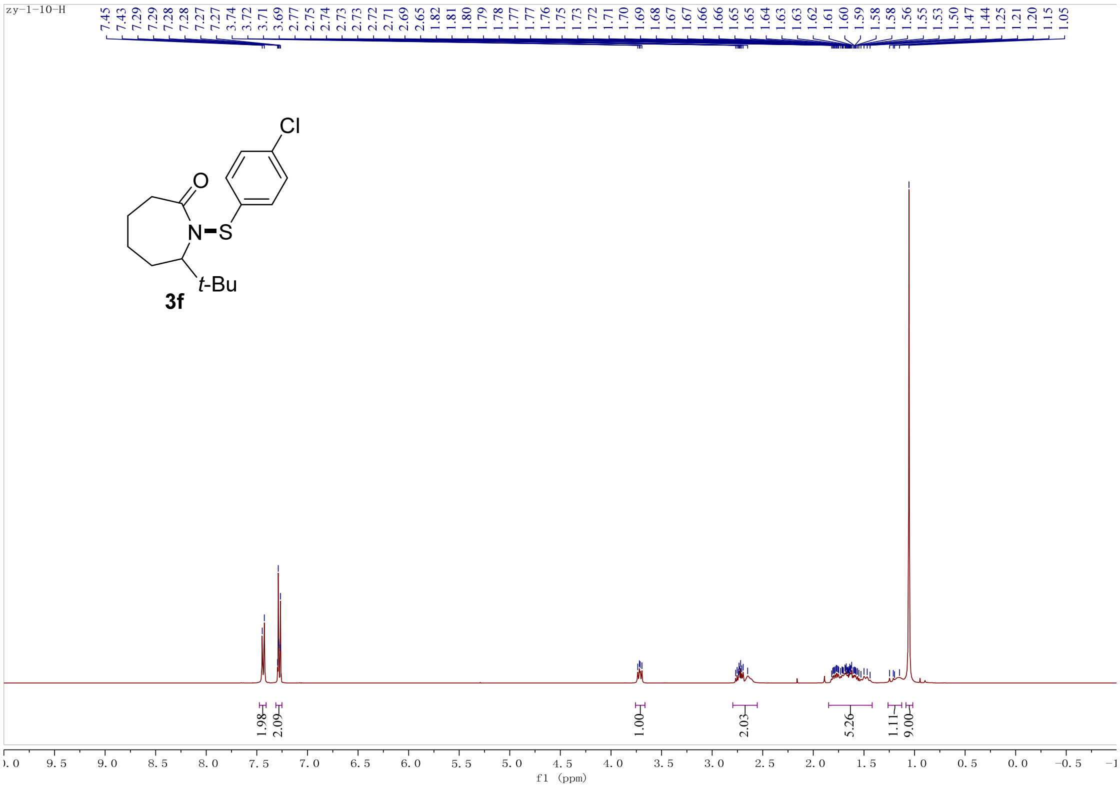

Supplementary Figure 51. ^{1}H NMR Spectrum of **3f**

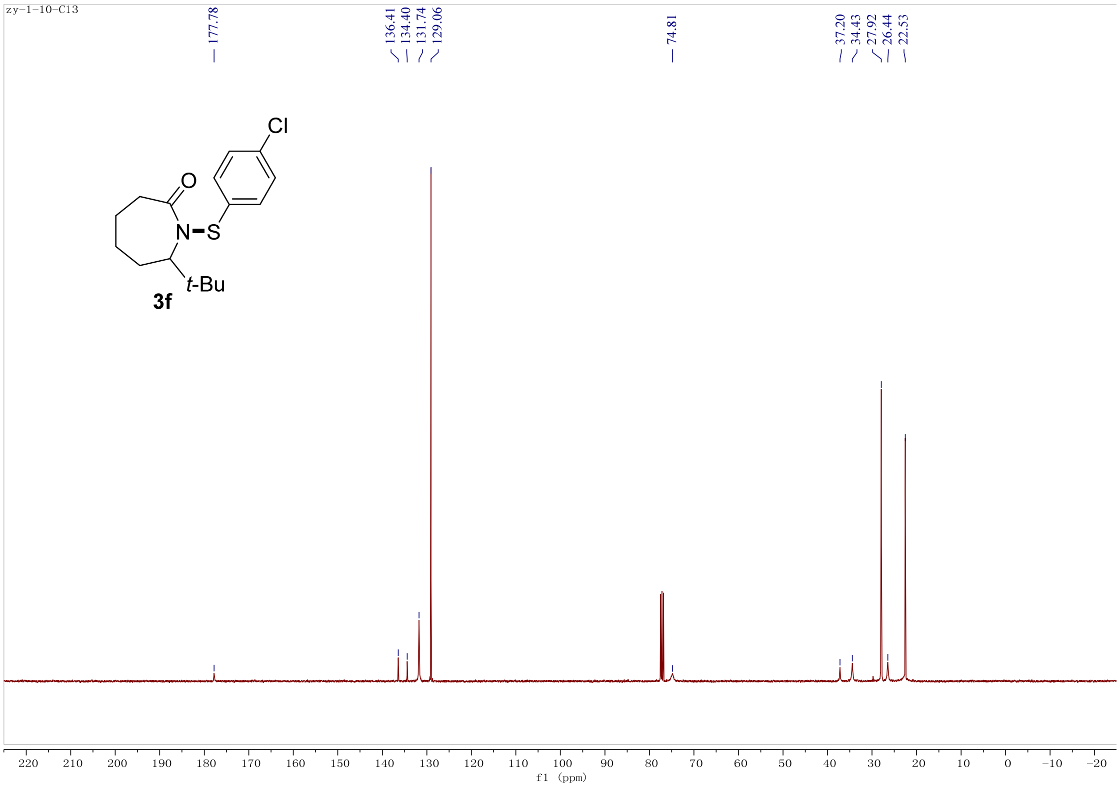

Supplementary Figure 52. ^{13}C NMR Spectrum of **3f**

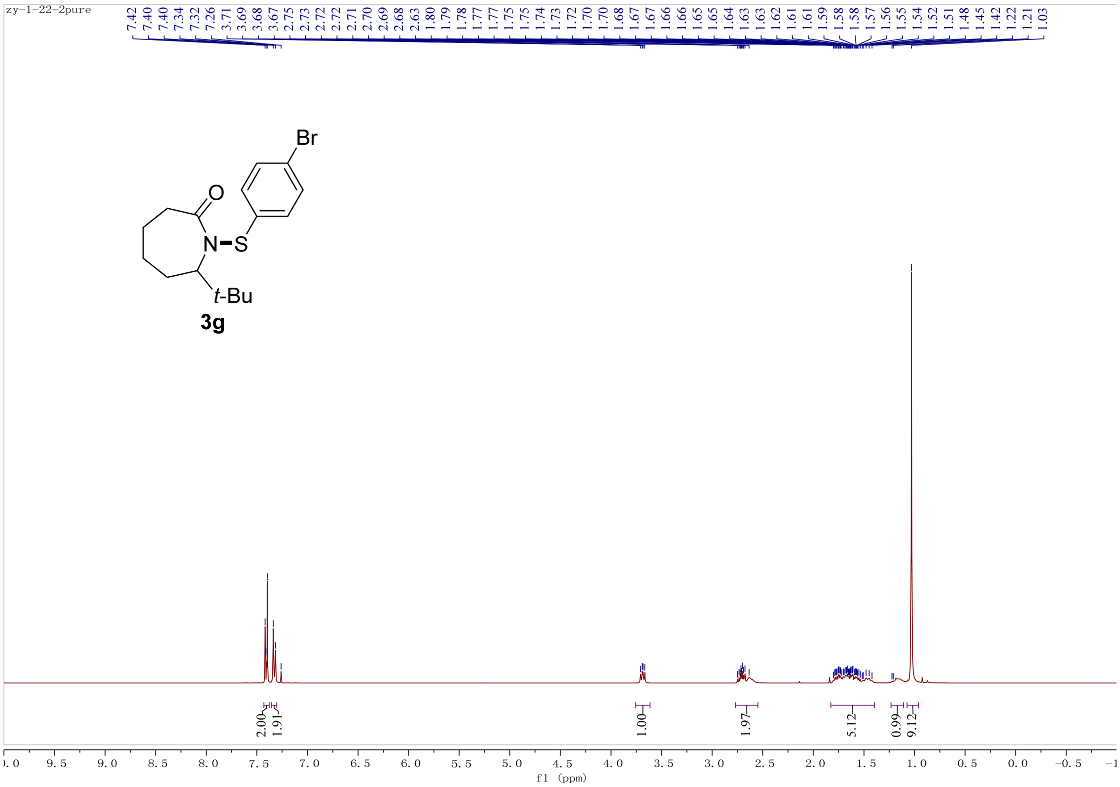

Supplementary Figure 53. ^{1}H NMR Spectrum of **3g**

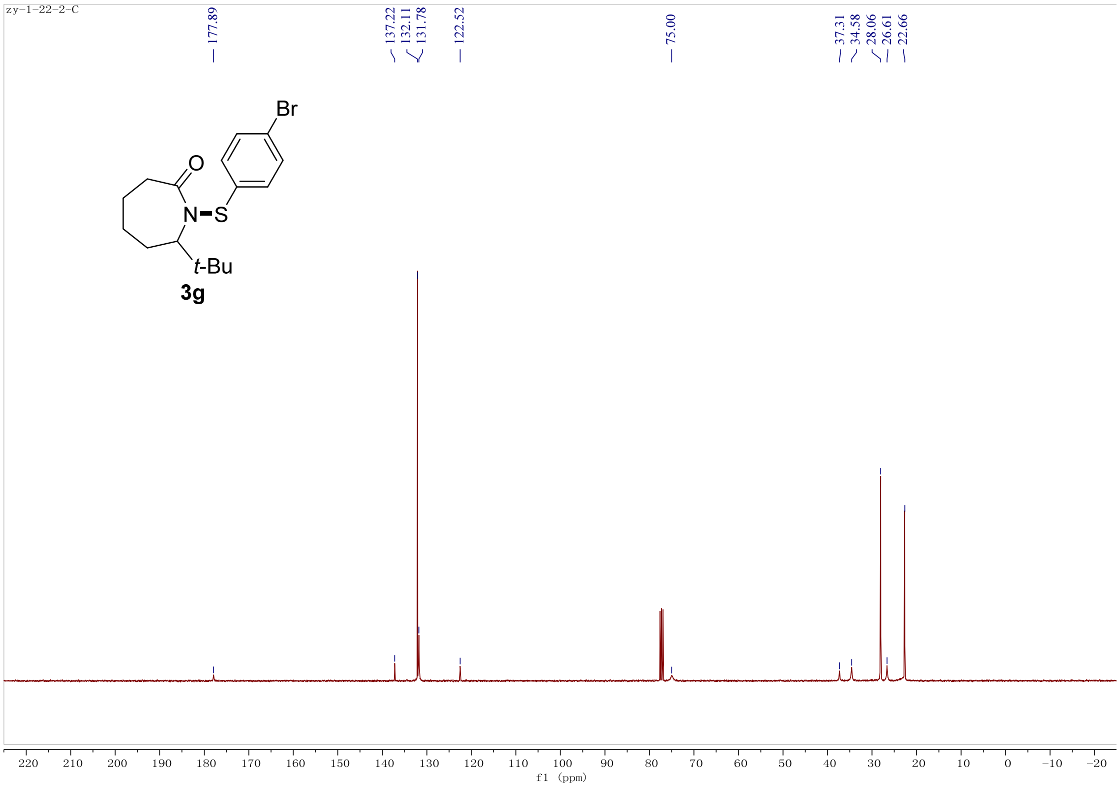

Supplementary Figure 54. ^{13}C NMR Spectrum of **3g**

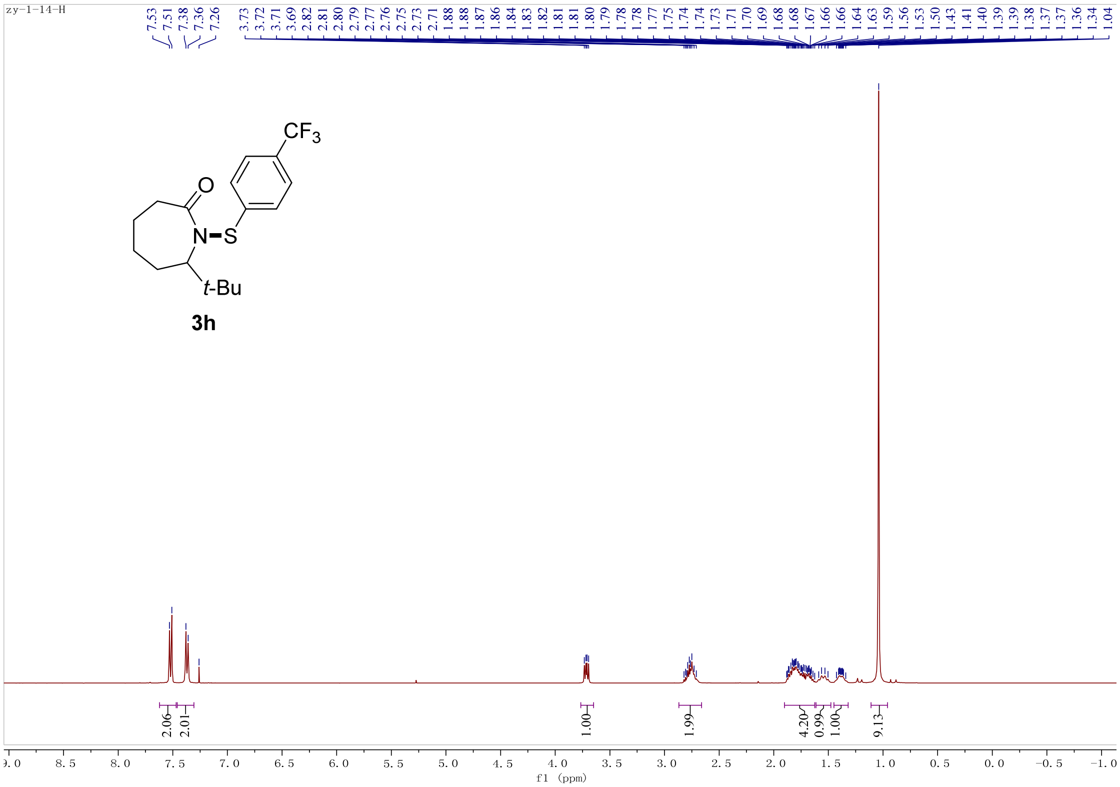

Supplementary Figure 55. ^{1}H NMR Spectrum of **3h**

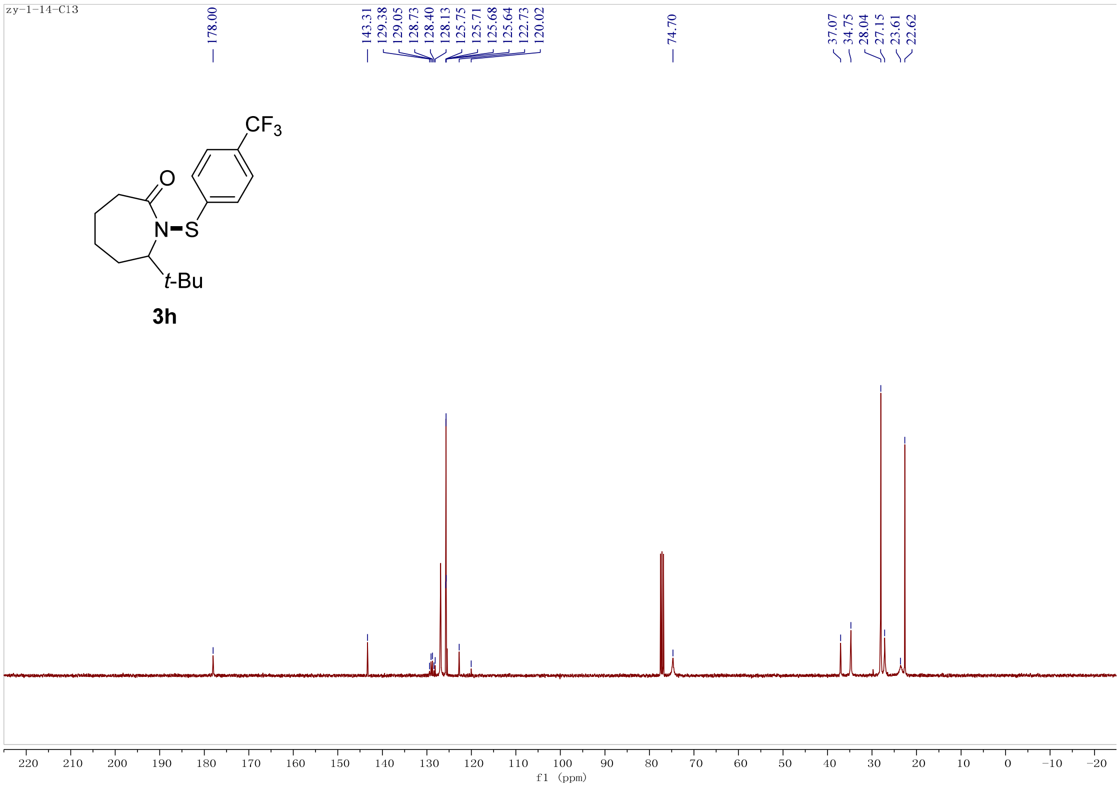

Supplementary Figure 56. ^{13}C NMR Spectrum of **3h**

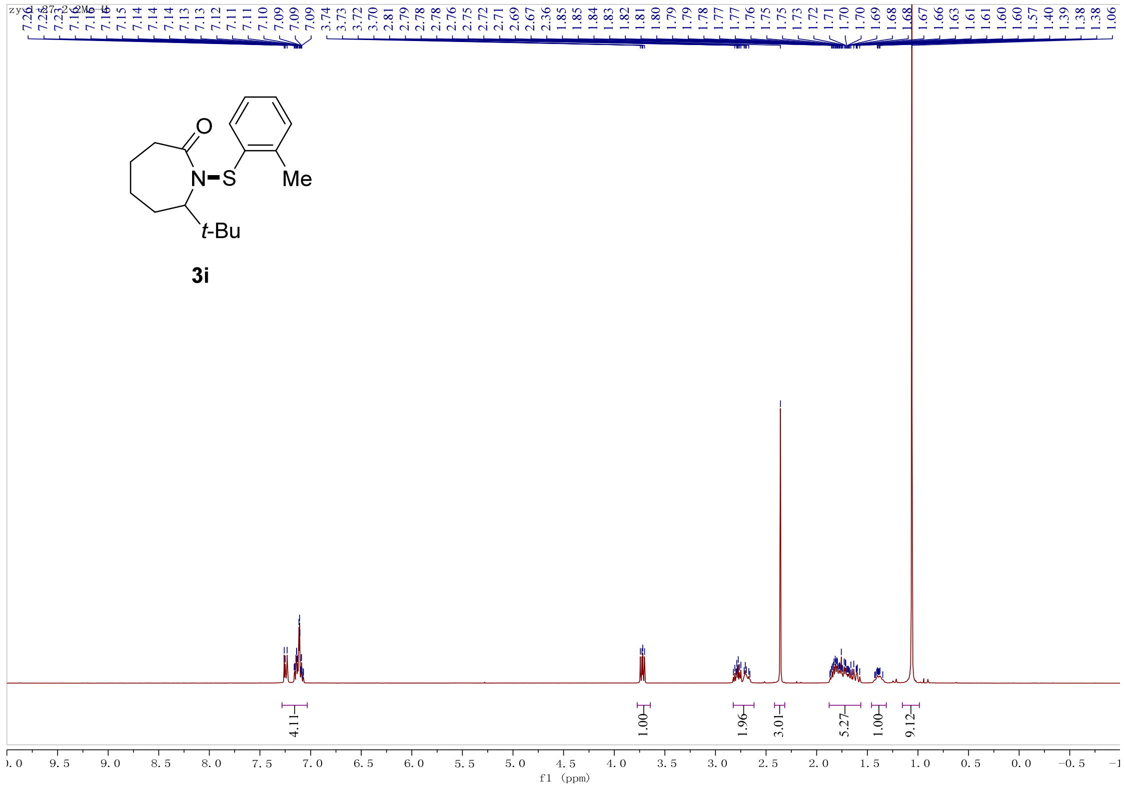

Supplementary Figure 57. ^{1}H NMR Spectrum of **3i**

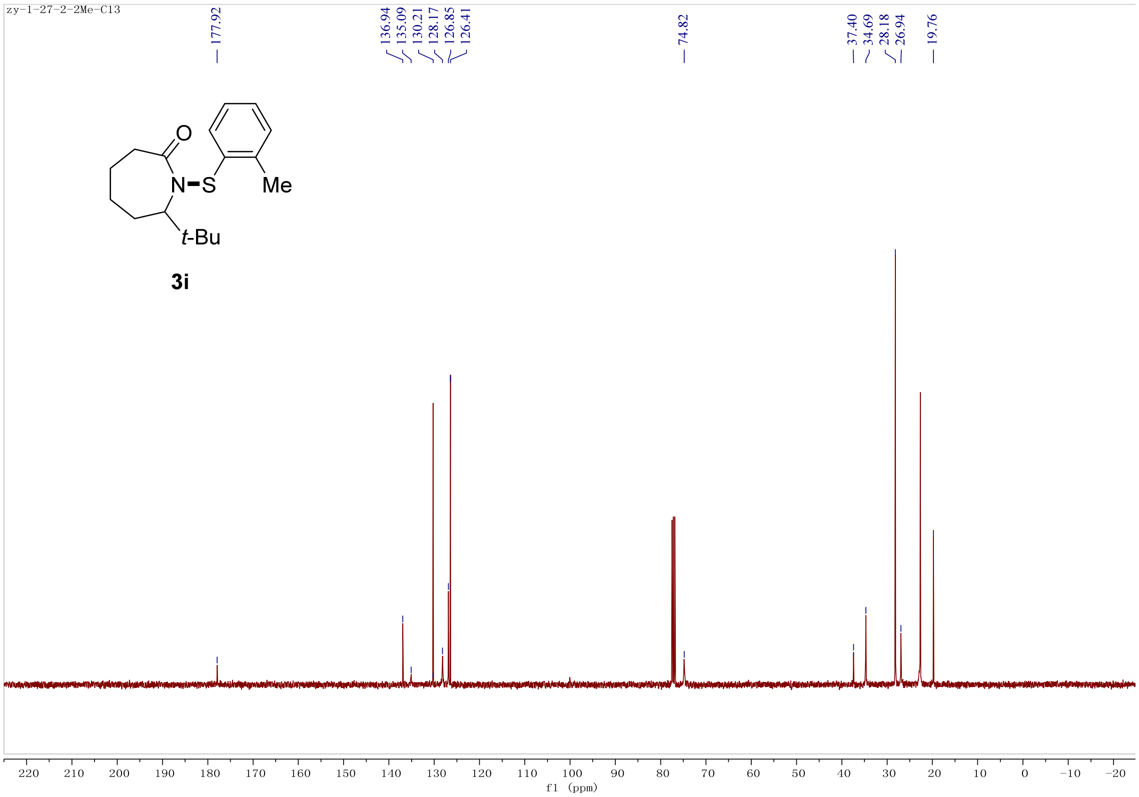

Supplementary Figure 58. ^{13}C NMR Spectrum of **3i**

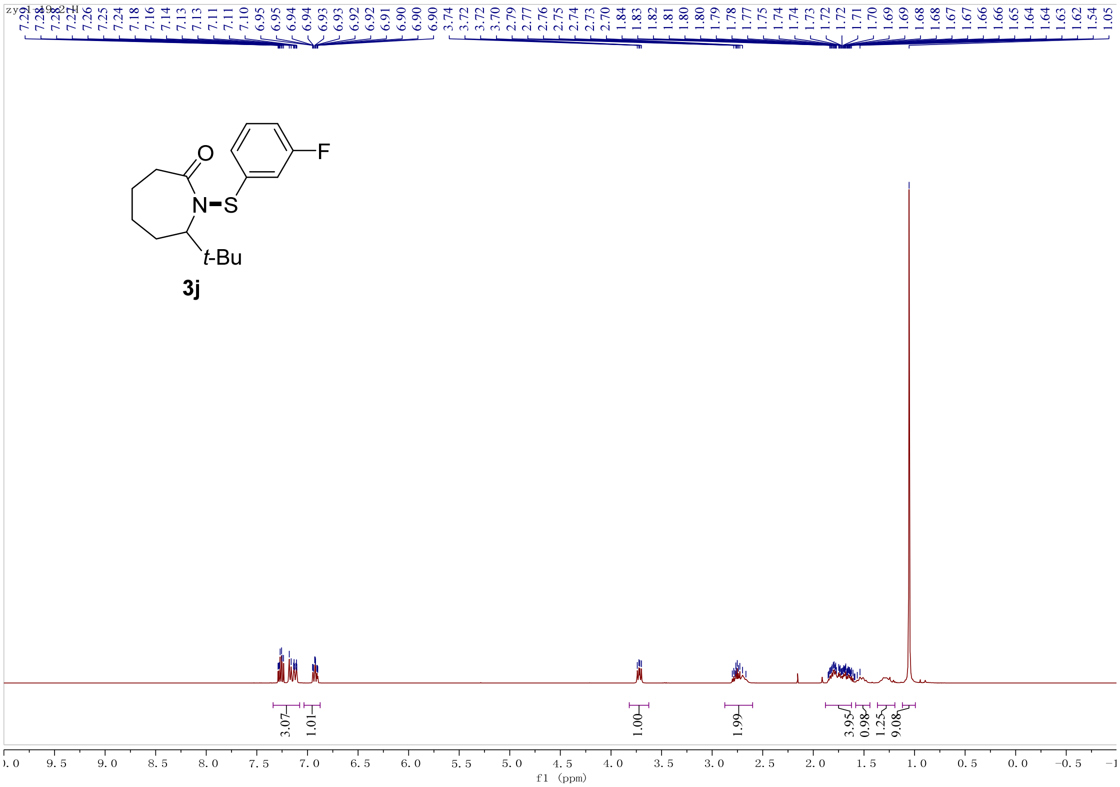

Supplementary Figure 59. ^{1}H NMR Spectrum of **3j**

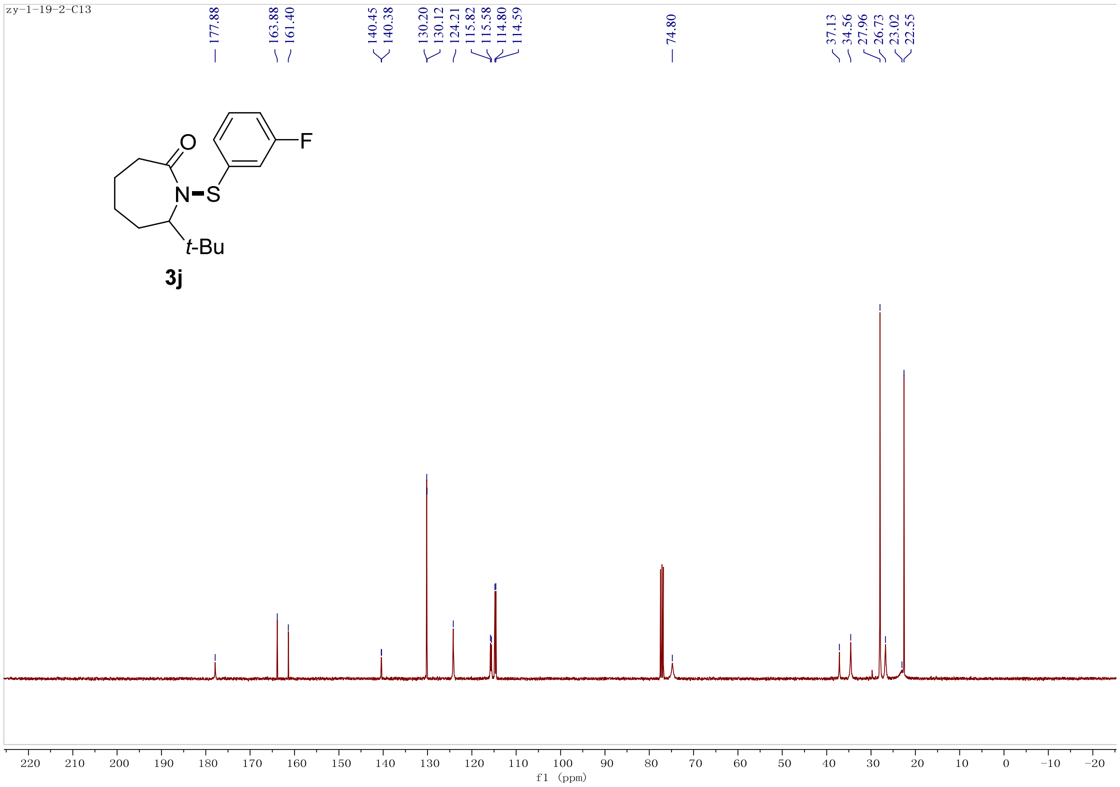

Supplementary Figure 60. ^{13}C NMR Spectrum of **3j**

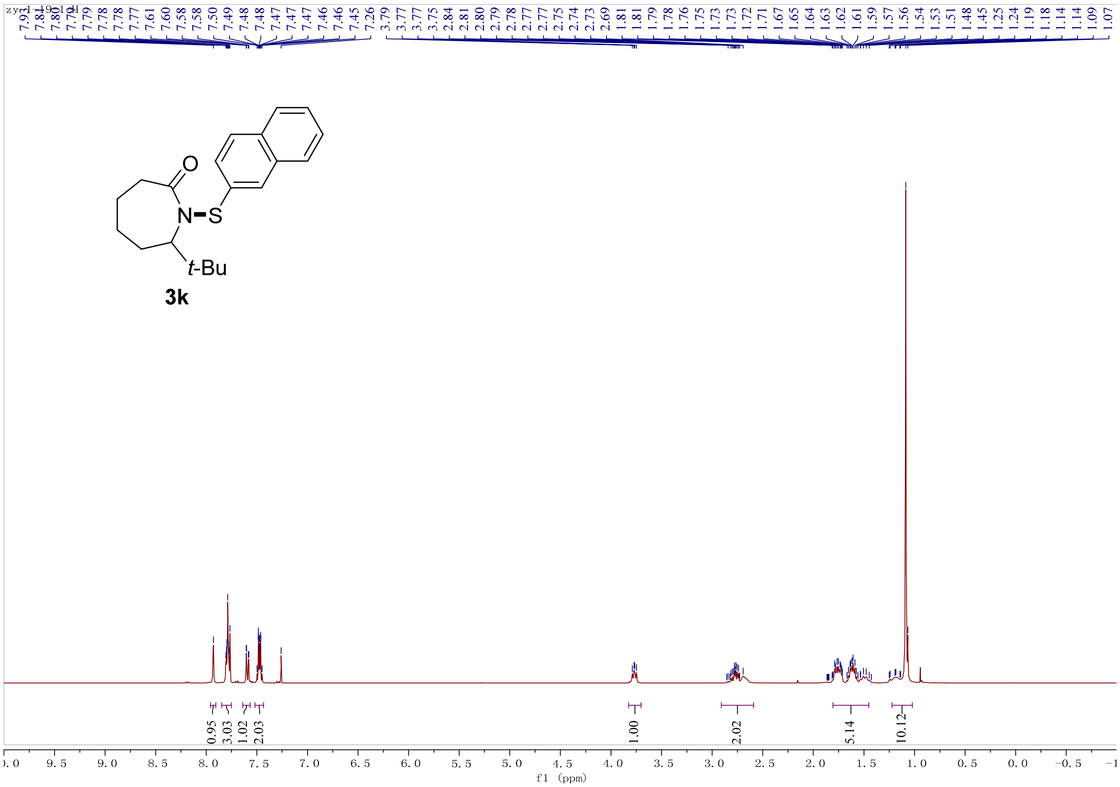

Supplementary Figure 61. ^{1}H NMR Spectrum of **3k**

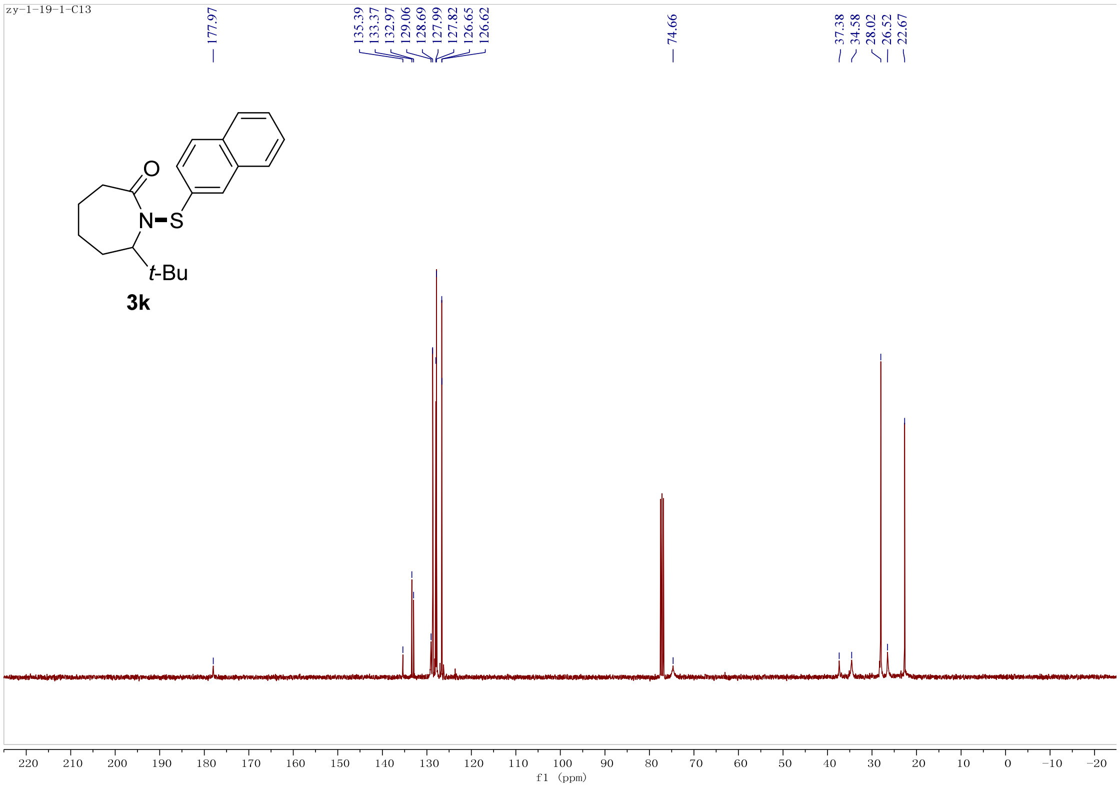

Supplementary Figure 62. ^{13}C NMR Spectrum of **3k**

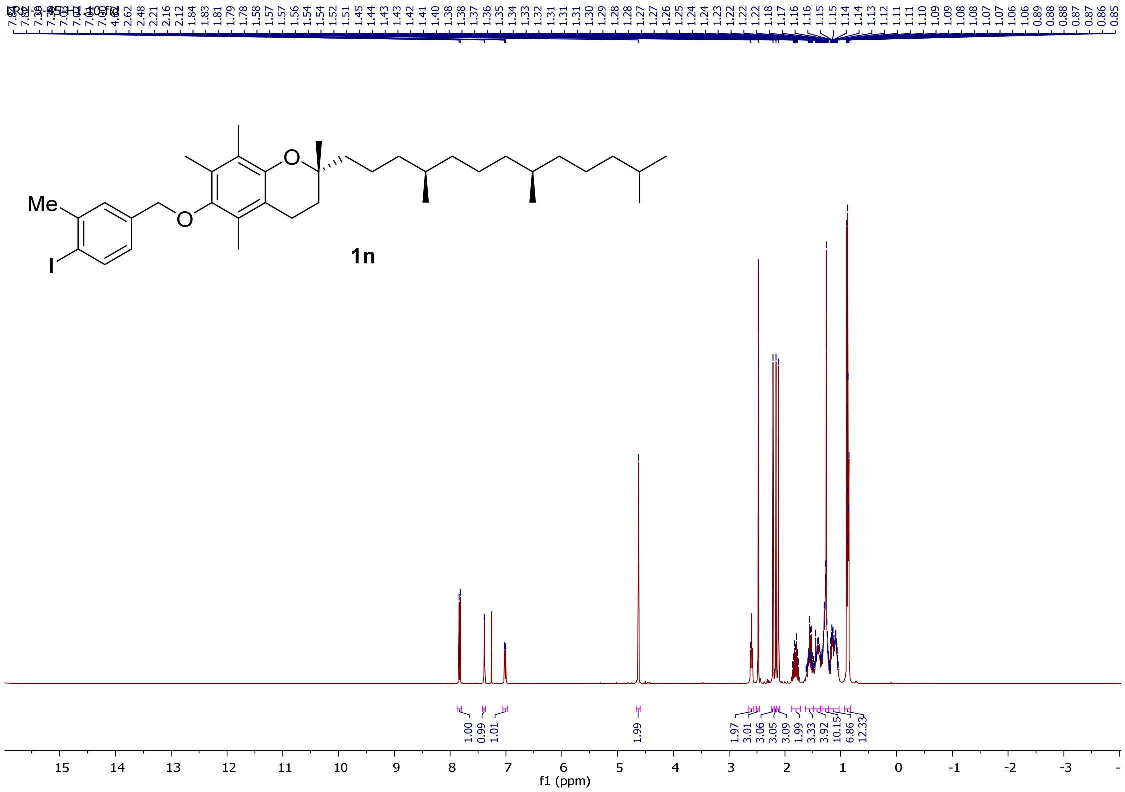

Supplementary Figure 63. ^{1}H NMR Spectrum of **1n**

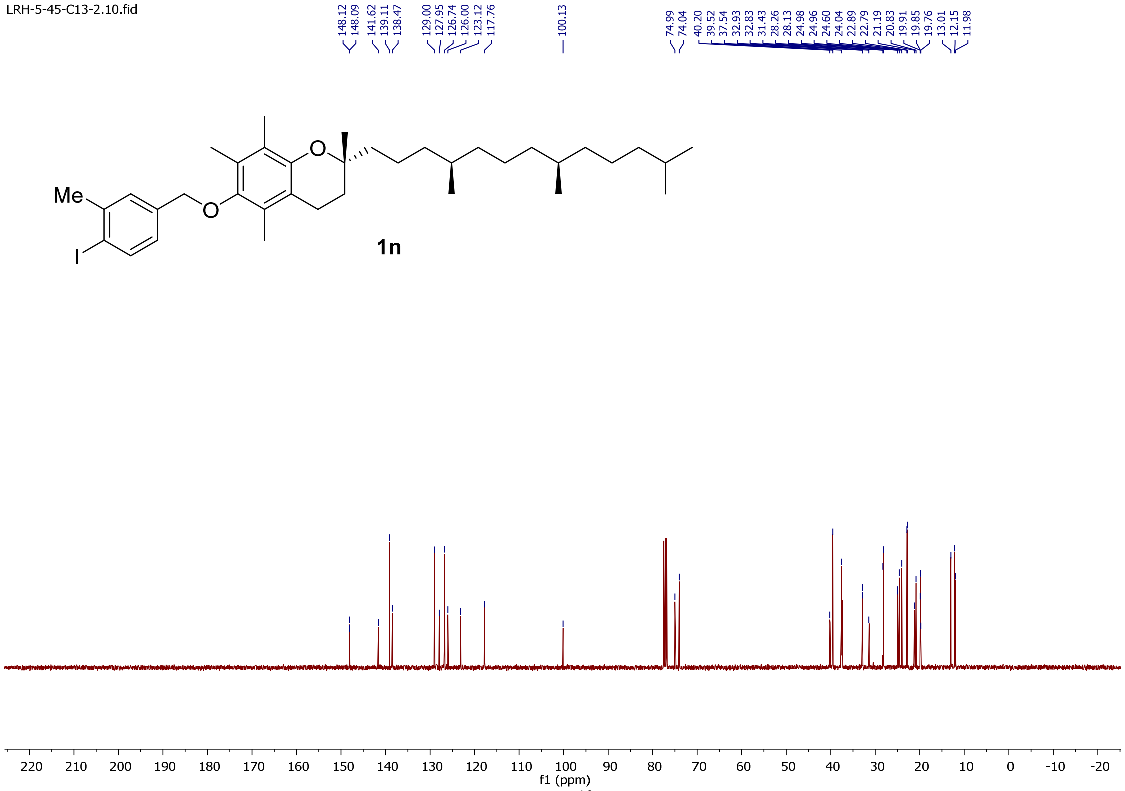

Supplementary Figure 64. ^{13}C NMR Spectrum of **1n**

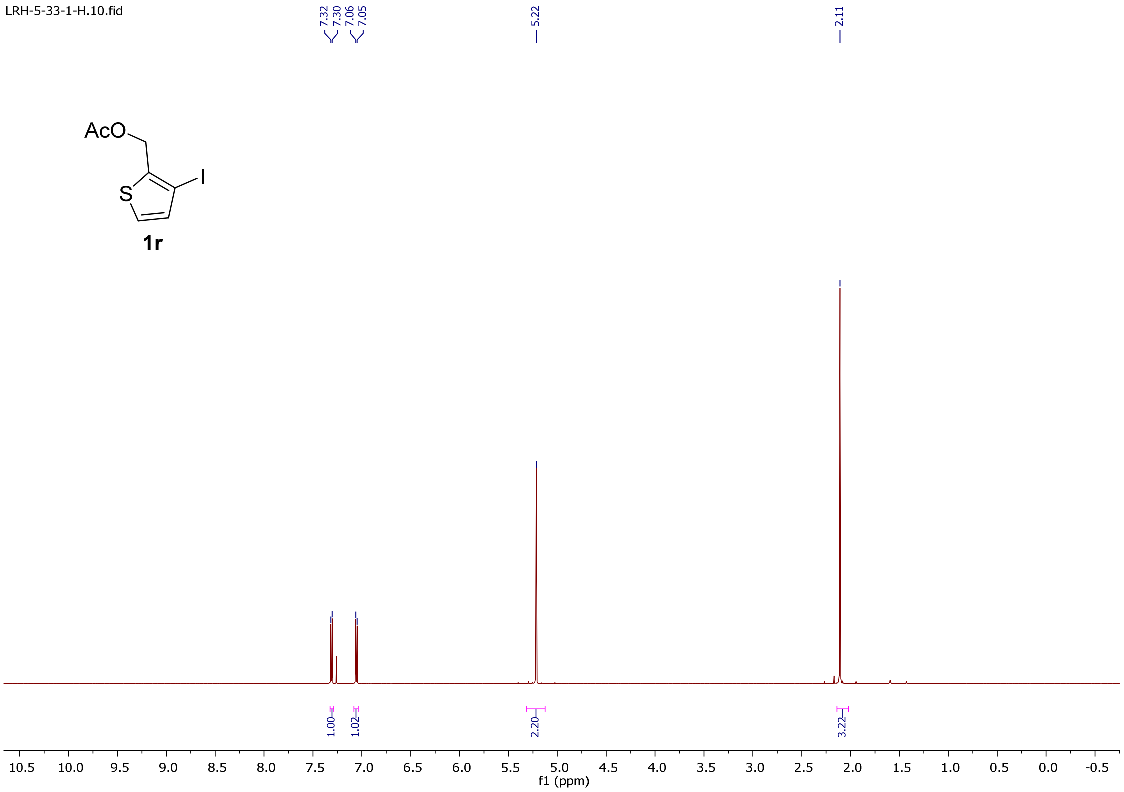

Supplementary Figure 65. ^{1}H NMR Spectrum of **1r**

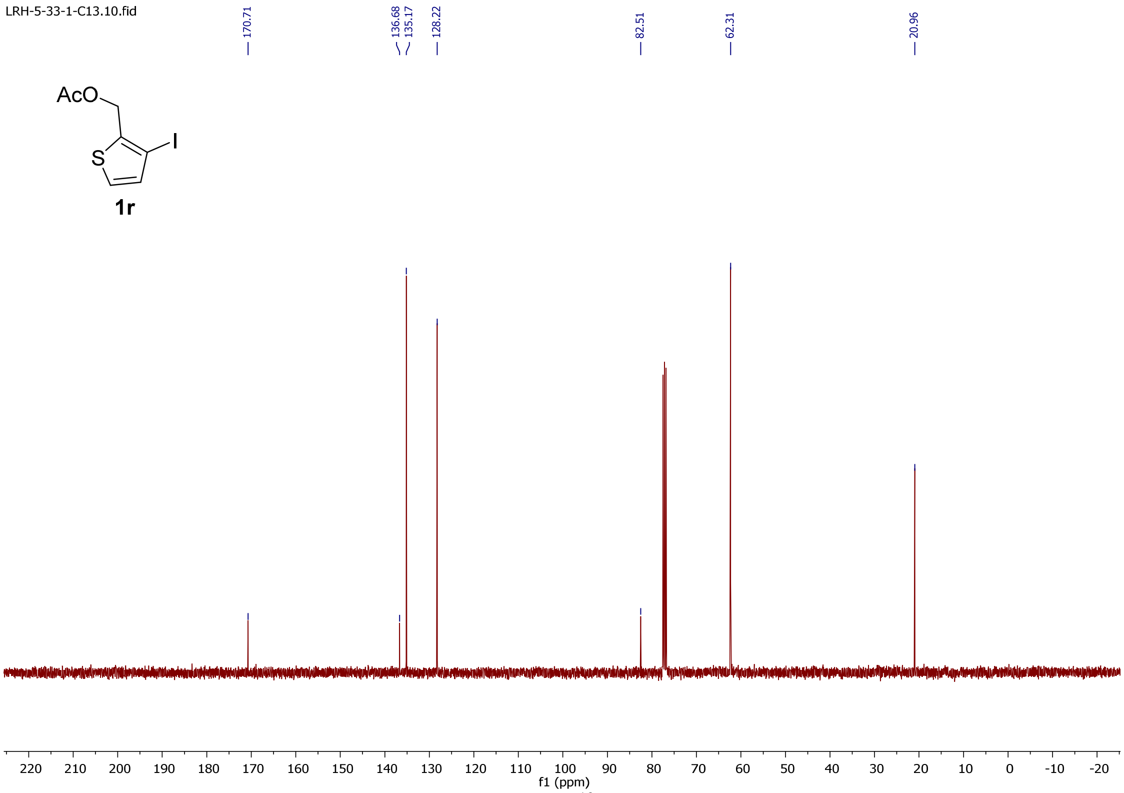

Supplementary Figure 66. ^{13}C NMR Spectrum of **1r**

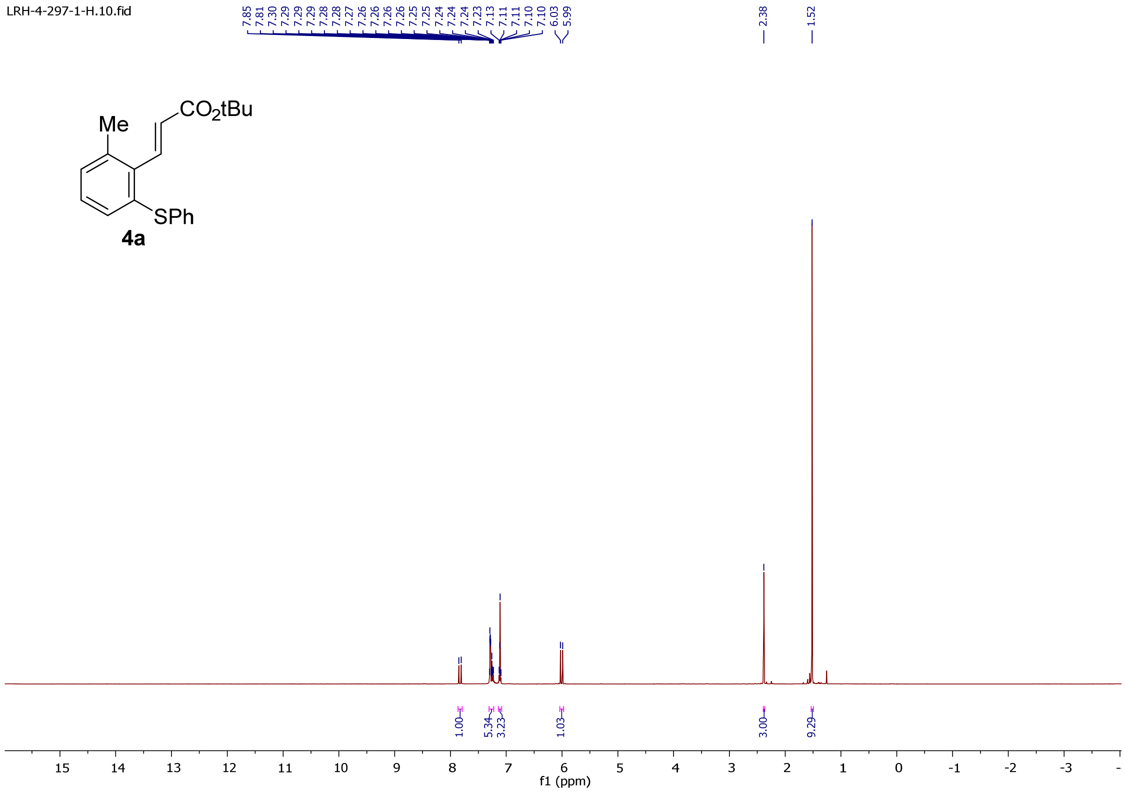

Supplementary Figure 67. ^{1}H NMR Spectrum of **4a**

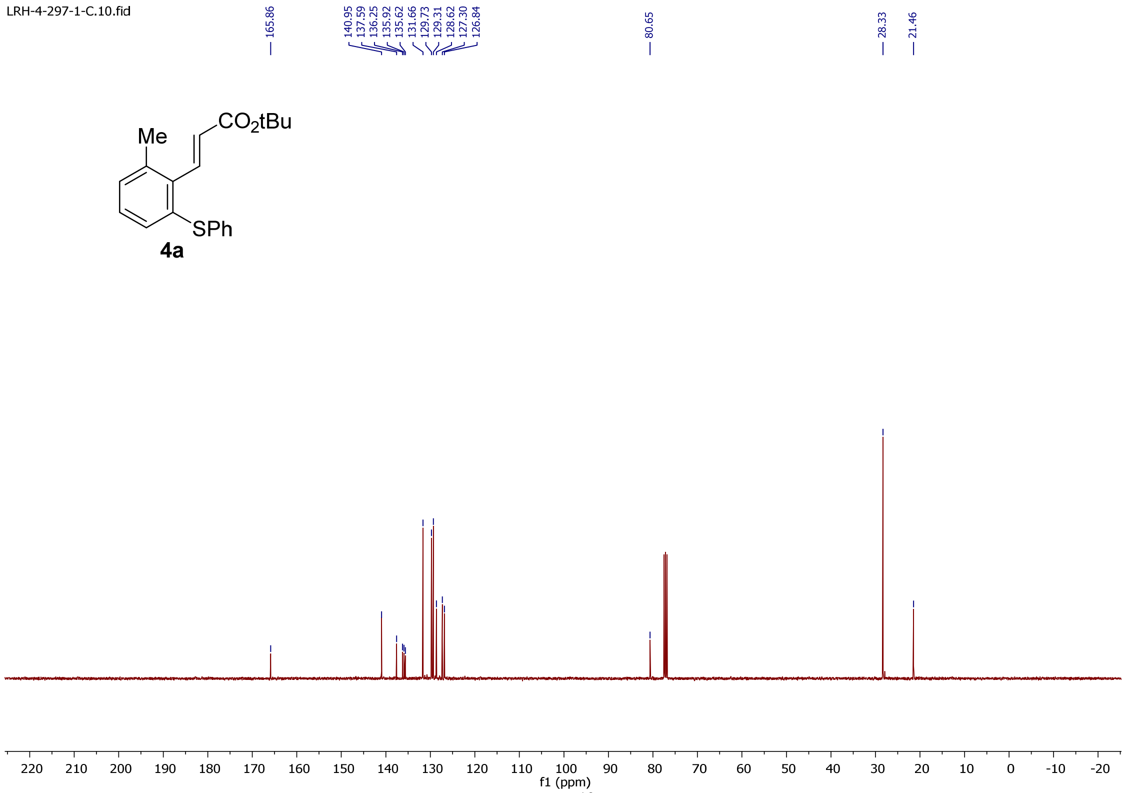

Supplementary Figure 68. ^{13}C NMR Spectrum of **4a**

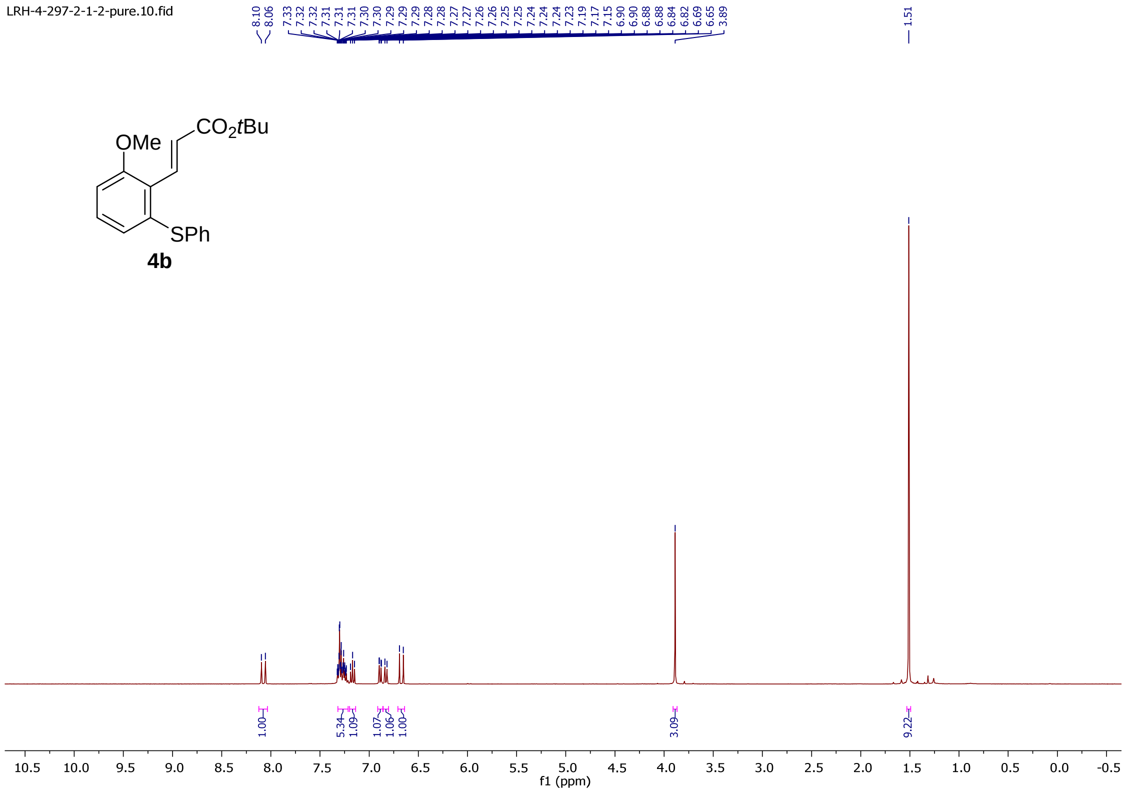

Supplementary Figure 69. ^{1}H NMR Spectrum of **4b**

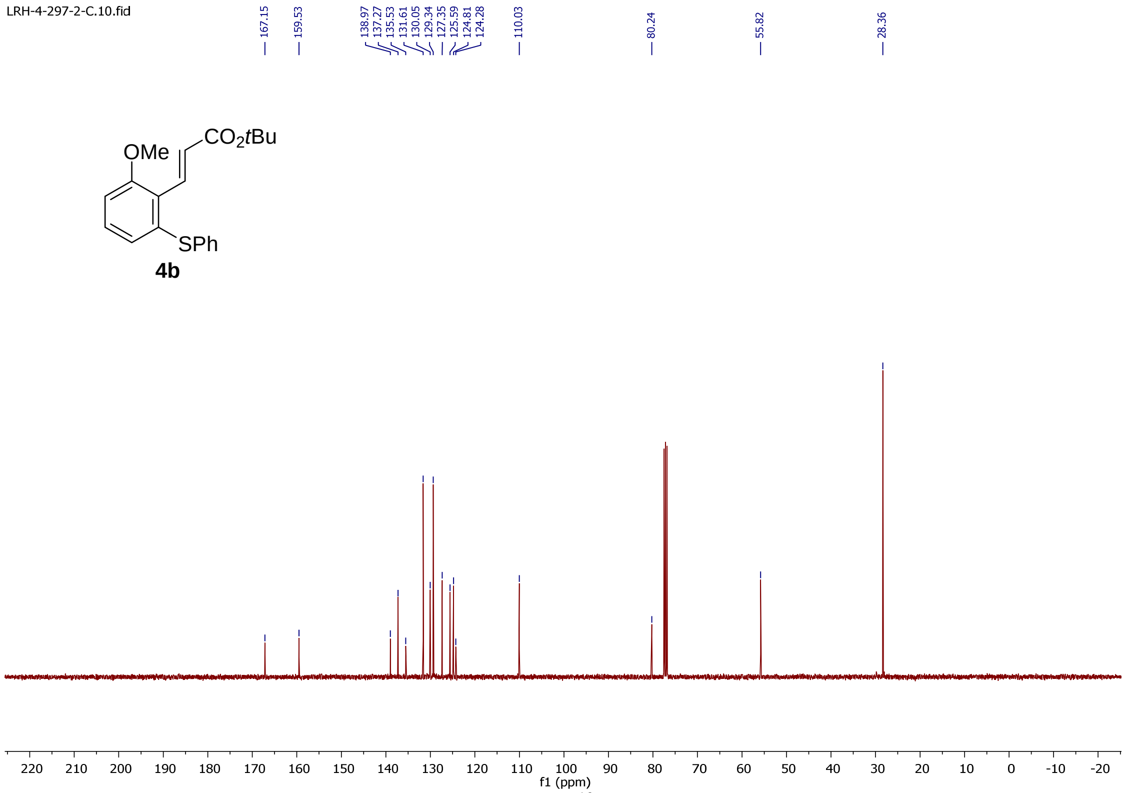

Supplementary Figure 70. ^{13}C NMR Spectrum of **4b**

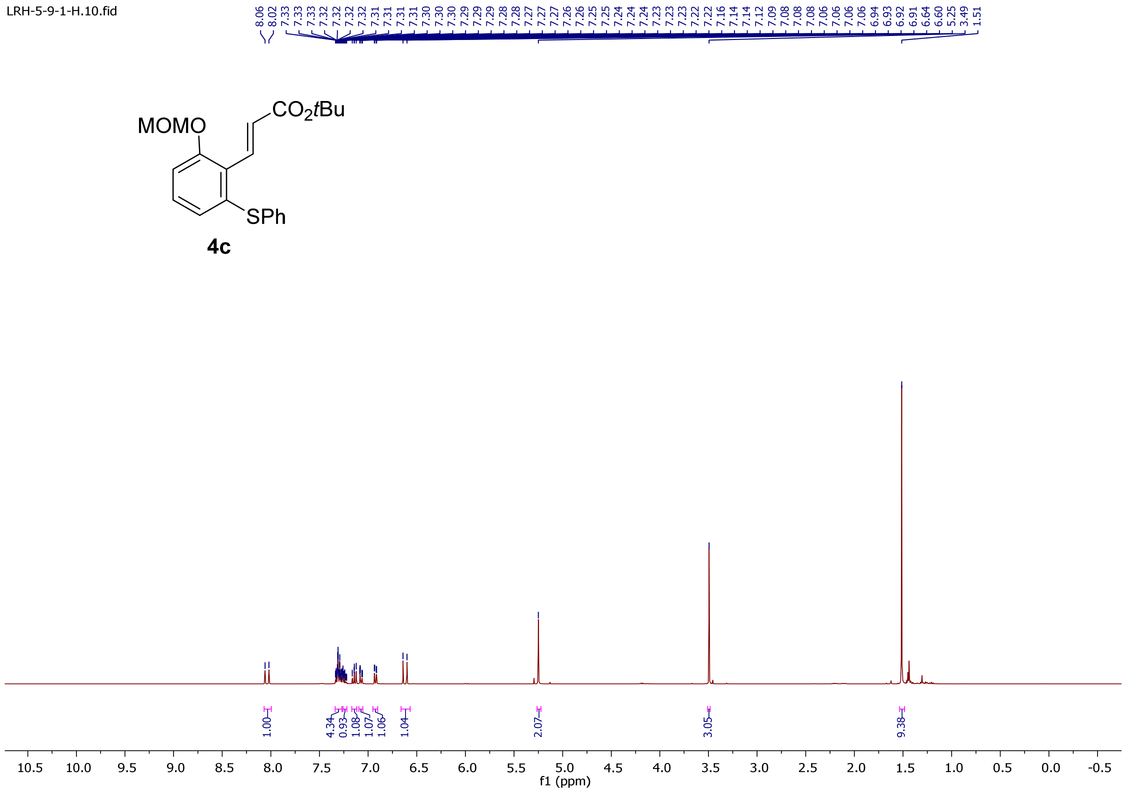

Supplementary Figure 71. ^{1}H NMR Spectrum of **4c**

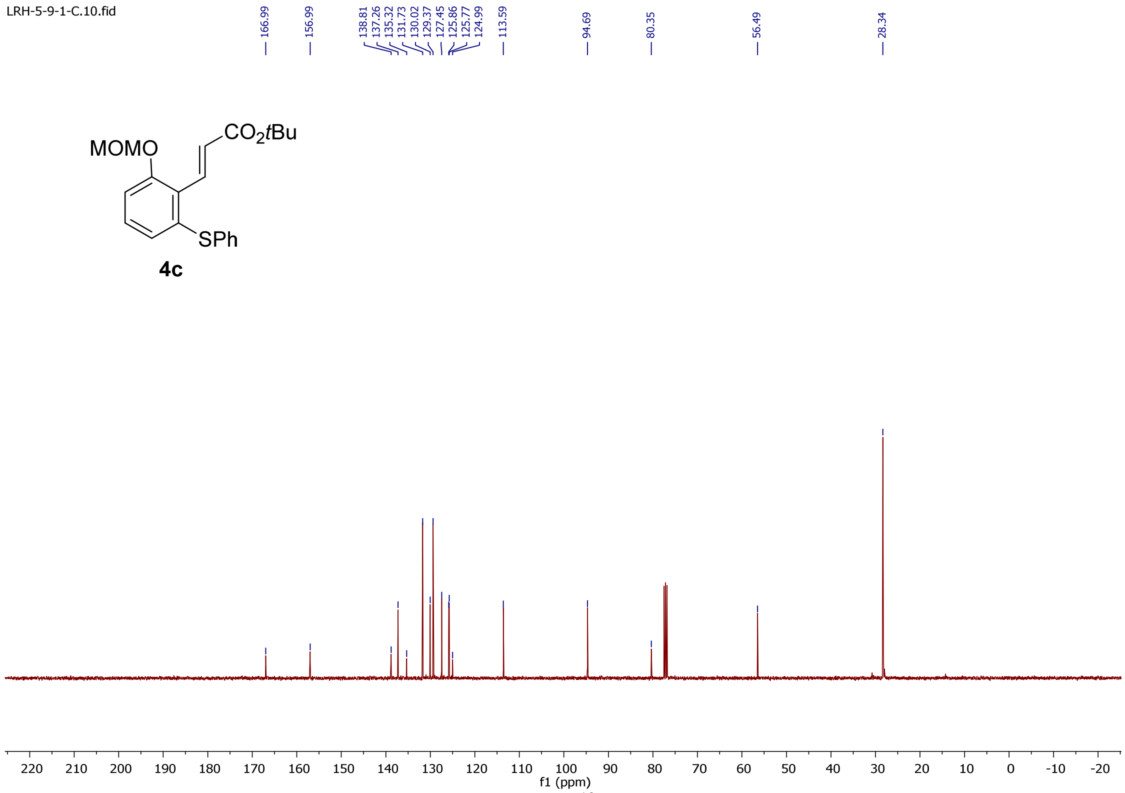

Supplementary Figure 72. ^{13}C NMR Spectrum of **4c**

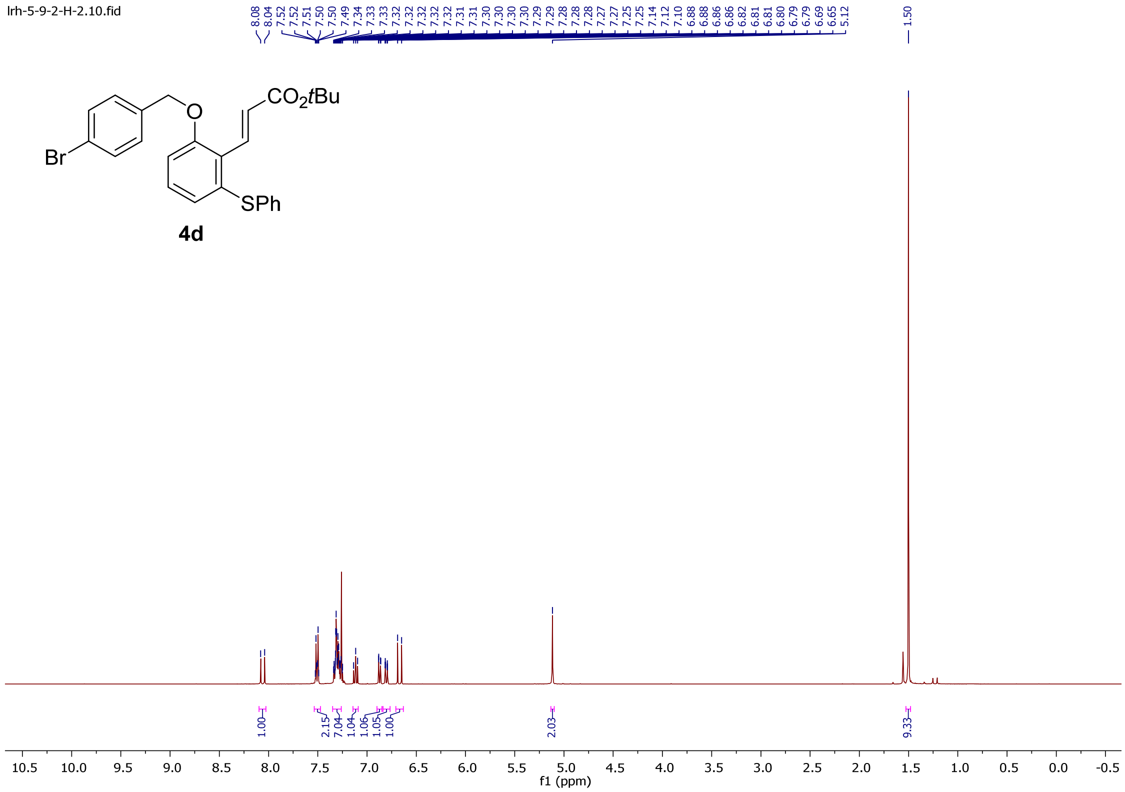

Supplementary Figure 73. ^{1}H NMR Spectrum of **4d**

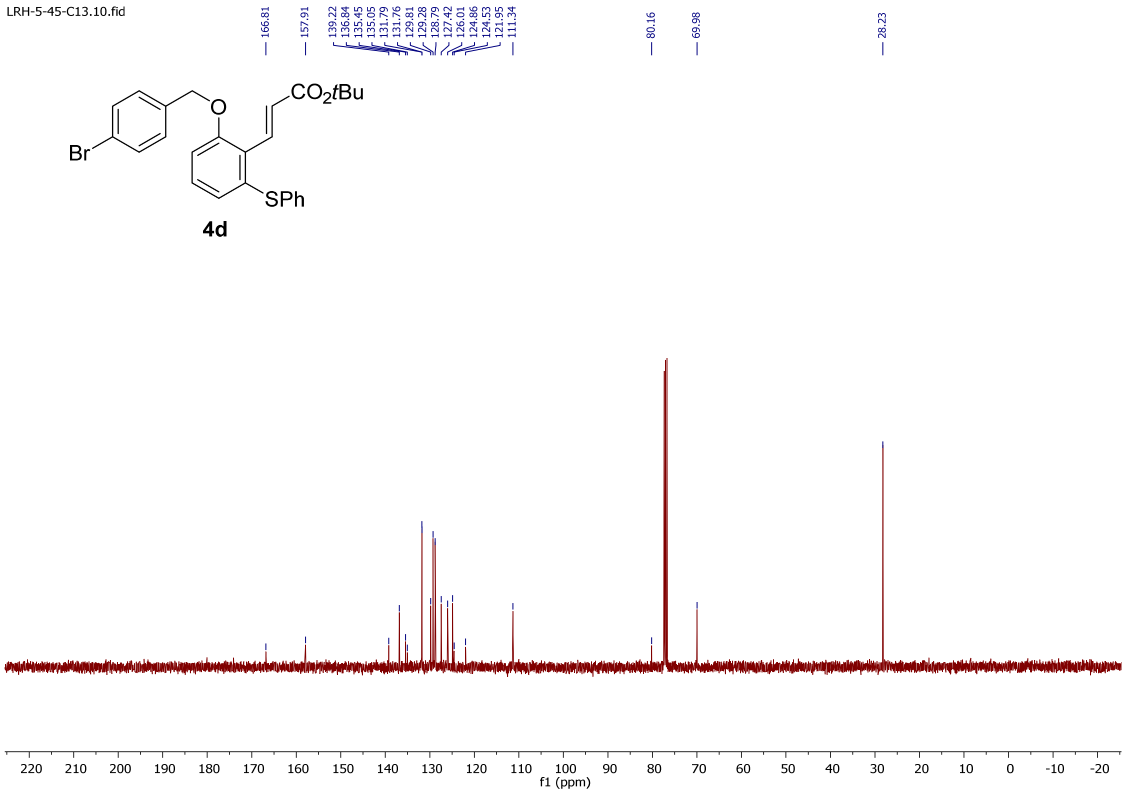

Supplementary Figure 74. ^{13}C NMR Spectrum of **4d**

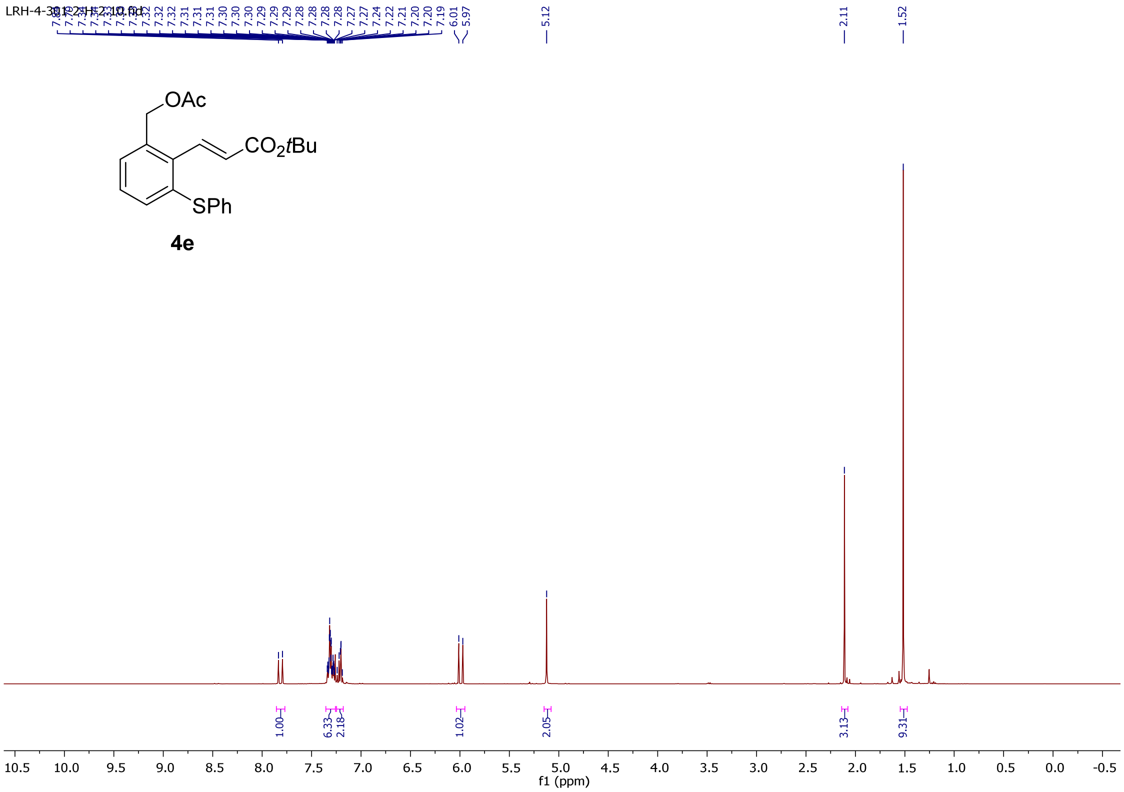

Supplementary Figure 75. ^{1}H NMR Spectrum of **4e**

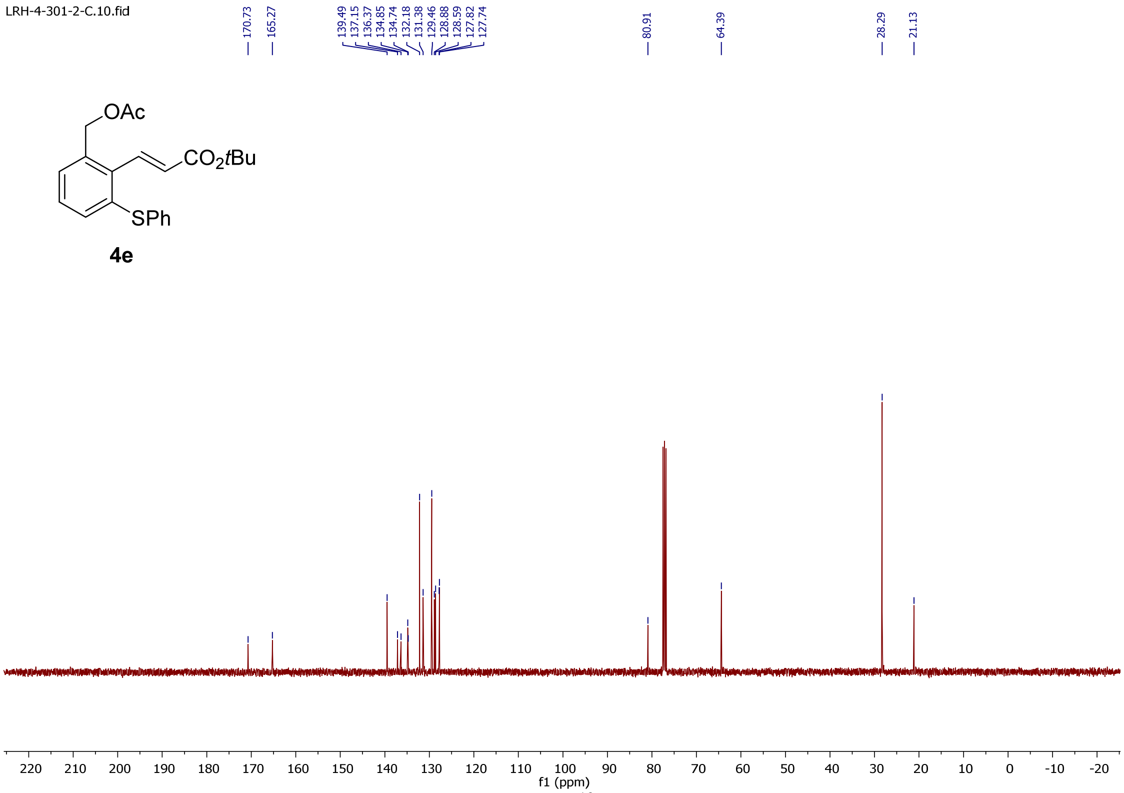

Supplementary Figure 76. ^{13}C NMR Spectrum of **4e**

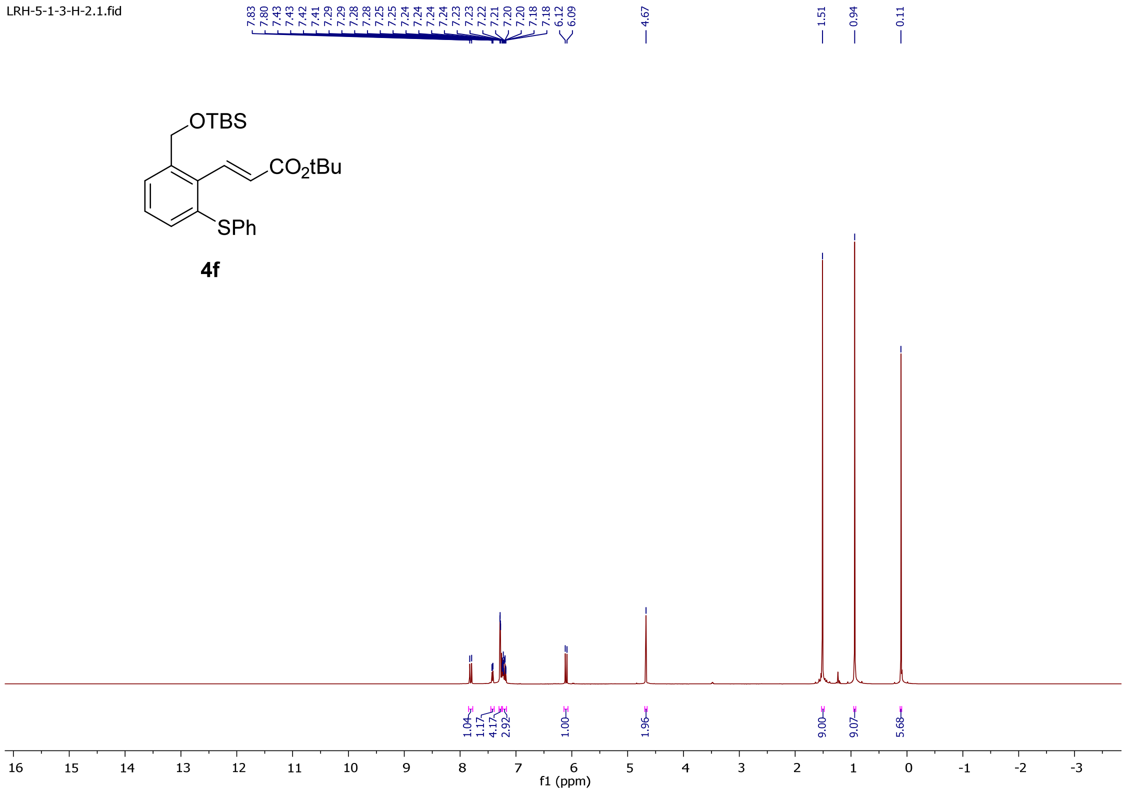

Supplementary Figure 77. ^{1}H NMR Spectrum of **4f**

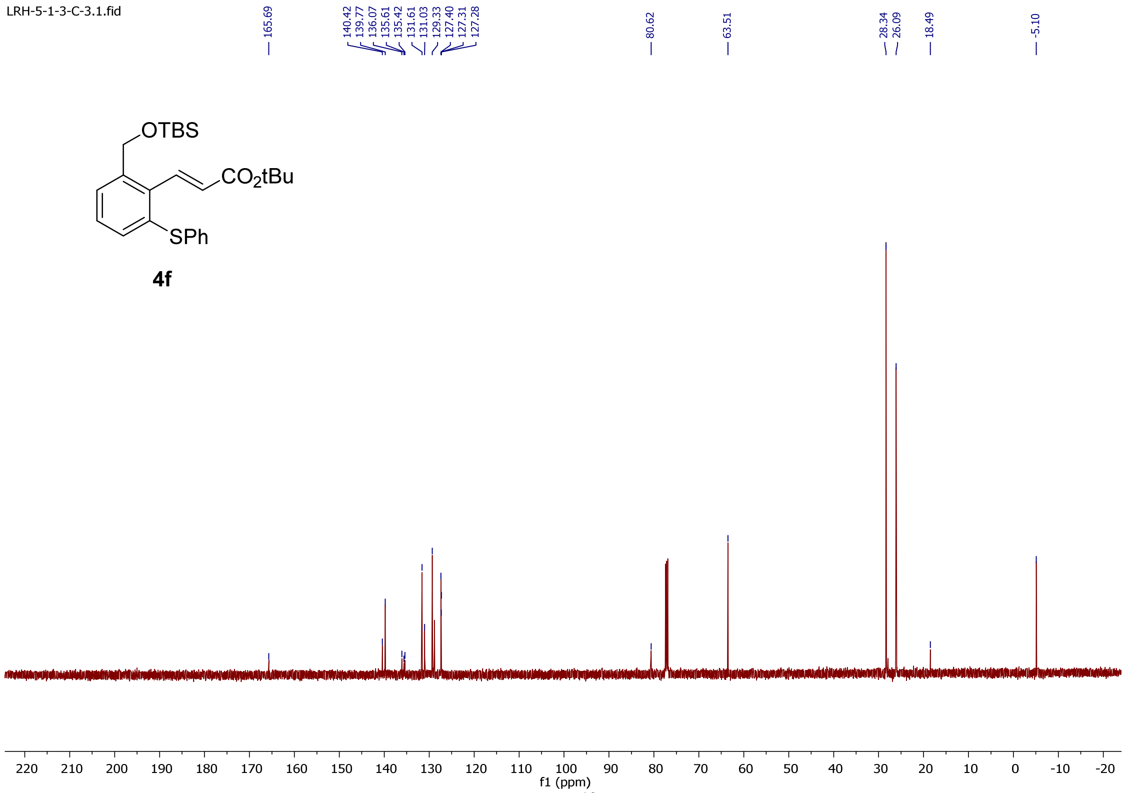

Supplementary Figure 78. ^{13}C NMR Spectrum of **4f**

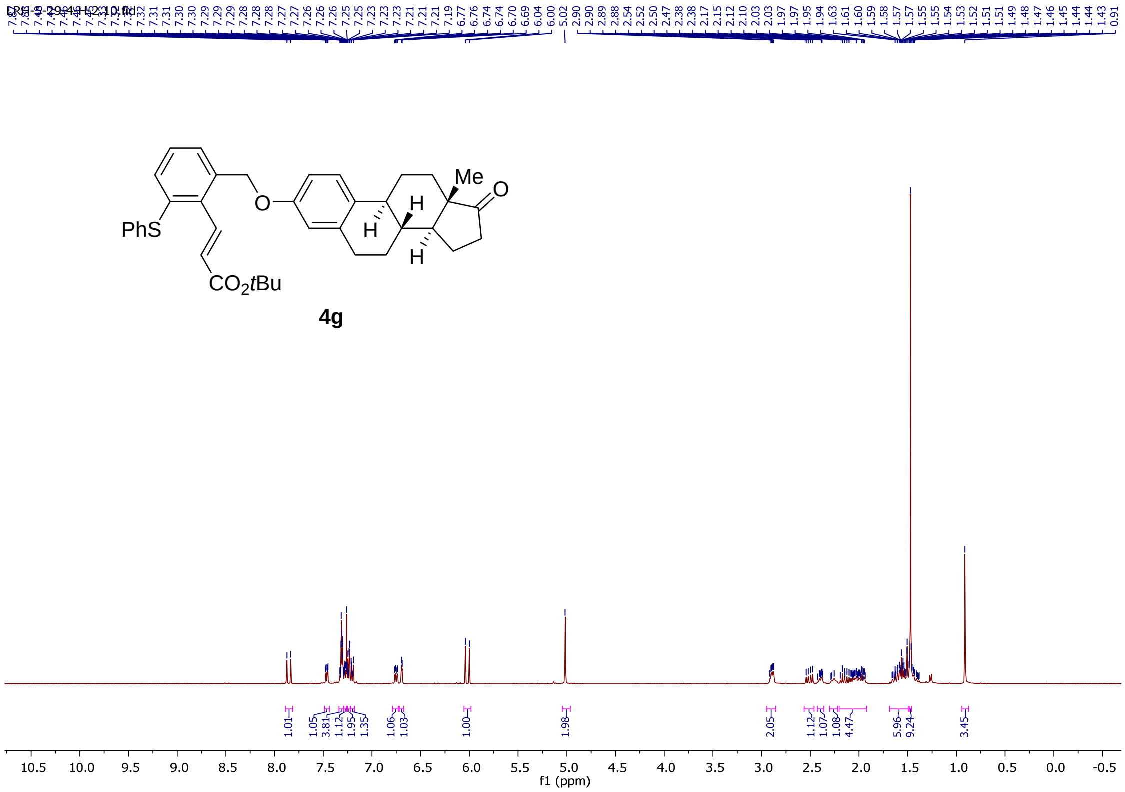

Supplementary Figure 79. ^{1}H NMR Spectrum of **4g**

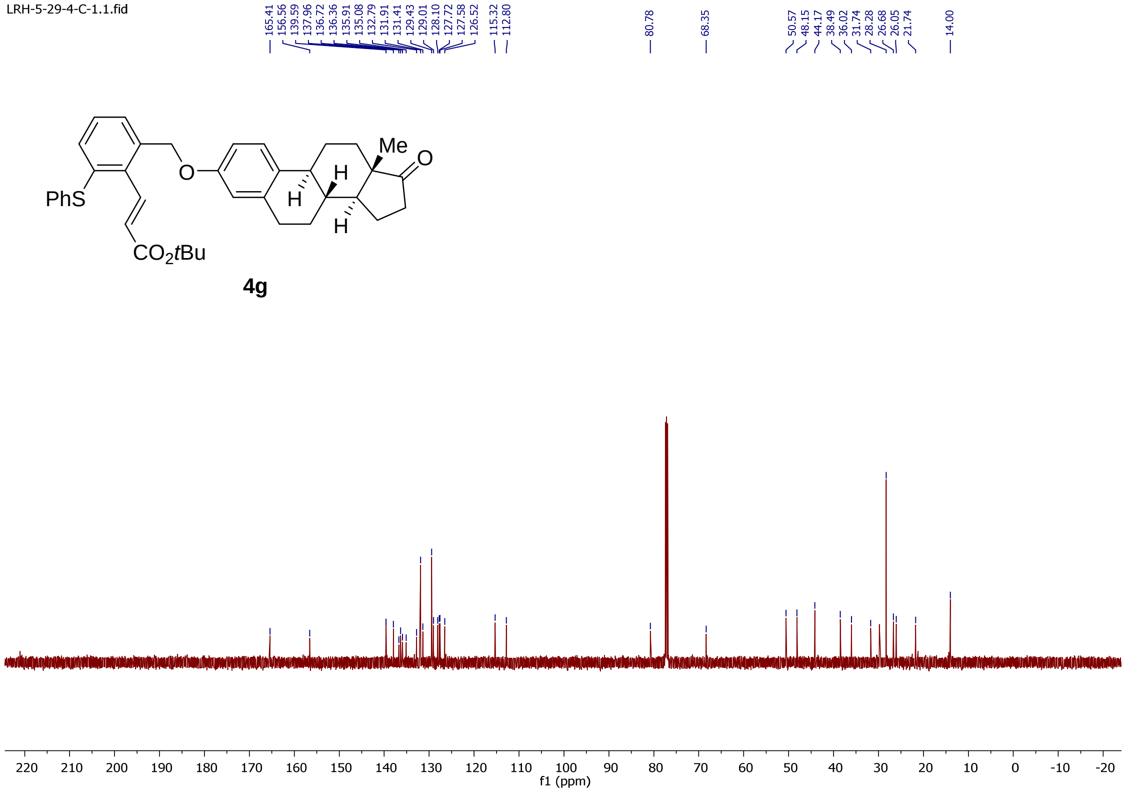

Supplementary Figure 80. ^{13}C NMR Spectrum of **4g**

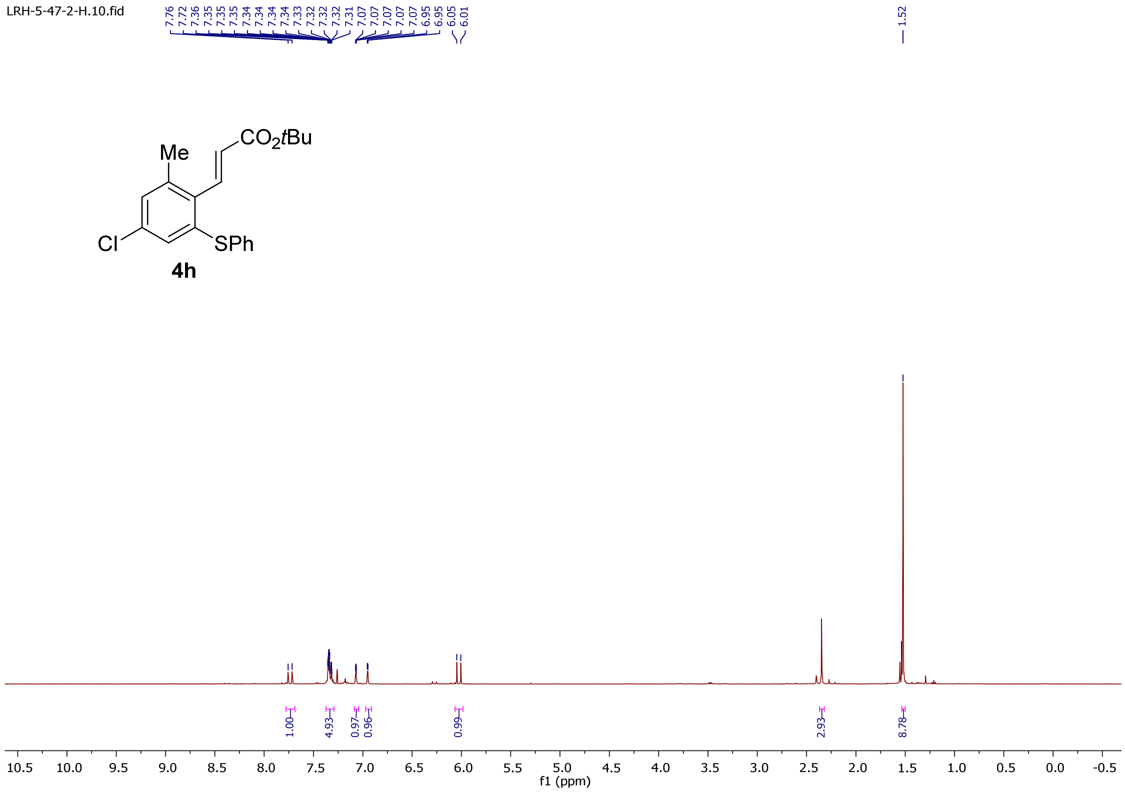

Supplementary Figure 81. ^{1}H NMR Spectrum of **4h**

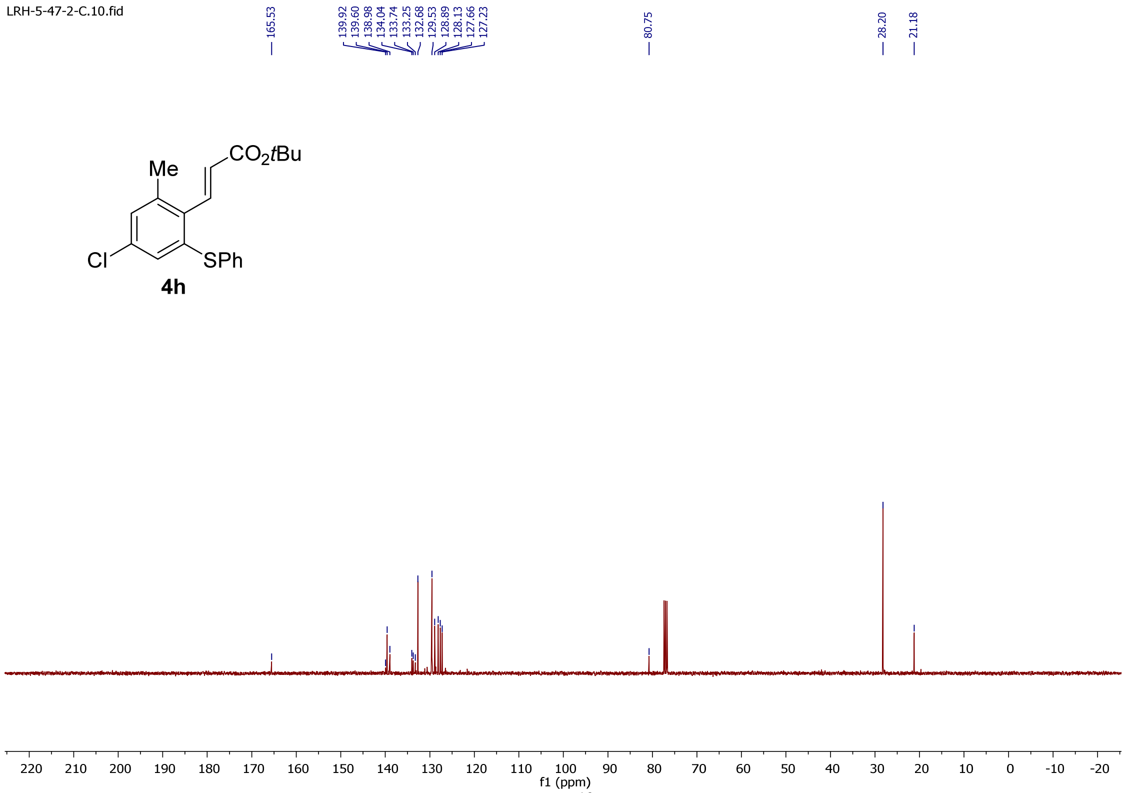

Supplementary Figure 82. ^{13}C NMR Spectrum of **4h**

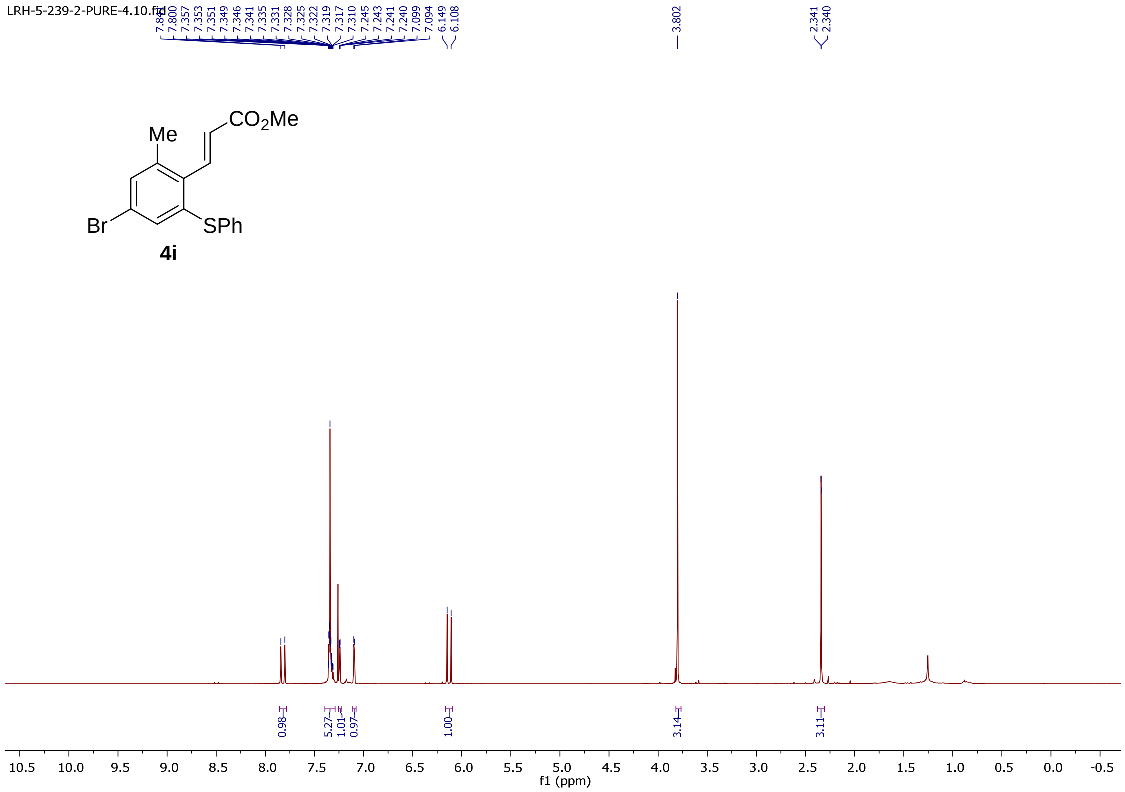

Supplementary Figure 83. ^{1}H NMR Spectrum of **4i**

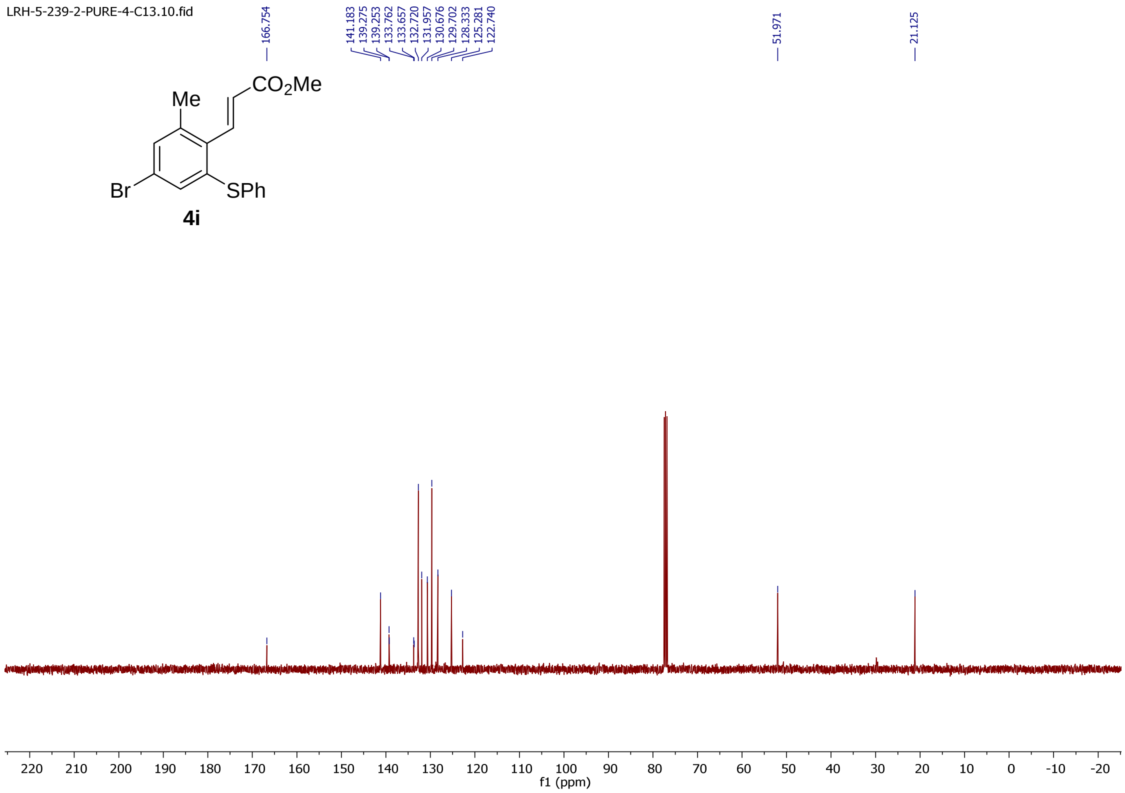

Supplementary Figure 84. ^{13}C NMR Spectrum of **4i**

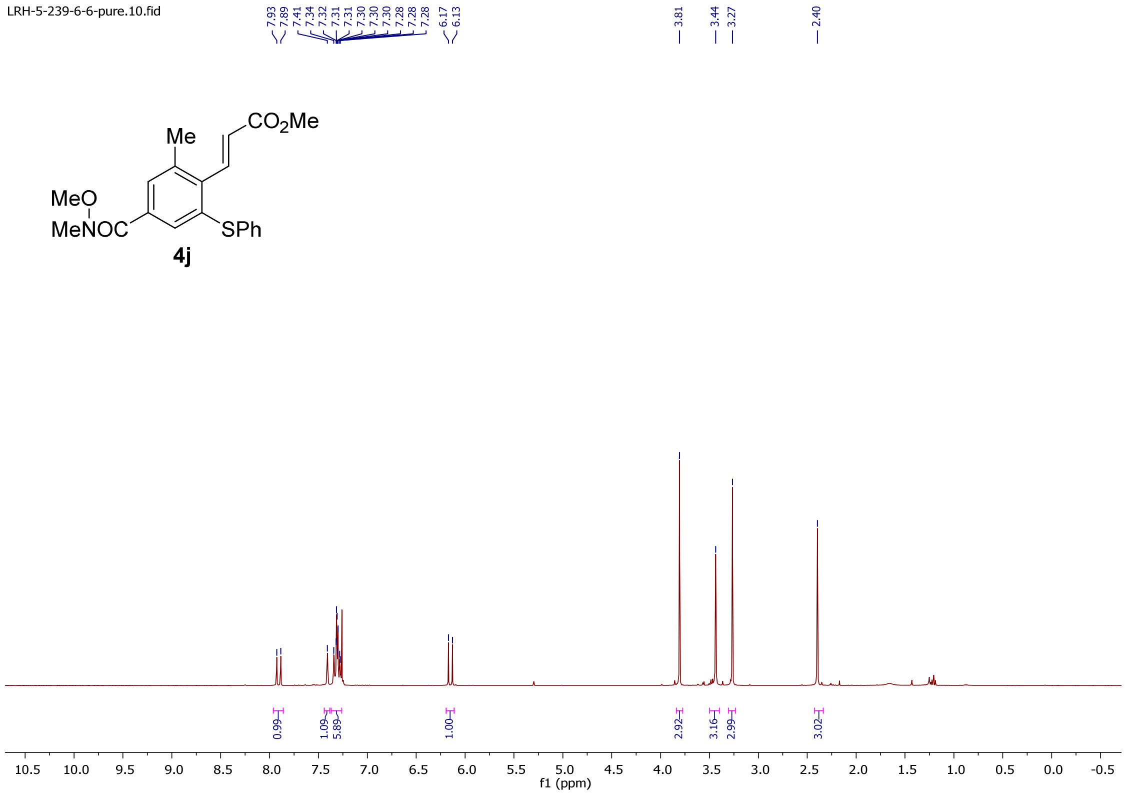

Supplementary Figure 85. ^{1}H NMR Spectrum of **4j**

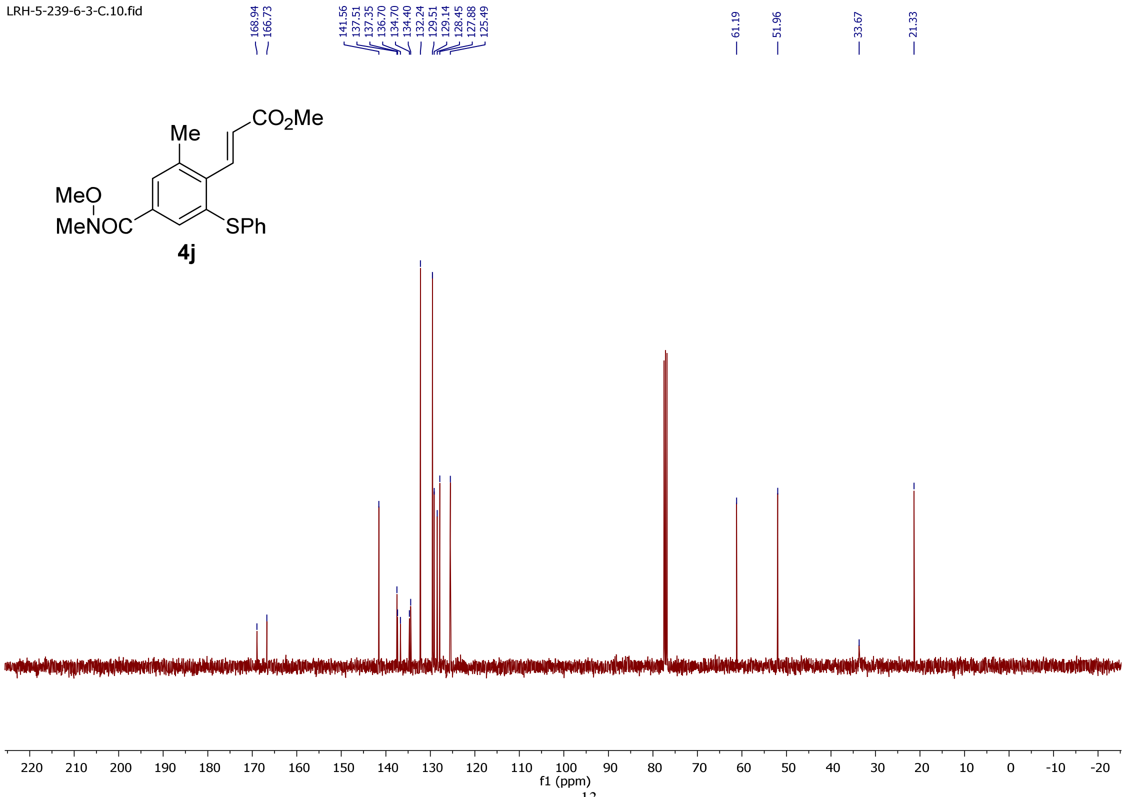

Supplementary Figure 86. ^{13}C NMR Spectrum of **4j**

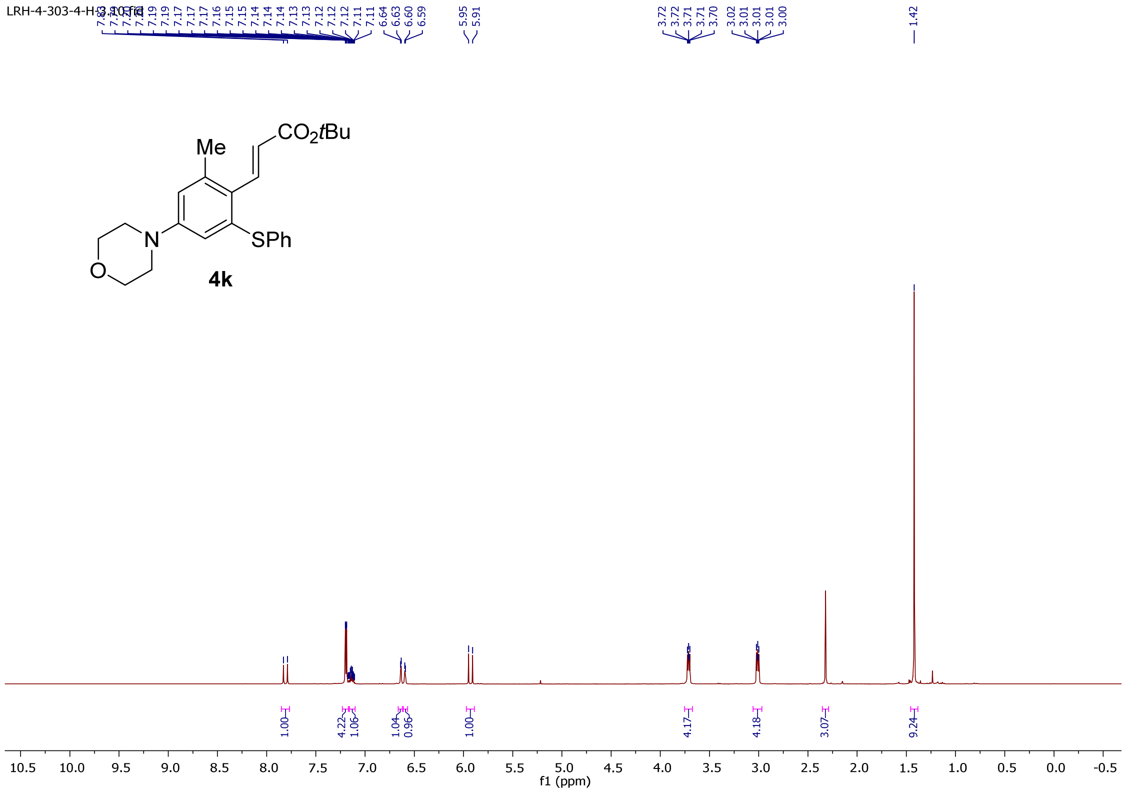

Supplementary Figure 87. ^{1}H NMR Spectrum of **4k**

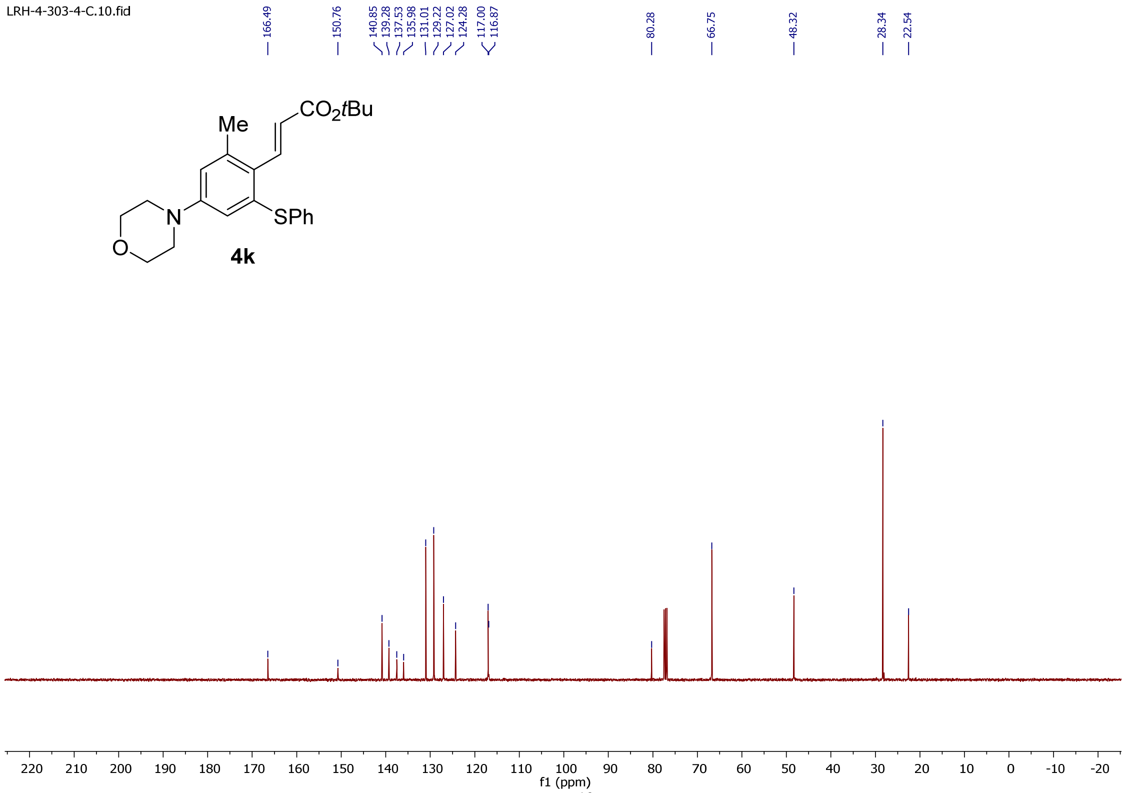

Supplementary Figure 88. ^{13}C NMR Spectrum of **4k**

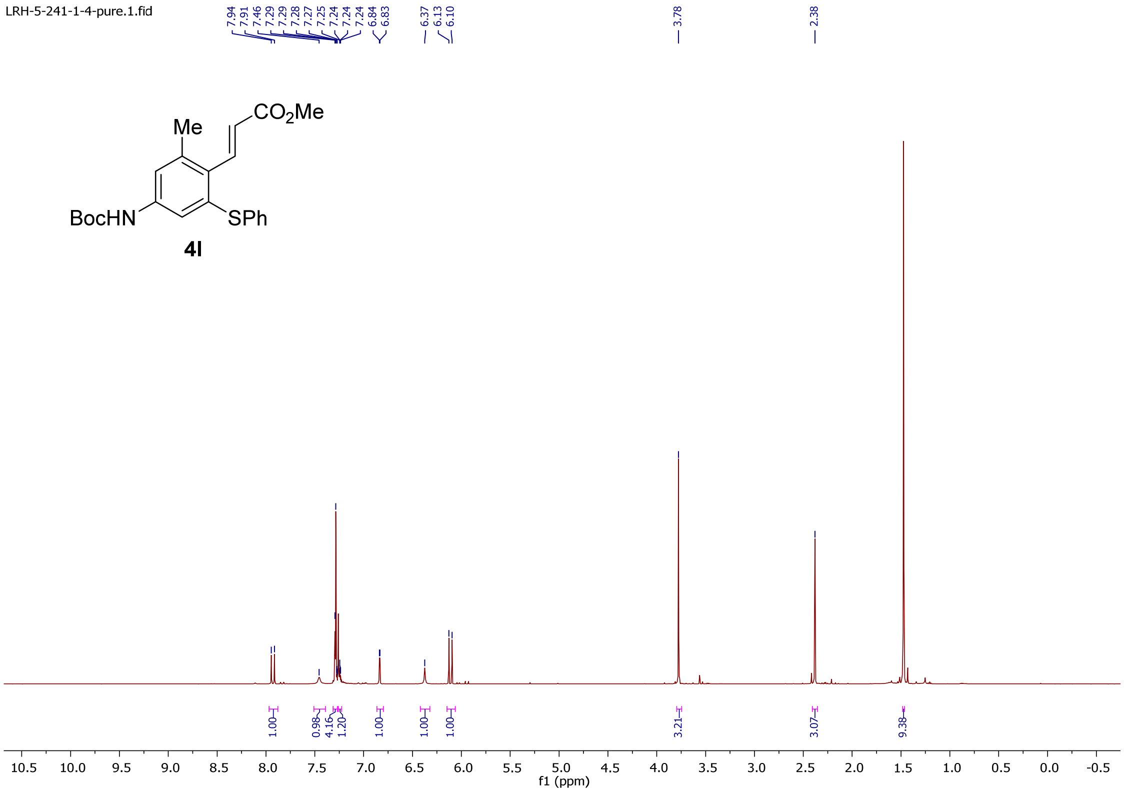

Supplementary Figure 89. ^{1}H NMR Spectrum of **4l**

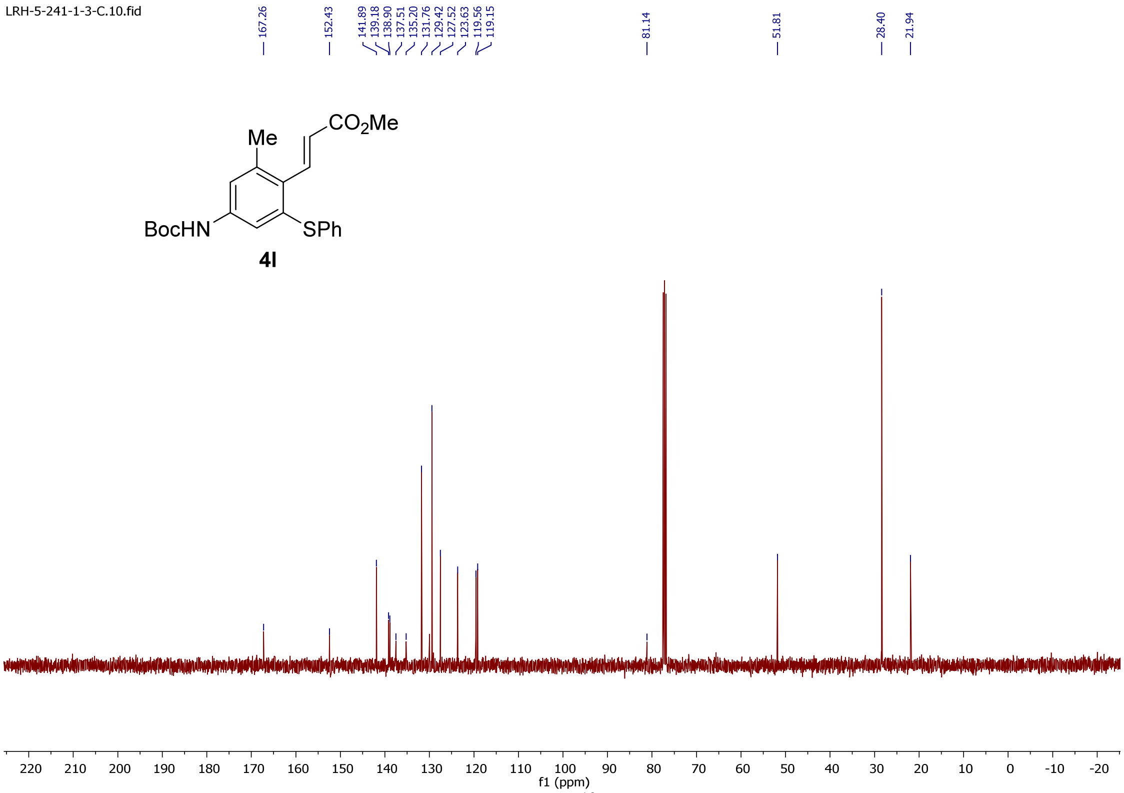

Supplementary Figure 90. ^{13}C NMR Spectrum of **4l**

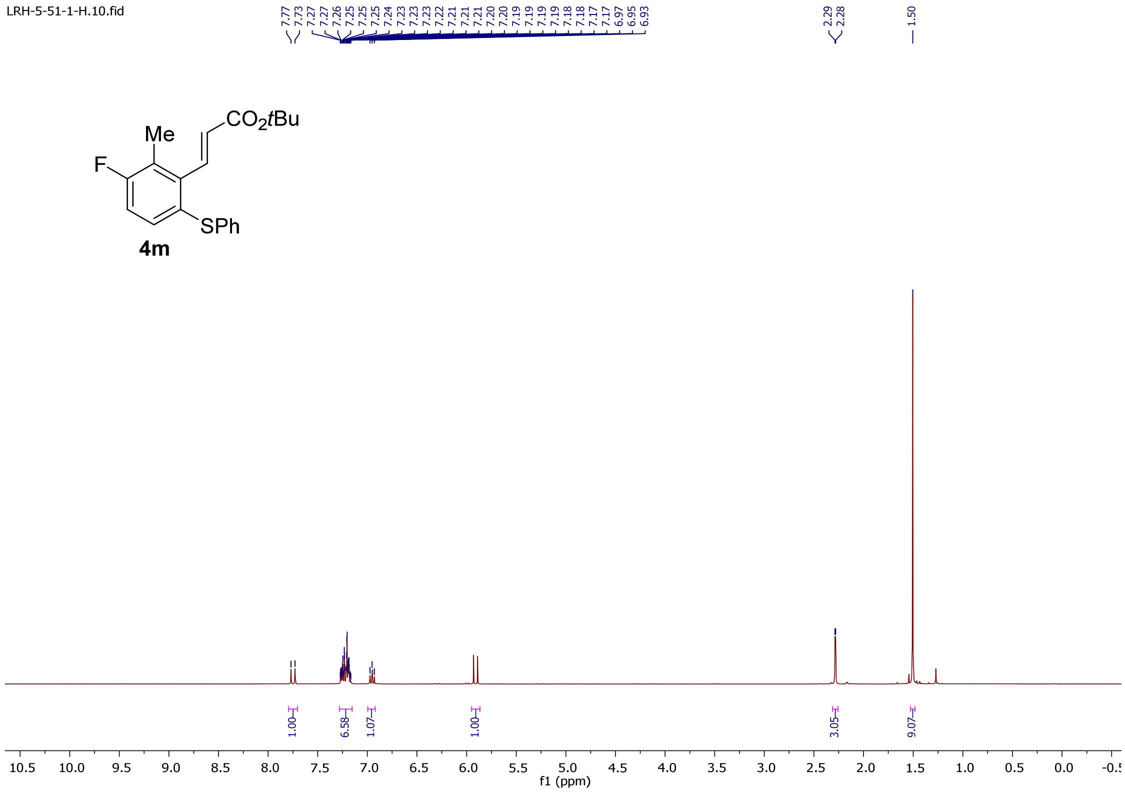

Supplementary Figure 91. ^{1}H NMR Spectrum of **4m**

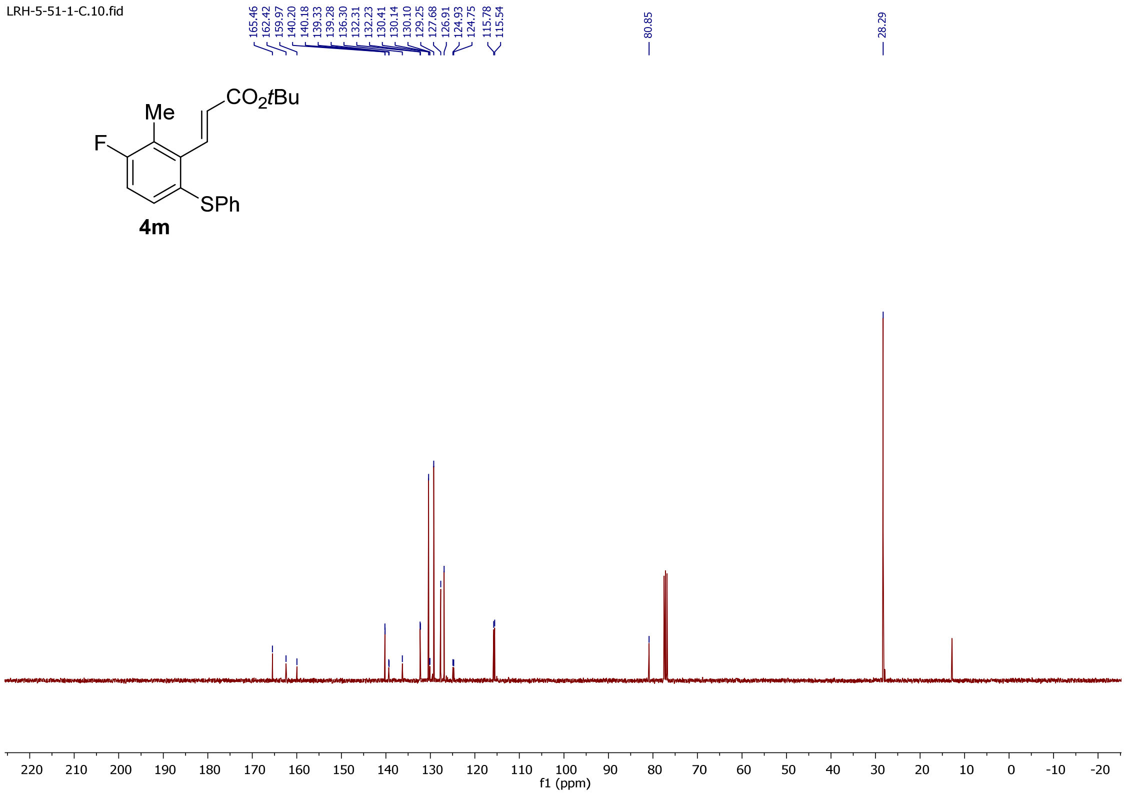

Supplementary Figure 92. ^{13}C NMR Spectrum of **4m**

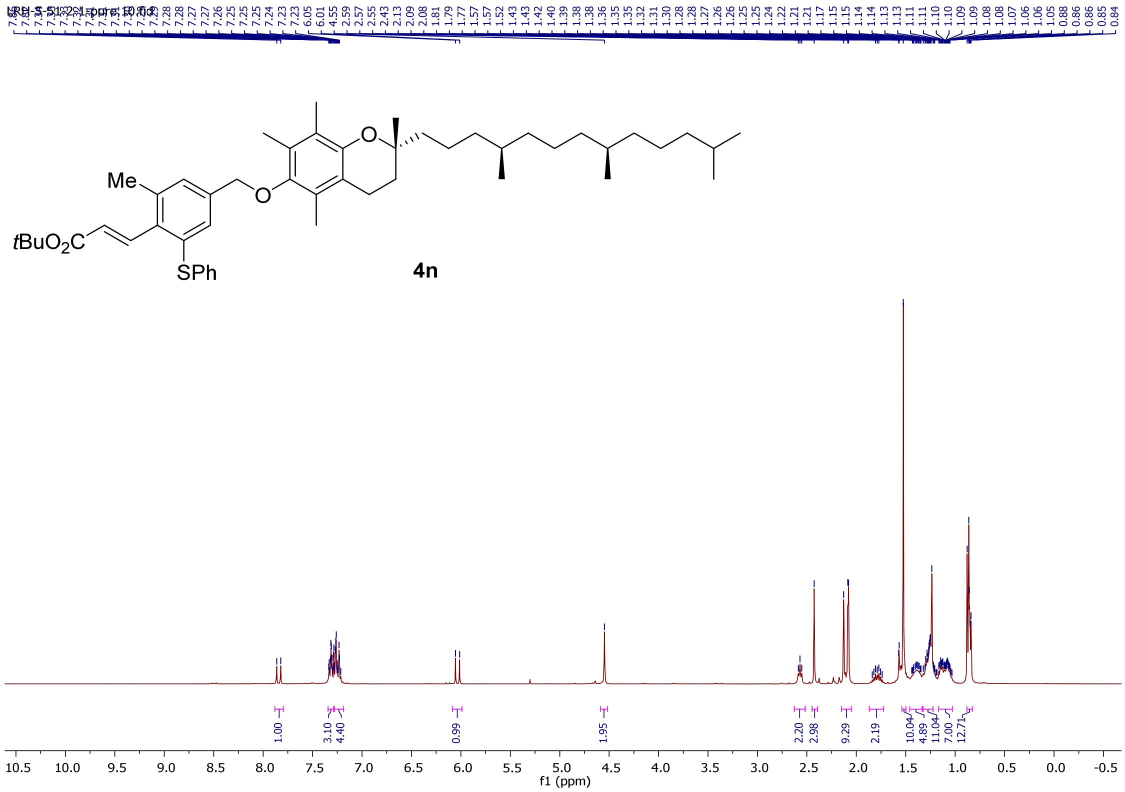

Supplementary Figure 93. ^{1}H NMR Spectrum of **4n**

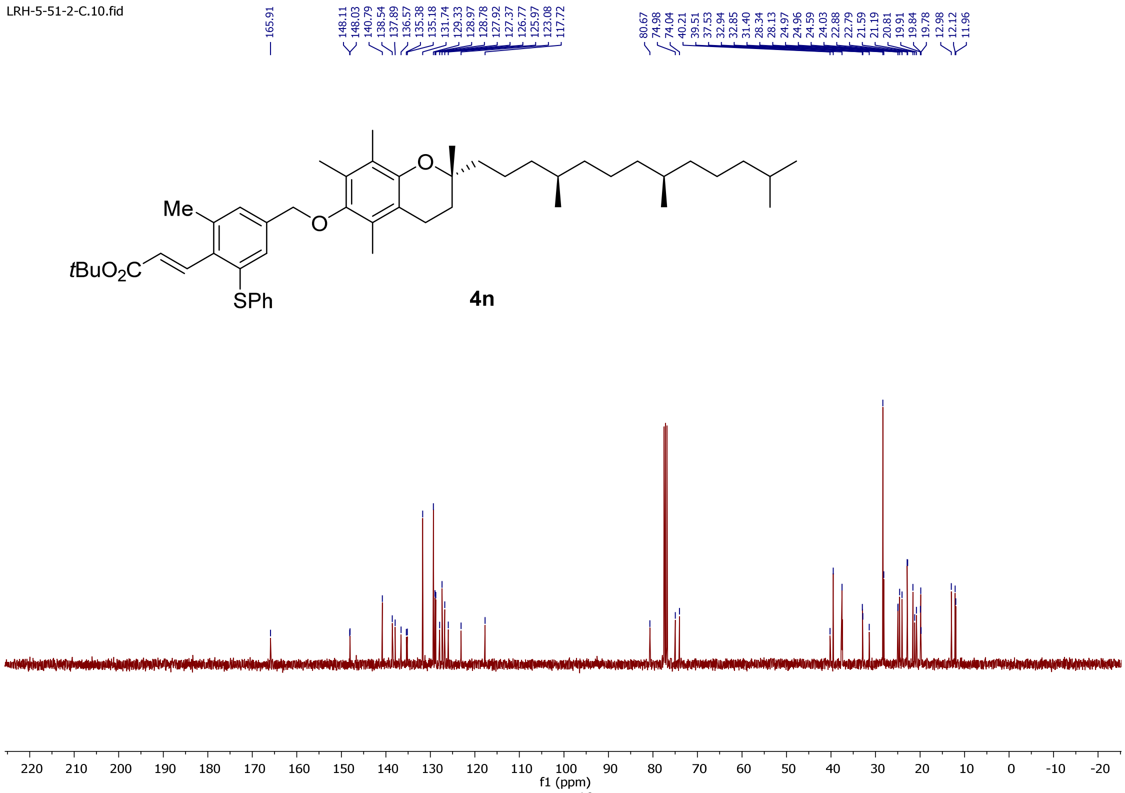

Supplementary Figure 94. ^{13}C NMR Spectrum of **4n**

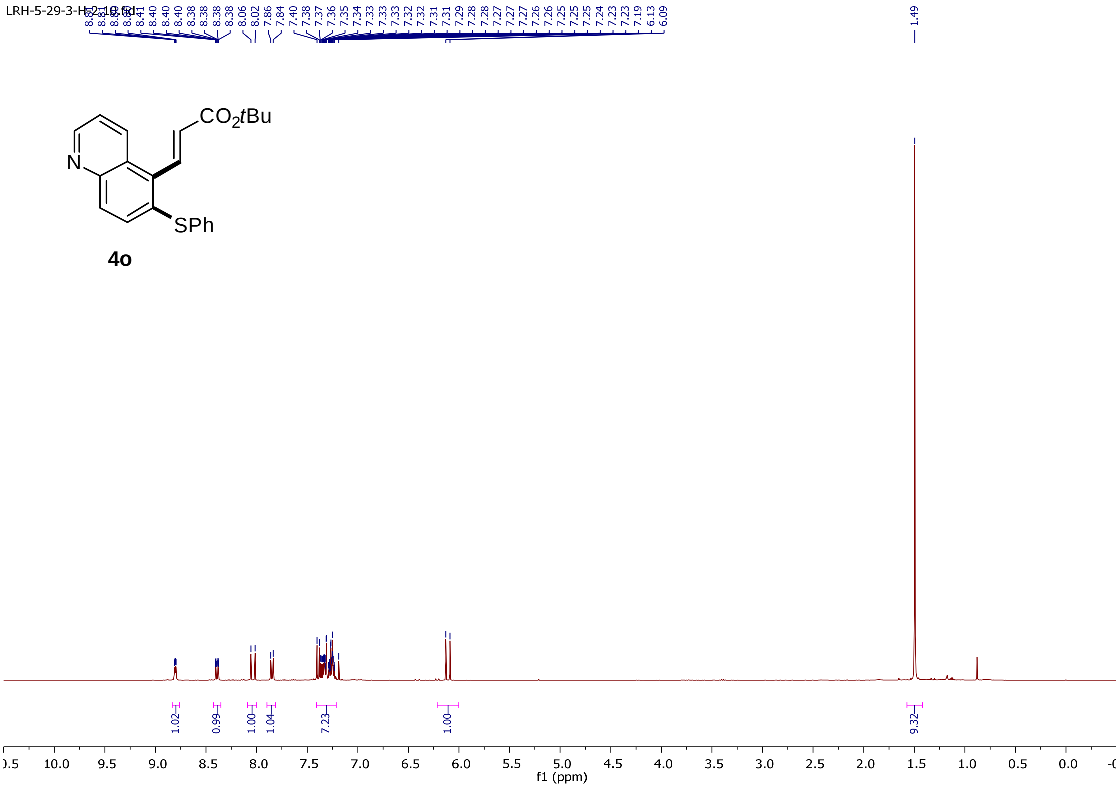

Supplementary Figure 95. ^{1}H NMR Spectrum of **4o**

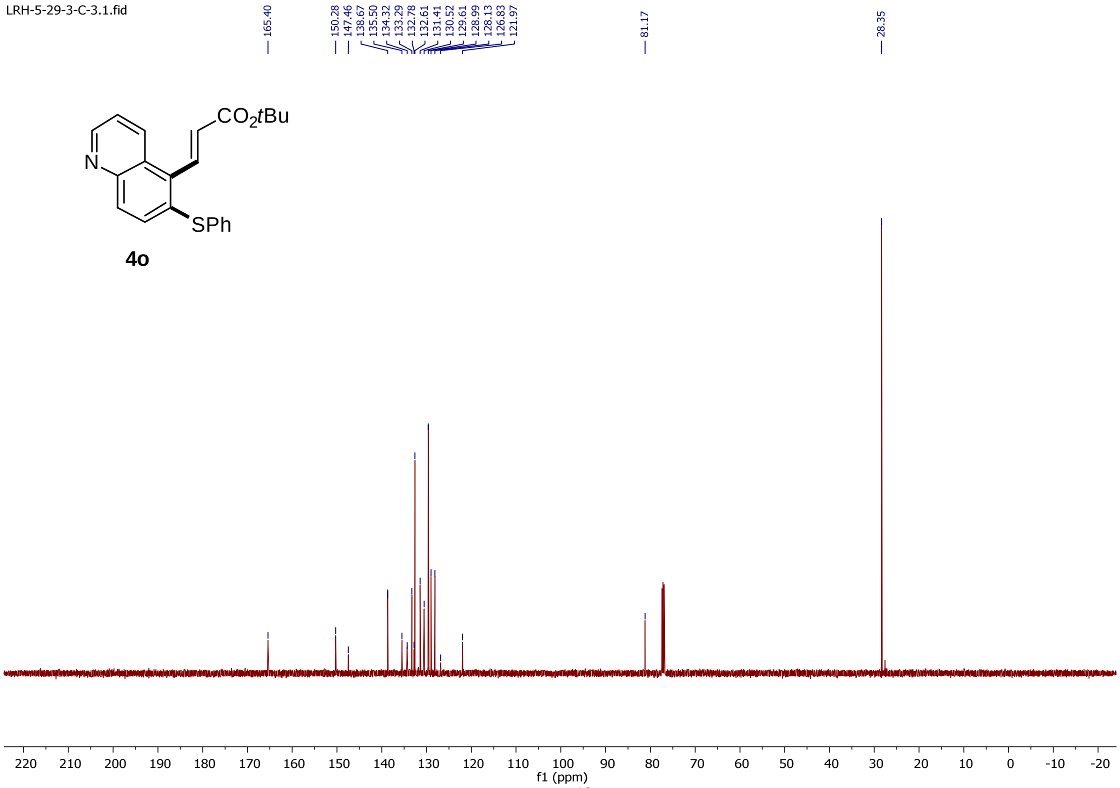

Supplementary Figure 96. ^{13}C NMR Spectrum of **4o**

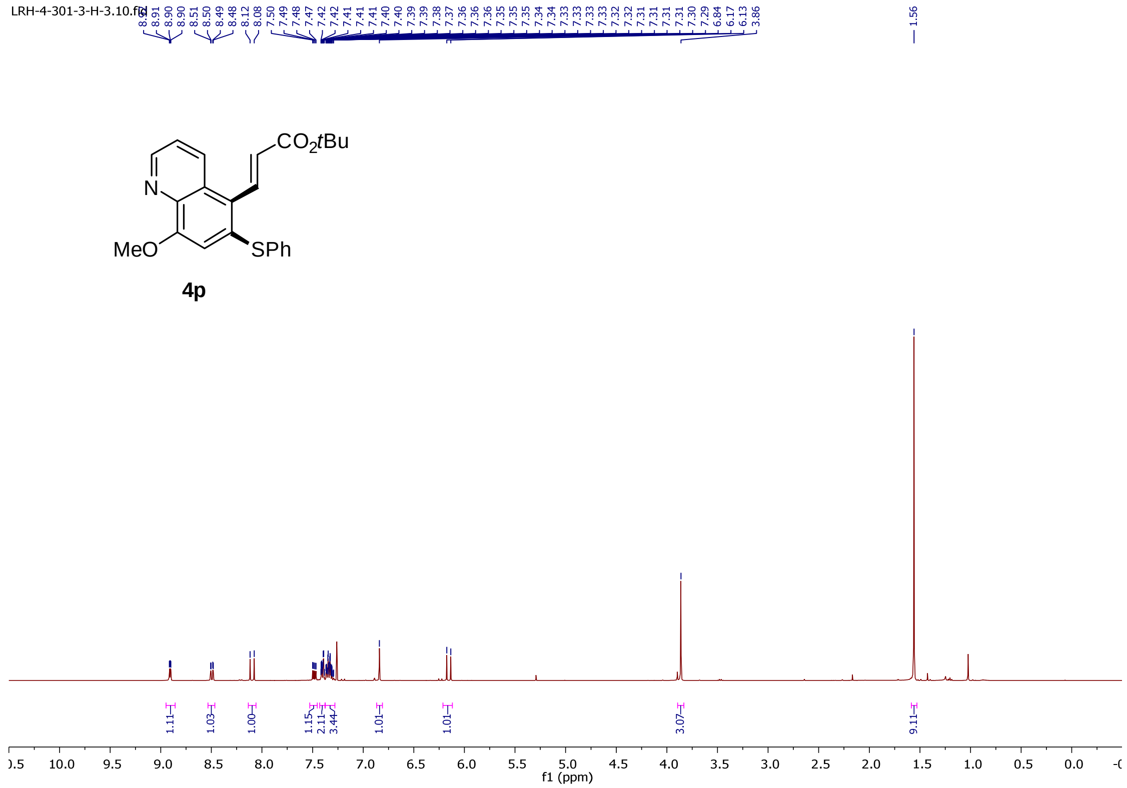

Supplementary Figure 97. ^{1}H NMR Spectrum of **4p**

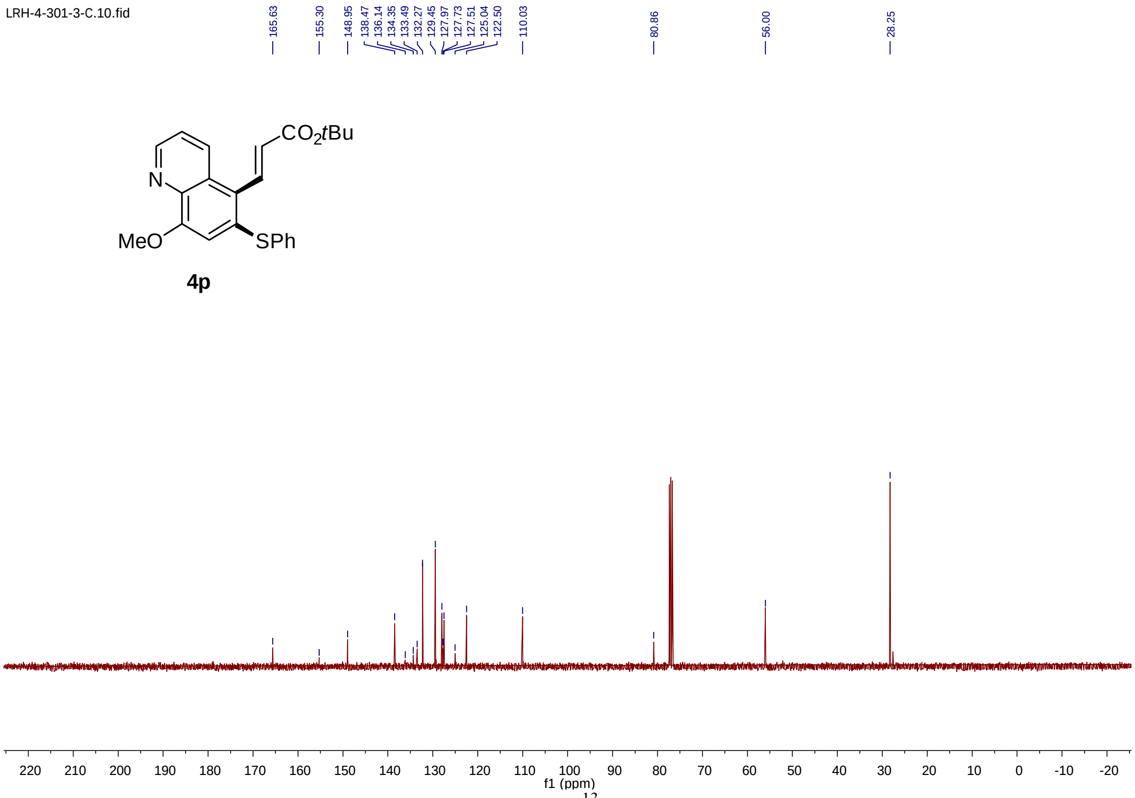

Supplementary Figure 98. ^{13}C NMR Spectrum of **4p**

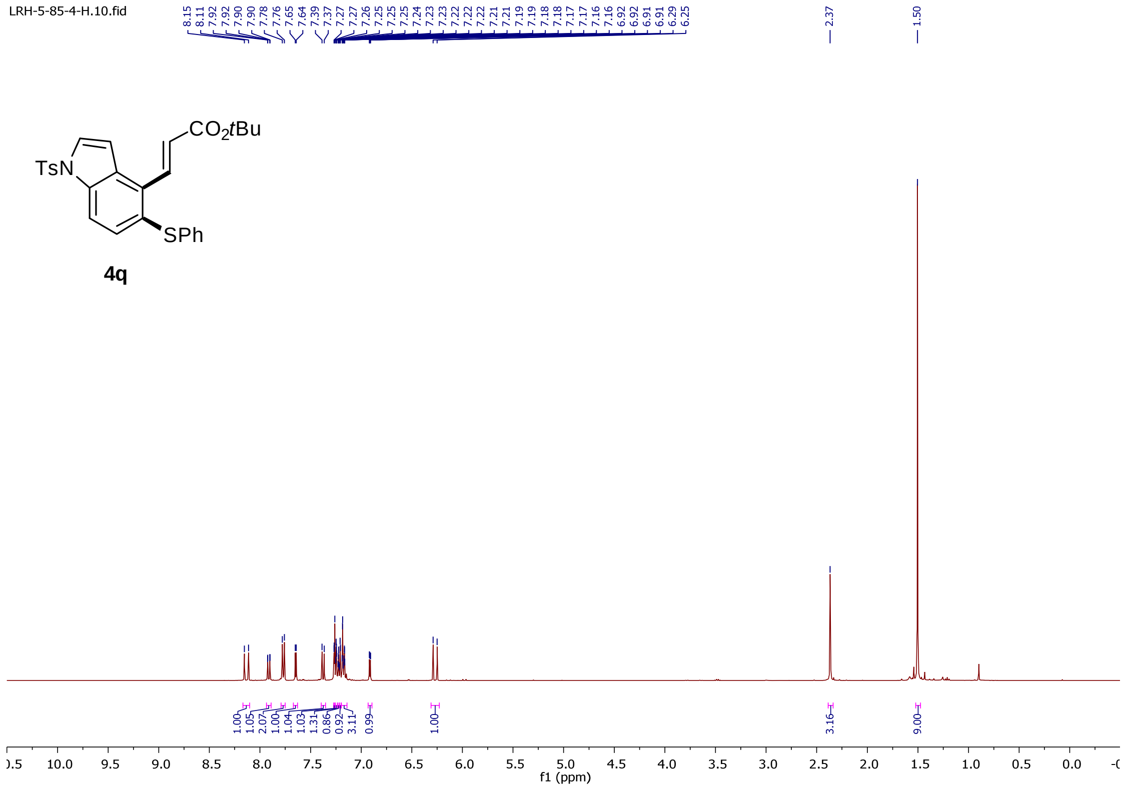

Supplementary Figure 99. ^{1}H NMR Spectrum of **4q**

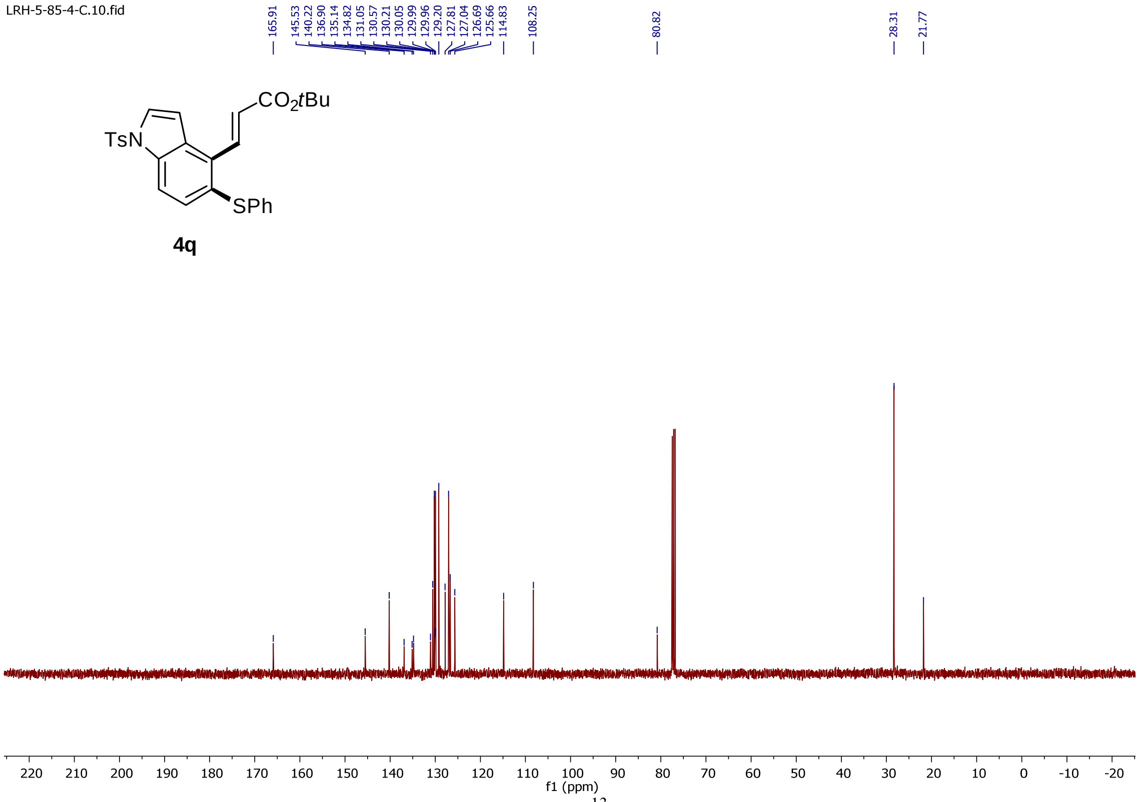

Supplementary Figure 100. ^{13}C NMR Spectrum of **4q**

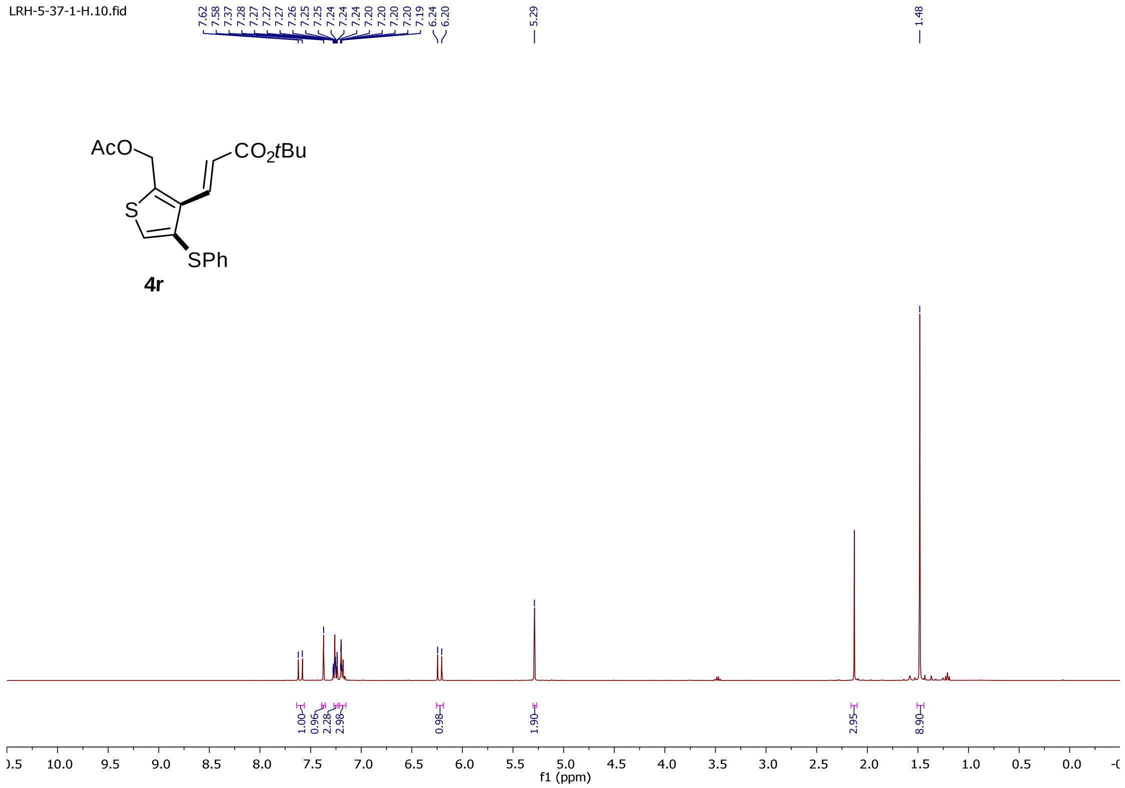

Supplementary Figure 101. ^{1}H NMR Spectrum of **4r**

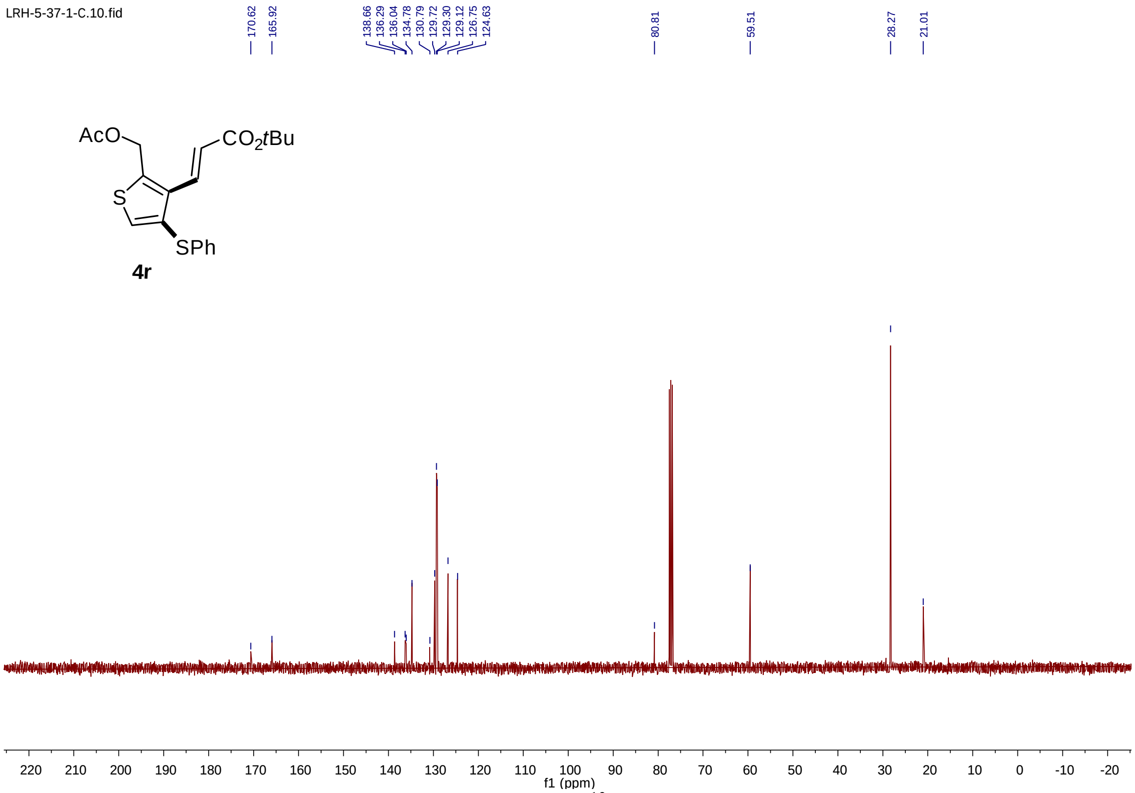

Supplementary Figure 102. ^{13}C NMR Spectrum of **4r**

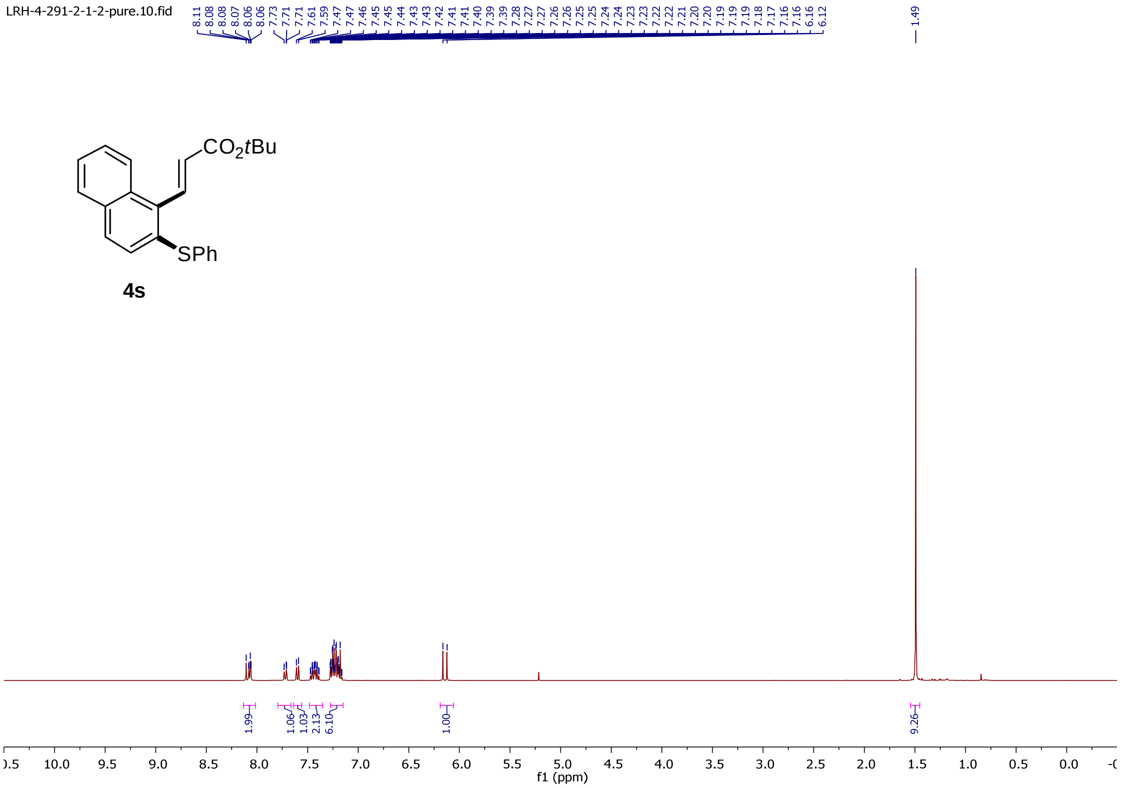

Supplementary Figure 103. ^{1}H NMR Spectrum of **4s**

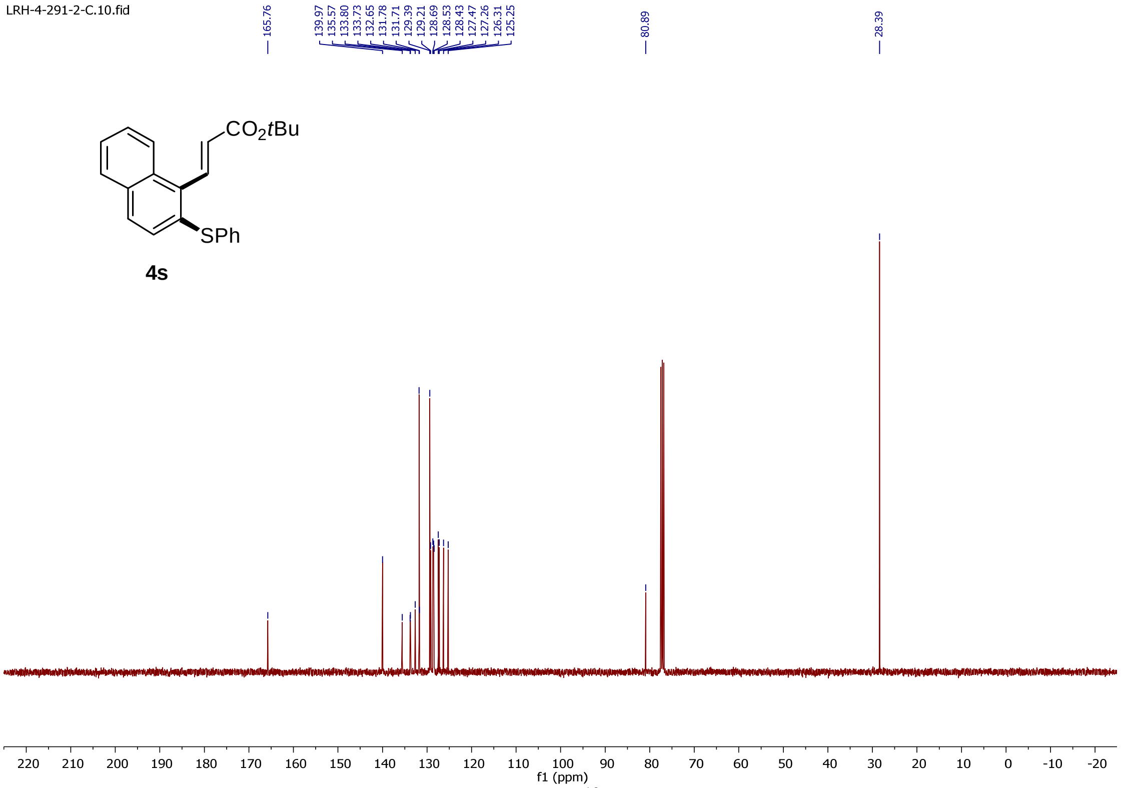

Supplementary Figure 104. ^{13}C NMR Spectrum of **4s**

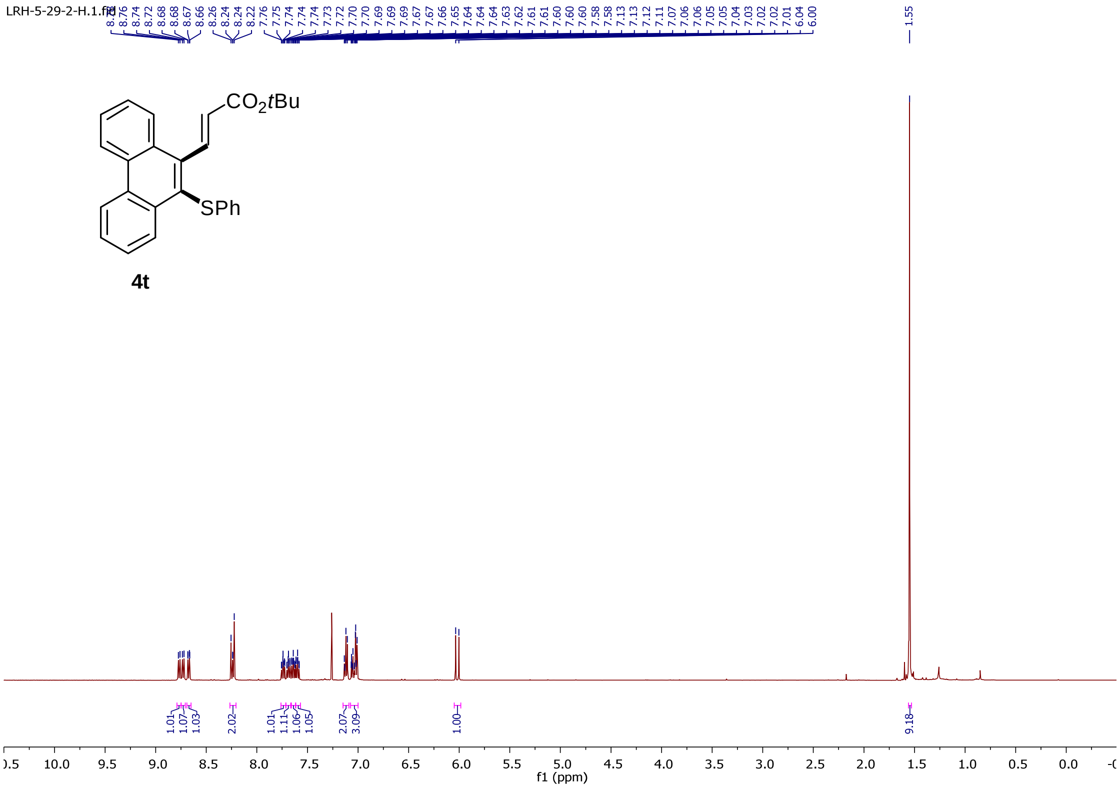

Supplementary Figure 105. ^{1}H NMR Spectrum of **4t**

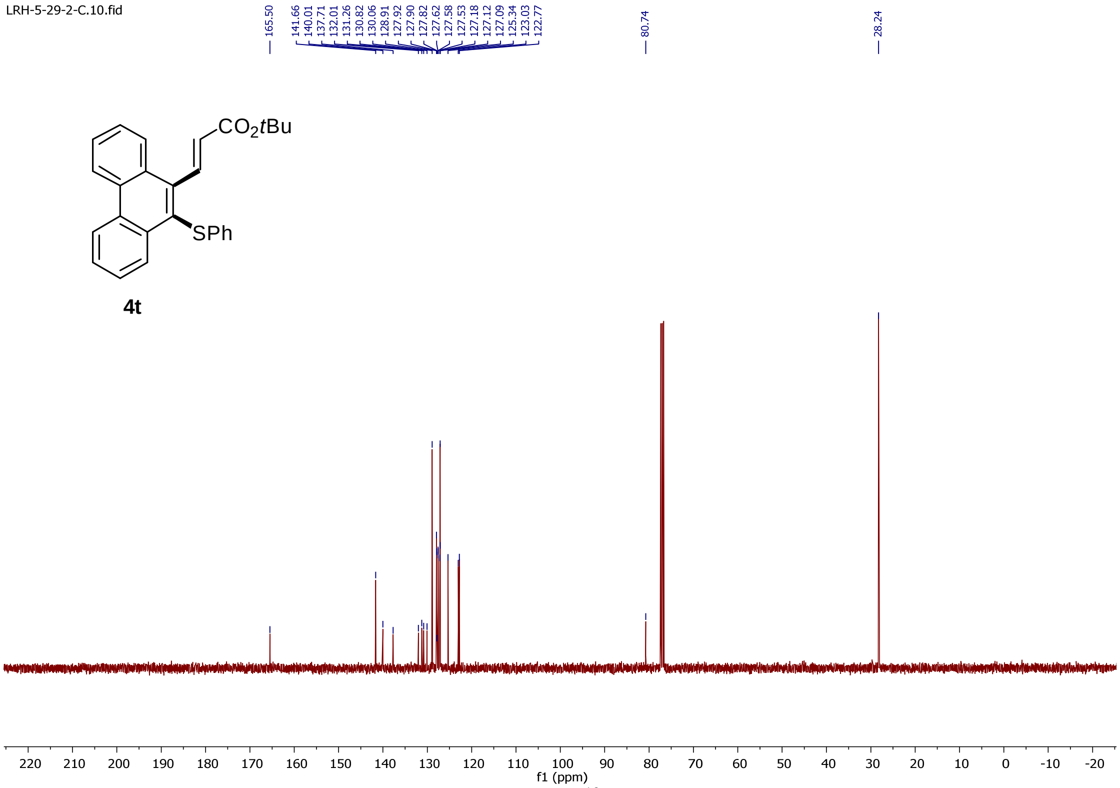

Supplementary Figure 106. ^{13}C NMR Spectrum of **4t**

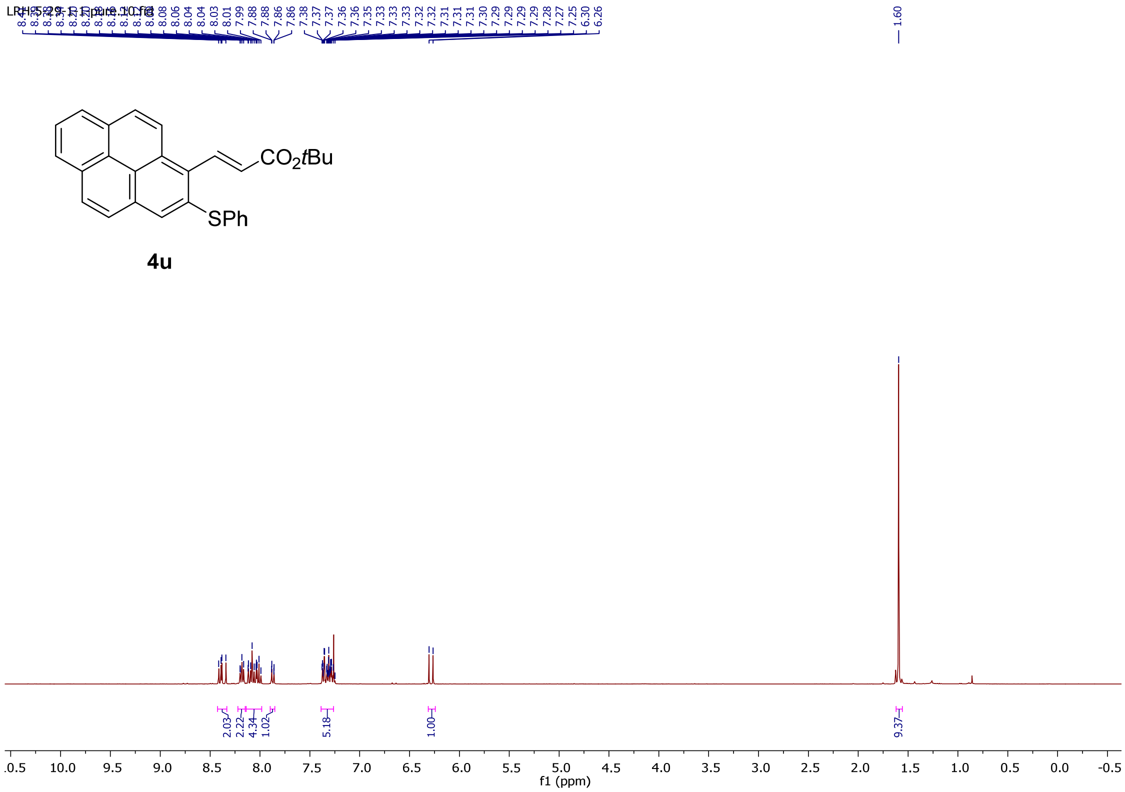

Supplementary Figure 107. ^{1}H NMR Spectrum of **4u**

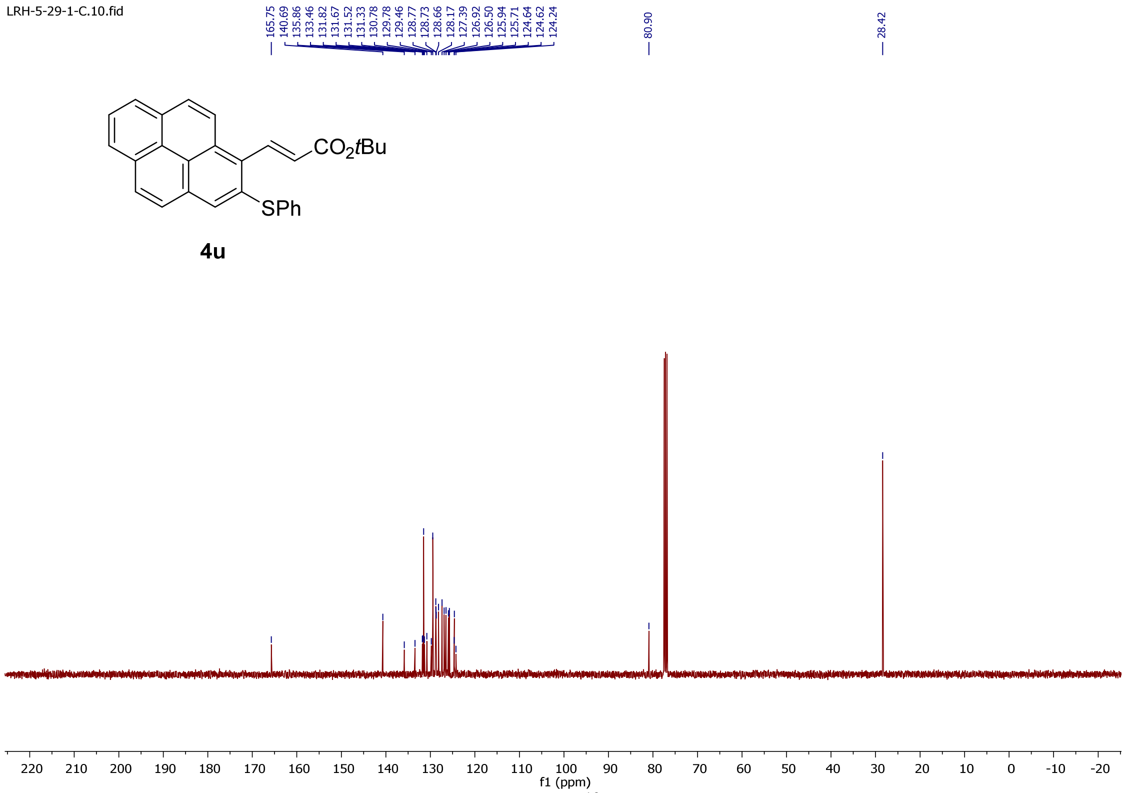

Supplementary Figure 108. ^{13}C NMR Spectrum of **4u**

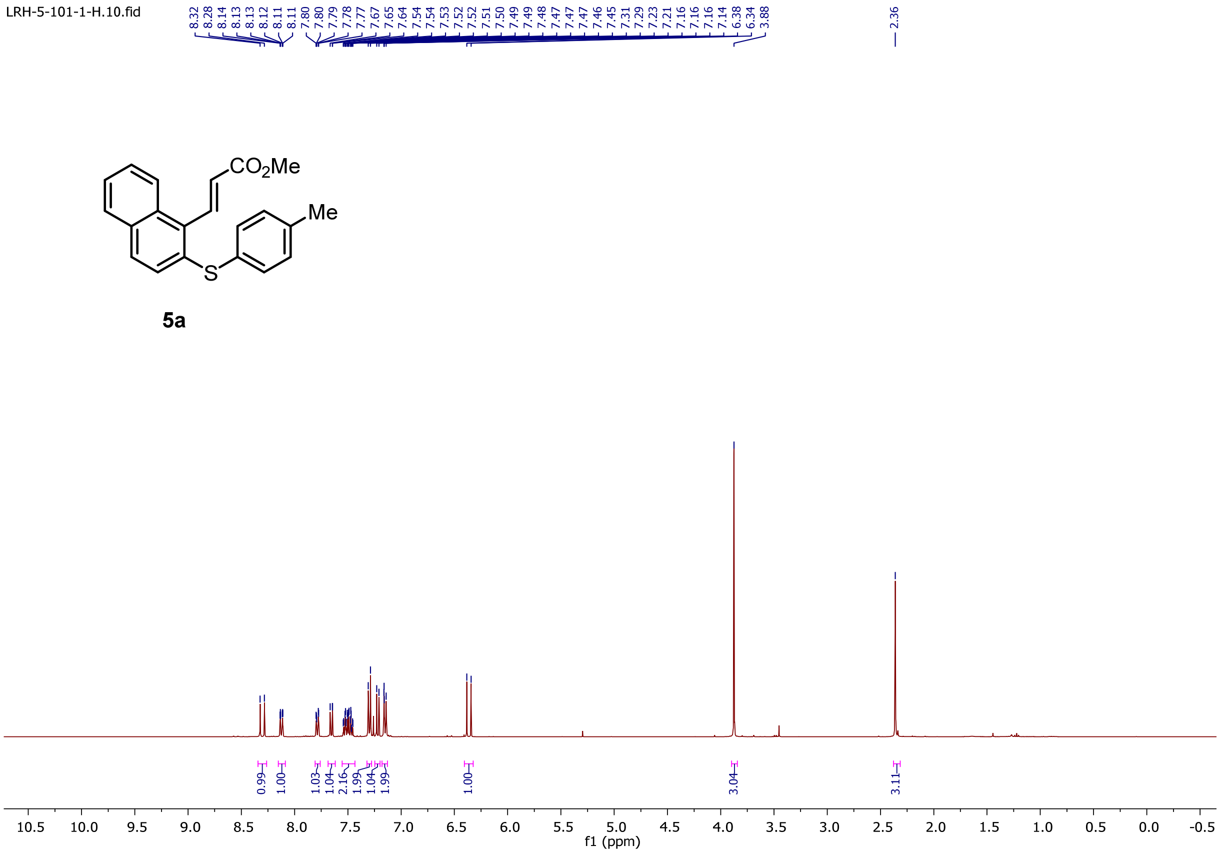

Supplementary Figure 109. ^{1}H NMR Spectrum of **5a**

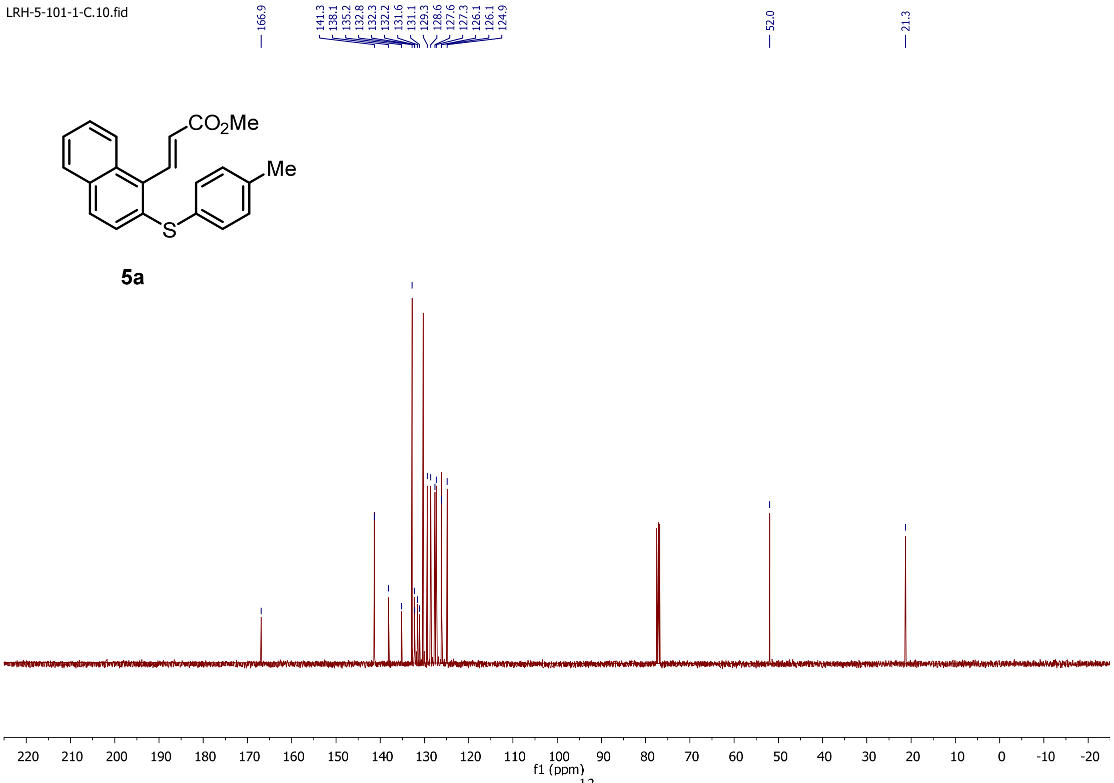

Supplementary Figure 110. ^{13}C NMR Spectrum of **5a**

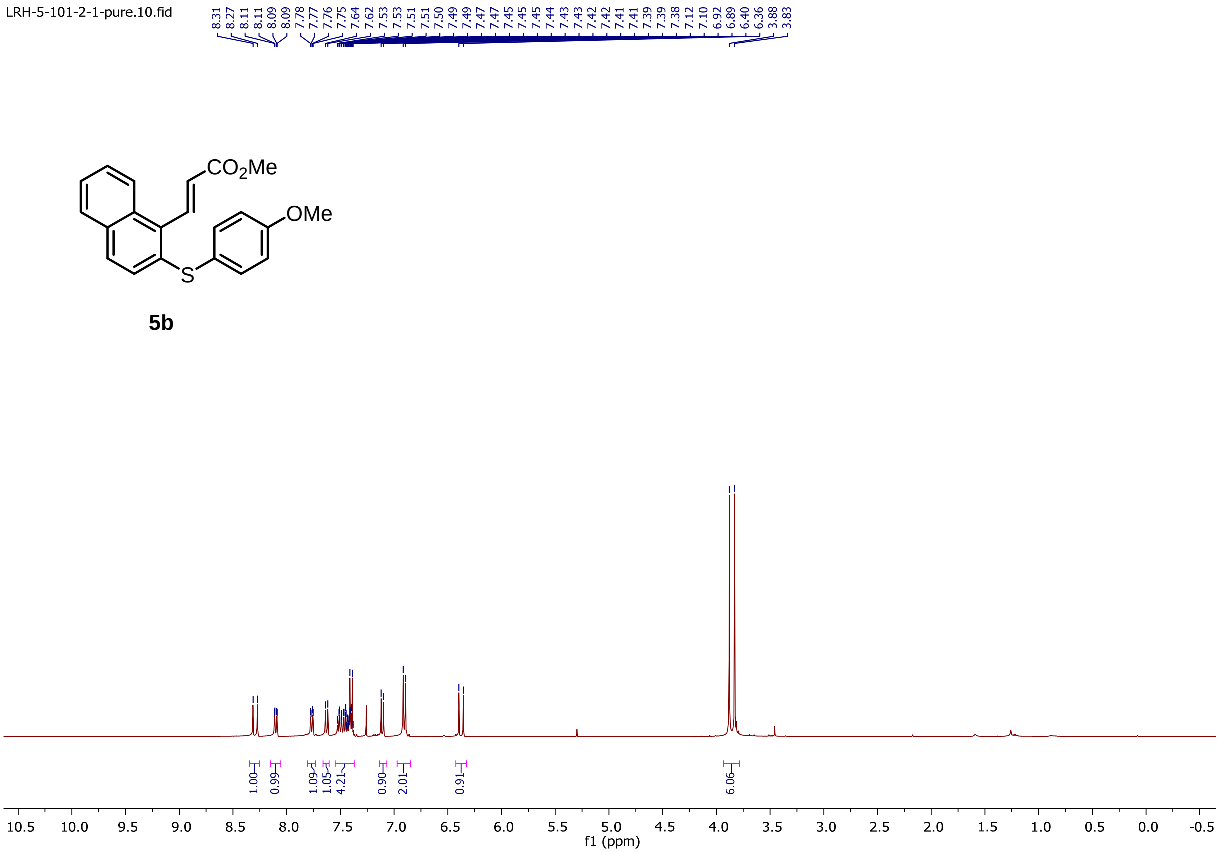

Supplementary Figure 111. ^{1}H NMR Spectrum of **5b**

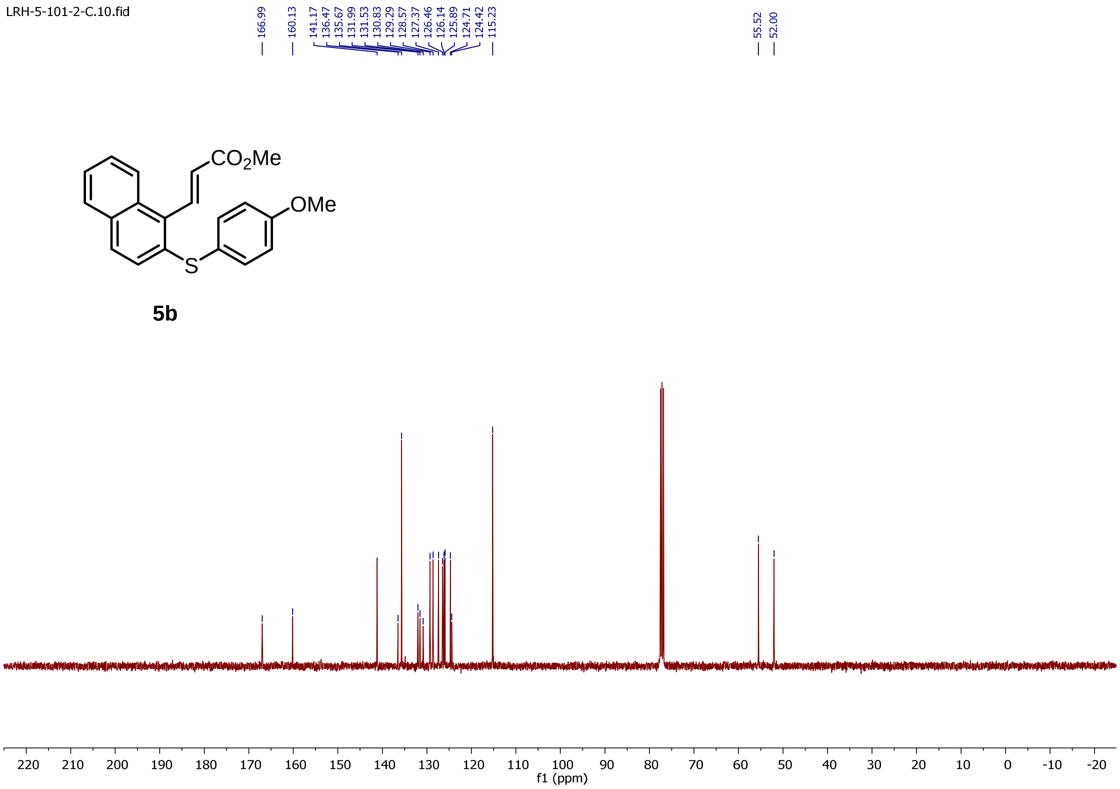

Supplementary Figure 112. ^{13}C NMR Spectrum of **5b**

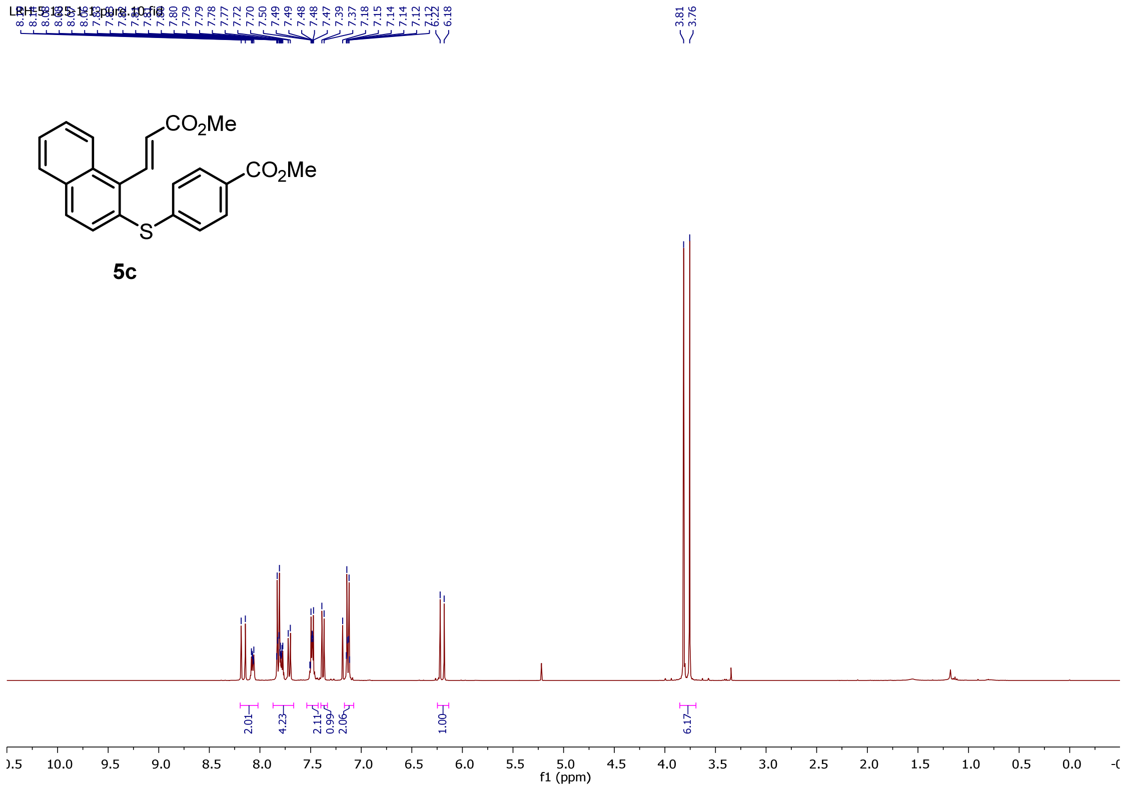

Supplementary Figure 113. ^{1}H NMR Spectrum of **5c**

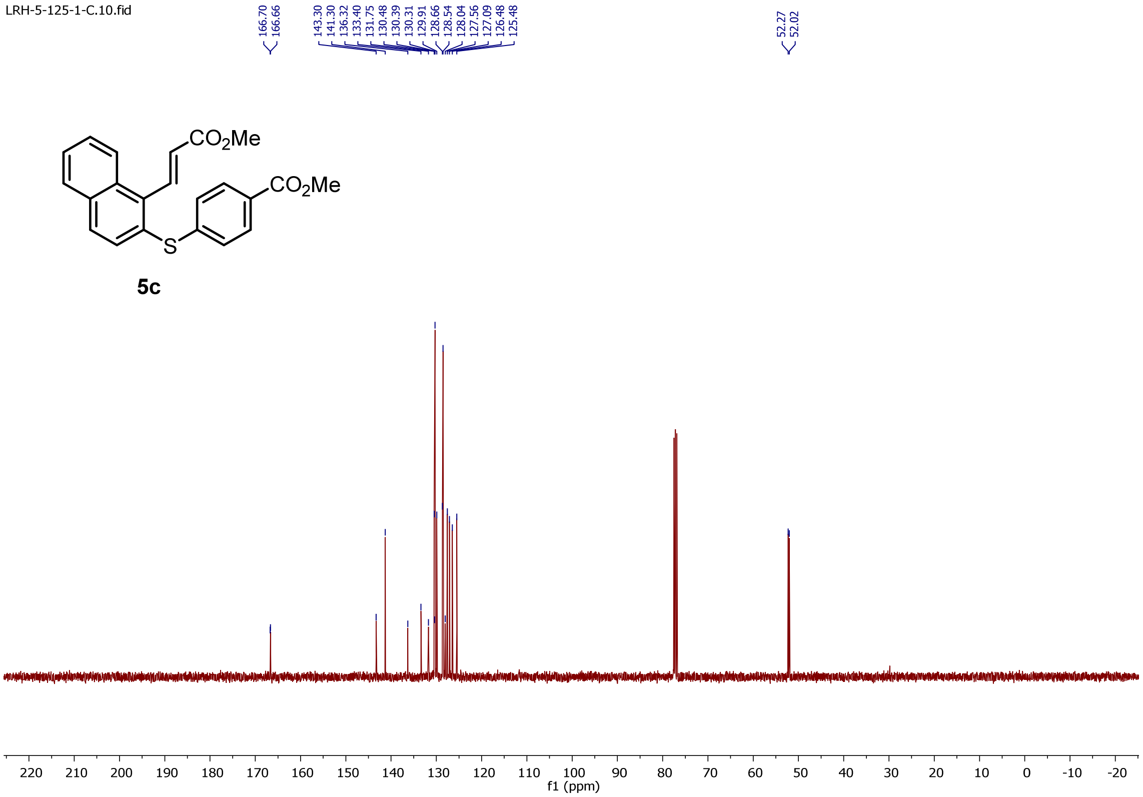

Supplementary Figure 114. ^{13}C NMR Spectrum of **5c**

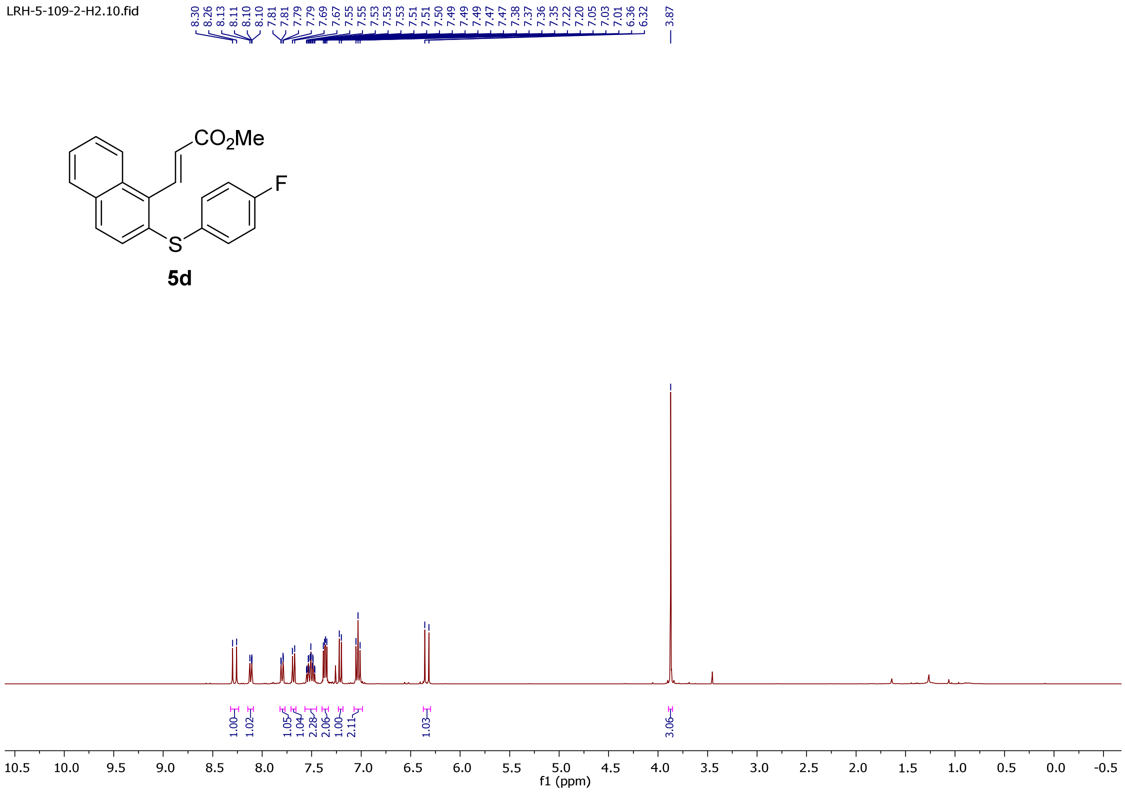

Supplementary Figure 115. ^{1}H NMR Spectrum of **5d**

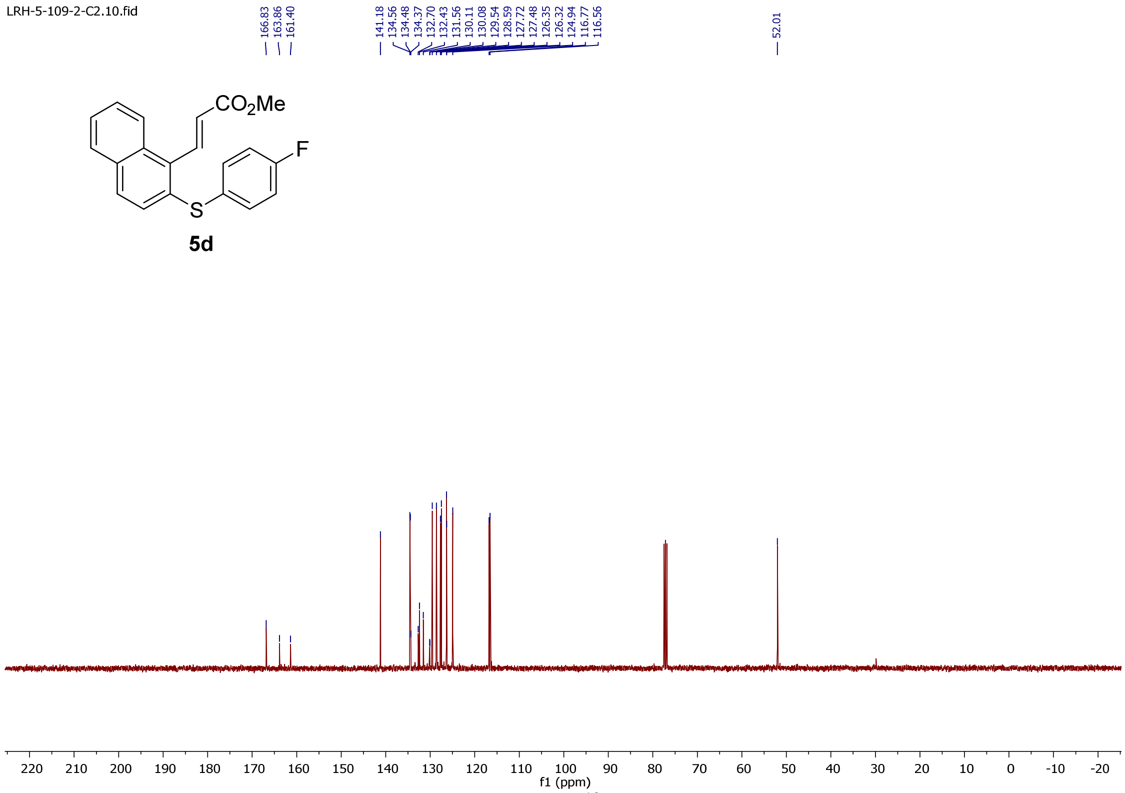

Supplementary Figure 116. ^{13}C NMR Spectrum of **5d**

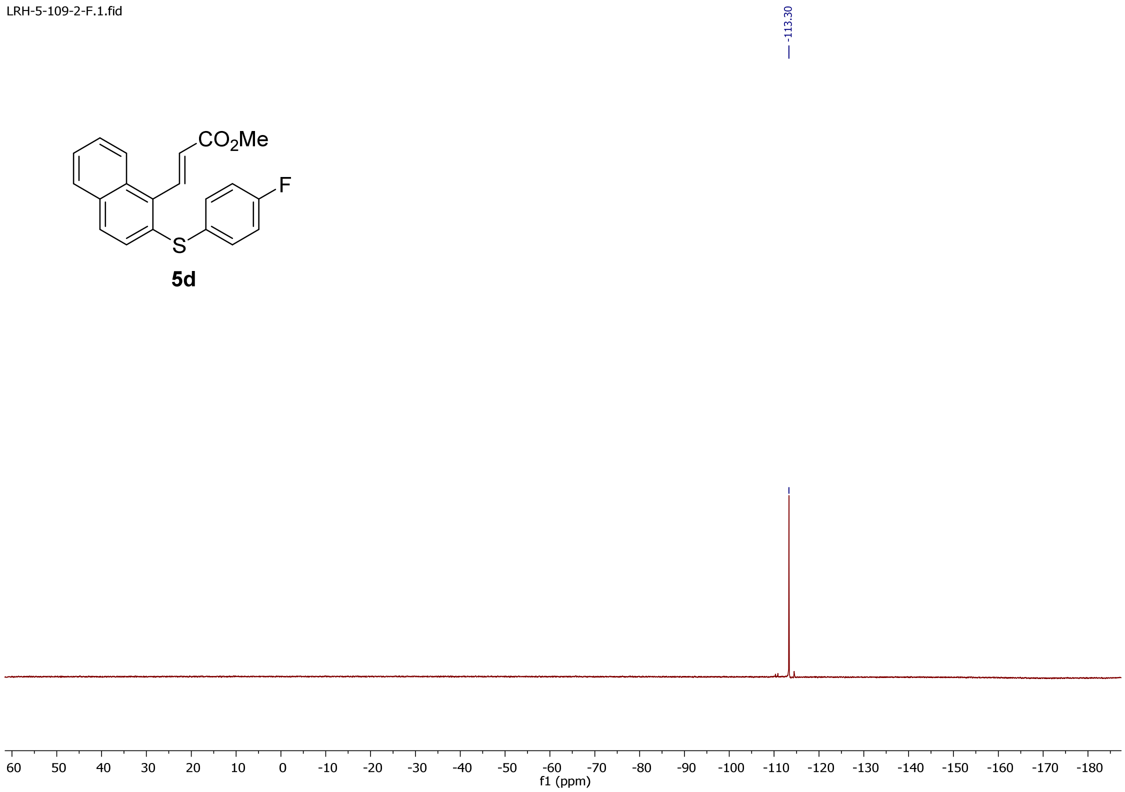

Supplementary Figure 117. ^{19}F NMR Spectrum of **5d**

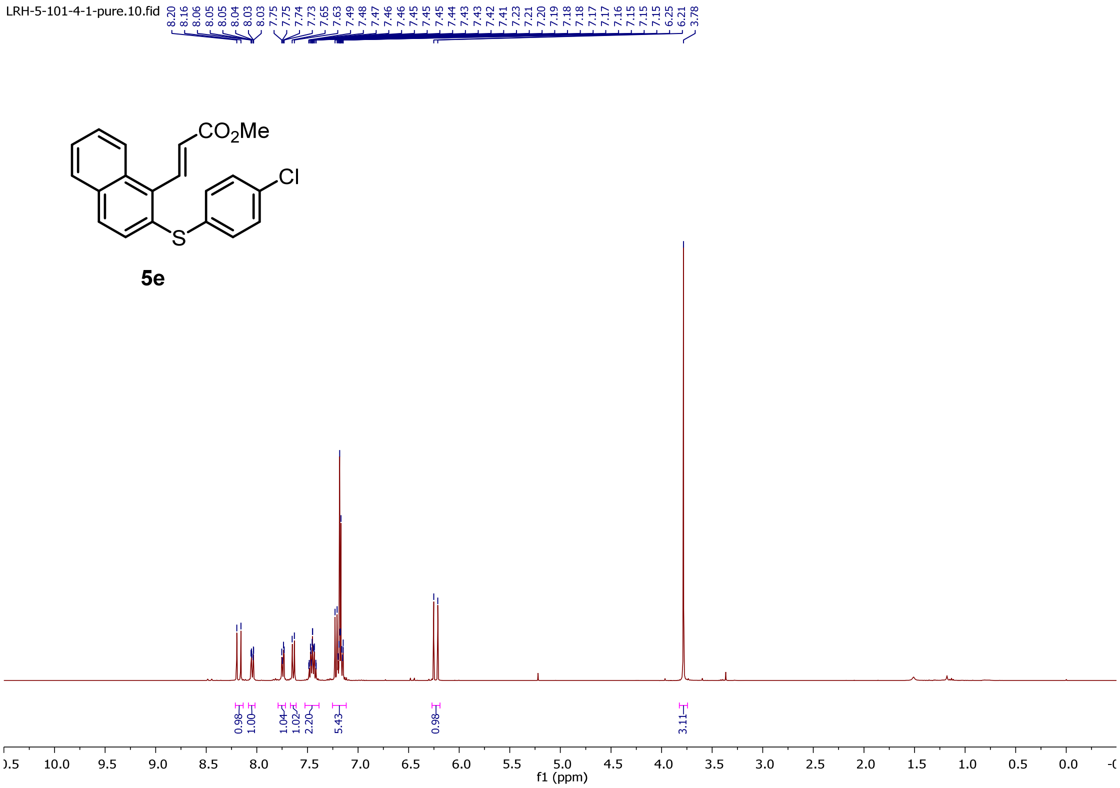

Supplementary Figure 118. ^{1}H NMR Spectrum of **5e**

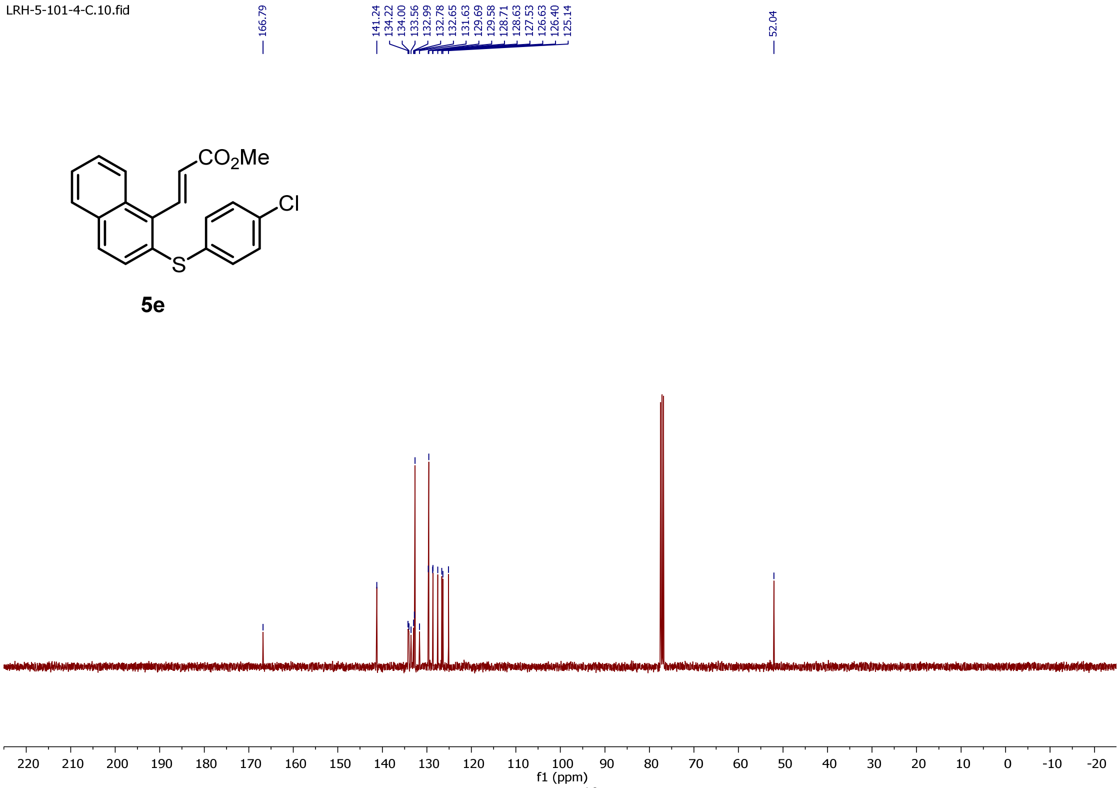

Supplementary Figure 119. ^{13}C NMR Spectrum of **5e**

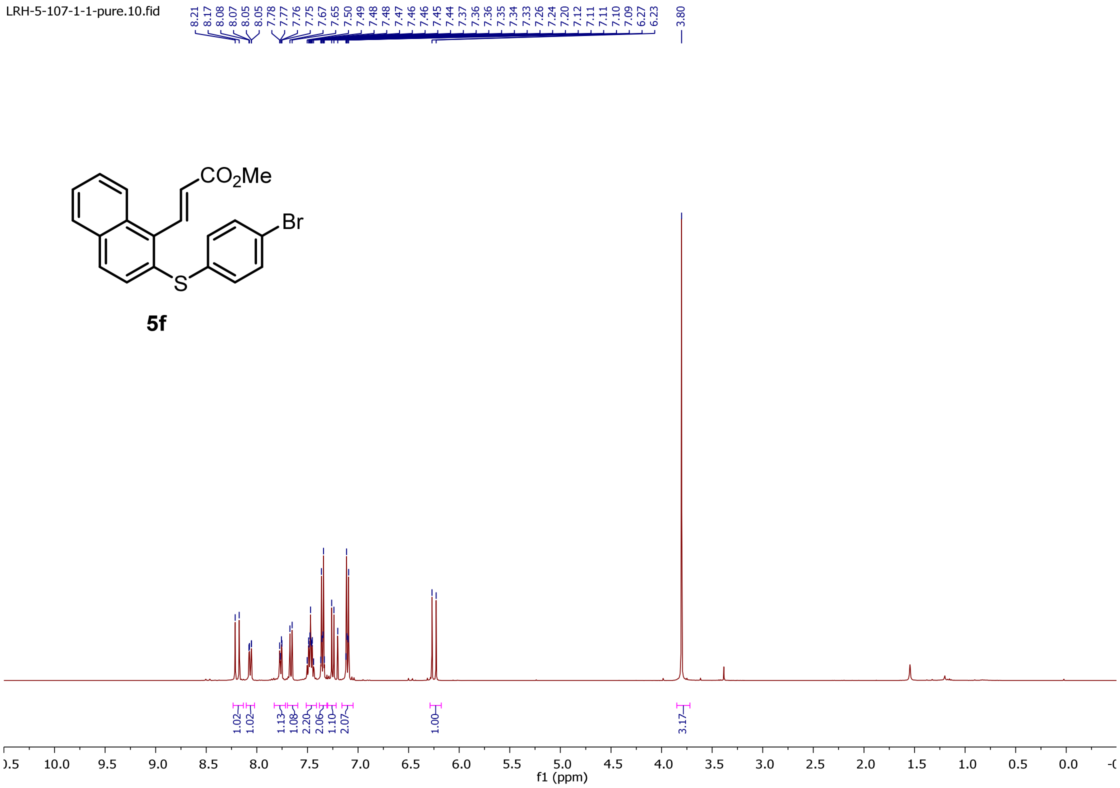

Supplementary Figure 120. ^{1}H NMR Spectrum of **5f**

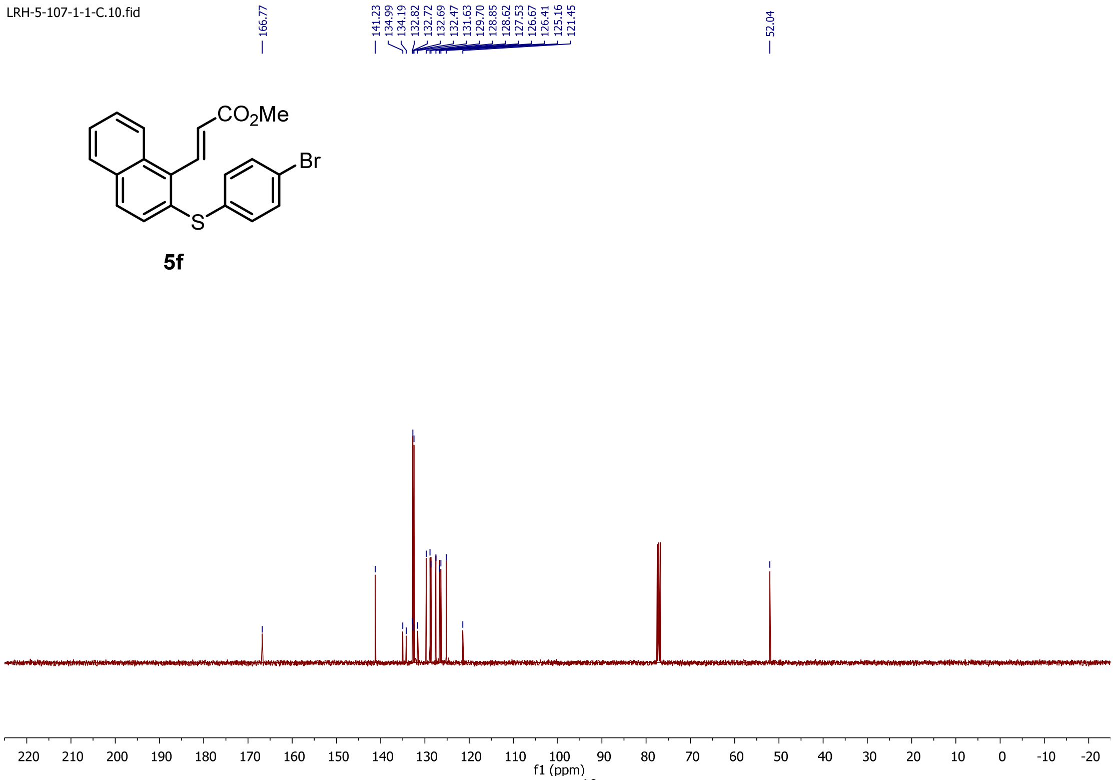

Supplementary Figure 121. ^{13}C NMR Spectrum of **5f**

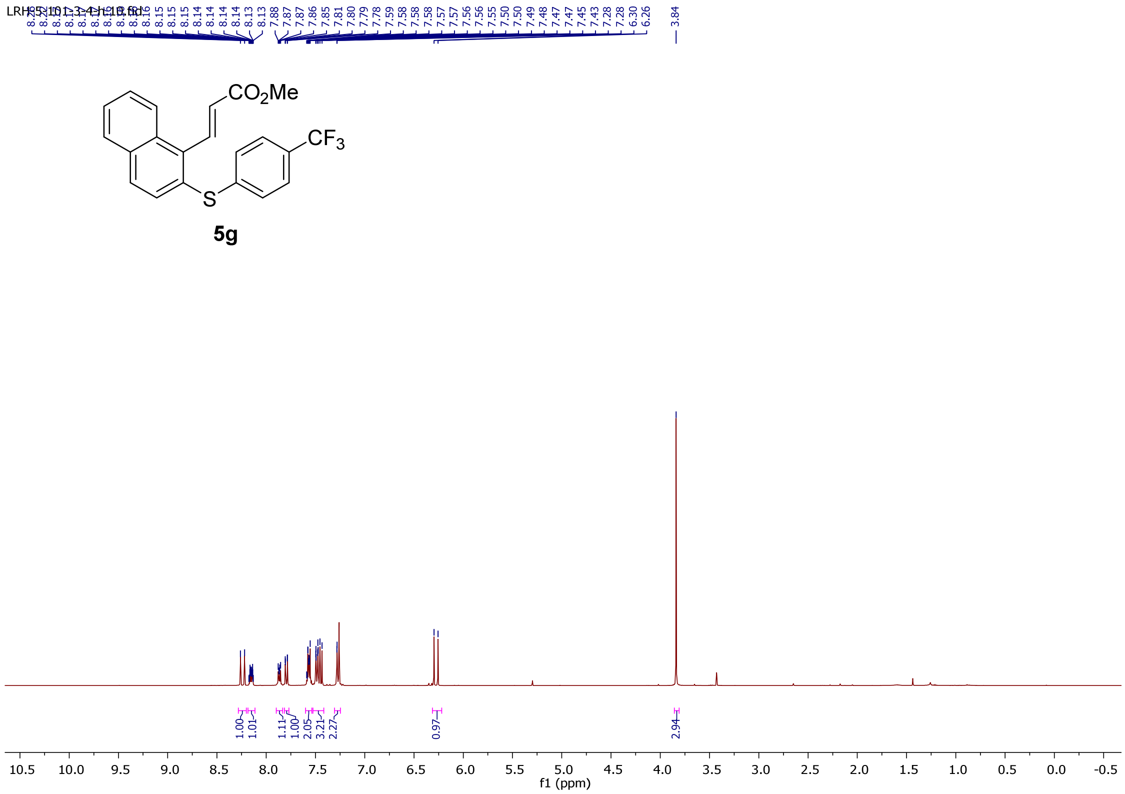

Supplementary Figure 122. ^{1}H NMR Spectrum of **5g**

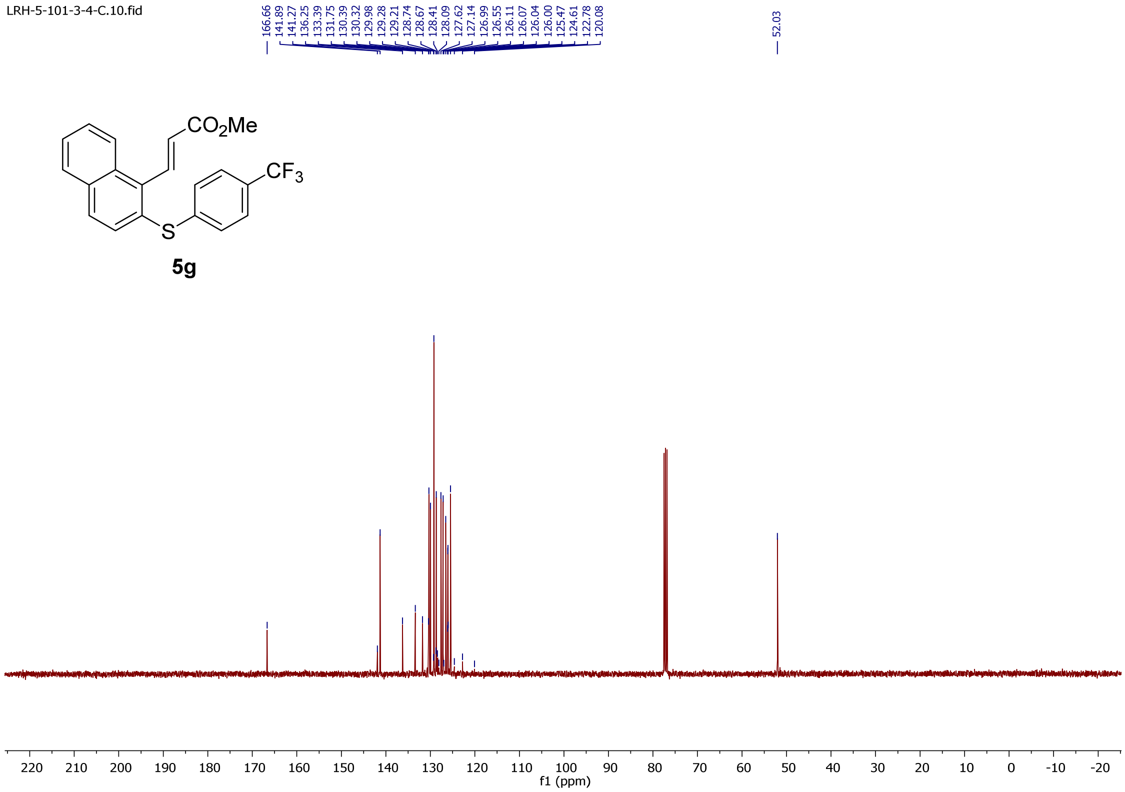

Supplementary Figure 123. ^{13}C NMR Spectrum of **5g**

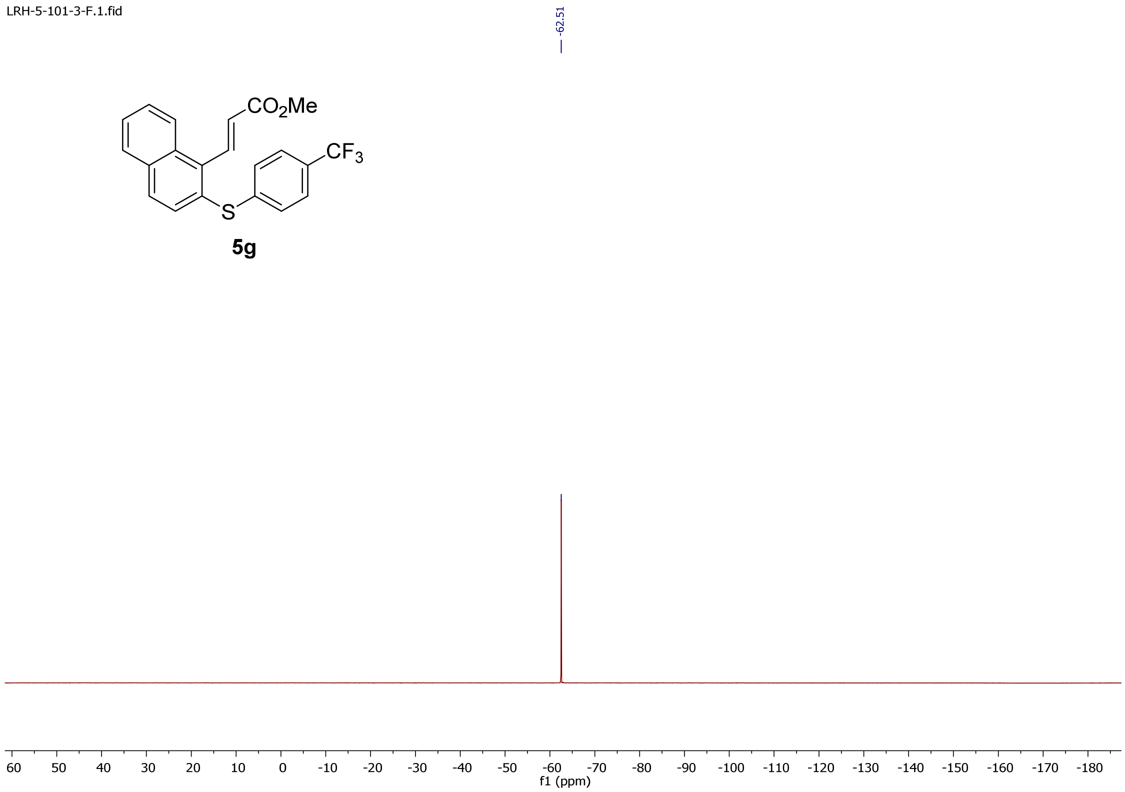

Supplementary Figure 124. ^{19}F NMR Spectrum of **5g**

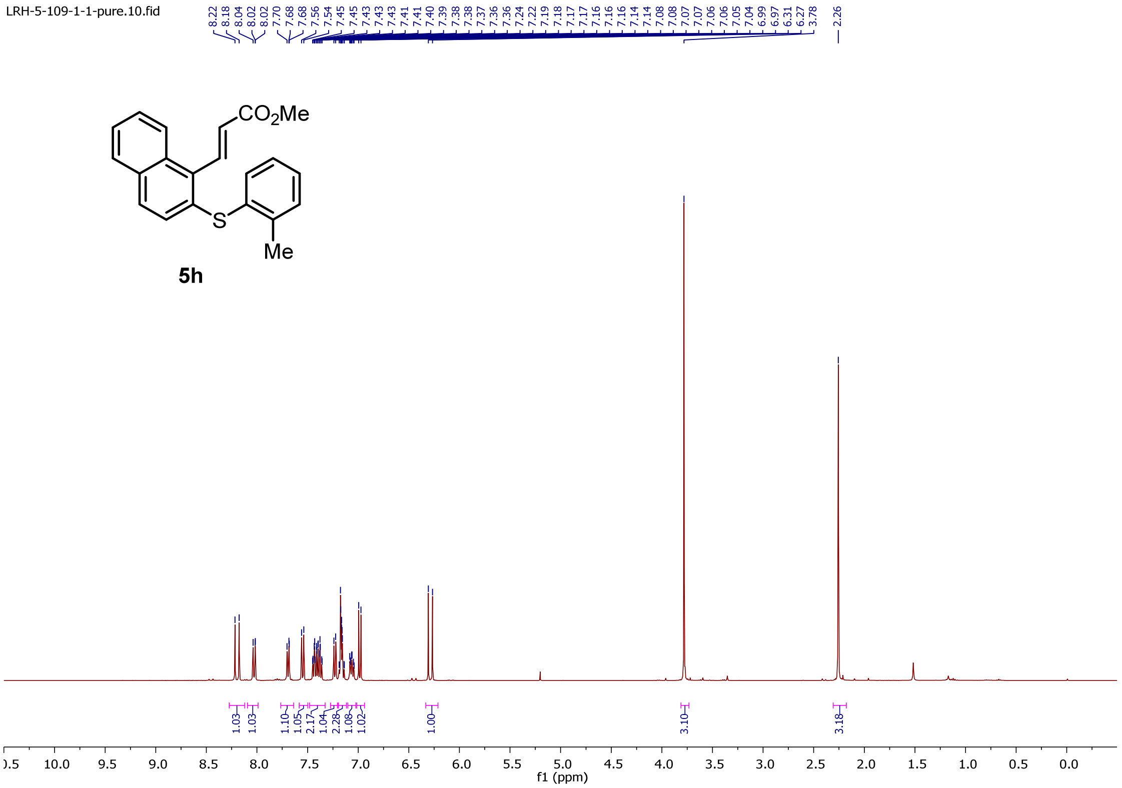

Supplementary Figure 125. ^{1}H NMR Spectrum of **5h**

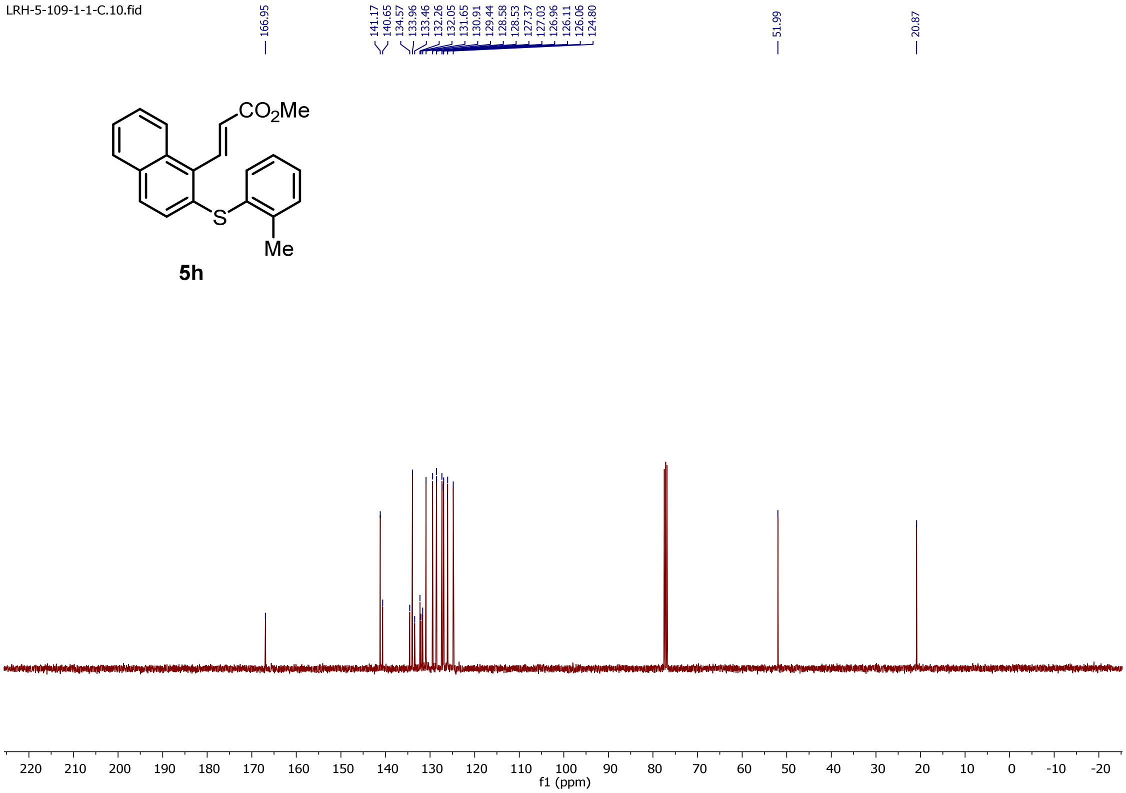

Supplementary Figure 126. ^{13}C NMR Spectrum of **5h**

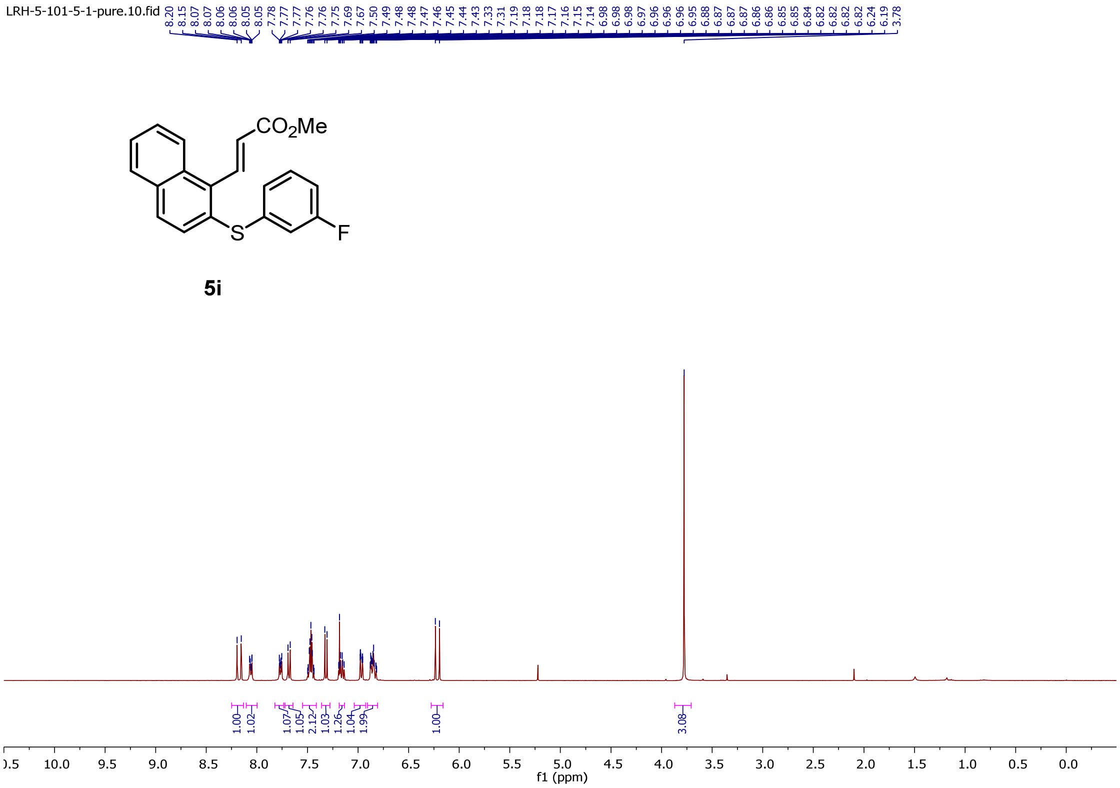

Supplementary Figure 127. ^{1}H NMR Spectrum of **5i**

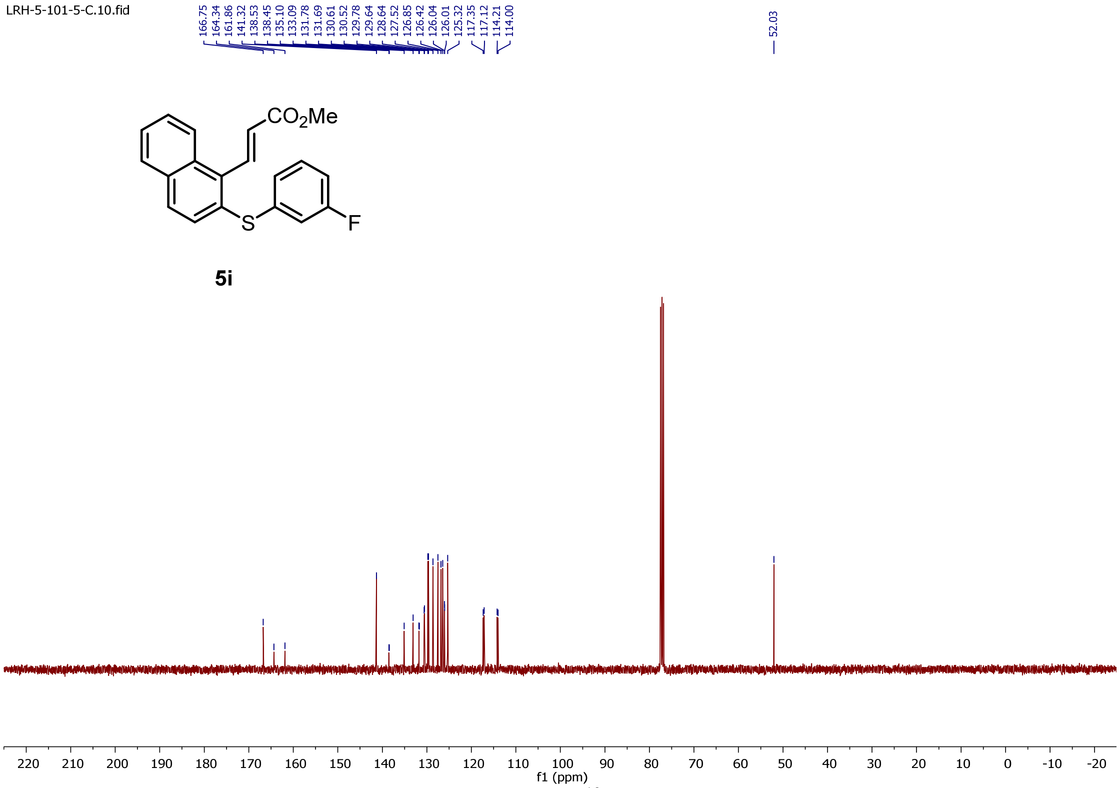

Supplementary Figure 128. ^{13}C NMR Spectrum of **5i**

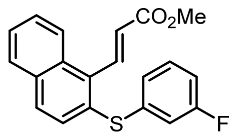

5i

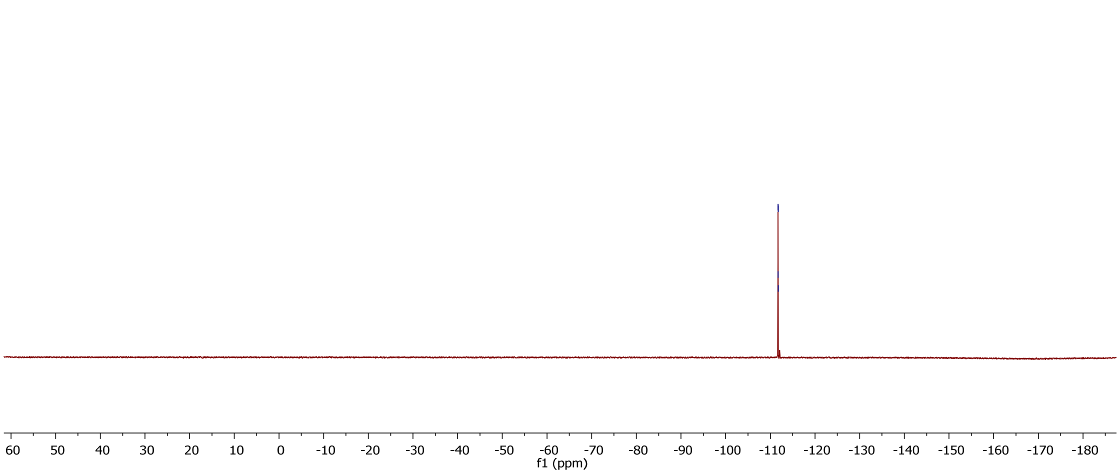

Supplementary Figure 129. ^{19}F NMR Spectrum of **5i**

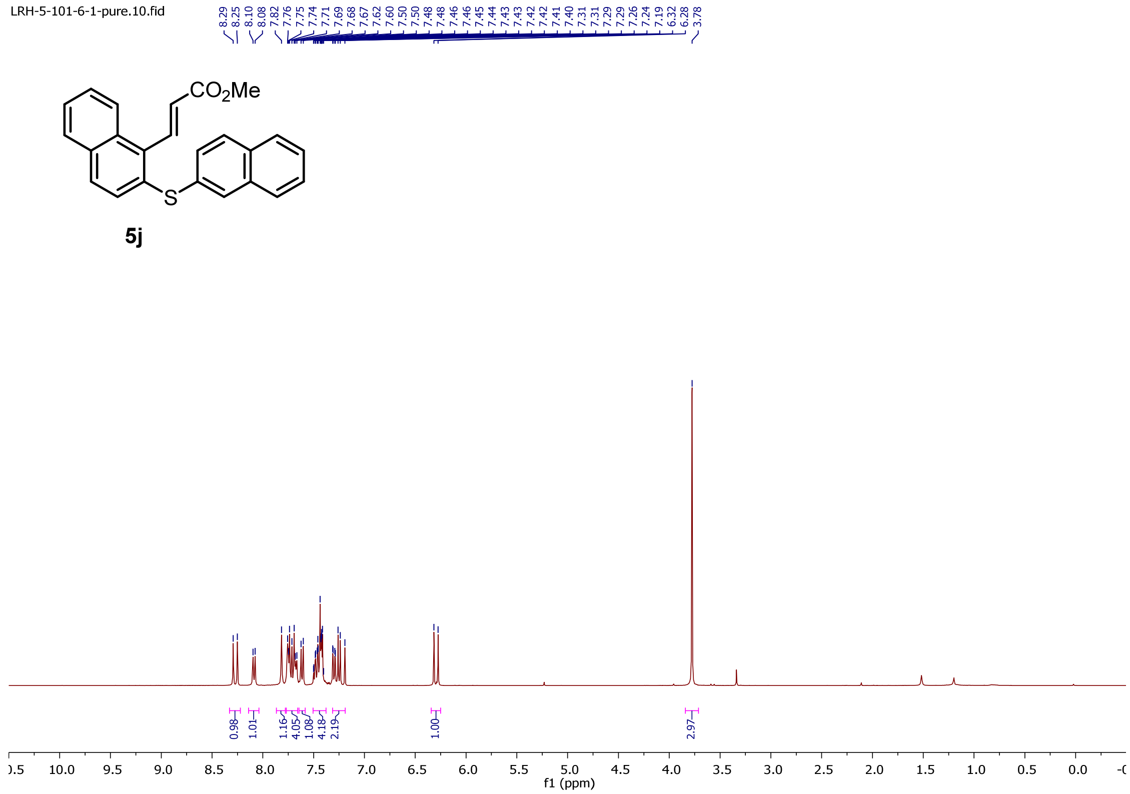

Supplementary Figure 130. ^{1}H NMR Spectrum of **5j**

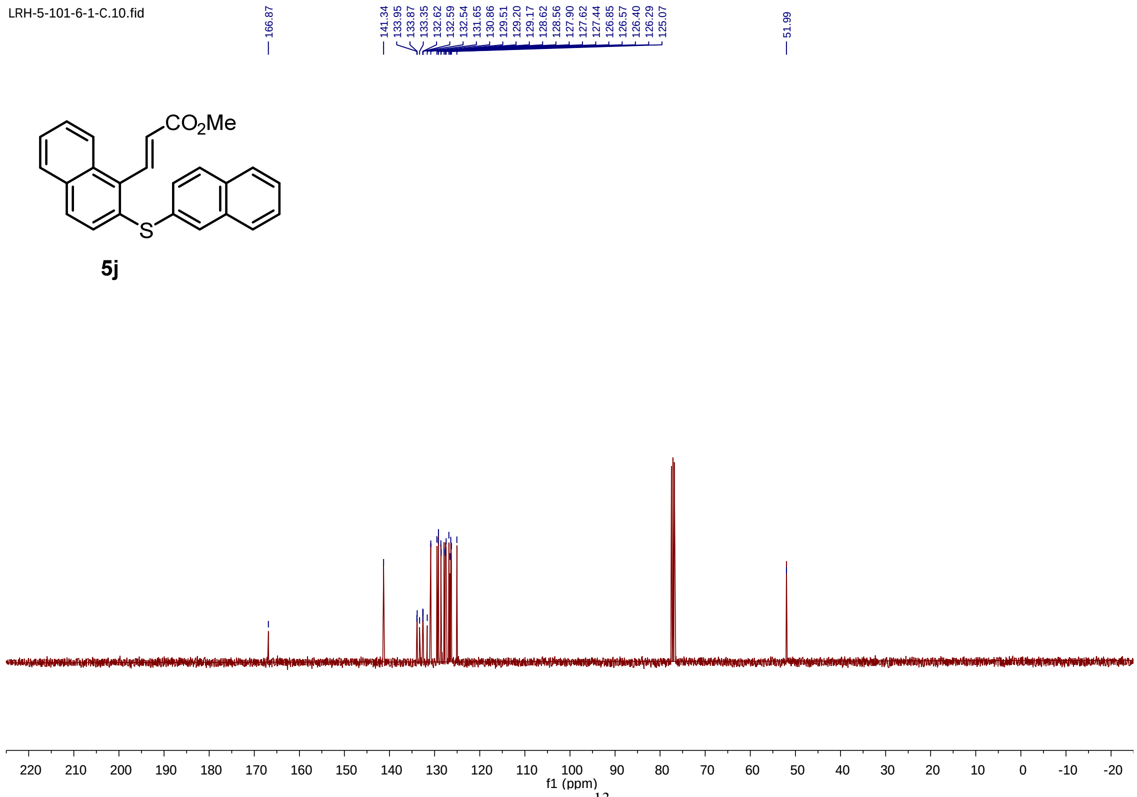

Supplementary Figure 131. ^{13}C NMR Spectrum of **5j**

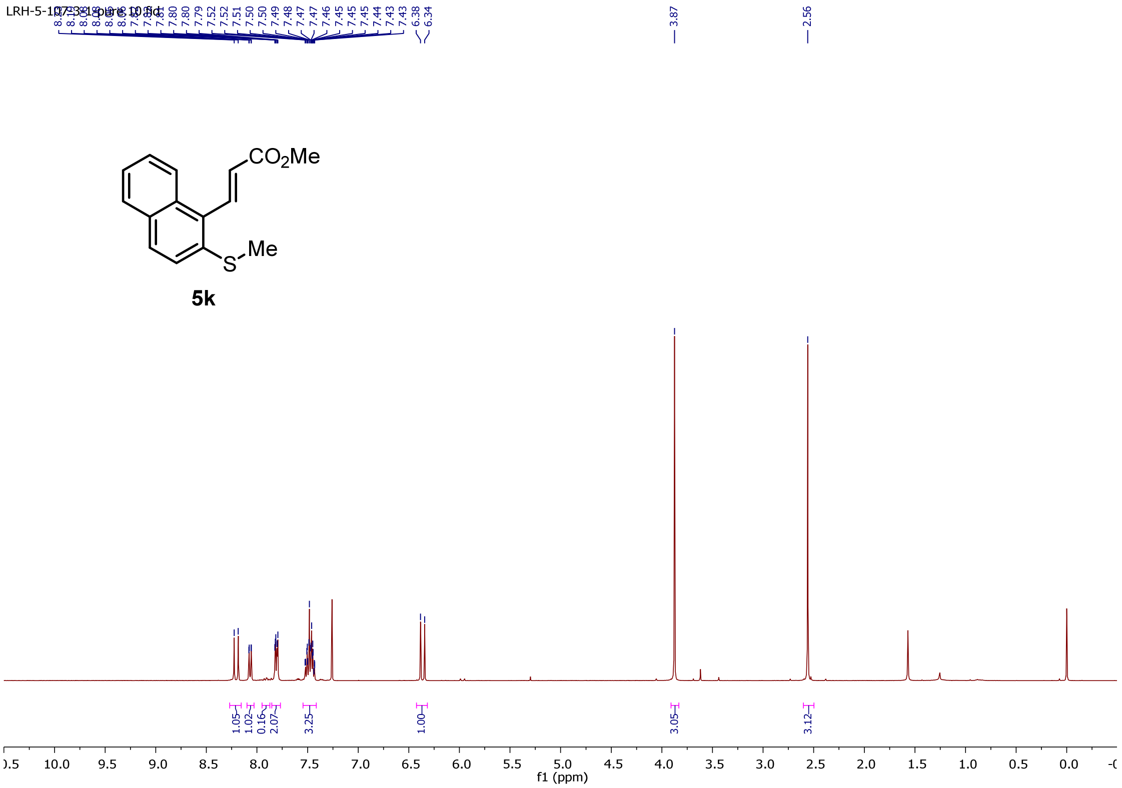

Supplementary Figure 132. ^{1}H NMR Spectrum of **5k**

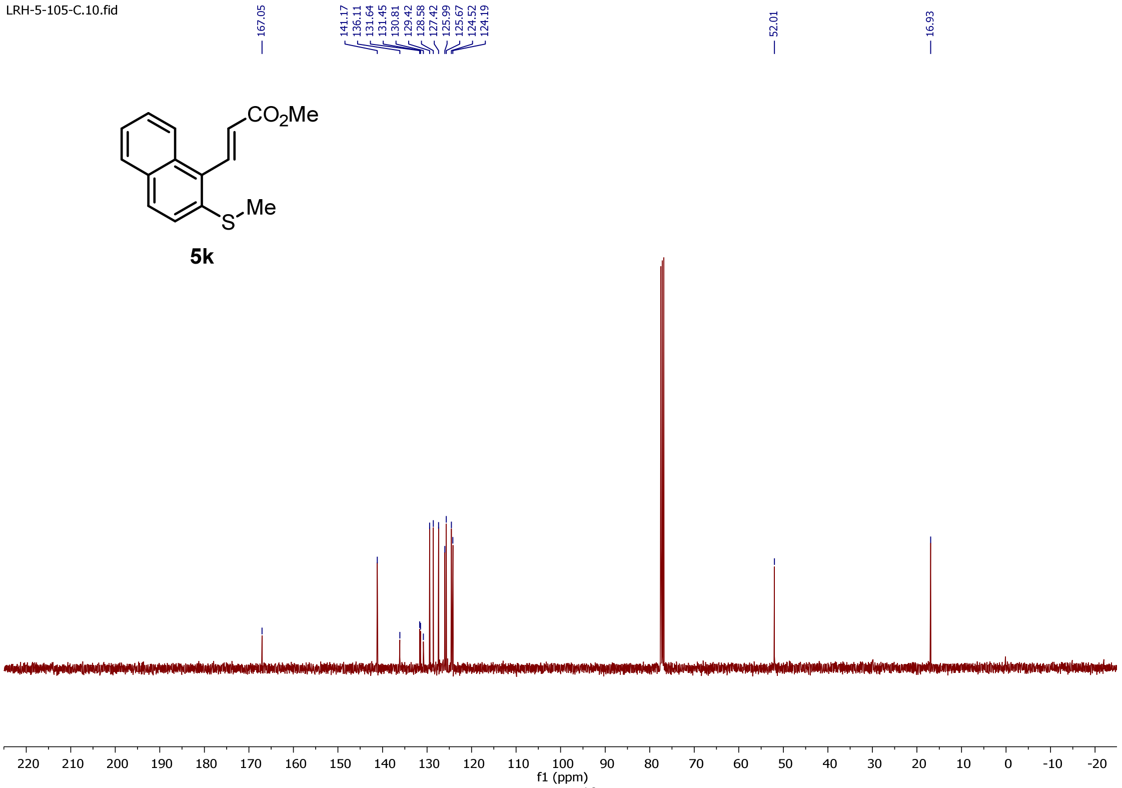

Supplementary Figure 133. ^{13}C NMR Spectrum of **5k**

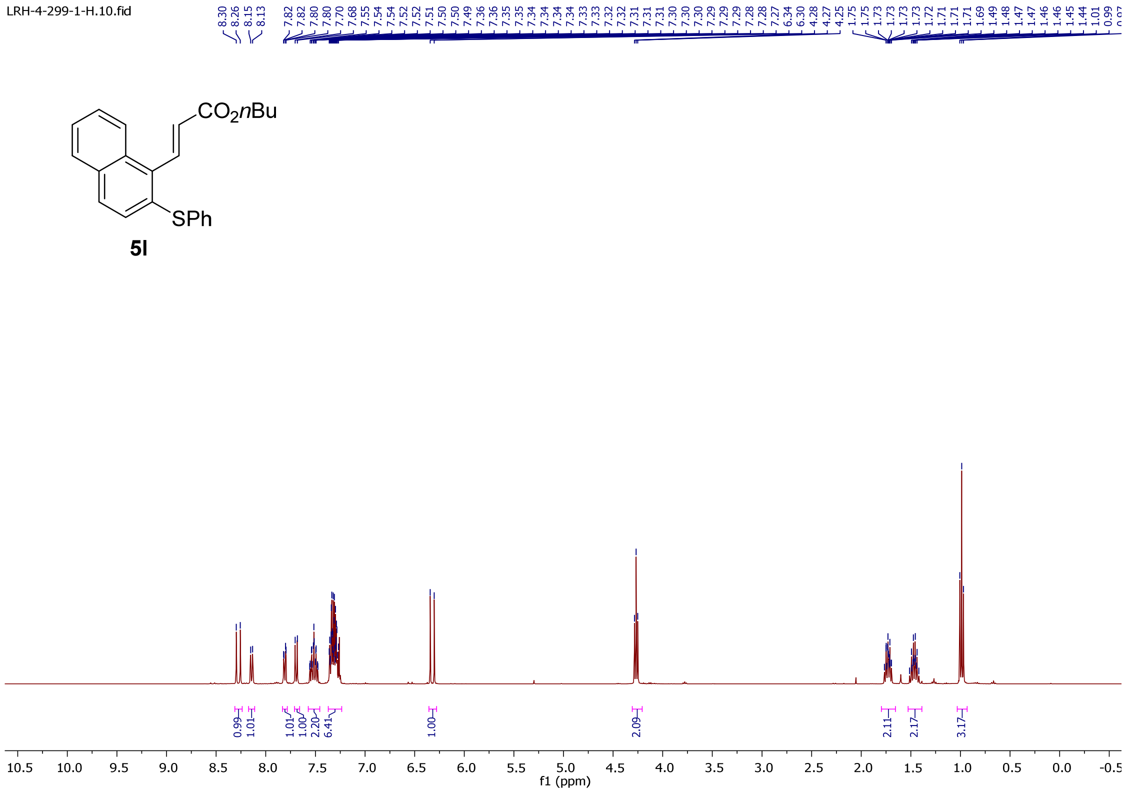

Supplementary Figure 134. ^{1}H NMR Spectrum of **5l**

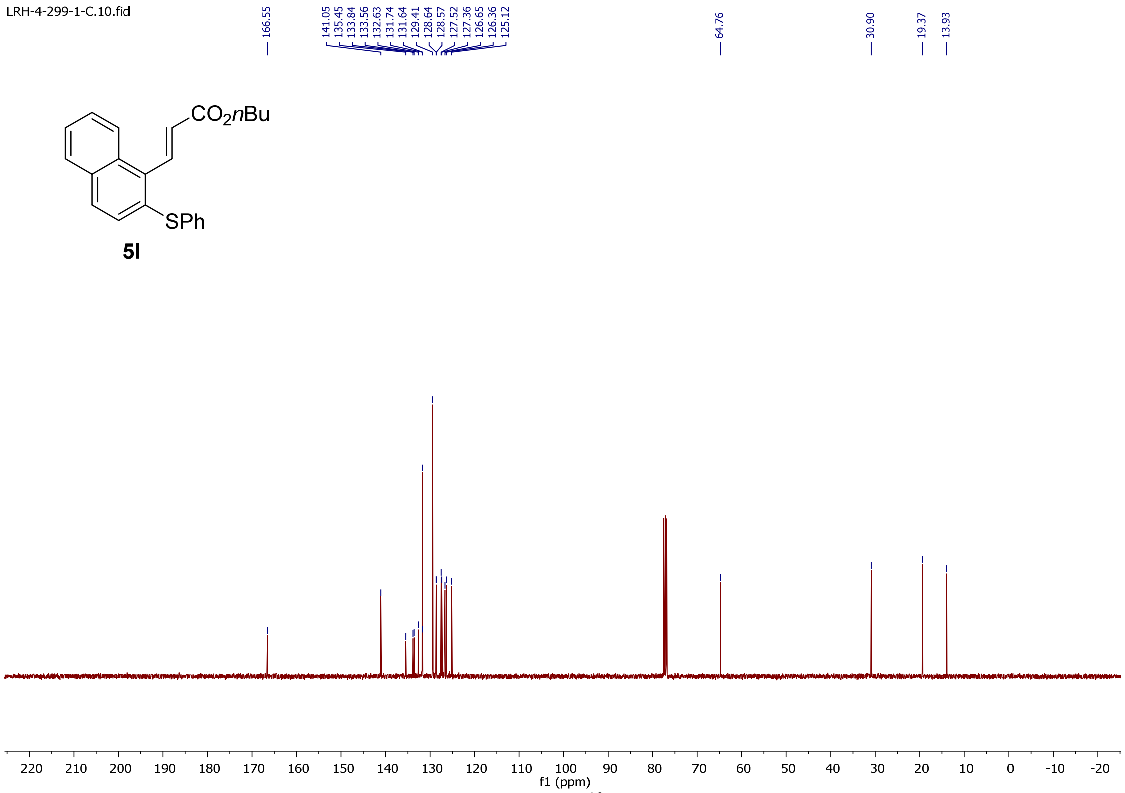

Supplementary Figure 135. ^{13}C NMR Spectrum of **5l**

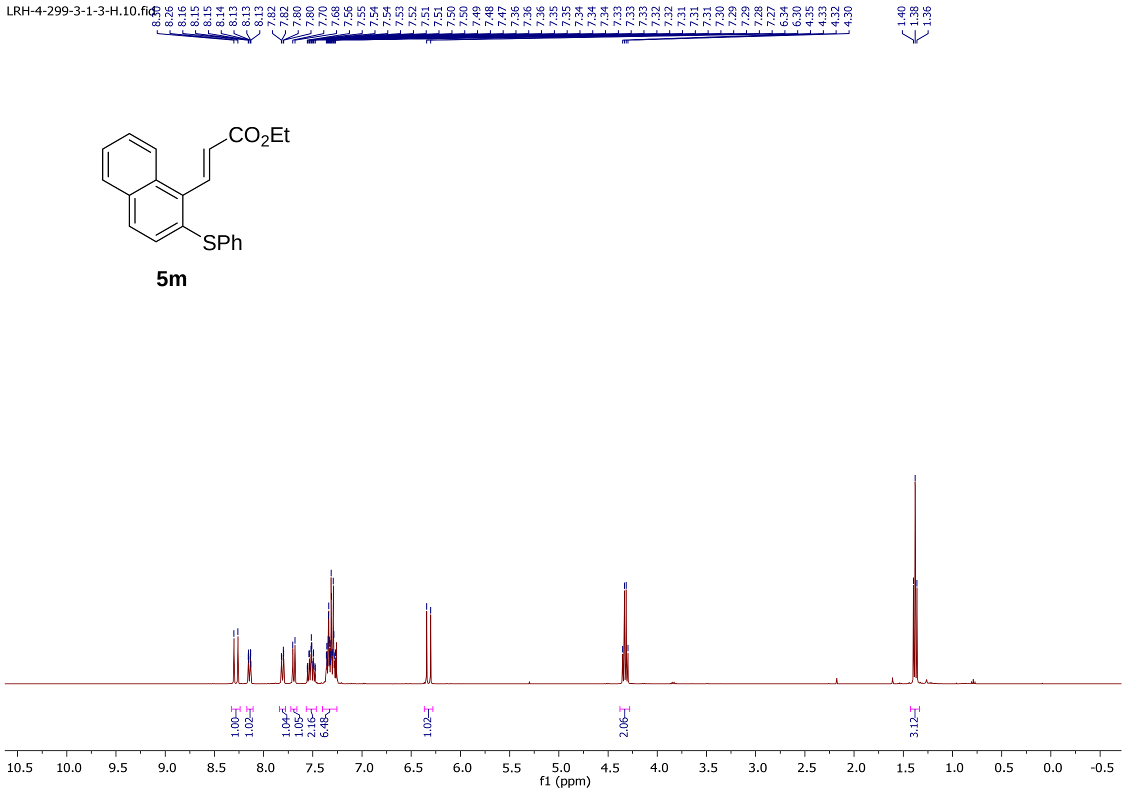

Supplementary Figure 136. ^{1}H NMR Spectrum of **5m**

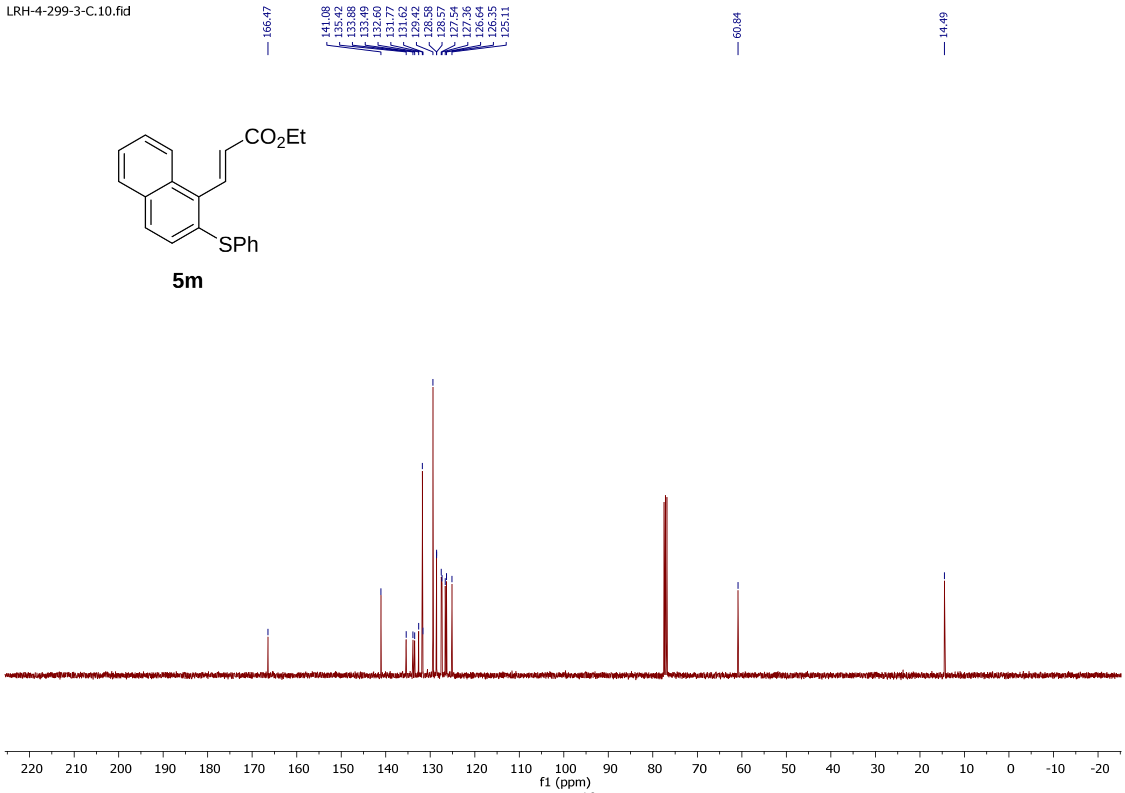

Supplementary Figure 137. ^{13}C NMR Spectrum of **5m**

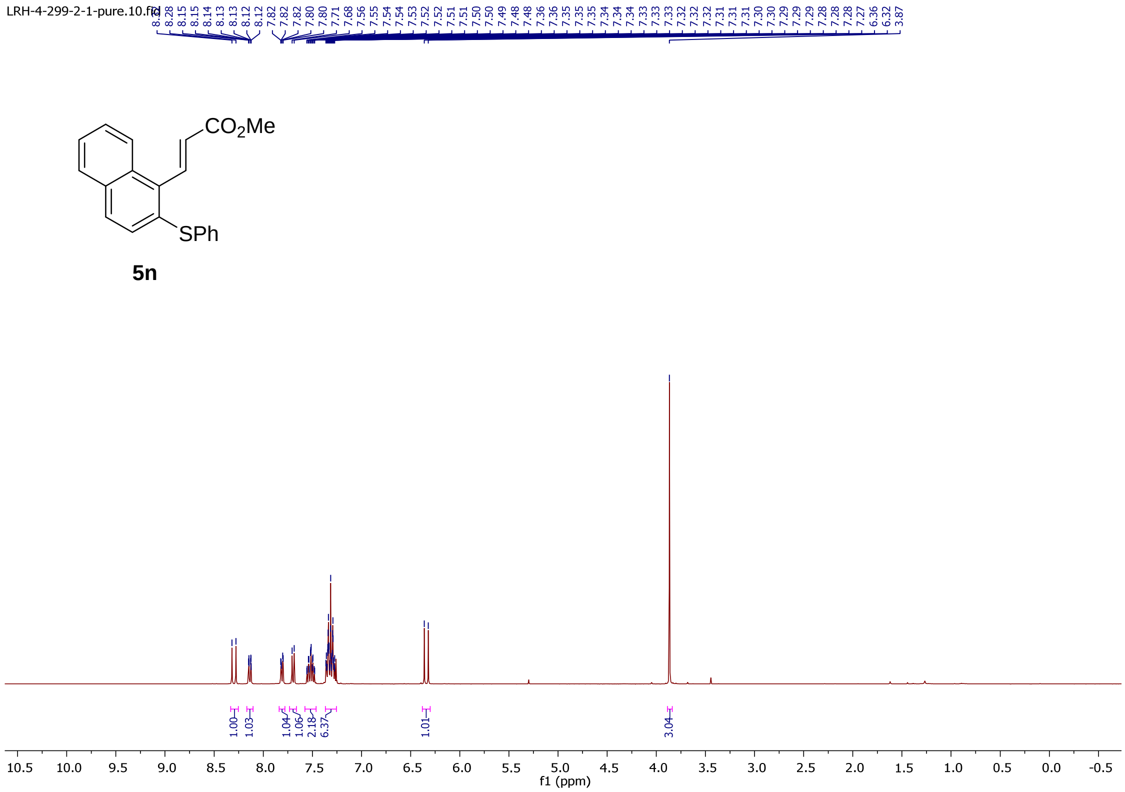

Supplementary Figure 138. ^{1}H NMR Spectrum of **5n**

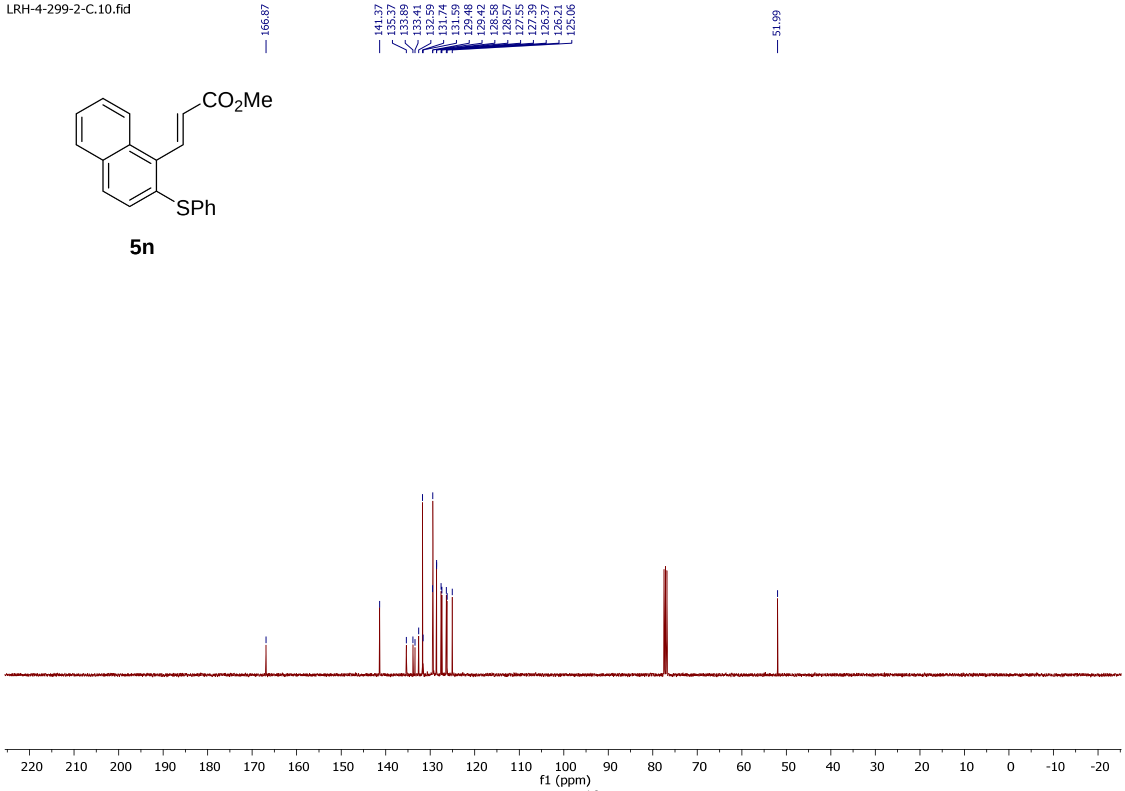

Supplementary Figure 139. ^{13}C NMR Spectrum of **5n**

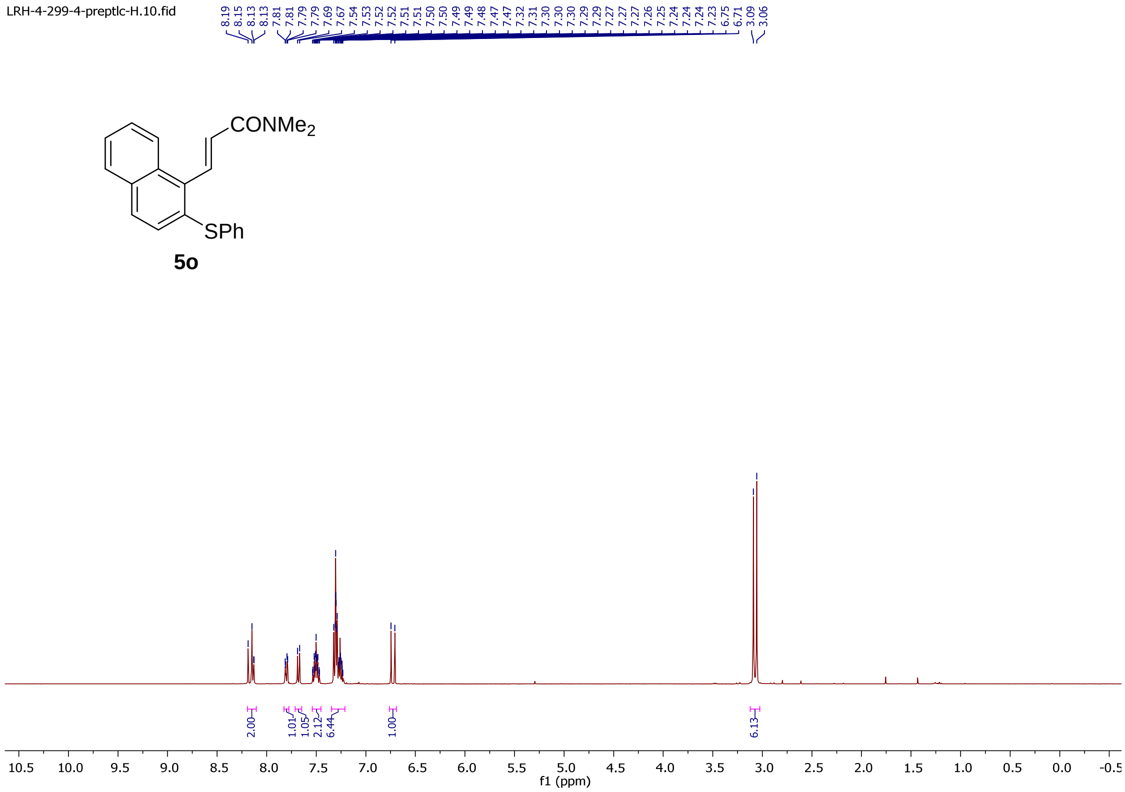

Supplementary Figure 140. ^{1}H NMR Spectrum of **5o**

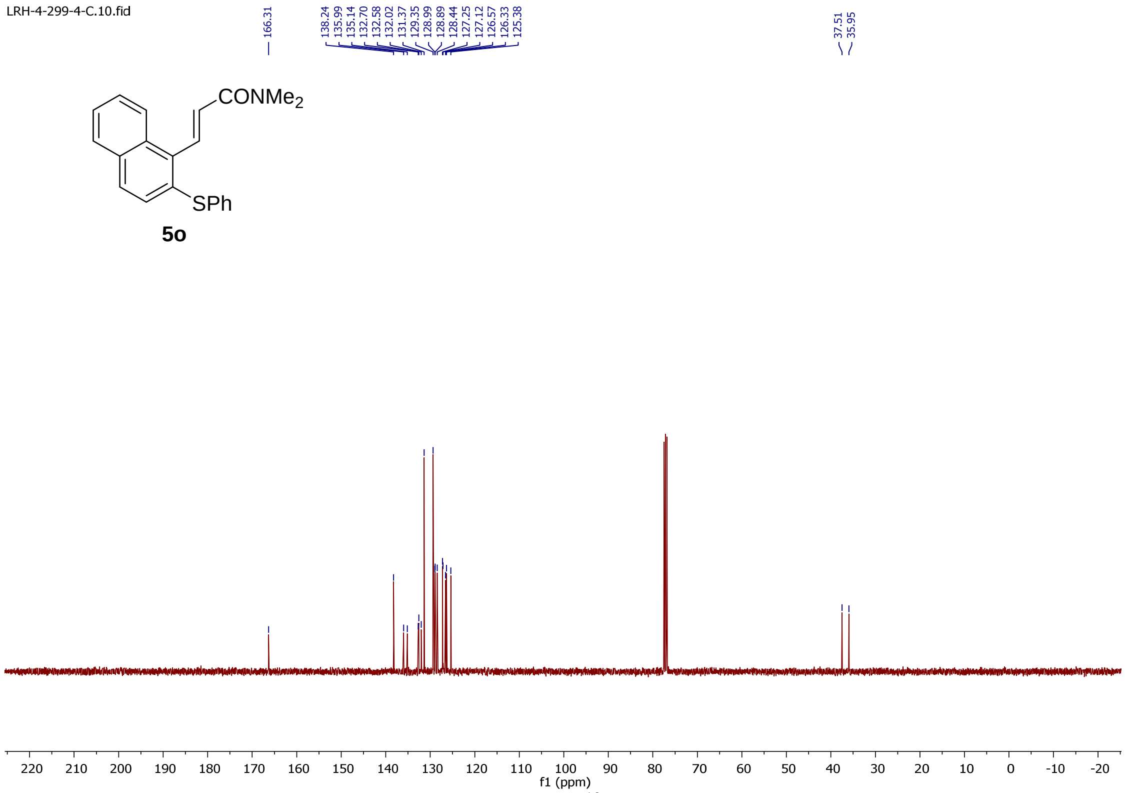

Supplementary Figure 141. ^{13}C NMR Spectrum of **5o**

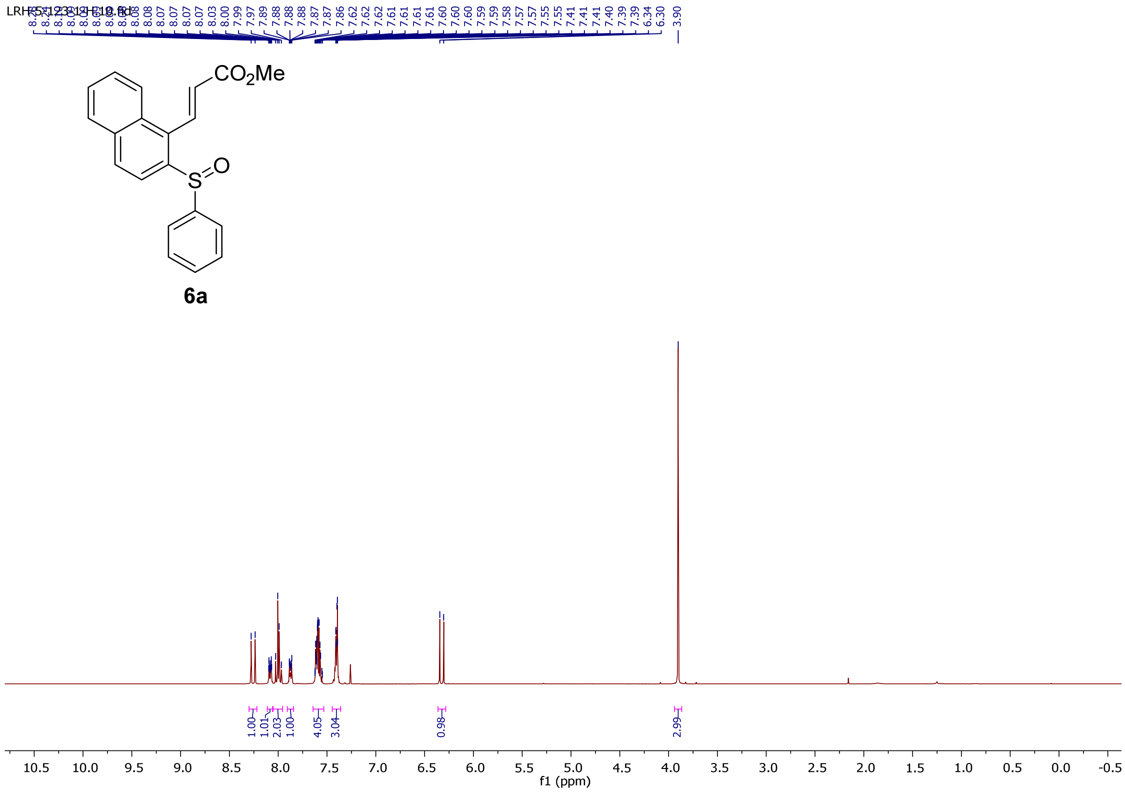

Supplementary Figure 142. ^{1}H NMR Spectrum of **6a**

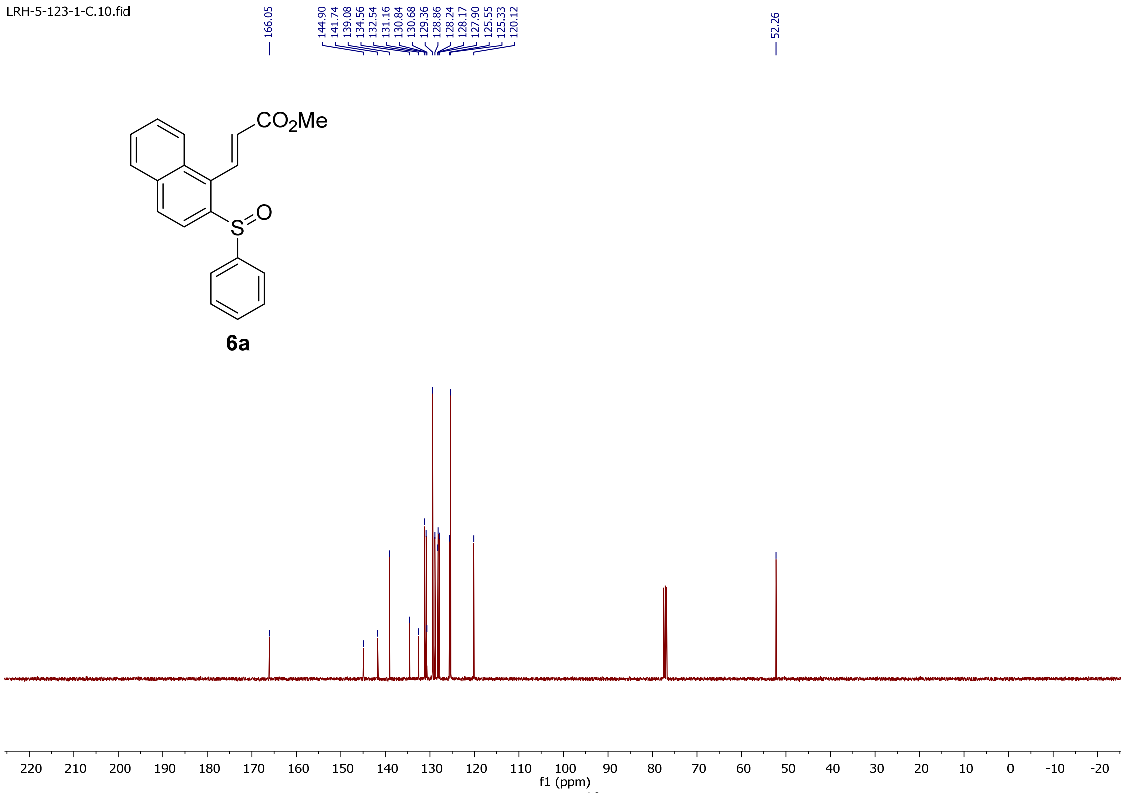

Supplementary Figure 143. ^{13}C NMR Spectrum of **6a**

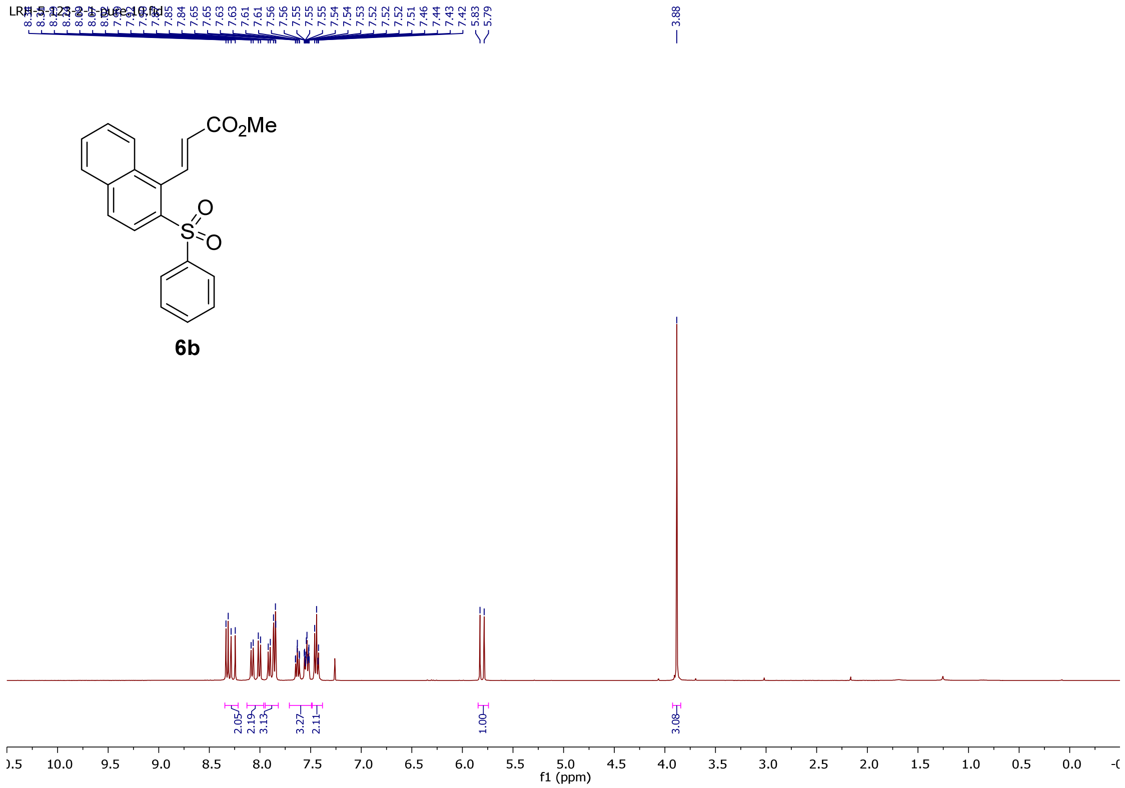

Supplementary Figure 144. ^{1}H NMR Spectrum of **6b**

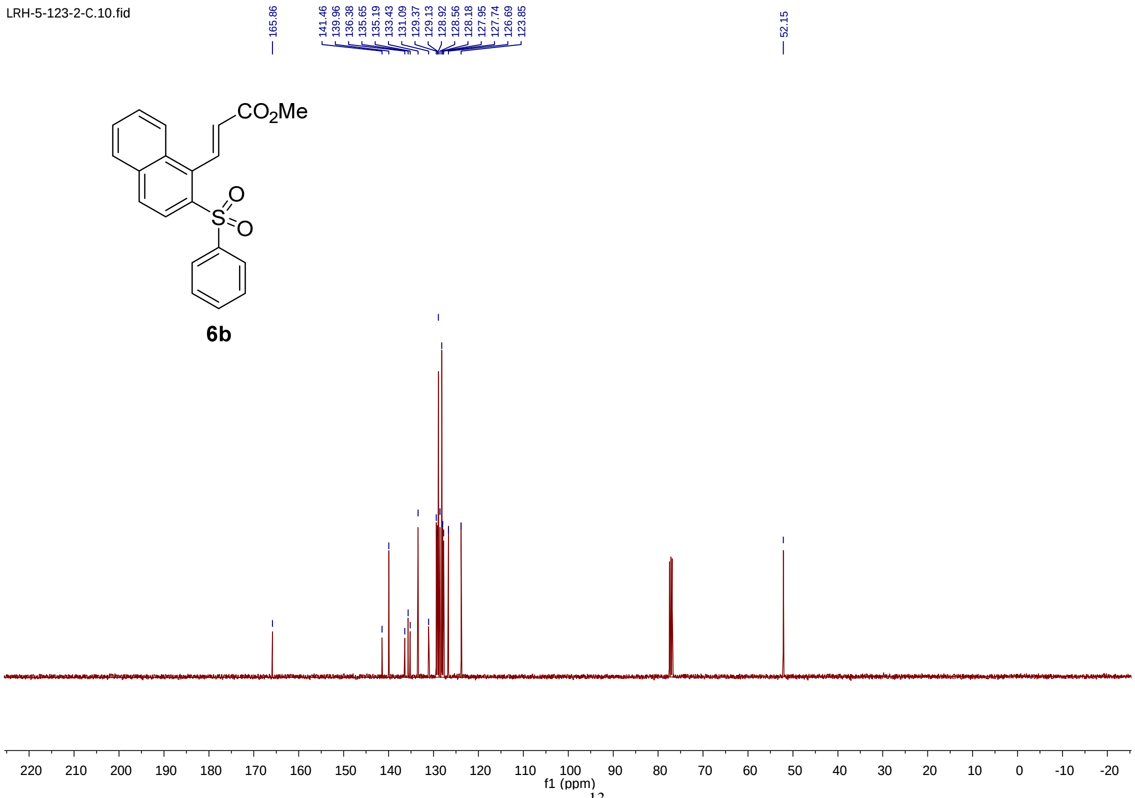

Supplementary Figure 145. ^{13}C NMR Spectrum of **6b**

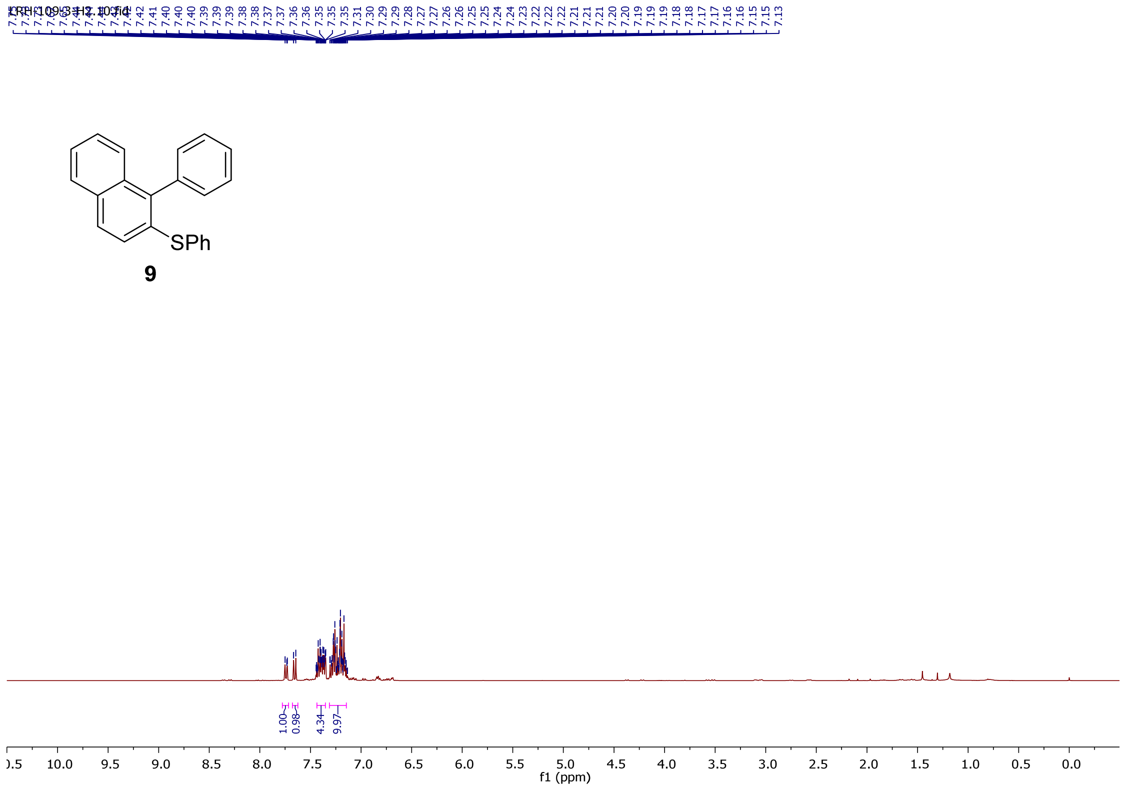

Supplementary Figure 146. ^{1}H NMR Spectrum of **9**

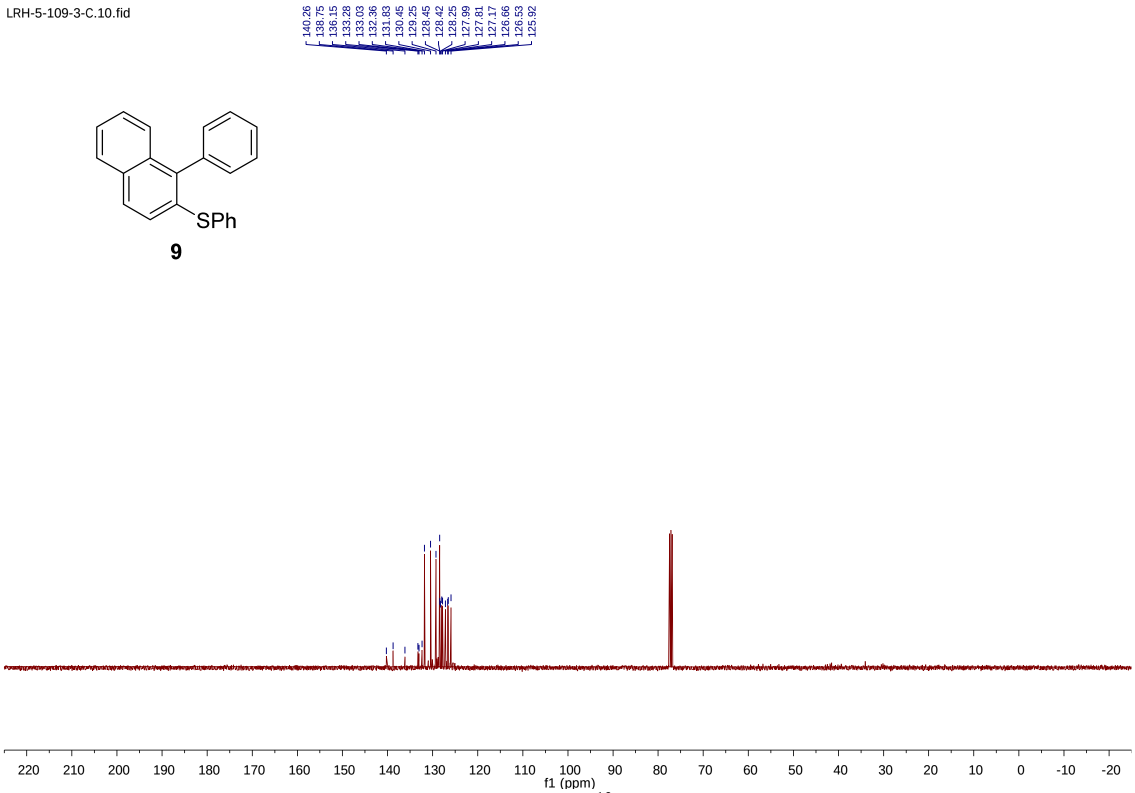

Supplementary Figure 147. ^{13}C NMR Spectrum of **9**

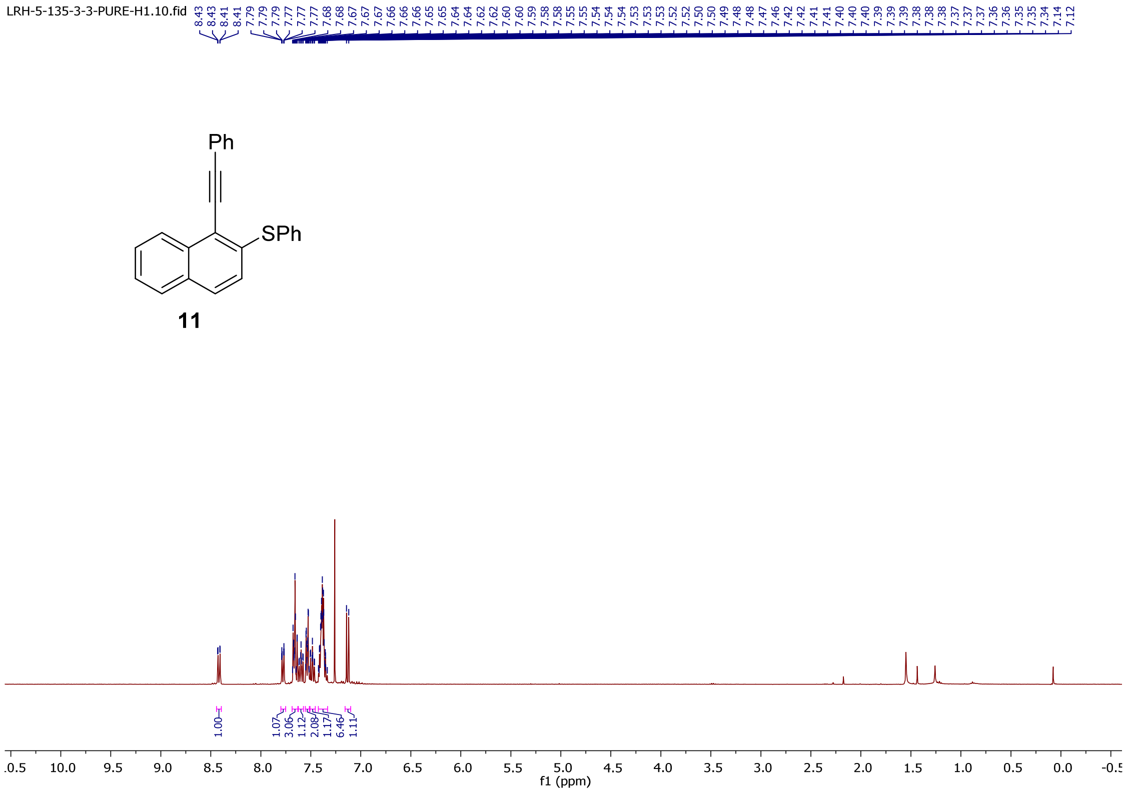

Supplementary Figure 148. ^{1}H NMR Spectrum of **11**

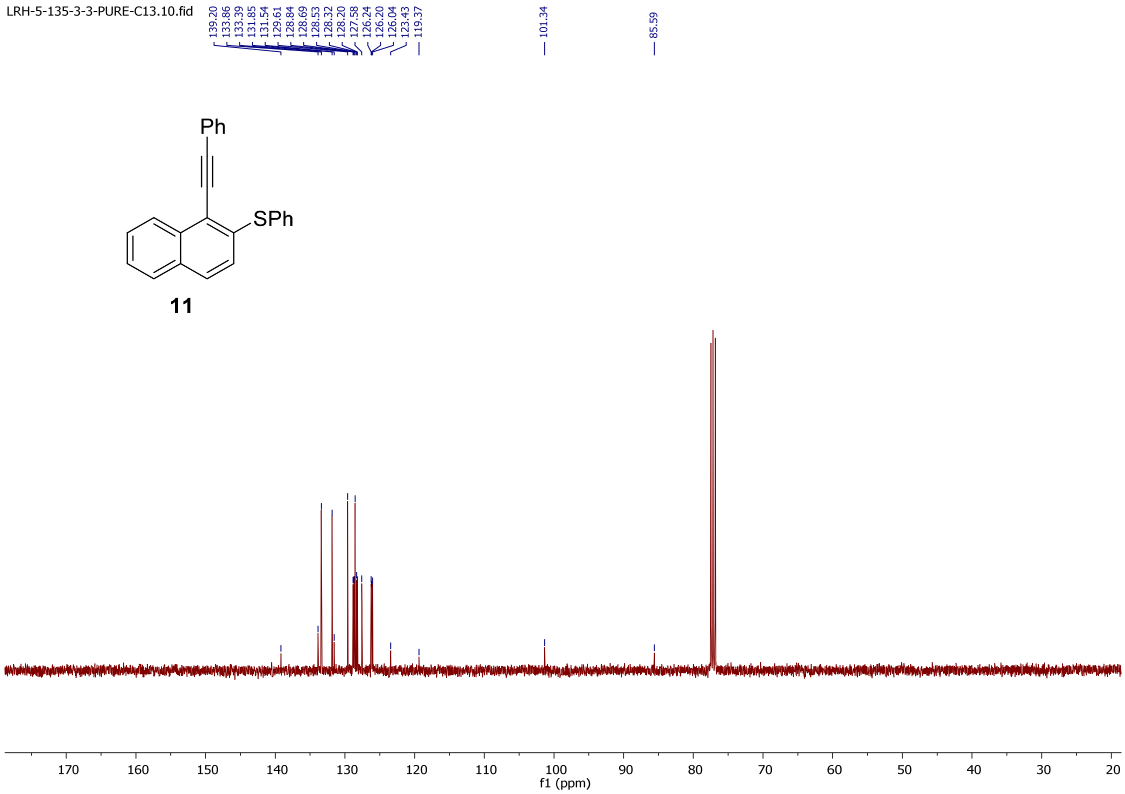

Supplementary Figure 149. ^{13}C NMR Spectrum of **11**

Supplementary References

1. Burkhardt, I. & Dickschat, J. S. Synthesis and Absolute Configuration of Natural 2-Pyrones. *Eur. J. Org. Chem.* **2018**, 3144-3157 (2018).
2. Duffault, J.-M. & Tellier, F. A New Route to the Synthesis of Bicyclo [3.3.2] Nonene by Radical Cyclisation. *Synth. Commun.* **28**, 2467-2481 (1998).
3. Bao, M., Shimizu, M., Shimada, S. & Tanaka, M. Efficient synthesis of N-acylarenesulfenamides by acylation of arenesulfenamides. *Tetrahedron* **59**, 303-309 (2003).
4. Biallas, P., Mensak, T. M., Kunz, K.-A. & Kirsch, S. F. The Deazidoalkoxylation: Sequential Nucleophilic Substitutions with Diazidated Diethyl Malonate. *J. Org. Chem.* **84**, 1654-1663 (2019).
5. Esker, J. L. & Newcomb, M. Amidyl radicals from N-(phenylthio)amides. *Tetrahedron Lett.* **34**, 6877-6880 (1993).
6. Gaykar, R. N., Bhattacharjee, S. & Biju, A. T. Transition-Metal-Free Thioamination of Arynes Using Sulfenamides. *Org. Lett.* **21**, 737-740 (2019).
7. Lee, C., Lim, Y. N. & Jang, H.-Y. Copper-Catalyzed Synthesis of N-Formyl/Acylsulfenamides and -thiosulfonamides. *Eur. J. Org. Chem.* **2015**, 5934-5938 (2015).
8. Winter, D. K., Drouin, A., Lessard, J. & Spino, C. Photochemical Rearrangement of N-Chlorolactams: A Route to N-Heterocycles through Concerted Ring Contraction. *J. Org. Chem.* **75**, 2610-2618 (2010).
9. Winnacker, M., Tischner, A., Neumeier, M. & Rieger, B. New insights into synthesis and oligomerization of ε-lactams derived from the terpenoid ketone (−)-menthone. *RSC Adv.* **5**, 77699-77705 (2015).
10. Li, Z.-S. *et al.* Synthesis and biological evaluation of nonsymmetrical aromatic disulfides as novel inhibitors of acetohydroxyacid synthase. *Biorg. Med. Chem. Lett.* **23**, 3723-3727 (2013).
11. Ramalingan, C. & Park, Y.-T. Mercury-Catalyzed Rearrangement of Ketoximes into Amides and Lactams in Acetonitrile. *J. Org. Chem.* **72**, 4536-4538 (2007).
